# Photochemical Functionalization of 4‑Diazoisoquinoline-1,3(2*H*,4*H*)‑diones and Their 1‑Sulfoxide Analogues

**DOI:** 10.1021/acsorginorgau.5c00017

**Published:** 2025-03-24

**Authors:** Joseph P. Milton, Dorota Gryko

**Affiliations:** 154690 Institute of Organic Chemistry, Polish Academy of Sciences, Warsaw 01-224, Poland

**Keywords:** photochemistry, photolysis, diazo compounds, insertion reactions, isoquinoline-1,3(2*H*,4*H*)-diones

## Abstract

Isoquinoline-1,3­(2*H*,4*H*)-diones have been the subject of numerous studies toward new pharmaceuticals, and therefore novel, mild functionalizations of this scaffold are highly desirable. As diazo compounds are versatile reagents in organic synthesis, 4-diazoisoquinoline-1,3­(2*H*,4*H*)-diones should give access to a broad range of various derivatives. Indeed, they enable the introduction of fluorinated moieties via photochemical O–H insertion reactions in serviceable yields, typically in under 2 h. Exchanging the 1-carbonyl group with the sulfoxide moiety causes a hypsochromic shift in the absorption of the diazo compounds, and thus, violet light is required for effective O–H and S–H insertion reactions.

## Graphical abstract

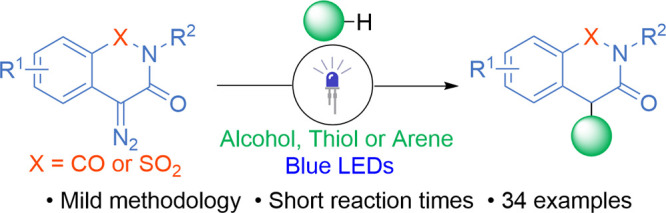

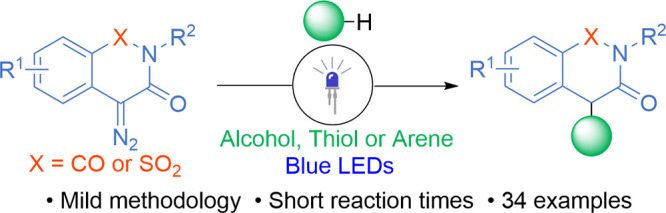

Heterocyclic scaffolds are highly prevalent in natural compounds, drugs, and lead candidates.^1^ Among them, isoquinoline-1,3­(2*H*,4*H*)-diones draw particular interest in biological assays, where they have been probed as inhibitors of HIV-1 integrase^2,3^ and ALR2,^4^ antagonists for progesterone receptors, among others in recent years (Figure 1A).^5–8^ In fact, they have been investigated as potential hypnotic agents since the 1920’s,^9^ illustrating that interest in this class of compounds has been constant over the last 100 years. Nevertheless, the development of mild, efficient, and practical modifications of this skeleton still presents a challenge and is highly desirable.^10^

**Figure 1:**
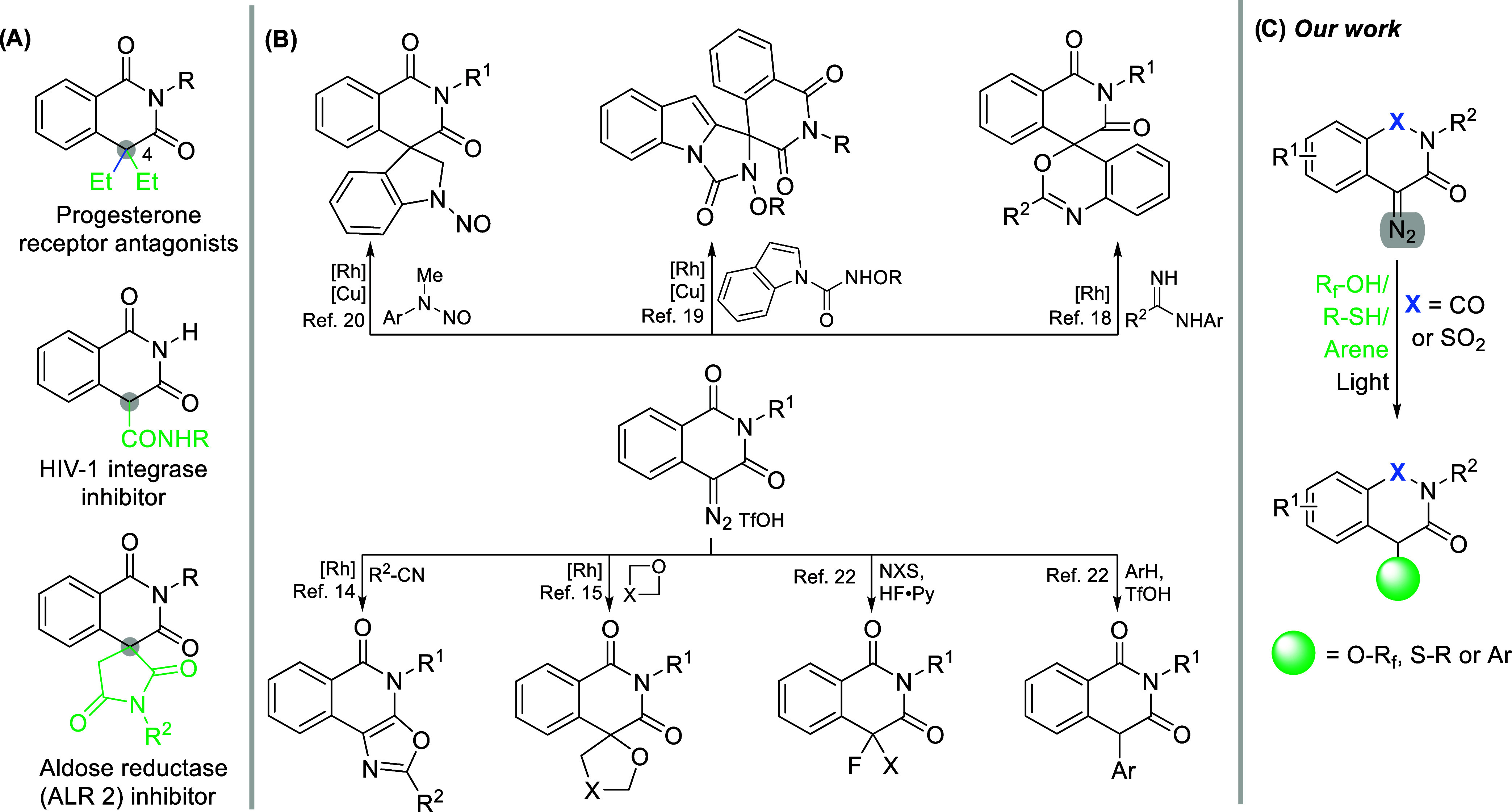
(A) Examples of bioactive isoquinoline-1,3­(2*H*,4*H*)-diones, (B) known reactions of 4-diazoisoquinoline-1,3­(2*H*,4*H*)-diones, and (C) our work.

With the well-documented impact of fluorine on the pharmacokinetic and physicochemical properties of a compound and approximately 20% of pharmaceuticals containing a fluorinated moiety,^11–13^ *we sought a straightforward methodology to introduce fluorinated moi* *eties into the isoquinoline-1,3­(2H,4H)-dione scaffold.*

In the past decade, reactions of 4-diazoisoquinoline-1,3­(2*H*,4*H*)-diones have only been probed with a rhodium catalyst in combination with nitriles,^14^ heterocycles,^15^ and carboxylic acids^16,17^ by Krasavin and Dar’in (Figure 1B). Recently, the Fan group has used them to synthesize a variety of spirocyclic heterocycles under either Rh or dual catalytic conditions (Rh and Cu).^18–20^ One of these diazo compound was tested in a three-component acyloxylation reaction with a ruthenium catalyst.^21^ Eliminating the need for transition metals, it has been shown that strong acids such as hydrofluoric and triflic acid enable the introduction of fluorine atoms or aryl moieties to the 4-position.^22^ On the other hand, some closely related heterocyclic diazo compounds such as 3-diazooxindoles^23–26^ and 4-diazo-2-tosyl-1,4-dihydroisoquinolin-3­(2*H*)-ones^27^ are known to react effectively under blue light-emitting diode (LED) conditions. With our previous experience in the photochemistry of diazo compounds,^28–30^ we envisaged *that 4-diazoisoquinoline-1,3­(2H,4H)-diones should productively react with fluorinated alcohols under mild photochemical conditions*.

Changes at the 4-position of isoquinoline-1,3­(2*H*,4*H*)-diones have a pronounced impact on their pharmaceutical activity (Figure 1A). As the 1,1,1,3,3,3-hexafluoroisopropoxy moiety has already provided promising results in biological screenings,^31–34^ we decided to start our investigation with 4-diazo-2-methylisoquinoline-1,3­(2*H*,4*H*)-dione (**1a**) with 1,1,1,3,3,3-hexafluoro-2-propanol (HFIP) under blue LED irradiation to undergo the classical photochemical O–H insertion reaction.^30,35,36^

Using a 2:8 solution of EtOAc and HFIP enabled the formation of the desired product **2**, but in a low yield (17% by NMR, Table 1, entry 1). The more HFIP we used, the better the yield became; thus, the reaction was performed in neat HFIP (entries 2 and 3); reducing the concentration did not improve the yield to any substantial effect (entry 4) but increasing it to 0.2 M or higher started to have adverse effects (entries 5 and 6). Kinetics studies showed that a yield of 80% was consistently achieved for the timespan of 1–4 h; however, the product slowly decomposes when exposed to prolonged irradiation (see SI Figure 2.3). After 2 h, product **2** was isolated in 67% yield (entry 7).

**Table 1:**
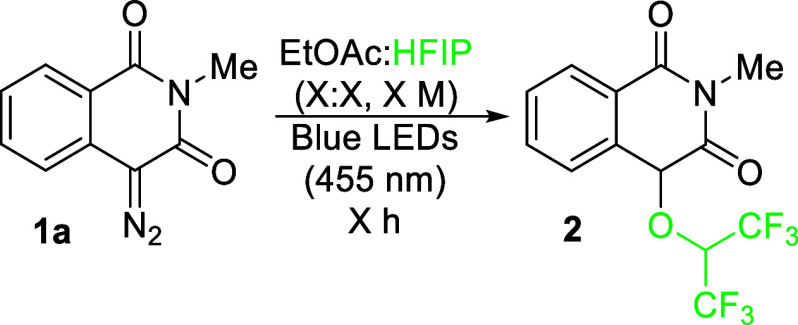
Optimization Studies^a^

entry	HFIP/EtOAc	*c* (M)	time (h)	yield (%)^b^
1	2:8	0.1	18	17
2	6:4	0.1	18	45
3	1:0	0.1	18	60
4	1:0	0.05	18	57
5	1:0	0.2	18	53
6	1:0	0.4	18	46
7	1:0	0.1	2	80 (67)^c^

a **1a** (0.1 mmol), blue LEDs (455 nm).

b NMR yields determined with trichloroethene as the internal standard.

c Isolated yield.

Other *N*-alkyl-substituted 4-diazoisoquinoline-1,3­(2*H*,4*H*)-diones performed relatively consistently, allowing fluorinated derivatives to be synthesized in 60–71% yield (Scheme 1, **2–6**). However, the reaction with 4-diazo-2-phenylisoquinoline-1,3­(2*H*,4*H*)-dione led to product **7** in a diminished yield because of the lower stability of the substrate and product under the reaction conditions. Furthermore, the presence of electron-withdrawing or electron-donating substituents at position 7 of the aromatic ring slightly diminishes the reaction yield in comparison to the unsubstituted analogues (**8**–**10**). In the case of the 6- and 7-methoxy derivatives, prolongation of the reaction time was required to achieve products **11** and **12** in serviceable yield. Intriguingly, the presence of halogens at the 6-position destabilizes these compounds (**13** and **14**), and thus, they could not be successfully isolated. A similar issue was encountered with compound **15**, which possesses a chlorine atom with an analogous electron-withdrawing effect, which gave a diminished yield, highlighting the problematic nature of the electron-withdrawing substituents in the *ortho*-position. Furthermore, reactions with other fluorinated alcohols were less efficient than HFIP (**17** – **21**); however, the reaction with 1,1,1-trifluoroisopropanol provided a satisfactory diastereomeric ratio of 1:10 (**18**). The O–H insertion product with nonafluoro-*tert*-butyl alcohol (**21a**) underwent an interesting rearrangement reaction to a ketone forming the respective trione compound (**21b**) under silica gel column chromatography.

**Scheme 1:**
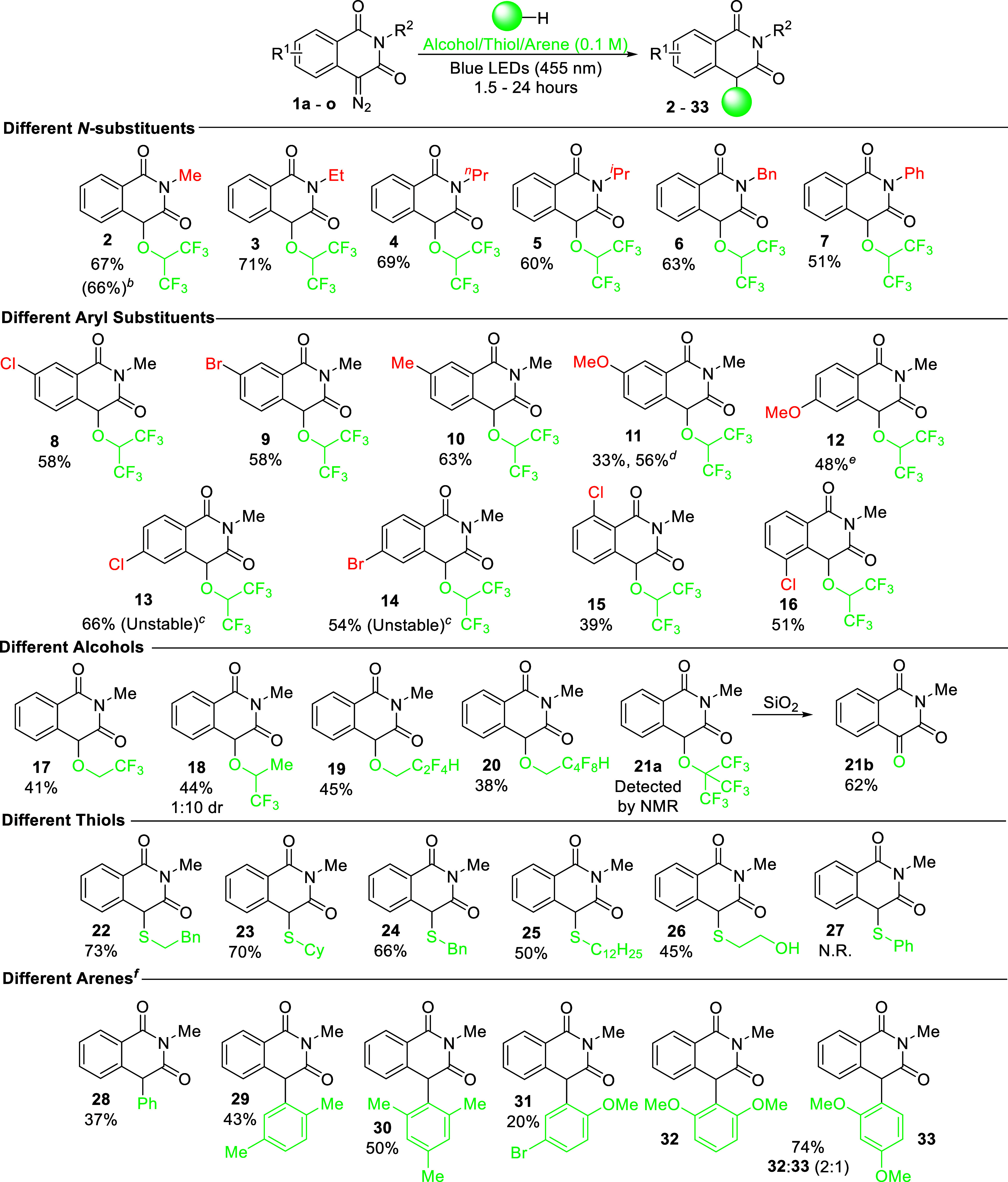
Scope of Insertion Reactions with 4-Diazoisoquinoline-1,3­(2*H*,4*H*)-diones^f^

a Reaction conditions: 4-diazoisoquinoline-1,3­(2*H*,4*H*)-dione (0.2 mmol), alcohol or thiol (0.1 M), blue LEDs (455 nm), 1.5 h, unless otherwise stated, isolated yields are given.
b Gram-scale reaction, 7 h.
c 0.1 mmol scale reaction, yield determined by ^19^F NMR with methyl 4-(trifluoromethyl)­benzoate as the internal standard, the product was not isolated.
d 4.5 h.
e 20 h.
f 4-Diazo-2-methylisoquinoline-1,3­(2*H*,4*H*)-dione (0.2 mmol), arene (0.1 M), blue LEDs (455 nm), 24 h.

For more typical alcohols, such as ethanol and isopropanol, no product formation was observed, which might suggest that the mechanism reported by Koenigs and co-workers may operate instead of a formal carbene O–H insertion. They proposed that the relatively acidic HFIP coordinates to the α-carbonyl moiety, which upon light irradiation triggers a photoexcited proton transfer to generate an unstable diazonium-like intermediate, which is immediately attacked by the deprotonated alcohol. This would also justify why other examined fluorinated alcohols give lower yields, as they are less acidic and, thus, the coordination to the amide would be weaker.^24,37^

The introduction of fluorinated moieties is easily scalable and is demonstrated by performing the reaction on a gram scale (5.0 mmol) with diazo compound **1a** in 50 mL of HFIP, with an extended reaction time of 7 h that yielded over 1 g of product **2** in 66% yield, a comparable yield to the smaller-scale reaction. With HFIP still being a commodity solvent^34,38^ and having a relatively high cost, we recycled the solvent (>80% efficiency) by distilling the excess after the reaction completion.

With the photochemical insertion of O–H working successfully, we tested the developed method for similar reactions such as S–H and C–H insertions.

Indeed, all thiols examined performed well, especially 2-phenylethyl mercaptan and cyclohexanethiol (**22** and **23**, respectively). 2-Mercaptoethanol (**26**) gave high chemoselectivity with only exclusive formation of the S–H insertion product; we presume this is due to nonfluorinated alcohols being unproductive in the reaction, whereas alkyl thiols perform effectively. Furthermore, reactions with aromatic solvents provided C–H insertion products as the main constituents of the product mixture (Scheme 1). The yield gradually improved with an increased electron richness of the aromatic ring from benzene (**28**, 37%), *p*-xylene (**29**, 43%) to mesitylene (**30**, 50%). 4-Bromoanisole, despite having a strong electron-donating methoxy group, gave a poor yield of only 20%, showing that the electron-withdrawing bromine group significantly affects the reactivity (**31**). 1,3-Dimethoxybenzene provided the highest yield of arenes tested, which afforded a statistical amount of the two regioisomers despite the steric hindrance present at the 2-position (**32** and **33**). While the yields are generally relatively moderate with arenes, Golushko et al. reported that such a reaction only provides traces of material under microwave-assisted rhodium-catalyzed conditions.^22^ Alternative methods for such a transformation either involve the use of triflic acid^22^ or a palladium cross-coupling,^39^ thus demonstrating the mildness of the developed photochemical reaction. We also attempted to perform N–H insertion reactions, but a rearrangement reaction takes place that is analogous to that described by Li et al.^40^ and similar to works by Wakchaure et al.^41,42^

2*H*-Benzo­[*e*]­[1,2]­thiazin-3­(4*H*)-one 1,1-dioxide, a closely related heterocycle, where, effectively, the 1-carbonyl group is replaced with a sulfoxide moiety, has previously been reported to possess impressive anti-inflammatory properties, some displaying even higher activity than indomethacin,^43^ which is part of the WHO list of essential medicines.^44^ We, thus, examined this novel series of diazo compounds (**34a–c**) in our new method to introduce fluorinated moieties.

Ultraviolet–visible (UV–vis) spectra of these new diazo compounds are similar in shape to the 4-diazoisoquinoline-1,3­(2*H*,4*H*)-dione (**1a**) but the λ_max_ is hypsochromically shifted approximately 35 nm (Figure 2); consequently, violet LED irradiation instead of blue LEDs is preferable for their activation. The O–H insertion reaction of diazo compound **34a** with HFIP formed the desired product (**35**) in 53% yield with violet LEDs in 1.5 h; blue LED irradiation led to product **35** in a comparable yield (52%), with an extended reaction time (2.5 h, Scheme 2).

**Figure 2:**
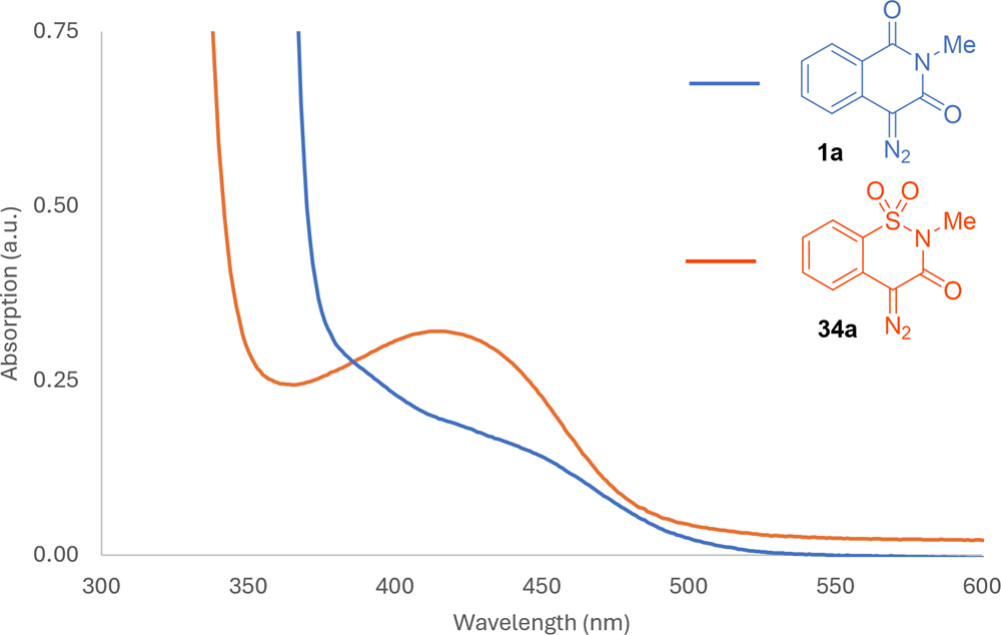
UV–vis spectra of 4-diazo-2-methylisoquinoline-1,3­(2*H*,4*H*)-dione (**1a**) and 4-diazo-2-methyl-2*H*-benzo­[*e*]­[1,2]­thiazin-3­(4*H*)-one 1,1-dioxide (**34a**) (*c* = 0.006 M in DCM).

**Scheme 2:**
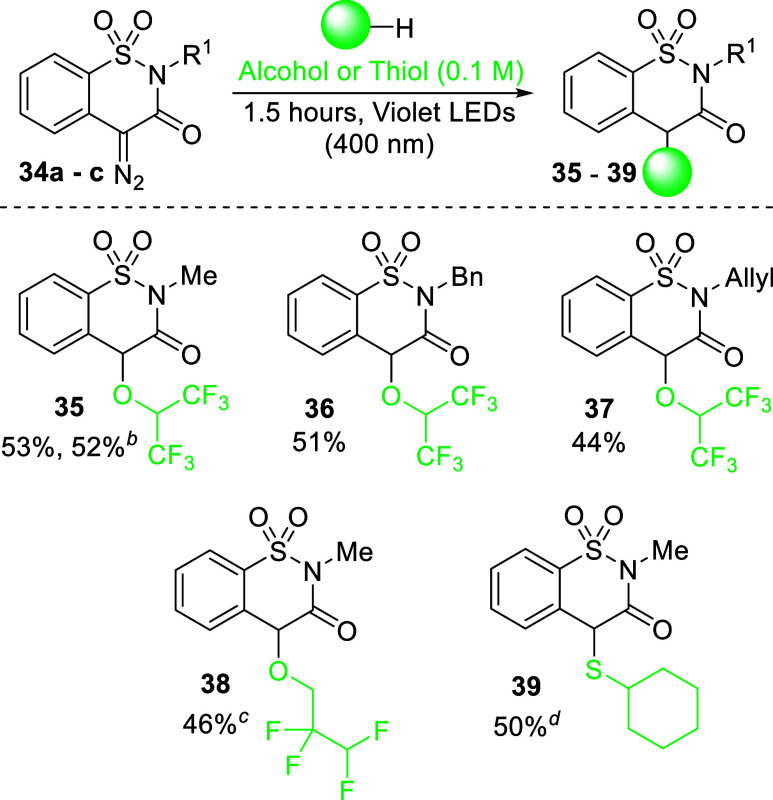
Scope of O–H and S–H Insertions with 4-Diazo-2*H*-benzo­[*e*]­[1,2]­thiazin-3­(4*H*)-one 1,1-Dioxides^d^

a Reaction conditions: 4-diazo-2*H*-benzo­[*e*]­[1,2]­thiazin-3­(4*H*)-one 1,1-dioxide (0.15 mmol), alcohol or thiol (0.1 M), violet LEDs (400 nm), 1.5 h.
b Blue LEDs (455 nm), 2.5 h.
c 4 h.
d 7 h.

Similar reactivity was observed with other *N*-substituted derivatives, such as *N*-benzyl (**36**, 51%) and *N*-allyl (**37**, 44%). The X–H insertion reaction was also viable with 2,2,3,3-tetrafluoropropan-1-ol (**38**, 46%) and cyclohexanethiol (**39**, 50%) in similar moderate yields but both required extended reaction times of 4 and 7 h, respectively. In brief, this new series of diazo compounds is generally less reactive compared to 4-diazoisoquinoline-1,3­(2*H*,4*H*)-diones while requiring a different light source and an extended reaction time in more situations.

In conclusion, fluorinated moieties can be effectively introduced into the isoquinoline-1,3­(2*H*,4*H*)-dione scaffold under mild blue LED irradiation. Gratifyingly, the reaction is compatible up to a gram scale with HFIP as the reagent and solvent and is also recyclable and scalable. The developed conditions are also compatible with S–H and C–H insertion reactions. A novel series of diazo compounds, 4-diazo-2*H*-benzo­[*e*]­[1,2]­thiazin-3­(4*H*)-one 1,1-dioxides, were synthesized and efficiently react under violet LED irradiation, providing O–H and S–H insertion reactions in slightly diminished yields compared to 4-diazoisoquinoline-1,3­(2*H*,4*H*)-diones. We believe that this work will provide a useful stepping stone for further investigations of photochemical reactions of heterocyclic diazo compounds.

## Experimental Section

### General Procedures

All solvents and commercially available reagents were used as purchased without further purification. Dry solvents were obtained from a Solvent Purification System (SPS). All reactions were monitored by gas chromatography (GC) or TLC on Merck silica gel (GF254, 0.20 mm thickness) and were visualized with UV light. Column chromatography was performed using Merck silica gel 60 (230–400 mesh). Unless otherwise noted, all reactions were performed without the exclusion of air or moisture. Unless otherwise noted, all photochemical reactions were performed in 10 mL vials with an aluminum cap and a rubber septum.

^1^H and ^13^C NMR spectra were recorded at 25 °C on a Bruker 400 MHz, Bruker 500 MHz, Varian 500 MHz or a Varian 600 MHz instrument. NMR chemical shifts are reported in ppm and referenced to the residual solvent peak: ^1^H NMR 7.26 ppm (CDCl_3_) or 2.50 ppm (DMSO-*d*_6_); ^13^C NMR 77.16 ppm (CDCl_3_) or 39.52 ppm (DMSO-*d*_6_). In cases where the CDCl_3_ peak could not be identified, TMS was instead used as the reference at 0.00 ppm. Multiplicities are indicated by singlet (s), doublet (d), triplet (t), quartet (q), pentet (p), heptet (hept), and multiplet (m). Coupling constants (*J*) are reported in hertz. All data analyses were performed using the MestReNova software package. Elemental analysis (C, H, N, S, Br, Cl, and F) was performed on a PerkinElmer 240 Elemental Analyzer. High-resolution mass spectra were recorded on a Waters SYNAPT G2-S HDMS using electrospray ionization (ESI) or atmospheric-pressure chemical ionization (APCI) with a time-of-flight (TOF) detector. Gas chromatography analysis coupled with a flame ionization detector (GC-FID) was performed on a Shimadzu GCMS-QP2010 SE with helium as the carrier gas and a Zebron ZB 5MSi column.

### General Procedure for the Insertion Reactions of 4-Diazoisoquinoline-1,3­(2*H*,4*H*)-dione

4-Diazoisoquinoline-1,3­(2*H*,4*H*)-dione (0.2 mmol) is charged in a vial and dissolved in the respective alcohol, thiol, or arene (2 mL), and then the vial is capped. The vial is irradiated with blue LEDs for typically 1.5 h (24 h with arenes) at approximately 15 °C and then the cap is removed.

#### For Volatile Solvents

The solution is transferred to a flask with DCM and all of the volatiles are removed in vacuo. The crude residue is purified by column chromatography (SiO_2_, 10% EtOAc in hexane) to afford the X–H insertion product.

#### For Nonvolatile Solvents

The crude reaction mixture is pipetted directly on top of a prepacked column and subjected to column chromatography (SiO_2_, 10% EtOAc in hexane) to afford the X–H insertion product.

### General Procedure for the Insertion Reactions of 4-Diazo-2*H*-benzo­[*e*]­[1,2]­thiazin-3­(4*H*)-one 1,1-dioxides

4-Diazo-2*H*-benzo­[*e*]­[1,2]­thiazin-3­(4*H*)-one 1,1-dioxide (0.15 mmol) is charged in a vial and dissolved in the respective alcohol or thiol (1.5 mL), and then the vial is capped. The vial is irradiated with violet LEDs for 1.5 h at approximately 15 °C, then the reaction mixture is transferred to a flask with DCM, and all of the volatiles are removed in vacuo. The crude residue is purified by column chromatography (SiO_2_, 10% EtOAc in hexane) to afford the X–H insertion product.

## Supplementary Material



## Data Availability

The data underlying this study are available in the published article and the Supporting Information.

## Notes

Experimental details and procedures; optimization studies; and spectral data for all new compounds (PDF)
