# Supplementary material for: Photochemical Functionalization of 4‑Diazoisoquinoline-1,3(2H,4H)‑diones and Their 1‑Sulfoxide Analogues
Source: ACS Org Inorg Au. 2025 Mar 24;5(3):205–10. doi: 10.1021/acsorginorgau.5c00017 (PMC12142437; doi:10.1021/acsorginorgau.5c00017)

SUPPORTING INFORMATION FOR:

PHOTOCHEMICAL FUNCTIONALIZATION OF 4-DIAZOISOQUINOLINE-1,3(2*H*,4*H*)-DIONES AND THEIR 1-SULFOXIDE ANALOGS

Joseph P. Milton, Dorota Gryko*

Institute of Organic Chemistry, Polish Academy of Sciences, Kasprzaka 44/52, 01-224, Warsaw, Poland

**Correspondence: dorota.gryko@icho.edu.pl*

Table of Contents

1. General Information 5

2. Optimisation of the HFIP Insertion with 4-Diazo-2-methylisoquinoline-1,3(2*H*,4*H*)-dione 7

2.1. Amount of HFIP vs. Reaction Yield 7

2.2. Effect of Concentration on the Reaction Yield 8

2.3. Reaction Profile over Time 8

2.4. Assessing the Reaction with DCM 9

3. UV-Vis Spectra of Diazo Compounds 10

3.1. Influence of *N*-substituents 10

3.2. Influence of the Position of Cl-Substituents on the Aryl Ring 10

3.3. Influence of Substituents at the 7-Position 11

3.4. UV-Vis of Sulfoxide-Containing Diazo Compounds 11

3.5. Comparing the Two Families of Diazo Compounds 12

4. Mechanistic Considerations 13

5. General Procedures 14

5.1. General Procedure A - Synthesis of Substituted (2-(Carboxymethyl)benzoic acids) 14

5.2. General Procedure B - Synthesis of Substituted *N*-Methylisoquinoline-1,3(2*H*,4*H*)-diones 14

5.3. General Procedure C - Synthesis of 2*H*-Benzo[*e*][1,2]thiazin-3(4*H*)-one 1,1-dioxides 15

5.4. General Procedure D - Synthesis of Diazo Compounds 16

5.5. General Procedure E - O-H and S-H Insertion Reactions of 4-Diazoisoquinoline-1,3(2*H*,4*H*)-diones 16

5.6. General Procedure F – Arene Insertion Reactions of 4-Diazo-2-methylisoquinoline-1,3(2*H*,4*H*)-diones 17

5.7. General Procedure G - O-H and S-H Insertion Reactions of 4-Diazo-2*H*-benzo[*e*][1,2]thiazin-3(4*H*)-one 1,1-dioxides 17

6. (2-(Carboxymethyl))benzoic acids 18

7. Isoquinoline-1,3(2*H*,4*H*)-diones 21

8. 2*H*-Benzo[*e*][1,2]thiazin-3(4*H*)-one 1,1-dioxides 30

9. 4-Diazo-2-methylisoquinoline-1,3(2H,4H)-diones 33

10. 4-Diazo-2*H*-benzo[*e*][1,2]thiazin-3(4*H*)-one 1,1-dioxide 38

10. O-H Insertion Products 40

11. S-H Insertion Products 58

12. Arene Insertions 62

13. Reactions of 4-diazo-2*H*-benzo[*e*][1,2]thiazin-3(4*H*)-one 1,1-dioxides 66

14. References 71

15. NMR Spectra 73

4-methoxy-2-methylbenzoic acid (**S1**) ... 73
2-(carboxymethyl)-4-methoxybenzoic acid (**S2**) ... 74
2-(carboxymethyl)-5-chlorobenzoic acid (**S3**) ... 75
2-(carboxymethyl)-3-chlorobenzoic acid (**S4**) ... 76
4-bromo-2-(carboxymethyl)benzoic acid (**S5**) ... 77
7-chloro-2-methylisoquinoline-1,3(2*H*,4*H*)-dione (**S6**) ... 78
7-bromo-2-methylisoquinoline-1,3(2*H*,4*H*)-dione (**S7**) ... 79
2,7-dimethylisoquinoline-1,3(2*H*,4*H*)-dione (**S8**) ... 80
7-methoxy-2-methylisoquinoline-1,3(2*H*,4*H*)-dione (**S9**) ... 81
6-methoxy-2-methylisoquinoline-1,3(2*H*,4*H*)-dione (**S10**) ... 82
6-chloro-2-methylisoquinoline-1,3(2*H*,4*H*)-dione (**S11**) ... 83
6-bromo-2-methylisoquinoline-1,3(2*H*,4*H*)-dione (**S12**) ... 84
8-chloro-2-methylisoquinoline-1,3(2*H*,4*H*)-dione (**S13**) ... 85
5-chloro-2-methylisoquinoline-1,3(2*H*,4*H*)-dione (**S14**) ... 86
2-methyl-2*H*-benzo[*e*][1,2]thiazin-3(4*H*)-one 1,1-dioxide (**S15**) ... 87
2-benzyl-2*H*-benzo[*e*][1,2]thiazin-3(4*H*)-one 1,1-dioxide (**S16**) ... 88
2-allyl-2*H*-benzo[*e*][1,2]thiazin-3(4*H*)-one 1,1-dioxide (**S17**) ... 89
7-chloro-4-diazo-2-methylisoquinoline-1,3(2*H*,4*H*)-dione (**1g**) ... 90
7-bromo-4-diazo-2-methylisoquinoline-1,3(2*H*,4*H*)-dione (**1h**) ... 91
4-diazo-2,7-dimethylisoquinoline-1,3(2*H*,4*H*)-dione (**1i**) ... 92
4-diazo-7-methoxy-2-methylisoquinoline-1,3(2*H*,4*H*)-dione (**1j**) ... 93
4-diazo-6-methoxy-2-methylisoquinoline-1,3(2*H*,4*H*)-dione (**1k**) ... 94
6-chloro-4-diazo-2-methylisoquinoline-1,3(2*H*,4*H*)-dione (**1l**) ... 95
6-bromo-4-diazo-2-methylisoquinoline-1,3(2*H*,4*H*)-dione (**1m**) ... 96
8-chloro-4-diazo-2-methylisoquinoline-1,3(2*H*,4*H*)-dione (**1n**) ... 97
5-chloro-4-diazo-2-methylisoquinoline-1,3(2*H*,4*H*)-dione (**1o**) ... 98
2-methyl-4-diazo-2H-benzo[*e*][1,2]thiazin-3(4*H*)-one 1,1-dioxide (**34a**) ... 99
2-benzyl-4-diazo-2H-benzo[*e*][1,2]thiazin-3(4*H*)-one 1,1-dioxide (**34b**) ... 100
4-diazo-2-allyl-2*H*-benzo[*e*][1,2]thiazin-3(4*H*)-one 1,1-dioxide (**34c**) ... 101
4-((1,1,1,3,3,3-hexafluoropropan-2-yl)oxy)-2-methylisoquinoline-1,3(2*H*,4*H*)-dione (**2**) ... 102
2-ethyl-4-((1,1,1,3,3,3-hexafluoropropan-2-yl)oxy)isoquinoline-1,3(2*H*,4*H*)-dione (**3**) ... 103
4-((1,1,1,3,3,3-hexafluoropropan-2-yl)oxy)-2-propylisoquinoline-1,3(2*H*,4*H*)-dione (**4**) ... 105
4-((1,1,1,3,3,3-hexafluoropropan-2-yl)oxy)-2-isopropylisoquinoline-1,3(2*H*,4*H*)-dione (**5**) ... 106
2-benzyl-4-((1,1,1,3,3,3-hexafluoropropan-2-yl)oxy)isoquinoline-1,3(2*H*,4*H*)-dione (**6**) ... 108

4-((1,1,1,3,3,3-hexafluoropropan-2-yl)oxy)-2-phenylisoquinoline-1,3(2*H*,4*H*)-dione (**7**)......... 109

7-chloro-4-((1,1,1,3,3,3-hexafluoropropan-2-yl)oxy)-2-methylisoquinoline-1,3(2*H*,4*H*)-dione (**8**) 111

7-bromo-4-((1,1,1,3,3,3-hexafluoropropan-2-yl)oxy)-2-methylisoquinoline-1,3(2*H*,4*H*)-dione (**9**) 112

4-((1,1,1,3,3,3-hexafluoropropan-2-yl)oxy)-2,7-dimethylisoquinoline-1,3(2*H*,4*H*)-dione (**10**). 114

4-((1,1,1,3,3,3-hexafluoropropan-2-yl)oxy)-7-methoxy-2-methylisoquinoline-1,3(2*H*,4*H*)-dione (**11**).. 115

4-((1,1,1,3,3,3-hexafluoropropan-2-yl)oxy)-6-methoxy-2-methylisoquinoline-1,3(2*H*,4*H*)-dione (**12**).. 117

8-chloro-4-((1,1,1,3,3,3-hexafluoropropan-2-yl)oxy)-2-methylisoquinoline-1,3(2*H*,4*H*)-dione (**15**).. 118

5-chloro-4-((1,1,1,3,3,3-hexafluoropropan-2-yl)oxy)-2-methylisoquinoline-1,3(2*H*,4*H*)-dione (**16**).. 120

2-methyl-4-(2,2,2-trifluoroethoxy)isoquinoline-1,3(2*H*,4*H*)-dione)(**17**)................................ 121

2-methyl-4-((1,1,1-trifluoropropan-2-yl)oxy)isoquinoline-1,3(2*H*,4*H*)-dione (**18**) (dr 1:10)...... 123

2-methyl-4-(2,2,3,3-tetrafluoropropoxy)isoquinoline-1,3(2*H*,4*H*)-dione (**19**) 124

2-methyl-4-((2,2,3,3,4,4,5,5-octafluoropentyl)oxy)isoquinoline-1,3(2*H*,4*H*)-dione (**20**) 126

2-methylisoquinoline-1,3,4(2*H*)-trione (**21b**) 128

2-methyl-4-(phenethylthio)isoquinoline-1,3(2*H*,4*H*)-dione (**22**).. 129

4-(cyclohexylthio)-2-methylisoquinoline-1,3(2*H*,4*H*)-dione (**23**) 130

4-(benzylthio)-2-methylisoquinoline-1,3(2*H*,4*H*)-dione (**24**) 131

4-(dodecylthio)-2-methylisoquinoline-1,3(2*H*,4*H*)-dione (**25**) 132

4-((2-hydroxyethyl)thio)-2-methylisoquinoline-1,3(2*H*,4*H*)-dione (**26**).................................. 133

2-methyl-4-phenylisoquinoline-1,3(2*H*,4*H*)-dione (**28**)... 134

4-(2,5-dimethylphenyl)-2-methylisoquinoline-1,3(2*H*,4*H*)-dione (**29**) 135

4-mesityl-2-methylisoquinoline-1,3(*2H,*4*H*)-dione (**30**) 136

4-(5-bromo-2-methoxyphenyl)-2-methylisoquinoline-1,3(2*H*,4*H*)-dione (**31**) 137

4-((1,1,1,3,3,3-hexafluoropropan-2-yl)oxy)-2-methyl-2H-benzo[*e*][1,2]thiazin-3(4*H*)-one 1,1-dioxide (**35**)... 139

2-benzyl-4-((1,1,1,3,3,3-hexafluoropropan-2-yl)oxy)-2H-benzo[e][1,2]thiazin-3(4H)-one 1,1-dioxide (**36**)... 140

2-allyl-4-((1,1,1,3,3,3-hexafluoropropan-2-yl)oxy)-2*H*-benzo[*e*][1,2]thiazin-3(4*H*)-one 1,1-dioxide (**37**)... 142

2-methyl-4-(2,2,3,3-tetrafluoropropoxy)-2H-benzo[*e*][1,2]thiazin-3(4*H*)-one 1,1-dioxide (**38**) 143

4-(cyclohexylthio)-2-methyl-2H-benzo[*e*][1,2]thiazin-3(4*H*)-one 1,1-dioxide (**39**) 145

1. General Information

General - All solvents and commercially available reagents were used as purchased without any further purification. Dry solvents were obtained from a Solvent Purification System (SPS). All reactions were monitored by gas chromatography (GC) or TLC on Merck silica gel (GF254, 0.20 mm thickness) and were visualised with UV-light. Column chromatography was performed using Merck silica gel 60 (230 – 400 mesh). Unless otherwise noted, all reactions were performed without the exclusion of air or moisture. Unless otherwise noted, all photochemical reactions were performed in 10 mL vials with an aluminium cap with a rubber septum.

NMR - ^{1}H and $^{13}C\{^{1}H\}$ NMR spectra were recorded at 25 °C on a Bruker 400 MHz, Bruker 500 MHz, Varian 500 MHz or a Varian 600 MHz instrument. NMR chemical shifts are reported in ppm and referenced to the residual solvent peak: ^{1}H NMR - 7.26 ppm ($CDCl_3$) or 2.50 ppm (DMSO-d_6); $^{13}C\{^{1}H\}$ NMR - 77.16 ppm ($CDCl_3$) or 39.52 ppm (DMSO-d_6). In cases where the $CDCl_3$ peak could not be identified, TMS was instead used as the reference at 0.00 ppm. Multiplicities are indicated by singlet (s), doublet (d), triplet (t), quartet (q), pentet (p), heptet (hept), multiplet (m). Some multiplicities are preceded with "obs." meaning that we observed this multiplicity even if theoretically it should be different. Coupling constants (*J*) are reported in hertz. All data analysis was performed using the MestReNova software package.

EA – Elemental Analysis (C, H, N, S, Br, Cl, F) was performed on a Perkin-Elmer 240 Elemental Analyzer

HRMS – High resolution mass spectra were recorded on a Waters SYNAPT G2-S HDMS using electrospray ionization (ESI) or atmospheric-pressure chemical ionization (APCI) with a time-of-flight (TOF) detector.

GC – Gas chromatography analysis coupled with a flame ionisation detector (GC-FID) were performed on a Shimadzu GCMS-QP2010 SE with helium as the carrier gas and a Zebron ZB 5MSi column.

All GCs were recorded with the following parameters:

Pressure: 90.8 kPA; Total flow: 5.3 mL/min; Column flow: 1.11 mL/min; Linear velocity: 27.5 cm/s; Purge flow: 2.0 mL/min; Split ratio: 2.0

With the following method:

Rate (°C/min)	Temperature (°C)	Hold Time (minutes)
-	100	1.00
40	180	1.50
40	260	1.50
45	300	1.00
50	325	2.00

Photochemical Setup:

All photochemical reactions were performed using a homemade photoreactor composed of a cooling block (connected to a Huber MiniChiller 300 set at 15 °C) and a plate containing 6 LEDs of the desired wavelength. The photochemical vials were placed directly atop of the LEDs thus there is minimal distance between the light source and reaction mixture. Both blue (455 nm) and violet plates (400 nm) are rated at 21 V and 0.7 A, thus the power at each well for the light source is approximately 3W.

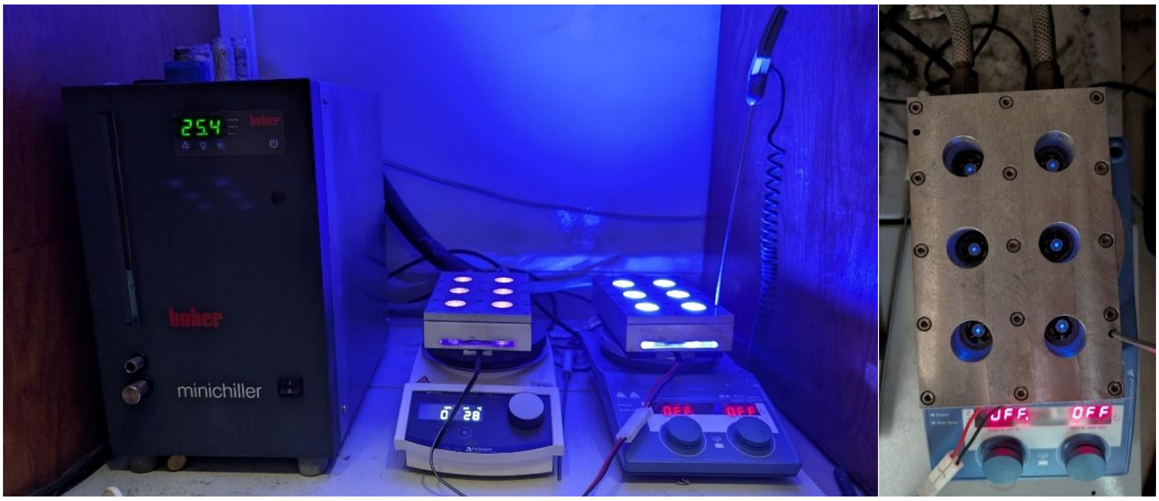

Figure S1: Left: Standard photochemical set ups with violet LEDs (400 nm) and blue LEDs (455 nm) accompanied by the chiller. Right: Aerial view of the wells of the photochemical reactors.

2. Optimisation of the HFIP Insertion with 4-Diazo-2-methylisoquinoline-1,3(2*H*,4*H*)-dione

Model reaction procedure:

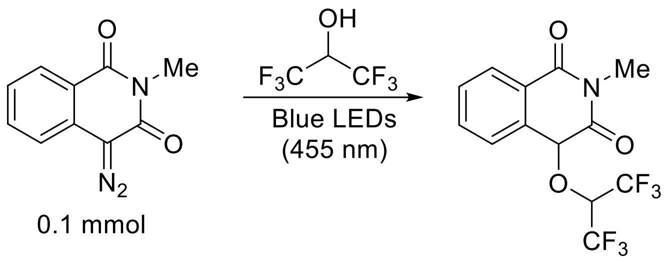

4-Diazoisoquinoline-1,3(2*H*,4*H*)-dione (20 mg, 0.1 mmol) is charged in a vial and dissolved in a HFIP/EtOAc mixture (X:X *c* = X M) then the vial is capped. The vial is irradiated with blue LEDs for X hours at approximately 15 °C then the cap is removed. The solvent is removed in vacuo and dried under a vacuum pump. Trichloroethene (0.1 mmol) is added, and the solution is dissolved in $CDCl_3$ and analyzed by NMR.

2.1. Amount of HFIP vs. Reaction Yield

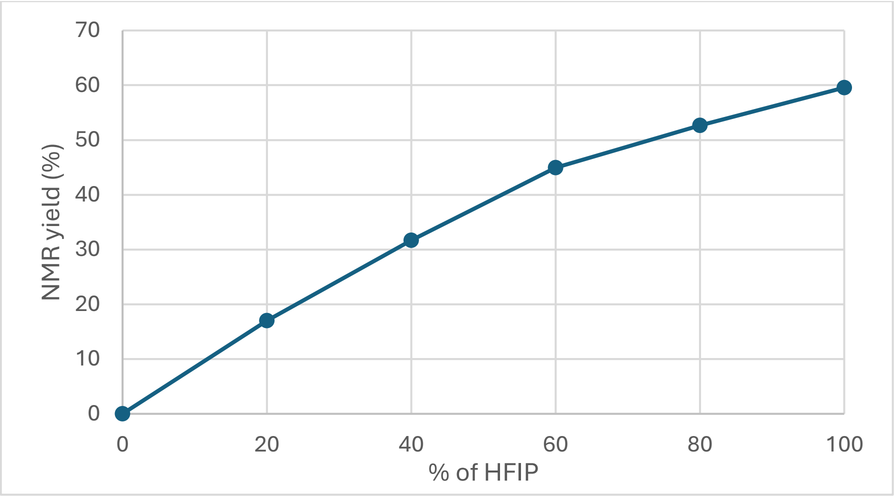

Figure S2: Graph showing the yield versus the % of HFIP in the solvent

Entry	HFIP:EtOAc	Yield (%)[a]
1	2:8	17
2	4:6	32
3	6:4	45
4	8:2	53
**5**	**1:0**	**60**

Reactions conditions: 4-diazo-2-methylisoquinoline-1,3(2*H*,4*H*)-dione (0.1 mmol), solvent (*c* = 0.1 M), blue LEDs, 18 hours. [a]Yield determined by ^1H NMR with trichloroethene as the internal standard.

2.2. Effect of Concentration on the Reaction Yield

Entry	HFIP Conc. (M)	Yield (%)[a]
1	0.050	57
2	0.066	61
**3**	**0.100**	**60**
4	0.133	56
5	0.200	53
6	0.400	46

Reaction conditions: 4-diazo-2-methylisoquinoline-1,3(2*H*,4*H*)-dione (0.1 mmol), HFIP (*c* = X M), blue LEDs, 18 hours. [a]Yield determined by ^{1}H NMR with trichloroethene as the internal standard.

2.3. Reaction Profile over Time

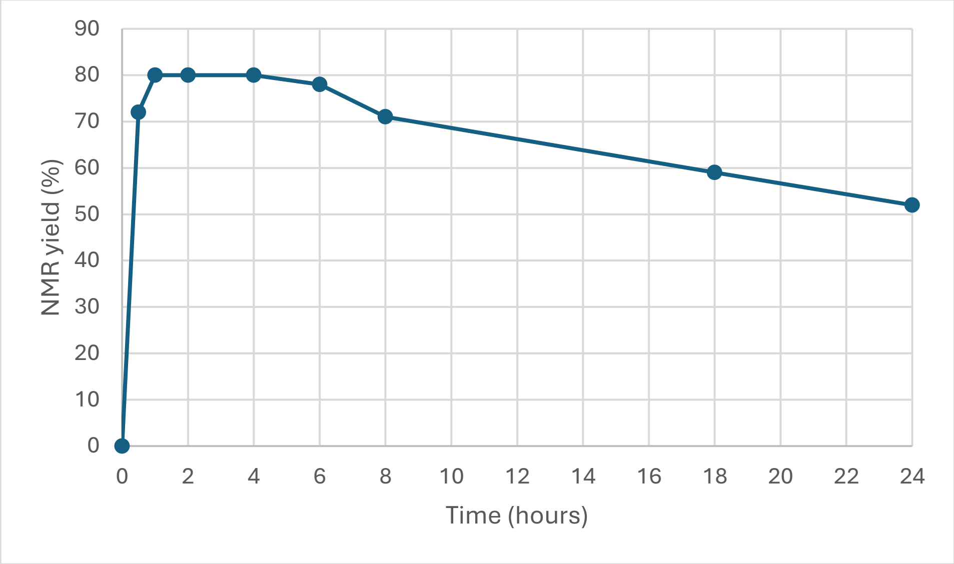

Figure S3: Kinetics study of the reaction between **1a** and HFIP

Entry	Time (hours)	Yield (%)[a]
1	0.5	72
**2**	**1**	**80**
3	2	80
4	4	80
5	6	78
6	8	71
7	18	59
8	24	52

Reaction conditions: 4-diazo-2-methylisoquinoline-1,3(2*H*,4*H*)-dione (0.1 mmol), HFIP (*c* = 0.1 M), blue LEDs, X hours. [a]Yield determined by ^{1}H NMR with trichloroethene as the internal standard.

2.4. Assessing the Reaction with DCM

Entry	HFIP:DCM	Yield (%)[a]
1	2:8	49
2	4:6	50
3	6:4	60

Reactions conditions: 4-diazo-2-methylisoquinoline-1,3(2*H*,4*H*)-dione (0.1 mmol), solvent (*c* = 0.1 M), blue LEDs, 1.5 hours. [a]Yield determined by ^{1}H NMR with trichloroethene as the internal standard.

Conclusions: All photochemical X-H insertion reactions were performed for 1.5 hours in neat HFIP to ensure that full conversion of the diazo compound is achieved for all substrates. Sustained reaction time gradually decomposes the product under the reaction conditions.

3. UV-Vis Spectra of Diazo Compounds

3.1. Influence of *N*-substituents

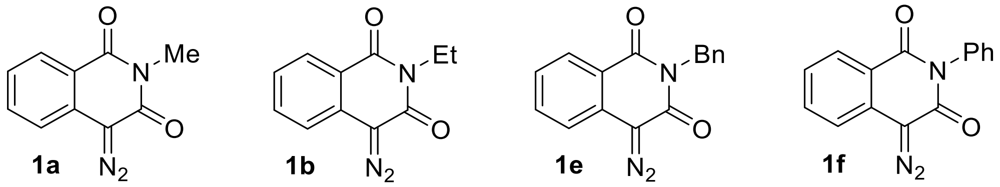

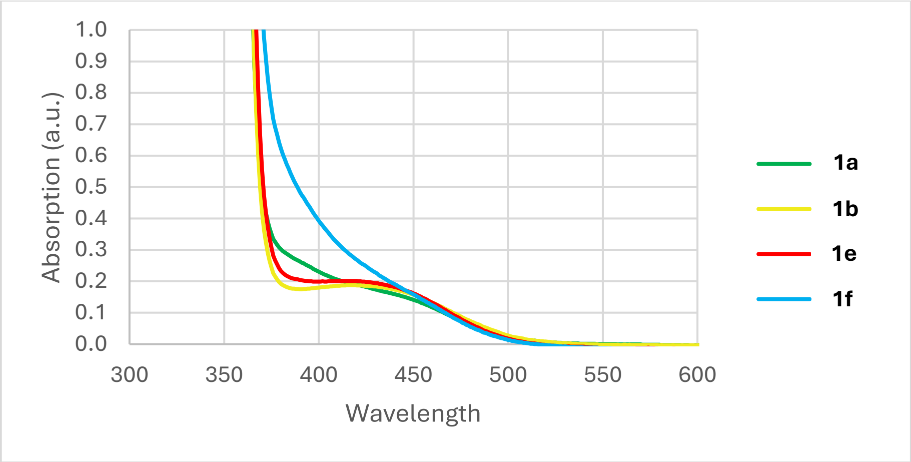

Figure S4: Influence of *N*-substituents on UV-Vis spectra. c = 0.006 M (DCM).

3.2. Influence of the Position of Cl-Substituents on the Aryl Ring

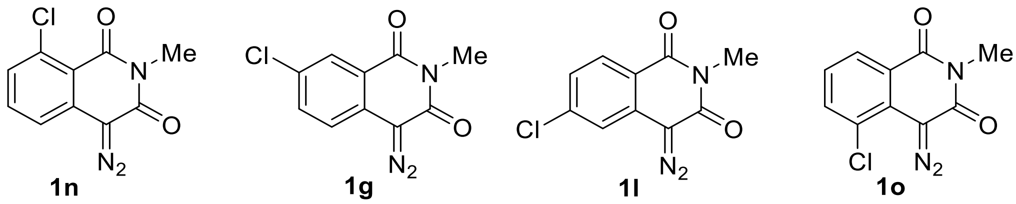

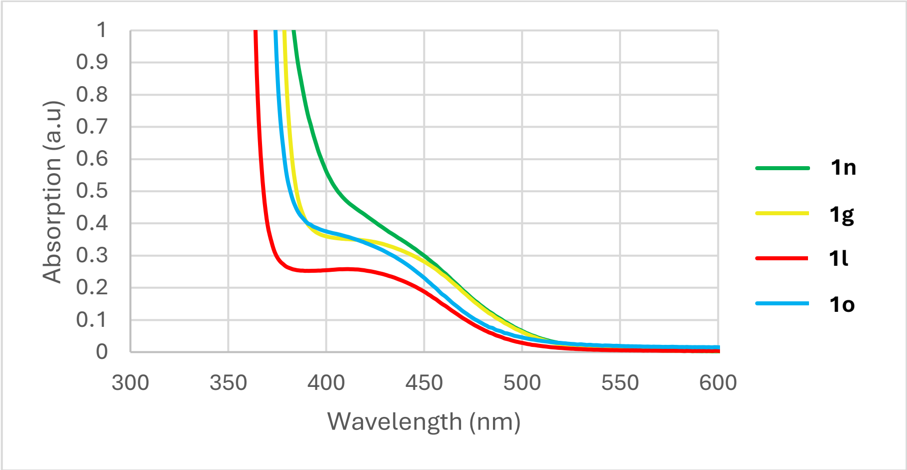

Figure S5: Influence of the position of Cl-substituents on the UV-Vis spectra.
c = 0.006 M (DCM)

3.3. Influence of Substituents at the 7-Position

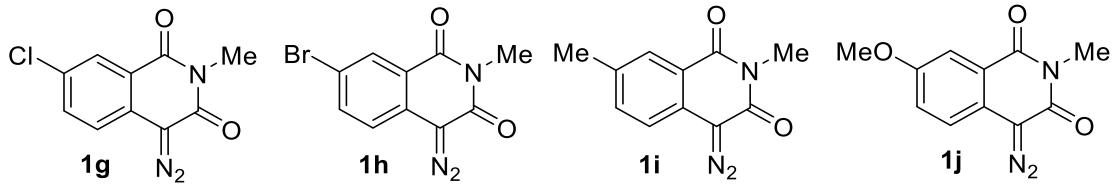

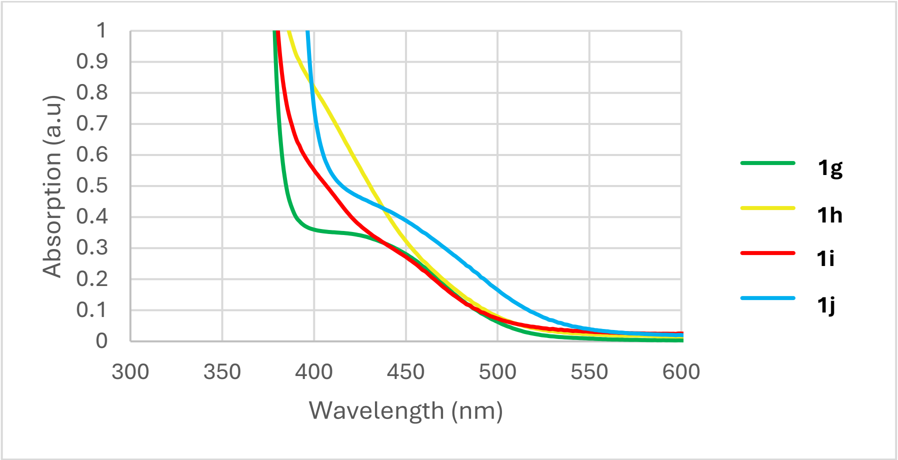

Figure S6: Influence of different functional groups at the 7-position on the UV-Vis spectra. c = 0.006 M (DCM)

3.4. UV-Vis of Sulfoxide-Containing Diazo Compounds

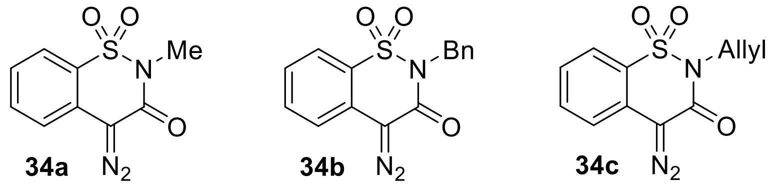

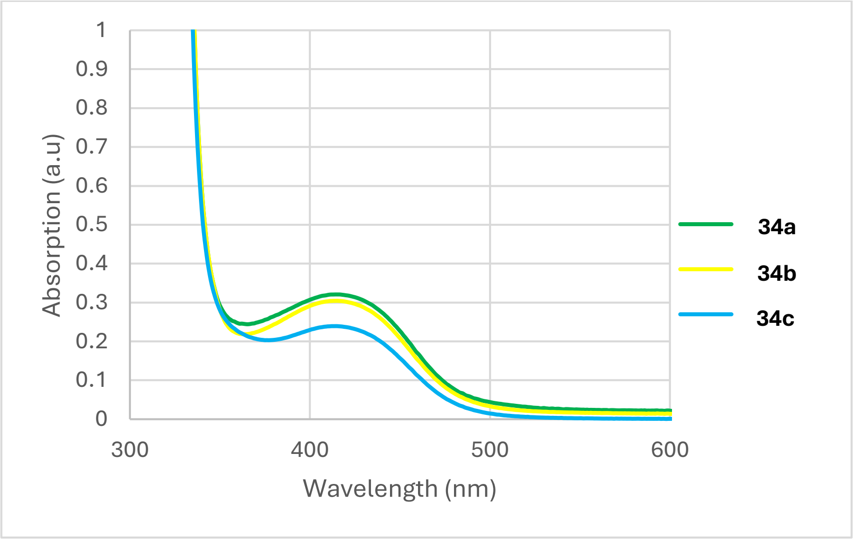

Figure S7: UV-Vis spectra of 4-diazo-2H-benzo[e][1,2]thiazin-3(4H)-one 1,1-dioxides. c = 0.006 M (DCM)

3.5. Comparing the Two Families of Diazo Compounds

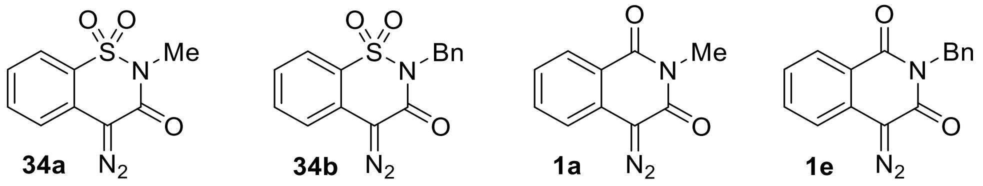

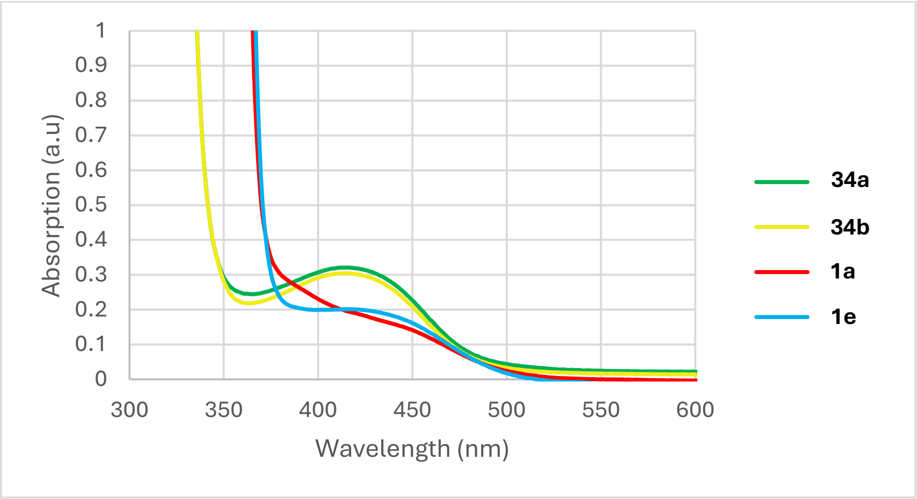

Figure S8: Comparing the UV-Vis spectra of the two families of diazo compound.
c = 0.006 M (DCM)

4. Mechanistic Considerations

To confirm that the reaction forms carbenes, we performed a control experiment with a 1:1 mixture (by volume) of trifluoroethanol (TFE) and styrene. 4-Diazo-2-methylisoquinoline-1,3(2*H*,4*H*)-dione (40 mg, 0.20 mmol) was dissolved in a 1:1 mixture of TFE and styrene (2 ml total) and irradiated for 1.5 hours. The volatiles were removed in vacuo and dried on a vacuum pump. Trichloroethene was added as internal standard, and the crude mixture was subjected to ^{1}H NMR analysis to afford approximately 21% O-H insertion adduct and 24% cyclopropanation adduct. Therefore, the reaction indeed forms a carbene intermediate.

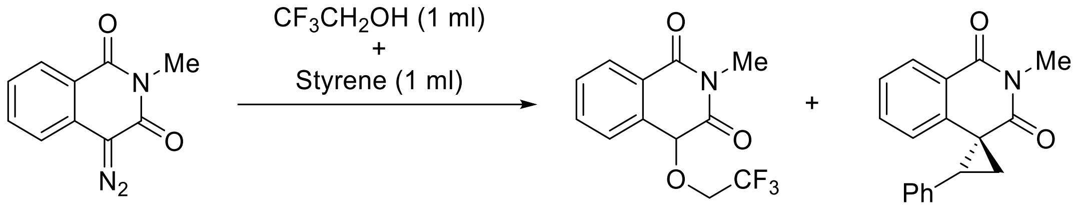

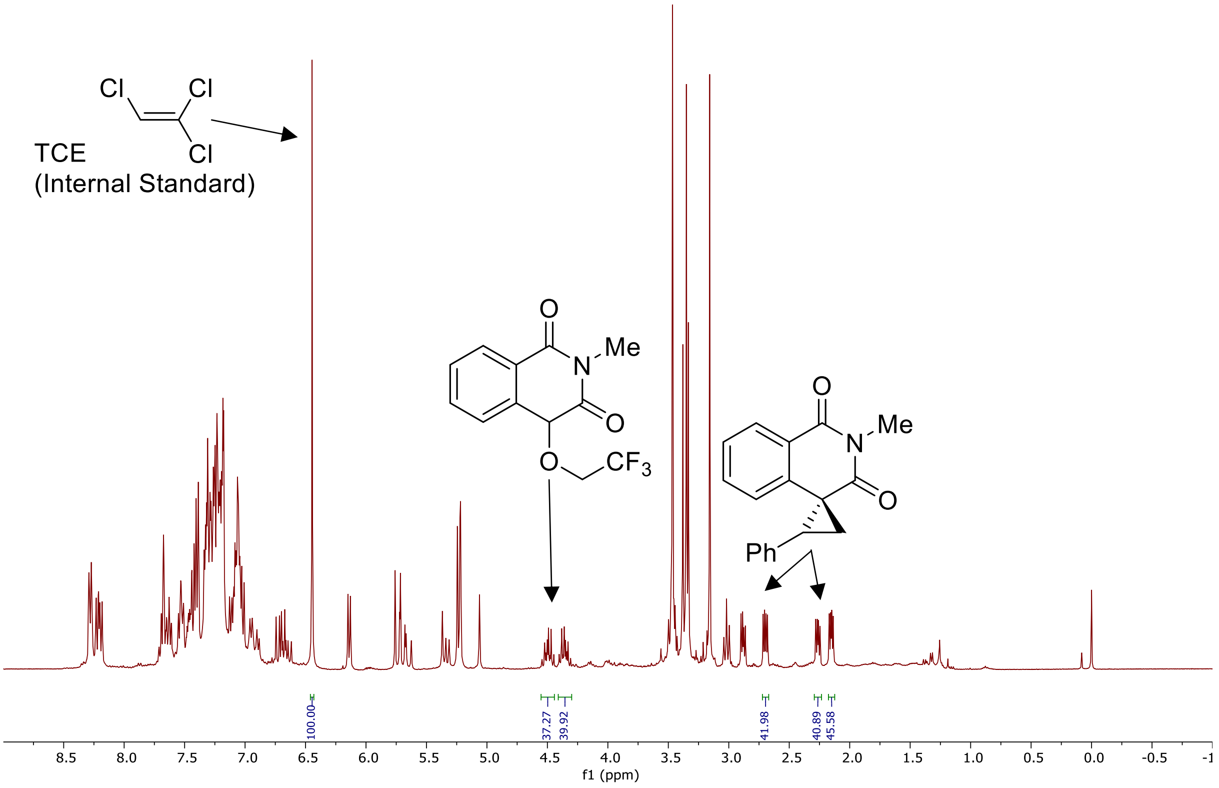

5. General Procedures

5.1. General Procedure A - Synthesis of Substituted (2-(Carboxymethyl)benzoic acids)

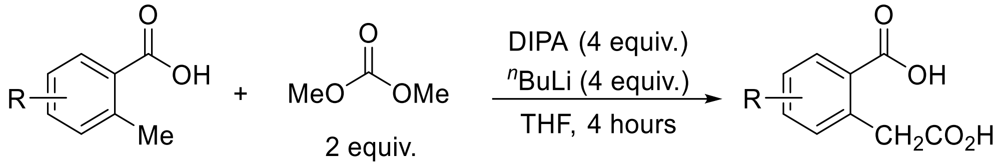

Slightly modified procedure to that reported by Tsou et al. [1]

Diisopropylamine (DIPA, 4.0 equiv.) is added to a flame-dried flask followed by the addition of dry THF (*c* = 3 M with respect to DIPA) then the flask is cooled to -78 °C. ^{n}BuLi (2.5 M in hexanes, 4.0 equiv.) is added dropwise to the solution, warmed to 0 °C and maintained at 0 °C for 5 minutes, then cooled to -78 °C. A solution of dimethyl carbonate (2.0 equiv.) and substituted 2-methylbenzoic acid (1.0 equiv.) in the minimum amount of dry THF, is added dropwise to the freshly prepared LDA solution. After the addition, the reaction is kept at -78 °C for 15 minutes, then warmed to room temperature and stirred for 4 hours. The reaction mixture gradually became turbid over time. After 4 hours, the mixture is diluted with water until the turbidity of the solution significantly reduced, stirred for 30 minutes and the layers are separated. The bottom aq. layer is acidified to pH ~2 with 2 M HCl and extracted with EtOAc thrice. The organic layers are washed with water and brine, dried (Na_2SO_4), filtered and concentrated. The crude residue is triturated in boiling chloroform (60 °C) to afford the diacid.

5.2. General Procedure B - Synthesis of Substituted *N*-Methylisoquinoline-1,3(2*H*,4*H*)-diones

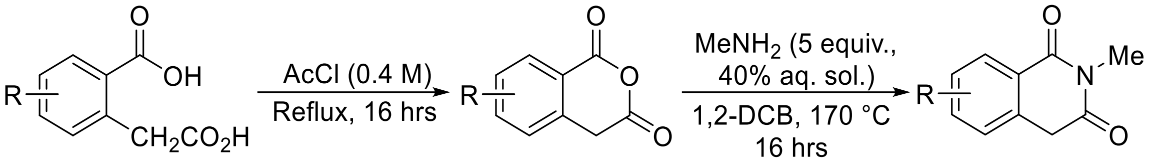

Substituted 2-(carboxymethyl)benzoic acid (1.0 equiv.) is charged in a flask and dissolved in acetyl chloride (*c* = 0.4 M with respect to the benzoic acid). The reaction mixture is refluxed for 16 hours, cooled to room temperature and the remaining acetyl chloride is removed in vacuo and the crude residue is dried on a vacuum pump. Once dry, methylamine (40% aq. solution, 5.0 equiv.) is added to the crude solid and stirred for 5 minutes, then 1,2-dichlorobenzene (1,2-DCB, *c* = 0.35 M with respect to the intermediate isochromane-1,3-dione) is added, a water-cooled reflux condenser is attached to the flask and the reaction is heated to 170 °C for 16 hours. The flask is cooled to room temperature and the crude reaction mixture is pipetted on top of a prepacked silica column and subjected to column chromatography (SiO_2, 20% EtOAc in hexane) to afford the desired isoquinoline-1,3(2*H*,4*H*)-dione.

5.3. General Procedure C - Synthesis of 2*H*-Benzo[e][1,2]thiazin-3(4*H*)-one 1,1-dioxides

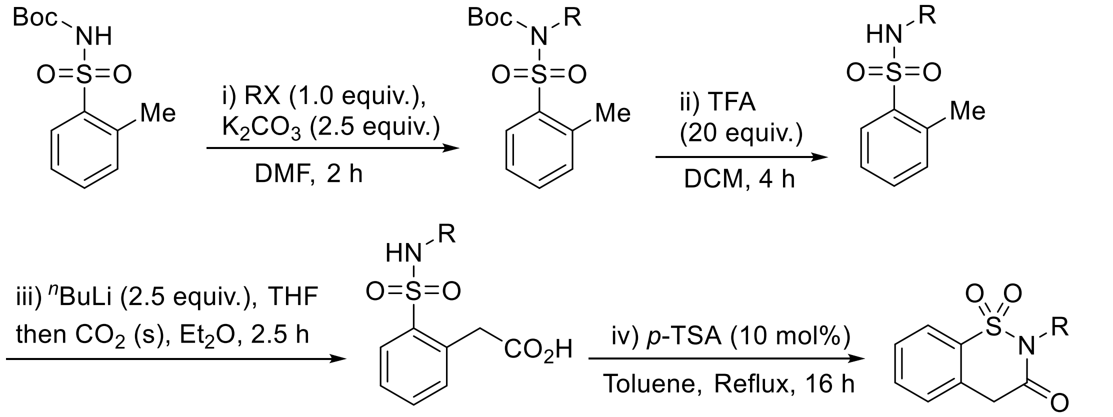

tert-Butyl (*o*-tolylsulfonyl)carbamate was synthesised according to the procedure described by Liu et al.[2] Steps i) and ii) are slightly modified procedures of Barbazanges et al.[3] Steps iii) and iv) are modified procedures of Lombardino and Wiseman.[4]

i) *tert*-Butyl (*o*-tolylsulfonyl)carbamate (1.0 equiv.) is charged in a flask followed by the addition of K_2CO_3 (2.5 equiv.) and DMF (*c* = 0.8 M with respect to the carbamate), then the reaction is vigorously stirred for 1 hour. Alkyl bromide/iodide (1.0 equiv.) is added dropwise and stirred for an additional hour. The reaction mixture is diluted with water and transferred to a separating funnel and extracted with diethyl ether thrice. The organic layer is washed with water and brine, dried (Na_2SO_4), filtered and concentrated and used immediately in the next step.

ii) The trisubstituted amine is charged in a flask and dissolved in DCM (*c* = 0.16 M with respect to the amine) followed by the dropwise addition of trifluoroacetic acid (TFA) (20.0 equiv.). After 4 hours, sat. aq. $NaHCO_3$ is added slowly to the reaction flask to quench the excess TFA. The contents of the flask is transferred to a separating funnel, then extracted with DCM thrice. The organic layer is washed with sat. aq. $NaHCO_3$ thrice and brine, dried (Na_2SO_4), filtered, concentrated and extensively dried on a vacuum pump.

iii) The solid thus obtained is transferred to a flame-dried flask, dissolved in dry THF (*c* = 0.2 M with respect to the sulfonamide) and cooled to 0 °C. ^{n}BuLi (2.5 M in hexanes, 2.5 equiv.) is added dropwise to the solution, which turns yellow and after further addition, red, then the ice bath is removed, and the reaction is allowed to stir for a further 30 minutes at room temperature. The contents of the flask are transferred via a syringe to a second flask containing dry ice and diethyl ether (*c* = 0.1 M with respect to the sulfonamide) with a stir bar and is left to stir for 2 hours. After this time, the reaction is quenched with 2 M HCl (same volume as THF in the reaction), and the solution is transferred to a separating funnel, where the aq. layer is extracted with chloroform thrice. The organic layer is washed with brine, dried (Na_2SO_4), filtered, concentrated and used immediately in the next step.

iv) Toluene (*c* = 0.0625 M with respect to the carboxylic acid) is added to the carboxylic acid followed by the addition of *p*-toluenesulfonic acid monohydrate (10 mol%) and the reaction is heated at reflux for 16 hours. Toluene is removed in vacuo and the crude residue is subjected to column chromatography (SiO_2, 10 -> 20% EtOAc in hexane) to afford the 2*H*-benzo[*e*][1,2]thiazin-3(4*H*)-one 1,1-dioxide product.

5.4. General Procedure D - Synthesis of Diazo Compounds

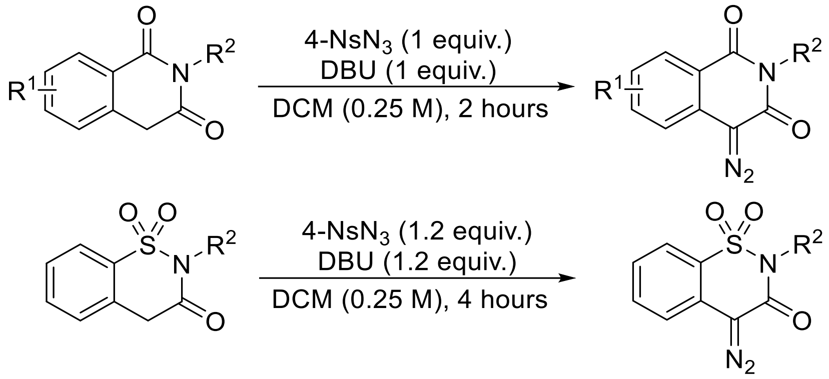

Diazo compounds were synthesised via an analogous procedure as reported by Kantin et al.[5]

A) Isoquinoline-1,3(2*H*,4*H*)-dione and 4-nitrobenzenesulfonyl azide[6] (4-NsN_3, 1.0 equiv.) are charged in a flask and dissolved in DCM (*c* = 0.25 M with respect to the isoquinoline-1,3(2*H*,4*H*)-dione). 1,8-Diazabicyclo(5.4.0)undec-7-ene (DBU) (1.0 equiv.) is then added dropwise to the flask and an immediate colour change is observed. After two hours, the solution is pipetted on top of a pre-packed column and subjected to column chromatography (SiO_2, 20% EtOAc in hexane) to afford the respective diazo compound.

B) An analogous general procedure is used for the synthesis of 4-diazo-2*H*-benzo[*e*][1,2]thiazin-3(4*H*)-one 1,1-dioxides, with 1.2 equiv. of 4-NsN_3 and 1.2 equiv. of DBU and the reaction time is extended to 4 hours.

5.5. General Procedure E - O-H and S-H Insertion Reactions of 4-Diazoisoquinoline-1,3(2*H*,4*H*)-diones

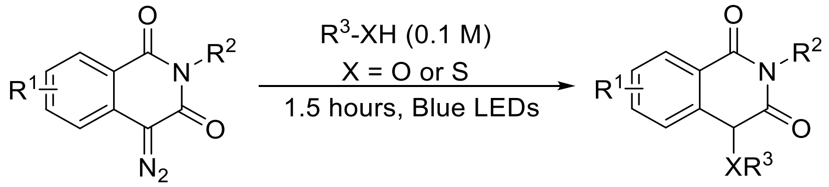

4-Diazoisoquinoline-1,3(2*H*,4*H*)-dione (0.20 mmol) is charged in a vial and dissolved in the respective alcohol or thiol (2 ml) then the vial is capped. The vial is irradiated with blue LEDs for typically 1.5 hours at approximately 15 °C then the cap is removed.

For volatile alcohols/thiols: The solution is transferred to a flask with DCM and all the volatiles are removed in vacuo. The crude residue is purified by column chromatography (SiO_2, 10% EtOAc in hexane) to afford the X-H insertion product.

For non-volatile alcohols/thiols: The solution is directly pipetted on top of a prepacked column and subjected to column chromatography (SiO_2, 10% EtOAc in hexane) to afford the X-H insertion product.

5.6. General Procedure F – Arene Insertion Reactions of 4-Diazo-2-methylisoquinoline-1,3(2*H*,4*H*)-diones

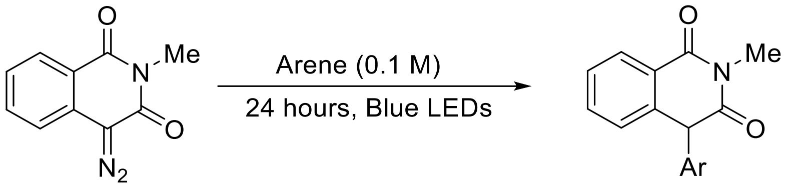

4-Diazo-2-methylisoquinoline-1,3(2*H*,4*H*)-dione (0.20 mmol) is charged in a vial and dissolved in the respective arene (2 ml) then the vial is capped. The vial is irradiated with blue LEDs for 24 hours at approx. 15 °C then the cap is removed, and the crude residue is pipetted on top of a prepacked silica column (SiO_2, 10% - 20% EtOAc in hexane) to afford the C-H insertion product.

5.7. General Procedure G - O-H and S-H Insertion Reactions of 4-Diazo-2*H*-benzo[*e*][1,2]thiazin-3(4*H*)-one 1,1-dioxides

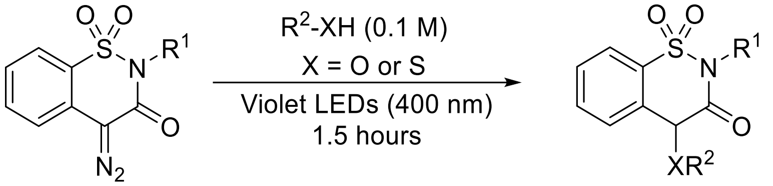

General Procedure: 4-Diazo-2*H*-benzo[*e*][1,2]thiazin-3(4*H*)-one 1,1-dioxide (0.15 mmol) is charged in a vial and dissolved in the respective alcohol or thiol (1.5 ml) then the vial is capped. The vial is irradiated with violet LEDs for 1.5 hours at approx. 15 °C then the reaction mixture is transferred to a flask with DCM and all the volatiles are removed in vacuo. The crude residue is purified by column chromatography (SiO_2, 10% EtOAc in hexane) to afford the X-H insertion product.

6. (2-(Carboxymethyl))benzoic acids

4-methoxy-2-methylbenzoic acid (S1)

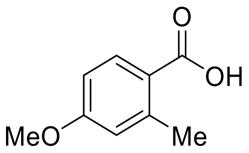

The title compound was synthesised from 4-hydroxy-2-methylbenzoic acid (2.28 g, 15.0 mmol) via an analogous procedure by Lobb et al. for the methylation of 2-chloro-4-methoxybenzoic acid[7] in 94% yield (2.34 g) as a brown solid.

^{1}H NMR (400 MHz, DMSO-d_6) δ 12.40 (s, 1H), 7.84 (d, *J* = 8.2 Hz, 1H), 6.84 – 6.81 (m, 2H), 3.79 (s, 3H), 2.52 (s, 3H).

^{13}C{^{1}H} NMR (101 MHz, DMSO-d_6) δ 167.9, 161.7, 142.1, 132.7, 122.1, 116.7, 111.1, 55.2, 21.8.

The spectroscopic data is consistent with that previously reported in the literature.[8]

2-(carboxymethyl)-4-methoxybenzoic acid (S2)

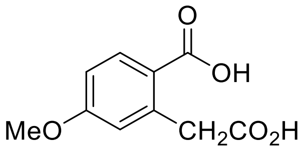

The title compound was synthesised according to General Procedure A from 4-methoxy-2-methylbenzoic acid (2.50 g, 15.0 mmol) in 53% yield (1.66 g) as a brown solid.

^{1}H NMR (400 MHz, DMSO-d_6) δ 12.27 (s, 2H), 7.89 (d, *J* = 8.4 Hz, 1H), 6.93 – 6.89 (m, 2H), 3.92 (s, 2H), 3.81 (s, 3H).

^{13}C{^{1}H} NMR (101 MHz, DMSO-d_6) δ 172.3, 167.6, 161.7, 139.2, 132.7, 122.4, 117.9, 111.9, 55.3. (One peak overlaps with the residual DMSO peaks.)

The spectroscopic data is consistent with that previously reported in the literature.[9]

2-(carboxymethyl)-5-chlorobenzoic acid (S3)

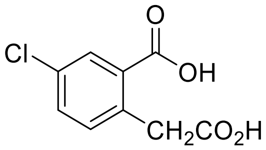

The title compound was synthesised according to General Procedure A from 5-chloro-2-methylbenzoic acid (2.13 g, 12.5 mmol) in 84% yield (2.26 g) as a white solid.

^{1}H NMR (500 MHz, DMSO-*d*$_6$) δ 12.74 (s, 2H), 7.85 (d, *J* = 2.4 Hz, 1H), 7.60 – 7.57 (m, 1H), 7.38 (dd, *J* = 8.3, 3.1 Hz, 1H), 3.93 (s, 2H).

^{13}C{^{1}H} NMR (126 MHz, DMSO-*d*$_6$) δ 172.1, 167.0, 135.5, 134.3, 132.5, 131.5, 129.8. (CH_2 peak is overlapping with one of the DMSO residual solvent peaks. One of the quaternary aromatic peaks were not observed).

m.p. 195 – 198 °C (lit. 195 – 198 °C)[10]

The spectroscopic data is consistent with that previously reported in the literature.[11]

2-(carboxymethyl)-3-chlorobenzoic acid (S4)

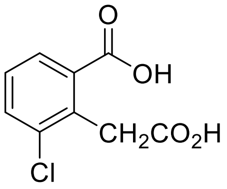

The title compound was synthesised according to General Procedure A from 3-chloro-2-methylbenzoic acid (2.13 g, 12.5 mmol) in 83% yield (2.24 g) as a yellow solid.

^{1}H NMR (500 MHz, DMSO-*d*$_6$) δ 12.82 (s, 2H), 7.84 (dd, *J* = 7.8, 1.4 Hz, 1H), 7.69 – 7.67 (m, 1H), 7.40 (td, *J* = 8.0, 4.6 Hz, 1H), 4.13 (s, 2H).

^{13}C{^{1}H} NMR (126 MHz, DMSO-*d*$_6$) δ 171.1, 167.7, 135.6, 133.8, 133.4, 132.5, 129.2, 128.3, 35.8.

HRMS (ESI$^-$) m/z calc'd for $C_9H_6O_4Cl^-$: 212.9955; found: 212.9957 [M-H$^+$]

m.p. 171 – 172 °C (lit. 174 – 176 °C)[10]

GC (FID):

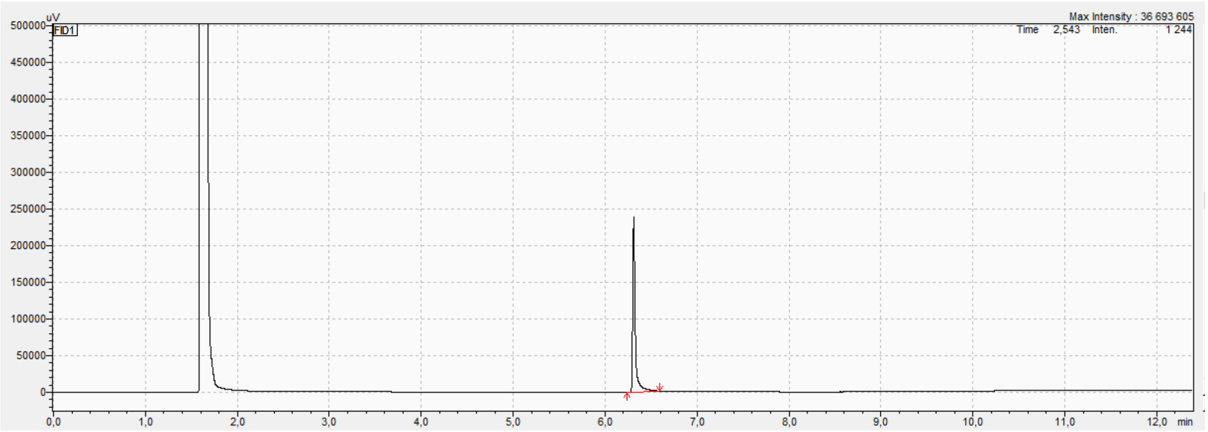

4-bromo-2-(carboxymethyl)benzoic acid (S5)

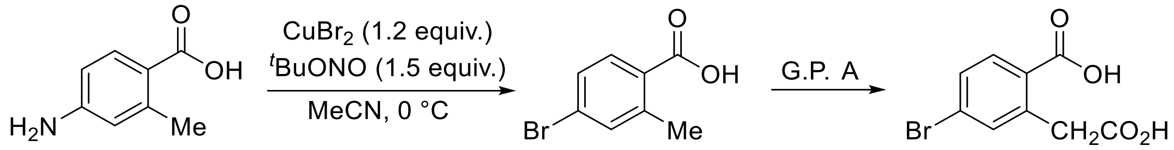

4-Bromo-2-methylbenzoic acid was synthesised according to an analogous procedure by Glennon et al. for the bromination of 4-amino-2-hydroxybenzoic acid.[12]

Copper (II) bromide (5.36 g, 24.0 mmol) is charged in a flask followed by the addition of MeCN (60 mL) and cooled to 0 °C. *tert*-Butyl nitrite (3.6 mL, 30 mmol) is added in one portion to the reaction mixture and 4-amino-2-methylbenzoic acid (3.02 g, 20.0 mmol) is added portionwise and the reaction is stirred for a further two hours at 0 °C. After this time, the solution is basified to pH ~10 with 3 M NaOH and washed with Et_2O twice. The aq. layer is acidified to pH ~2 and extracted with EtOAc thrice, the organic layer is washed with brine twice, dried (Na_2SO_4), filtered and concentrated. 4-bromo-2-methylbenzoic acid (2.32 g) was afforded in approximately 90% purity and used immediately in General Procedure A, without any further purification, to afford the title compound in 35% yield (2 steps, 1.79 g) as a brown solid.

^{1}H NMR (400 MHz, DMSO-d_6) δ 12.66 (s, 2H), 7.82 (d, J = 8.3 Hz, 1H), 7.62 – 7.57 (m, 2H), 3.94 (s, 2H).

^{13}C{^{1}H} NMR (101 MHz, DMSO-d_6) δ 172.0, 167.5, 139.2, 135.0, 132.4, 130.0, 129.9, 125.4, 39.4.

Despite drying on a vacuum pump at 65 °C for 72 hours, traces of EtOAc and $CHCl_3$ remained in the product and are identified in the spectra. Nevertheless, the peaks corresponding to the product are consistent with those reported by Trost and Kalnmals.[13]

7. Isoquinoline-1,3(2*H*,4*H*)-diones

2-Methylisoquinoline-1,3(2*H*,4*H*)-dione was synthesised according to the procedure reported by Jangir et al.[14] 2-Ethylisoquinoline-1,3(2*H*,4*H*)-dione, 2-propylisoquinoline-1,3(2*H*,4*H*)-dione, 2-isopropylisoquinoline-1,3(2*H*,4*H*)-dione, 2-benzylisoquinoline-1,3(2*H*,4*H*)-dione, and 2-phenylisoquinoline-1,3(2*H*,4*H*)-dione were all synthesised according to the procedures reported by Kantin et al.[5] When substituents are present on the aromatic rings, we found these methods were unsuccessful or gave low yields, thus we developed General Procedure B instead.

7-chloro-2-methylisoquinoline-1,3(2*H*,4*H*)-dione (S6)

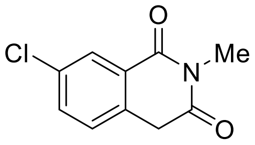

The title compound was synthesised according to General Procedure B from 5-chloro-2-(carboxymethyl)benzoic acid 2.15 g (10.0 mmol) in 83% yield (1.74 g) as a beige solid. Eluent for column chromatography (SiO_2): 20% EtOAc in hexane.

^{1}H NMR (500 MHz, $CDCl_3$) δ 8.17 (d, *J* = 2.3 Hz, 1H), 7.53 (dd, *J* = 8.2, 2.3 Hz, 1H), 7.22 (d, *J* = 8.5 Hz, 1H), 4.00 (s, 2H), 3.36 (s, 3H).

^{13}C{^{1}H} NMR (126 MHz, $CDCl_3$) δ 169.7, 164.1, 134.1, 133.8, 132.3, 129.0, 128.8, 126.9, 36.0, 27.0.

HRMS (ESI$^-$) m/z calc'd for $C_{10}H_7NO_2Cl^-$: 208.0165; found: 208.0166 [M-H$^+$]

m.p. 116 – 118 °C (lit. 116 – 118 °C)[15]

GC (FID):

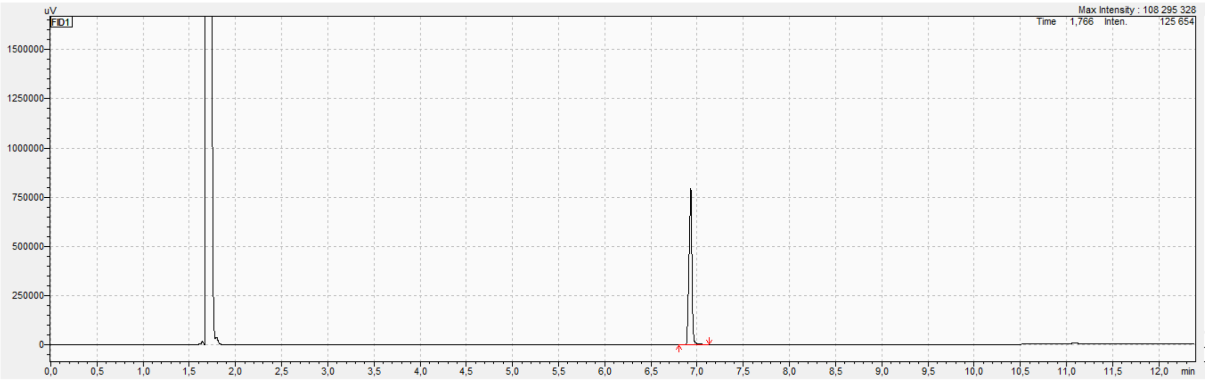

7-bromo-2-methylisoquinoline-1,3(2*H*,4*H*)-dione (S7)

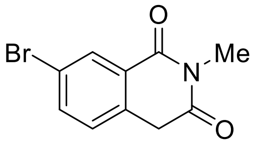

The title compound was synthesised according to General Procedure B from 5-bromo-2-(carboxymethyl)benzoic acid[16] (1.91 g, 7.37 mmol) in 70% yield (1.31 g) as a white solid. Eluent for column chromatography (SiO_2): 20% EtOAc in hexane.

^{1}H NMR (500 MHz, $CDCl_3$) δ 8.30 (d, *J* = 2.1 Hz, 1H), 7.67 (dd, *J* = 8.2, 2.1 Hz, 1H), 7.15 (d, *J* = 8.2 Hz, 1H), 3.97 (s, 2H), 3.34 (s, 3H).

^{13}C{^{1}H} NMR (126 MHz, $CDCl_3$) δ 169.6, 164.0, 136.7, 132.8, 131.9, 128.9, 127.1, 121.7, 36.0, 27.0.

HRMS (ESI$^-$) m/z calc'd for $C_{10}H_7NO_2Br^-$: 251.9660; found: 251.9663 $[M-H^+]^-$

m.p. 132 – 134 °C

GC (FID):

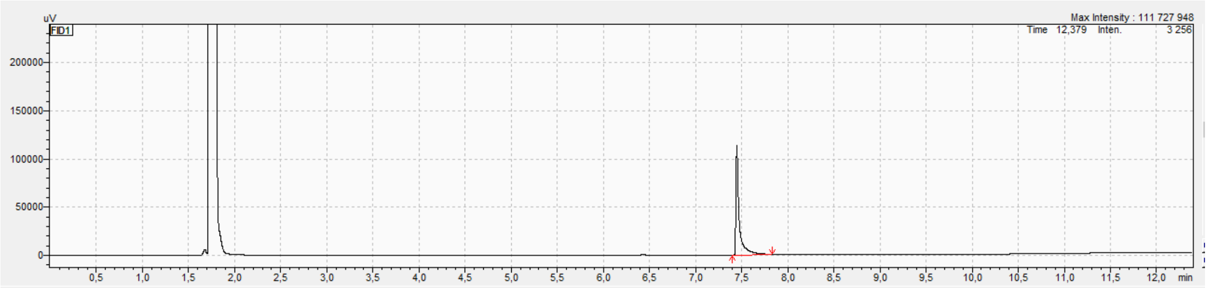

2,7-dimethylisoquinoline-1,3(2*H*,4*H*)-dione (S8)

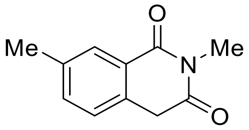

The title compound was synthesised according to General Procedure B from 2-(carboxymethyl)-5-methylbenzoic acid[17] (457 mg, 2.35 mmol) in 61% yield (271 mg) as a yellow solid. Eluent for column chromatography (SiO_2): 20% EtOAc in hexane.

^{1}H NMR (600 MHz, $CDCl_3$) δ 8.01 (s, 1H), 7.40 – 7.38 (m, 1H), 7.16 (d, *J* = 7.8 Hz, 1H), 4.00 (s, 2H), 3.36 (s, 3H), 2.42 (s, 3H).

^{13}C{^{1}H} NMR (151 MHz, $CDCl_3$) δ 170.6, 165.5, 137.8, 134.8, 131.2, 129.3, 127.2, 125.2, 36.2, 26.9, 21.2.

HRMS (ESI$^+$) m/z calc'd for $C_{11}H_{12}NO_2^+$: 190.0868; found: 190.0874 $[M+H]^+$

m.p. 134 – 136 °C

GC (FID):

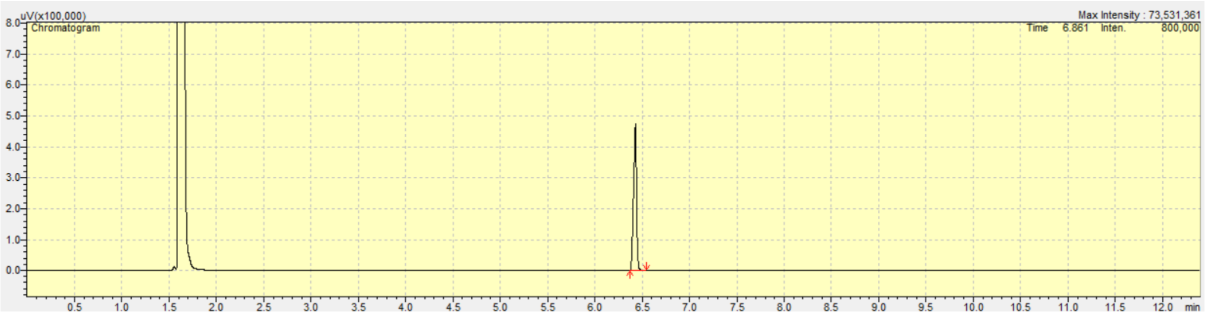

7-methoxy-2-methylisoquinoline-1,3(2*H*,4*H*)-dione (S9)

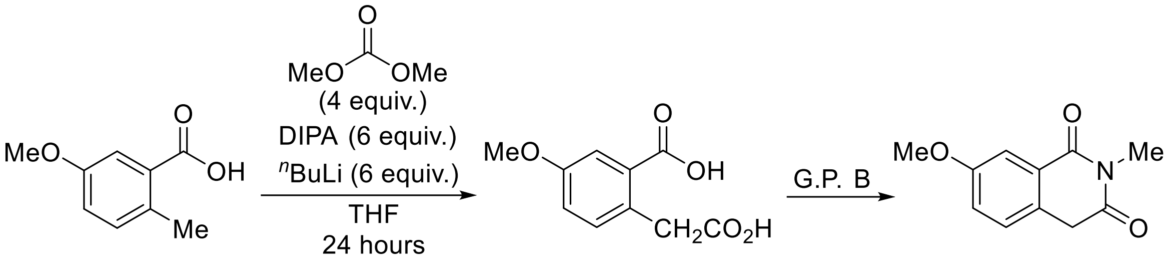

Diisopropylamine (7.31 g, 72.0 mmol, 6.00 equiv.) is added to a flame-dried flask followed by the addition of dry THF (24 mL) then the flask is cooled to -78 °C. ^{n}BuLi (29 mL, 2.5 M in hexanes, 72.0 mmol, 6.00 equiv.) is added dropwise to the solution, warmed to 0 °C and maintained at 0 °C for 5 minutes, then cooled to -78 °C. A solution of dimethyl carbonate (4.34 g, 4.00 equiv.) and 5-methoxy-2-methylbenzoic acid (2.00 g, 12.0 mmol, 1.0 equiv.) in the minimum amount of dry THF, is added dropwise to the freshly prepared LDA solution. After the addition, the reaction is kept at -78 °C for 1 hour, then warmed to room temperature and stirred for 18 hours. The reaction mixture gradually became turbid over time. After 18 hours, the mixture is diluted with water until the turbidity of the solution significantly reduced, stirred for 30 minutes and the layers are separated. The bottom aq. layer is acidified to pH ~2 with 2 M HCl and extracted with EtOAc thrice. The organic layers are washed with water and brine, dried (Na_2SO_4), filtered and concentrated. The crude residue is triturated in boiling chloroform (60 °C) which afforded 962 mg of 2-(carboxymethyl)-5-methoxybenzoic acid in approximately 90% purity. The beige solid was used in General Procedure B, without any further purification, to afford the title compound in 27% yield (2 steps, 674 mg) as a yellow solid. Eluent for column chromatography (SiO_2): gradually from 20% of EtOAc in hexane to 30%.

^{1}H NMR (600 MHz, $CDCl_3$) δ 7.68 (d, *J* = 2.6 Hz, 1H), 7.18 – 7.14 (m, 2H), 3.98 (s, 2H), 3.88 (s, 3H), 3.37 (s, 3H).

^{13}C{^{1}H} NMR (151 MHz, $CDCl_3$) δ 170.6, 165.3, 159.2, 128.5, 126.3, 122.1, 111.4, 55.8, 35.8, 27.0.

The spectroscopic data is consistent with that previously reported in the literature [18]

6-methoxy-2-methylisoquinoline-1,3(2*H*,4*H*)-dione (S10)

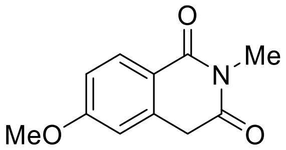

The title compound was synthesised according to General Procedure B from 2-(carboxymethyl)-4-methoxybenzoic acid (1.22 g, 5.82 mmol) in 62% yield (745 mg) as a colourless solid. Eluent for column chromatography (SiO_2): gradually from 20% of EtOAc in hexane to 30%.

^{1}H NMR (600 MHz, $CDCl_3$) δ 8.14 (dd, *J* = 8.9, 1.8 Hz, 1H), 6.95 (dd, *J* = 8.8, 2.4 Hz, 1H), 6.71 – 6.70 (m, 1H), 4.00 (s, 2H), 3.88 (s, 3H), 3.35 (s, 3H).

^{13}C{^{1}H} NMR (151 MHz, $CDCl_3$) δ 170.4, 164.9, 164.0, 136.4, 131.4, 118.3, 114.5, 111.4, 55.8, 36.7, 26.8.

HRMS (ESI$^+$) m/z calc'd for $C_{11}H_{12}NO_3^+$: 206.0817; found: 206.0818 $[M+H]^+$

m.p. 169 – 171 °C

GC (FID):

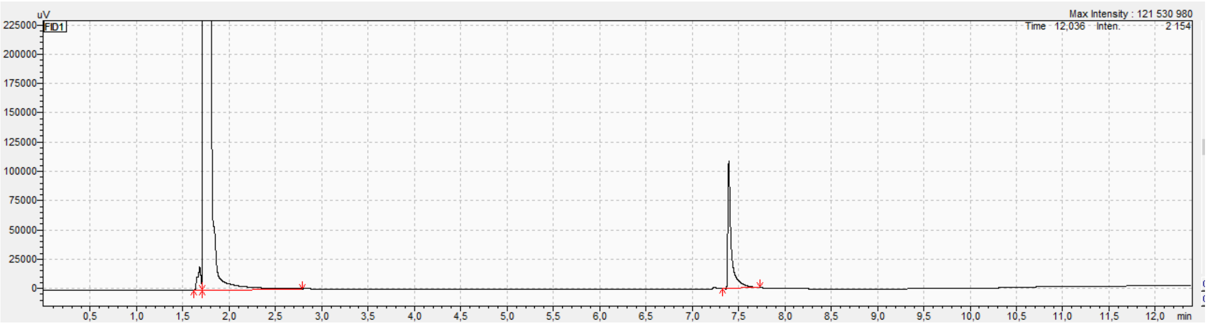

6-chloro-2-methylisoquinoline-1,3(2*H*,4*H*)-dione (S11)

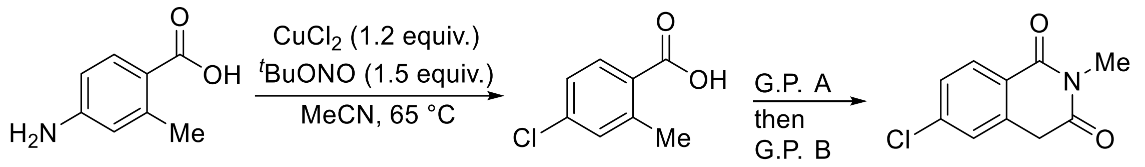

4-chloro-2-methylbenzoic acid was synthesised according to an analogous procedure by Doyle et al. for the chlorination of 2-aminobenzoic acid.[19]

To a flame-dried flask under argon was added copper(II) chloride (3.23 g, 24.0 mmol) and dry MeCN (40 mL), then the flask was cooled to 0 °C. *tert*-Butyl nitrite (3.09 g, 30.0 mmol) was added in one portion followed by the portionwise addition of 4-amino-2-methylbenzoic acid (3.02 g, 20.0 mmol), then the reaction mixture was heated to 65 °C for 1 hour. After this time, the reaction was cooled to room temperature and poured into a 20% conc. HCl solution and extracted with diethyl ether twice. The organic layers were washed with a 20% conc. HCl solution and brine, dried (Na_2SO_4), filtered and concentrated which afforded 2.78 g of 4-chloro-2-methylbenzoic acid in approximately 85% purity. The impure 4-chloro-2-methylbenzoic acid was subjected to General Procedure A and General Procedure B successively to afford the title compound as a beige solid in overall 14% yield (574 mg). Eluent for column chromatography (SiO_2): 20% EtOAc in hexane.

^{1}H NMR (500 MHz, $CDCl_3$) δ 8.15 (d, *J* = 8.5 Hz, 1H), 7.43 – 7.41 (m, 1H), 7.29 (s, 1H), 4.02 (s, 2H), 3.37 (s, 3H).

^{13}C{^{1}H} NMR (126 MHz, $CDCl_3$) δ 169.5, 164.4, 140.3, 135.7, 130.8, 128.5, 127.3, 124.0, 36.2, 27.0.

HRMS (APCI$^+$) m/z calc'd for $C_{10}H_9NO_2Cl^+$: 210.0322; found: 210.0326 $[M+H]^+$

m.p. 110 °C (dec., purple discolouration observed)

GC (FID):

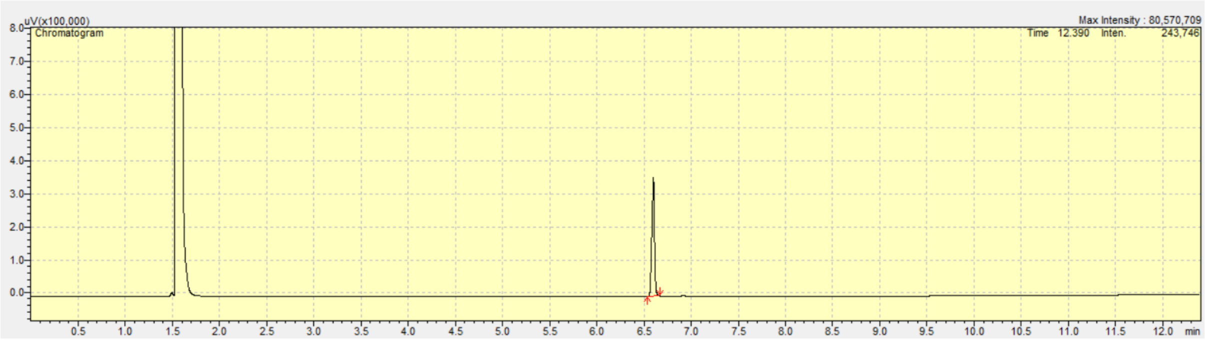

6-bromo-2-methylisoquinoline-1,3(2*H*,4*H*)-dione (S12)

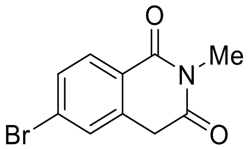

The title compound was synthesised according to General Procedure B from 4-bromo-2-(carboxymethyl)benzoic acid (1.69 g, 6.52 mmol) in 56% yield (929 mg) as a beige solid. Eluent for column chromatography (SiO_2): 20% EtOAc in hexane.

^{1}H NMR (500 MHz, $CDCl_3$) δ 8.07 (d, *J* = 8.4 Hz, 1H), 7.60 – 7.57 (m, 1H), 7.46 (s, 1H), 4.02 (s, 2H), 3.36 (s, 3H).

^{13}C{^{1}H} NMR (126 MHz, $CDCl_3$) δ 169.5, 164.6, 135.8, 131.5, 130.8, 130.3, 128.9, 124.4, 36.1, 27.0.

HRMS (ESI$^-$) m/z calc'd for $C_{10}H_7NO_2Br^-$: 251.9660; found: 251.9661 $[M-H^+]^-$

m.p. 150 °C (purple discolouration observed)

GC (FID):

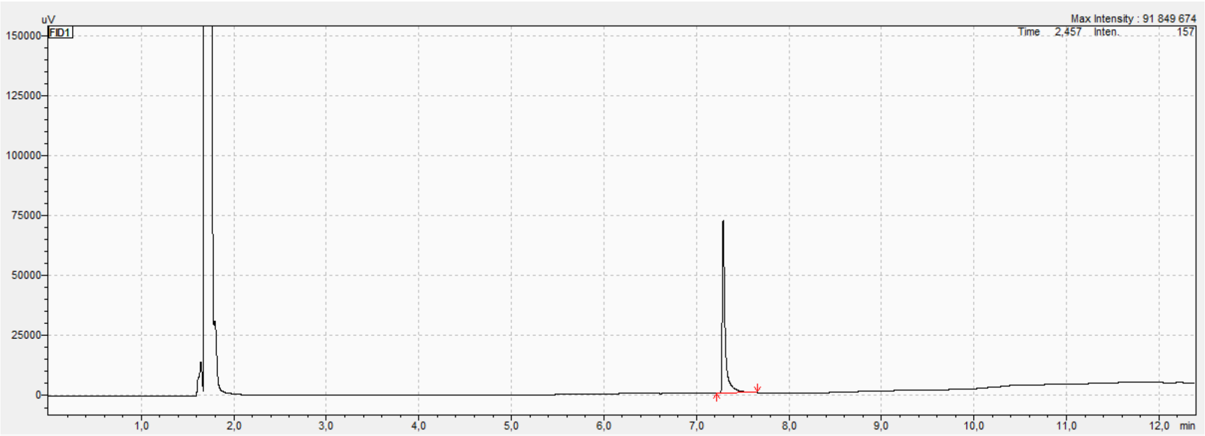

8-chloro-2-methylisoquinoline-1,3(2*H*,4*H*)-dione (S13)

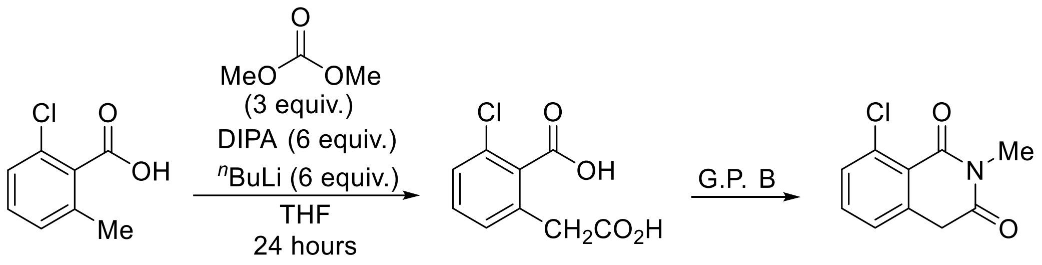

Diisopropylamine (DIPA) (7.59 g, 75.0 mmol, 6.00 equiv.) is added to a flame-dried flask followed by the addition of dry THF (30 mL) then the flask is cooled to -78 °C. ^{n}BuLi (30 mL, 2.5 M in hexanes, 75.0 mmol, 6.00 equiv.) is added dropwise to the solution, warmed to 0 °C and maintained at 0 °C for 5 minutes, then cooled to -78 °C. A solution of dimethyl carbonate (3.38 g, 37.5 mmol, 3.0 equiv.) and 2-chloro-6-methylbenzoic acid (2.13 g, 12.5 mmol) in the minimum amount of dry THF, is added dropwise to the freshly prepared LDA solution. After the addition, the reaction is kept at -78 °C for 1 hour, then warmed to room temperature and stirred for 18 hours. The solution is then diluted with water (75 mL) until the turbidity of the mixture significantly reduced, stirred for 30 minutes and the layers are separated. The bottom aq. layer is acidified to pH ~2 with 2 M HCl and extracted with EtOAc thrice. The organic layers are washed with water and brine, dried (Na_2SO_4), filtered and concentrated and dried further on a vacuum pump. The brown sticky residue was subjected to General Procedure B, without further purification, to afford the title compound in 26% yield (670 mg) as a brown solid. Eluent for column chromatography (SiO_2): 20% EtOAc in hexane.

^{1}H NMR (500 MHz, $CDCl_3$) δ 7.50 – 7.44 (m, 2H), 7.21 – 7.19 (m, 1H), 4.05 (s, 2H), 3.36 (s, 3H).

^{13}C{^{1}H} NMR (126 MHz, $CDCl_3$) δ 169.0, 163.0, 136.9, 136.6, 133.3, 131.8, 126.3, 122.5, 37.1, 27.2.

HRMS (ESI$^-$) m/z calc'd for $C_{10}H_7NO_2Cl^-$: 208.0165; found: 208.0167 $[M-H^+]^-$

m.p. 195 °C (dec.)

GC (FID):

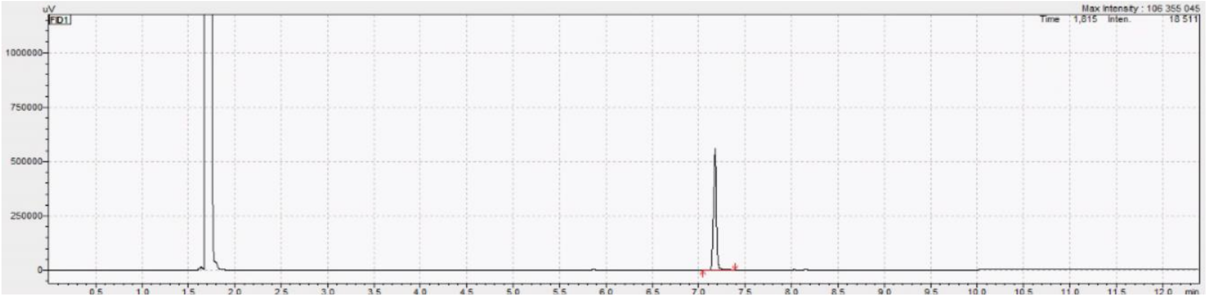

5-chloro-2-methylisoquinoline-1,3(2*H*,4*H*)-dione (S14)

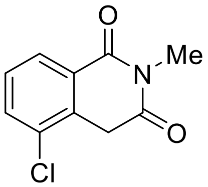

The title compound was synthesised according to General Procedure B from 2-(carboxymethyl)-3-chlorobenzoic acid (2.13 g, 9.91 mmol) in 93% yield (1.94 g) as a white solid. Eluent for column chromatography (SiO_2): 20% EtOAc in hexane.

^{1}H NMR (500 MHz, $CDCl_3$) δ 8.16 (d, *J* = 7.9 Hz, 1H), 7.64 (d, *J* = 7.9 Hz, 1H), 7.42 (t, *J* = 7.9 Hz, 1H), 4.02 (s, 2H), 3.37 (s, 3H).

^{13}C{^{1}H} NMR (126 MHz, $CDCl_3$) δ 169.1, 164.3, 134.1, 132.9, 132.3, 128.8, 127.8, 127.3, 35.0, 27.0.

HRMS (ESI$^-$) m/z calc'd for $C_{10}H_7NO_2Cl^-$: 208.0165; found: 208.0166 $[M-H^+]^-$

m.p. 126 – 128 °C

GC (FID):

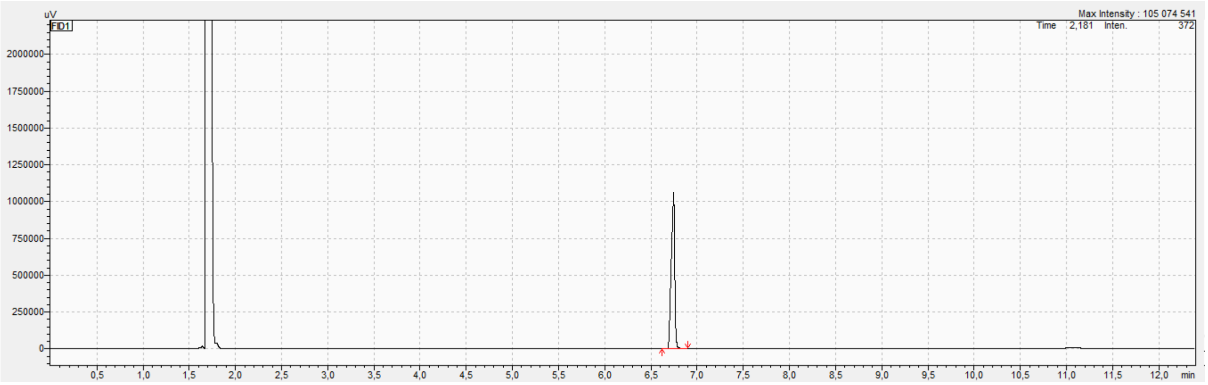

8. 2*H*-Benzo[*e*][1,2]thiazin-3(4*H*)-one 1,1-dioxides

2-methyl-2*H*-benzo[*e*][1,2]thiazin-3(4*H*)-one 1,1-dioxide (S15)

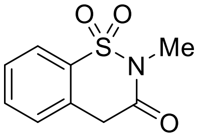

The title compound was synthesised according to General Procedure C, from *tert*-butyl (*o*-toylsulfonyl)carbamate (2.71 g, 10.0 mmol) in 43% yield (899 mg) as a white solid. Eluent for column chromatography (SiO_2): gradually from 10% of EtOAc in hexane to 20%.

^{1}H NMR (500 MHz, $CDCl_3$) δ 7.92 (d, *J* = 7.8 Hz, 1H), 7.60 (td, *J* = 7.6, 1.3 Hz, 1H), 7.53 – 7.49 (m, 1H), 7.38 (d, *J* = 7.6 Hz, 1H), 4.09 (s, 2H), 3.28 (s, 3H).

^{13}C{^{1}H} NMR (126 MHz, $CDCl_3$) δ 168.3, 135.9, 133.7, 131.3, 128.8, 128.1, 123.1, 39.5, 27.2.

HRMS (ESI$^-$) m/z calc'd for $C_9H_8NO_3S^-$: 210.0225; found: 210.0224 $[M-H^+]^-$

m.p. 88 – 91 °C (lit. 89 - 91 °C)[20]

GC (FID):

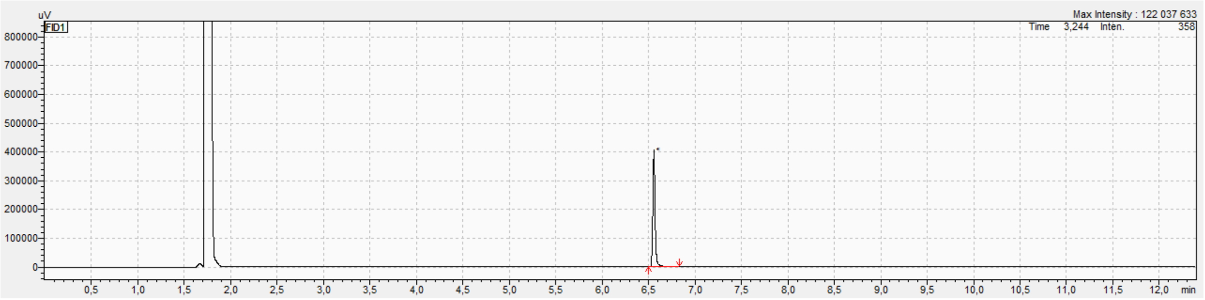

2-benzyl-2*H*-benzo[*e*][1,2]thiazin-3(4*H*)-one 1,1-dioxide (S16)

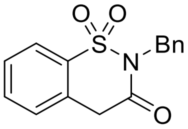

The title compound was synthesised according to General Procedure C, from *tert*-butyl (*o*-toylsulfonyl)carbamate (2.17 g, 8.00 mmol) in 43% yield (988 mg) as a white solid. Eluent for column chromatography (SiO_2): gradually from 10% of EtOAc in hexane to 20%.

^{1}H NMR (600 MHz, $CDCl_3$) δ 7.93 (dd, *J* = 7.8, 1.3 Hz, 1H), 7.59 (td, *J* = 7.6, 1.3 Hz, 1H), 7.52 – 7.49 (m, 1H), 7.42 – 7.40 (m, 2H), 7.38 – 7.36 (m, 1H), 7.32 – 7.29 (m, 2H), 7.27 – 7.24 (m, 1H), 5.00 (s, 2H), 4.12 (s, 2H).

^{13}C{^{1}H} NMR (151 MHz, $CDCl_3$) δ 168.2, 136.4, 136.0, 133.7, 131.4, 128.9, 128.7, 128.6, 128.1, 128.0, 123.0, 45.4, 39.9.

HRMS (ESI$^-$) m/z calc'd for $C_{15}H_{12}NO_3S^-$: 286.0538; found: 286.0537 $[M-H^+]^-$

m.p. 149 – 152 °C (lit. 152 – 155 °C)[4]

GC (FID):

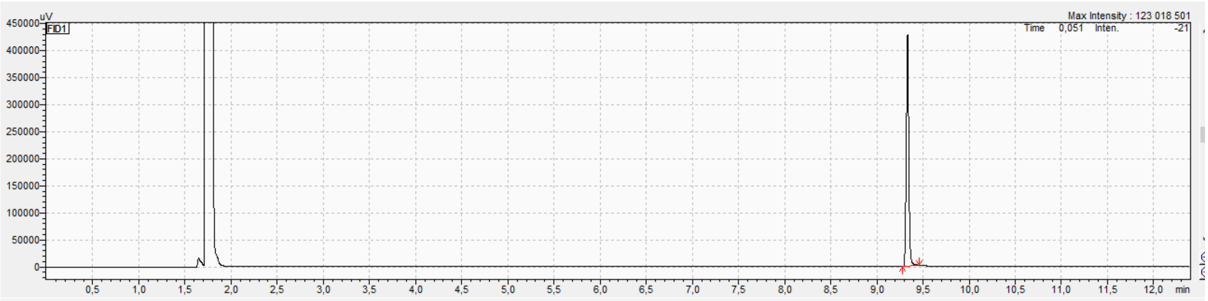

2-allyl-2H-benzo[*e*][1,2]thiazin-3(4*H*)-one 1,1-dioxide (S17)

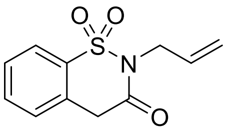

The title compound was synthesised according to General Procedure C, from *tert*-butyl (*o*-toylsulfonyl)carbamate (2.71 g, 10.0 mmol) in 45% yield (1.06 g) as a viscous colourless oil. Eluent for column chromatography (SiO_2): gradually from 10% of EtOAc in hexane to 20%.

^{1}H NMR (500 MHz, $CDCl_3$) δ 7.91 (d, *J* = 7.7 Hz, 1H), 7.60 (td, *J* = 7.6, 1.4 Hz, 1H), 7.51 (t, *J* = 7.6 Hz, 1H), 7.38 (d, *J* = 7.6 Hz, 1H), 5.88 (ddt, *J* = 17.2, 10.2, 5.7 Hz, 1H), 5.30 (dq, *J* = 17.1, 1.4 Hz, 1H), 5.21 (dq, *J* = 10.3, 1.2 Hz, 1H), 4.45 (dt, *J* = 5.8, 1.5 Hz, 2H), 4.12 (s, 2H).

^{13}C{^{1}H} NMR (126 MHz, $CDCl_3$) δ 168.0, 136.4, 133.6, 131.7, 131.4, 128.9, 128.1, 123.0, 118.8, 44.1, 39.8.

HRMS (ESI$^-$) m/z calc'd for $C_{11}H_{10}NO_3S^-$: 236.0381; found: 236.0384 $[M-H^+]^-$

GC (FID):

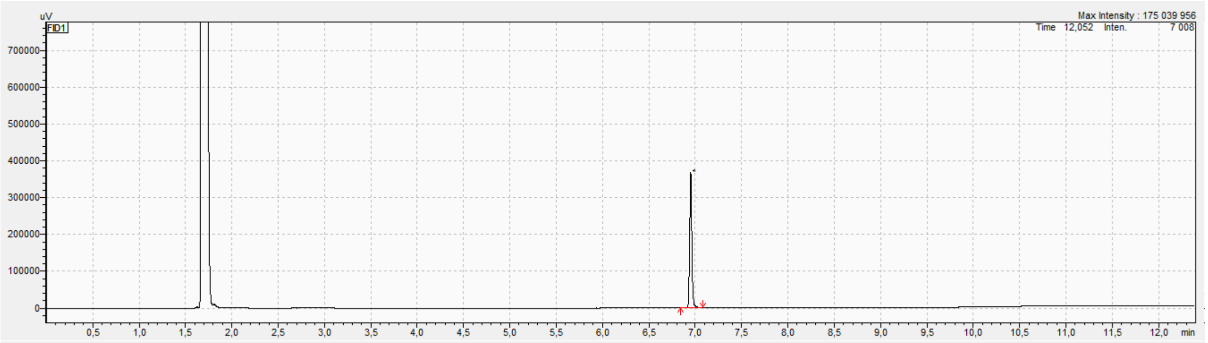

9. 4-Diazo-2-methylisoquinoline-1,3(2H,4H)-diones

4-Diazo-2-methylisoquinoline-1,3(2H,4H)-dione (**1a**), 4-diazo-2-ethylisoquinoline-1,3(2H,4H)-dione (**1b**), 4-diazo-2-propylisoquinoline-1,3(2H,4H)-dione (**1c**), 4-diazo-2-isopropylisoquinoline-1,3(2H,4H)-dione (**1d**), 2-benzyl-4-diazoisoquinoline-1,3(2H,4H)-dione (**1e**), 4-diazo-2-isopropylisoquinoline-1,3(2H,4H)-dione (**1f**) were synthesised according to the procedure reported by Kantin et al.[5]

Note: The ^{13}C NMR peak for the C=N_2 moiety is not observed in any case and this is consistent with literature reports.[5]

7-chloro-4-diazo-2-methylisoquinoline-1,3(2*H*,4*H*)-dione (1g)

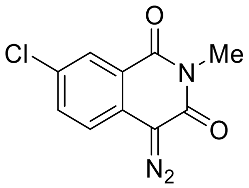

The title compound was synthesised according to General Procedure DA from 7-chloro-2-methylisoquinoline-1,3(2*H*,4*H*)-dione (839 mg, 4.00 mmol) in 69% yield (648 mg) as a yellow solid. Eluent for column chromatography (SiO_2): 20% EtOAc in hexane.

^{1}H NMR (500 MHz, $CDCl_3$) δ 8.27 (d, *J* = 2.3 Hz, 1H), 7.61 (dd, *J* = 8.4, 2.3 Hz, 1H), 7.07 (d, *J* = 8.4 Hz, 1H), 3.46 (s, 3H).

^{13}C{^{1}H} NMR (126 MHz, $CDCl_3$) δ 162.4, 162.0, 134.5, 131.9, 130.0, 125.0, 122.1, 120.1, 27.7.

HRMS (APCI$^+$) m/z calc'd for $C_{10}H_7N_3O_2Cl^+$: 236.0227; found 236.0228 $[M+H]^+$

7-bromo-4-diazo-2-methylisoquinoline-1,3(2*H*,4*H*)-dione (1h)

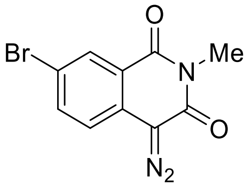

The title compound was synthesised according to General Procedure DA from 7-bromo-2-methylisoquinoline-1,3(2*H*,4*H*)-dione (867 mg, 3.41 mmol) in 74% yield (700 mg) as a yellow solid. Eluent for column chromatography (SiO_2): 20% EtOAc in hexane.

^{1}H NMR (500 MHz, $CDCl_3$) δ 8.41 (d, *J* = 2.1 Hz, 1H), 7.75 (dd, *J* = 8.4, 2.1 Hz, 1H), 7.00 (d, *J* = 8.4 Hz, 1H), 3.45 (s, 3H).

^{13}C{^{1}H} NMR (126 MHz, $CDCl_3$) δ 162.4, 161.9, 137.2, 133.0, 125.5, 122.3, 120.3, 119.3, 27.7.

HRMS (APCI$^+$) m/z calc'd for $C_{10}H_7N_3O_2Br^+$: 279.9722; found: 279.9720 $[M+H]^+$

4-diazo-2,7-dimethylisoquinoline-1,3(2*H*,4*H*)-dione (1i)

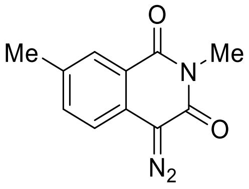

The title compound was synthesised according to General Procedure DA from 2,7-dimethylisoquinoline-1,3(2*H*,4*H*)-dione (269 mg, 1.42 mmol) in 95% yield (290 mg) as an orange solid. Eluent for column chromatography (SiO_2): 20% EtOAc in hexane.

^{1}H NMR (500 MHz, $CDCl_3$) δ 8.11 (s, 1H), 7.47 (dd, *J* = 8.1, 1.9 Hz, 1H), 7.01 (d, *J* = 8.0 Hz, 1H), 3.45 (s, 3H), 2.43 (s, 3H).

^{13}C{^{1}H} NMR (126 MHz, $CDCl_3$) δ 163.21, 163.15, 136.0, 135.4, 130.3, 123.5, 120.9, 118.7, 27.5, 21.2.

HRMS (APCI$^+$) m/z calc'd for $C_{11}H_{10}N_3O_2^+$: 216.0773; found: 216.0771 $[M+H]^+$

4-diazo-7-methoxy-2-methylisoquinoline-1,3(2*H*,4*H*)-dione (1j)

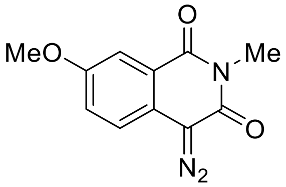

The title compound was synthesised according to General Procedure DA from 7-methoxy-2-methylisoquinoline-1,3(2*H*,4*H*)-dione (600 mg, 2.92 mmol) in 95% yield (640 mg) as an orange solid. Eluent for column chromatography (SiO_2): gradually from 20% of EtOAc in hexane to 30%.

^{1}H NMR (500 MHz, $CDCl_3$) δ 7.78 (d, *J* = 2.8 Hz, 1H), 7.27 – 7.24 (m, 1H), 7.03 (d, *J* = 8.7 Hz, 1H), 3.89 (s, 3H), 3.46 (s, 3H).

^{13}C{^{1}H} NMR (126 MHz, $CDCl_3$) δ 163.3, 163.0, 158.1, 123.6, 122.0, 120.3, 118.6, 112.2, 55.9, 27.6.

HRMS (APCI$^+$) m/z calc'd for $C_{11}H_{10}N_3O_3^+$: 232.0722; found: 232.0723 $[M+H]^+$

4-diazo-6-methoxy-2-methylisoquinoline-1,3(2*H*,4*H*)-dione (1k)

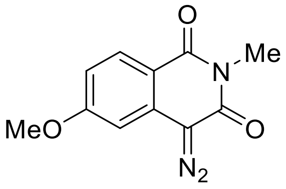

The title compound was synthesised according to General Procedure DA from 6-methoxy-2-methylisoquinoline-1,3(2*H*,4*H*)-dione (419 mg, 2.00 mmol) in 30% yield (210 mg) as an orange solid. Eluent for column chromatography (SiO_2): gradually from 20% of EtOAc in hexane to 30%.

^{1}H NMR (500 MHz, $CDCl_3$) δ 8.21 (d, *J* = 8.8 Hz, 1H), 6.86 (dd, *J* = 8.9, 2.4 Hz, 1H), 6.49 (d, *J* = 2.4 Hz, 1H), 3.90 (s, 3H), 3.43 (s, 3H).

^{13}C{^{1}H} NMR (126 MHz, $CDCl_3$) δ 164.4, 163.0, 162.8, 132.5, 128.7, 114.3, 113.0, 102.6, 55.9, 27.4.

HRMS (APCI$^+$) m/z calc'd for $C_{11}H_{10}N_3O_3^+$: 232.0722; found: 232.0720 $[M+H]^+$

6-chloro-4-diazo-2-methylisoquinoline-1,3(2*H*,4*H*)-dione (1l)

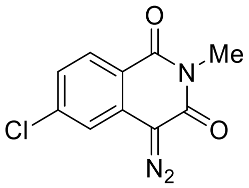

The title compound was synthesised according to General Procedure DA from 6-chloro-2-methylisoquinoline-1,3(2*H*,4*H*)-dione (560 mg, 2.67 mmol) in 81% yield (510 mg) as a yellow solid. Eluent for column chromatography (SiO_2): 20% EtOAc in hexane.

^{1}H NMR (500 MHz, $CDCl_3$) δ 8.22 (d, *J* = 8.5 Hz, 1H), 7.29 (dd, *J* = 8.5, 1.9 Hz, 1H), 7.11 (d, *J* = 1.9 Hz, 1H), 3.45 (s, 3H).

^{13}C{^{1}H} NMR (126 MHz, $CDCl_3$) δ 162.4, 162.3, 141.0, 131.8, 128.4, 126.4, 119.2, 118.5, 27.6.

HRMS (APCI$^+$) m/z calc'd for $C_{10}H_7N_3O_2Cl^+$: 236.0227; found: 236.0230 $[M+H]^+$

6-bromo-4-diazo-2-methylisoquinoline-1,3(2*H*,4*H*)-dione (1m)

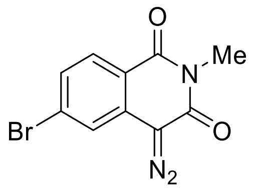

The title compound was synthesised according to General Procedure DA from 6-bromo-2-methylisoquinoline-1,3(2*H*,4*H*)-dione (762 mg, 3.0 mmol) in 62% yield (521 mg) as a yellow solid. Eluent for column chromatography (SiO_2): 20% EtOAc in hexane.

^{1}H NMR (500 MHz, $CDCl_3$) δ 8.13 (d, *J* = 8.5 Hz, 1H), 7.44 (dd, *J* = 8.5, 1.8 Hz, 1H), 7.27 (d, *J* = 1.8 Hz, 1H), 3.44 (s, 3H).

^{13}C{^{1}H} NMR (126 MHz, $CDCl_3$) δ 162.5, 162.2, 131.7, 129.5, 129.2, 128.4, 121.4, 119.6, 27.6.

HRMS (APCI$^+$) m/z calc'd for $C_{10}H_7N_3O_2Br^+$: 279.9722; found: 279.9723 $[M+H]^+$

8-chloro-4-diazo-2-methylisoquinoline-1,3(2*H*,4*H*)-dione (1n)

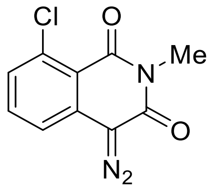

The title compound was synthesised according to General Procedure DA from 8-chloro-2-methylisoquinoline-1,3(2*H*,4*H*)-dione (419 mg, 2.00 mmol) in 59% yield (276 mg) as an orange solid. Eluent for column chromatography (SiO_2): 20% EtOAc in hexane.

^{1}H NMR (500 MHz, $CDCl_3$) δ 7.51 (t, *J* = 8.0 Hz, 1H), 7.36 (dd, *J* = 7.9, 1.1 Hz, 1H), 7.02 (dd, *J* = 7.9, 1.1 Hz, 1H), 3.44 (s, 3H).

^{13}C{^{1}H} NMR (126 MHz, $CDCl_3$) δ 161.7, 161.0, 138.4, 133.6, 129.7, 129.5, 117.5, 117.4, 27.7.

HRMS (ESI$^+$) *m/z* calc'd for $C_{10}H_6N_3O_2ClNa^+$: 258.0046; found: 258.0048 $[M+Na]^+$

5-chloro-4-diazo-2-methylisoquinoline-1,3(2*H*,4*H*)-dione (1o)

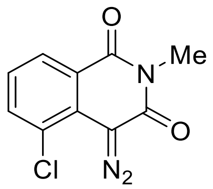

The title compound was synthesised according to General Procedure DA from 5-chloro-2-methylisoquinoline-1,3(2*H*,4*H*)-dione (839 mg, 4.00 mmol) in 96% yield (900 mg) as an orange solid. Eluent for column chromatography (SiO_2): 20% EtOAc in hexane.

^{1}H NMR (500 MHz, $CDCl_3$) δ 8.23 (d, *J* = 7.9 Hz, 1H), 7.58 (d, *J* = 7.9 Hz, 1H), 7.23 (t, *J* = 7.9 Hz, 1H), 3.46 (s, 3H).

^{13}C{^{1}H} NMR (126 MHz, $CDCl_3$) δ 163.2, 162.0, 135.5, 129.1, 126.7, 126.2, 124.3, 122.5, 28.1.

HRMS (ESI$^+$) *m/z* calc'd for $C_{10}H_6N_3O_2NaCl^+$: 258.0046; found: 258.0047 [M+Na]$^+$

10. 4-Diazo-2*H*-benzo[*e*][1,2]thiazin-3(4*H*)-one 1,1-dioxide

2-benzyl-4-diazo-2H-benzo[*e*][1,2]thiazin-3(4*H*)-one 1,1-dioxide (34a)

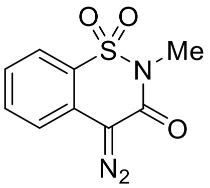

The title compound was synthesised according to General Procedure DB from 2-methyl-2*H*-benzo[*e*][1,2]thiazin-3(4*H*)-one 1,1-dioxide (423 mg, 2.00 mmol) in 66% yield (314 mg) as an orange solid. Eluent for column chromatography (SiO_2): 20% EtOAc in hexane.

^{1}H NMR (500 MHz, $CDCl_3$) δ 7.99 – 7.97 (m, 1H), 7.69 – 7.66 (m, 1H), 7.41 – 7.38 (m, 1H), 7.18 – 7.16 (m, 1H), 3.39 (s, 3H).

^{13}C{^{1}H} NMR (126 MHz, $CDCl_3$) δ 161.7, 134.0, 129.2, 126.1, 124.2, 123.2, 120.2, 26.8.

HRMS (APCI$^+$) *m/z* calc'd for $C_9H_8N_3O_3S^+$: 238.0286; found: 238.0284 [M+H]$^+$

2-benzyl-4-diazo-2H-benzo[*e*][1,2]thiazin-3(4*H*)-one 1,1-dioxide (34b)

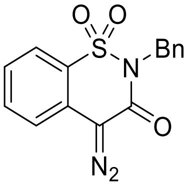

The title compound was synthesised according to General Procedure DB from 2-benzyl-2*H*-benzo[*e*][1,2]thiazin-3(4*H*)-one 1,1-dioxide (575 mg, 2.00 mmol) in 45% yield (284 mg) as an orange solid. Eluent for column chromatography (SiO_2): 20% EtOAc in hexane.

^{1}H NMR (500 MHz, $CDCl_3$) δ 7.99 – 7.96 (m, 1H), 7.66 – 7.63 (m, 1H), 7.48 – 7.46 (m, 2H), 7.40 – 7.36 (m, 1H), 7.33 – 7.30 (m, 2H), 7.28 – 7.25 (m, 1H), 7.14 (d, *J* = 8.0 Hz, 1H), 5.12 (s, 2H).

^{13}C{^{1}H} NMR (126 MHz, $CDCl_3$) δ 161.7, 135.9, 133.9, 129.7, 129.0, 128.6, 128.1, 126.1, 124.1, 123.2, 120.3, 44.8.

HRMS (APCI$^+$) *m/z* calc'd for $C_{15}H_{12}N_3O_3S^+$: 314.0599; found: 314.0598 [M+H]$^+$

4-diazo-2-allyl-2*H*-benzo[*e*][1,2]thiazin-3(4*H*)-one 1,1-dioxide (34c)

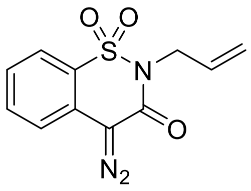

The title compound was synthesised according to General Procedure DB from 2-allyl-2*H*-benzo[*e*][1,2]thiazin-3(4*H*)-one 1,1-dioxide (475 mg, 2.00 mmol) in 86% yield (453 mg) as an orange solid. Eluent for column chromatography (SiO_2): 20% EtOAc in hexane.

^{1}H NMR (500 MHz, $CDCl_3$) δ 7.98 – 7.96 (m, 1H), 7.67 (td, *J* = 8.0, 1.3 Hz, 1H), 7.41 – 7.38 (m, 1H), 7.18 – 7.16 (m, 1H), 5.96 (ddt, *J* = 16.5, 10.3, 6.1 Hz, 1H), 5.38 (dq, *J* = 17.2, 1.4 Hz, 1H), 5.27 (dq, *J* = 10.2, 1.2 Hz, 1H), 4.56 (dt, *J* = 6.1, 1.4 Hz, 2H).

^{13}C{^{1}H} NMR (151 MHz, $CDCl_3$) δ 161.5, 133.9, 132.0, 129.8, 126.1, 124.0, 123.3, 120.3, 119.3, 44.1.

HRMS (APCI$^+$) *m/z* calc'd for $C_{11}H_{10}N_3O_3S$: 264.0443$^+$; found: 264.0447 $[M+H]^+$

10. O-H Insertion Products

4-((1,1,1,3,3,3-hexafluoropropan-2-yl)oxy)-2-methylisoquinoline-1,3(2*H*,4*H*)-dione (2)

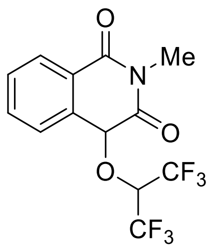

The title compound was synthesised according to General Procedure E from 4-diazo-2-methylisoquinoline-1,3(2*H*,4*H*)-dione (40 mg, 0.20 mmol) and HFIP (2 mL) in 67% yield (46 mg) as a white solid. Eluent for column chromatography (SiO_2): 10% EtOAc in hexane.

For gram-scale reaction: 4-diazo-2-methylisoquinoline-1,3(2*H*,4*H*)-dione (1.00 g, 4.97 mmol) is charged in a 500 mL flask followed by the addition of HFIP (50 mL). The flask is irradiated with the whole blue LED plate for 7 hours. After completion of the reaction, HFIP is removed via distillation affording 41 mL of HFIP. The crude residue is subjected to column chromatography affording the title compound as a white solid in 66% yield (1.12 g) as a yellow solid. Eluent for column chromatography (SiO_2): 10% EtOAc in hexane.

^{1}H NMR (500 MHz, $CDCl_3$) δ 8.23 (d, *J* = 7.7 Hz, 1H), 7.74 – 7.71 (m, 1H), 7.63 – 7.59 (m, 2H), 5.45 (hept, *J* = 5.9 Hz, 1H), 5.35 (s, 1H), 3.37 (s, 3H).

^{13}C{^{1}H} NMR (126 MHz, $CDCl_3$) δ 169.8, 163.5, 134.5, 132.8, 130.4, 129.3, 128.1, 125.2, 124.3 – 117.5 (m), 74.6 (obs. p, *J* = 31.9 Hz), 74.2, 27.1.

^{19}F NMR (470 MHz, $CDCl_3$) δ -73.0 (qd, *J* = 9.0, 5.9 Hz, 3F), -73.4 (qd, *J* = 9.1, 5.8 Hz, 3F).

HRMS (ESI$^-$) *m/z* calc'd for $C_{13}H_8NO_3F_6^-$: 340.0408; found: 340.0414 $[M-H^+]^-$

m.p. 117 – 120 °C

GC (FID):

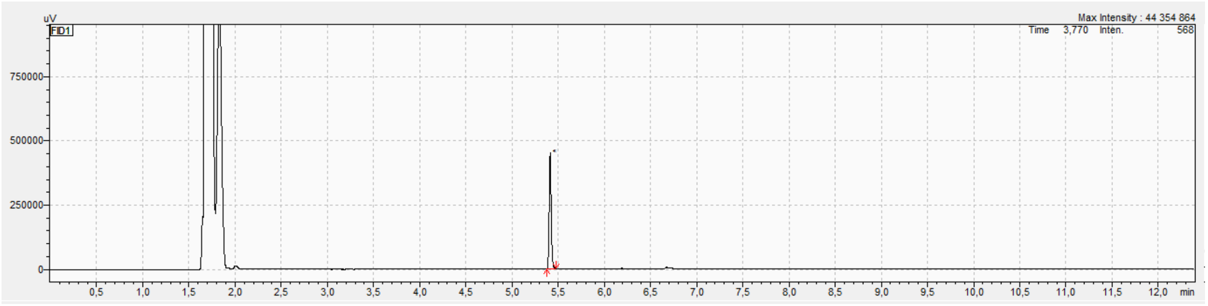

2-ethyl-4-((1,1,1,3,3,3-hexafluoropropan-2-yl)oxy)isoquinoline-1,3(2*H*,4*H*)-dione (3)

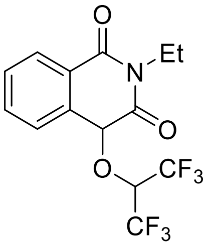

The title compound was synthesised according to General Procedure E from 4-diazo-2-ethylisoquinoline-1,3(2*H*,4*H*)-dione (43 mg, 0.20 mmol) and HFIP (2 mL) in 71% yield (51 mg) as a yellow solid. Eluent for column chromatography (SiO_2): 10% EtOAc in hexane.

^{1}H NMR (500 MHz, $CDCl_3$) δ 8.23 (dd, *J* = 8.3, 1.3 Hz, 1H), 7.74 – 7.70 (m, 1H), 7.60 (t, *J* = 7.4 Hz, 2H), 5.46 (hept, *J* = 5.9 Hz, 1H), 5.32 (s, 1H), 4.03 (q, *J* = 7.1 Hz, 2H), 1.24 (t, *J* = 7.1 Hz, 3H).

^{13}C{^{1}H} NMR (126 MHz, $CDCl_3$) δ 169.2, 162.9, 134.3, 132.7, 130.3, 129.1, 128.0, 125.2, 123.2 – 119.6 (m), 74.3 (obs. p, J = 32.3 Hz), 74.0, 35.7, 13.0.

^{19}F NMR (470 MHz, $CDCl_3$) δ -73.1 (qd, *J* = 9.1, 5.8 Hz, 3F), -73.4 (qd, *J* = 9.1, 5.8 Hz, 3F).

HRMS (ESI$^-$) *m/z* calc'd for $C_{14}H_{10}NO_3F_6^-$: 354.0565; found: 354.0564 $[M-H^+]^-$

m.p. 69 – 71 °C

GC (FID):

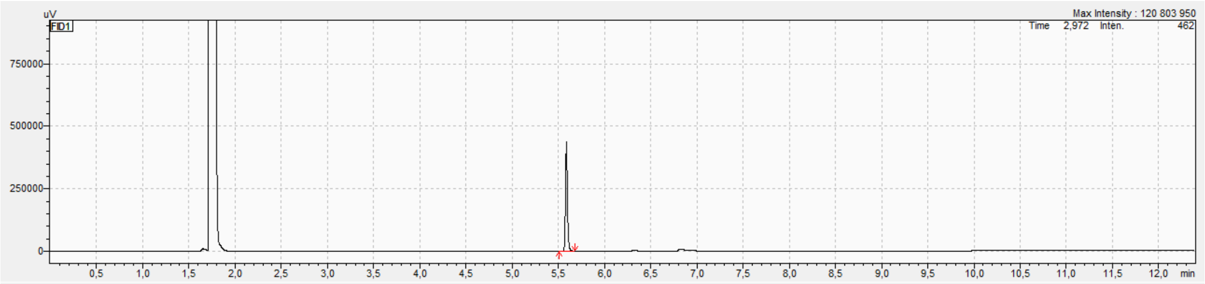

4-((1,1,1,3,3,3-hexafluoropropan-2-yl)oxy)-2-propylisoquinoline-1,3(2*H*,4*H*)-dione (4)

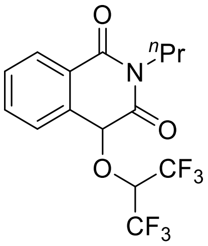

The title compound was synthesised according to General Procedure E from 4-diazo-2-propylisoquinoline-1,3(2*H*,4*H*)-dione (46 mg, 0.20 mmol) and HFIP (2 mL) in 69% yield (51 mg) as a white solid. Eluent for column chromatography (SiO_2): 10% EtOAc in hexane.

^{1}H NMR (500 MHz, $CDCl_3$) δ 8.24 – 8.22 (m, 1H), 7.74 – 7.71 (m, 1H), 7.62 – 7.58 (m, 2H), 5.47 (hept, *J* = 5.9 Hz, 1H), 5.33 (s, 1H), 3.94 – 3.91 (m, 2H), 1.69 – 1.62 (m, 2H), 0.96 (t, *J* = 7.4 Hz, 3H).

^{13}C{^{1}H} NMR (126 MHz, $CDCl_3$) δ 169.6, 163.3, 134.5, 132.8, 130.4, 129.3, 128.1, 125.3, 124.3 – 119.8 (m), 74.6 (obs. p, J = 32.8 Hz), 74.1, 42.2, 21.3, 11.4.

^{19}F NMR (470 MHz, $CDCl_3$) δ -73.0 (qd, *J* = 9.0, 5.9 Hz, 3F), -73.4 (qd, *J* = 9.0, 5.7 Hz, 3F).

HRMS (ESI$^-$) *m/z* calc'd for $C_{15}H_{12}NO_3F_6^-$: 368.0721; found: 368.0731 $[M-H^+]^-$

m.p. 59 – 62 °C

GC (FID):

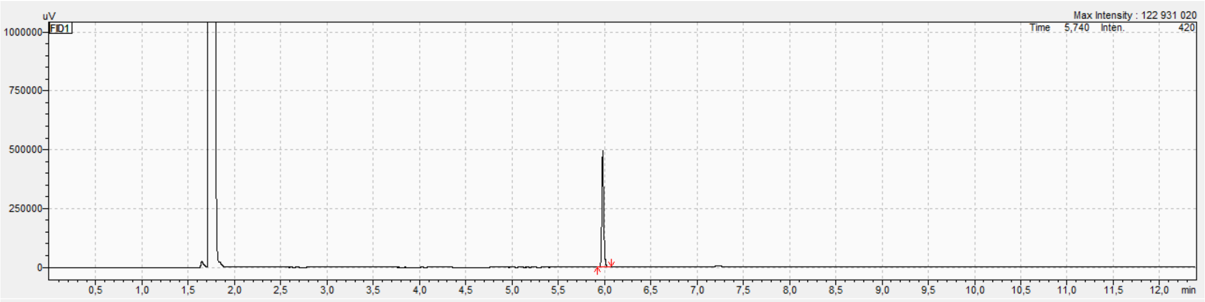

4-((1,1,1,3,3,3-hexafluoropropan-2-yl)oxy)-2-isopropylisoquinoline-1,3(2*H*,4*H*)-dione (5)

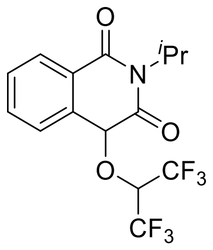

The title compound was synthesised according to General Procedure E from 4-diazo-2-isopropylisoquinoline-1,3(2*H*,4*H*)-dione (46 mg, 0.20 mmol) and HFIP (2 mL) in 60% yield (44 mg) as a white solid. Eluent for column chromatography (SiO_2): 10% EtOAc in hexane.

^{1}H NMR (500 MHz, $CDCl_3$) δ 8.21 (d, *J* = 7.9 Hz, 1H), 7.70 (td, *J* = 7.6, 1.4 Hz, 1H), 7.61 – 7.57 (m, 2H), 5.39 (hept, *J* = 5.9 Hz, 1H), 5.26 (s, 1H), 5.13 (hept, *J* = 7.0 Hz, 1H), 1.48 (dd, *J* = 6.9, 1.6 Hz, 6H).

^{13}C{^{1}H} NMR (126 MHz, $CDCl_3$) δ 169.8, 163.6, 134.3, 132.7, 130.4, 129.3, 128.0, 126.0, 124.3 – 119.8 (m), 74.8, 74.5 (obs. p, *J* = 32.8 Hz), 46.2, 19.63, 19.61.

^{19}F NMR (470 MHz, $CDCl_3$) δ -73.1 (qd, *J* = 9.0, 5.8 Hz, 3F), -73.4 (qd, *J* = 9.0, 5.8 Hz, 3F).

m.p. 62 – 65 °C

HRMS (ESI$^-$) *m/z* calc'd for $C_{15}H_{12}NO_3F_6^-$: 368.0721; found: 368.0728 $[M-H^+]^-$

GC (FID):

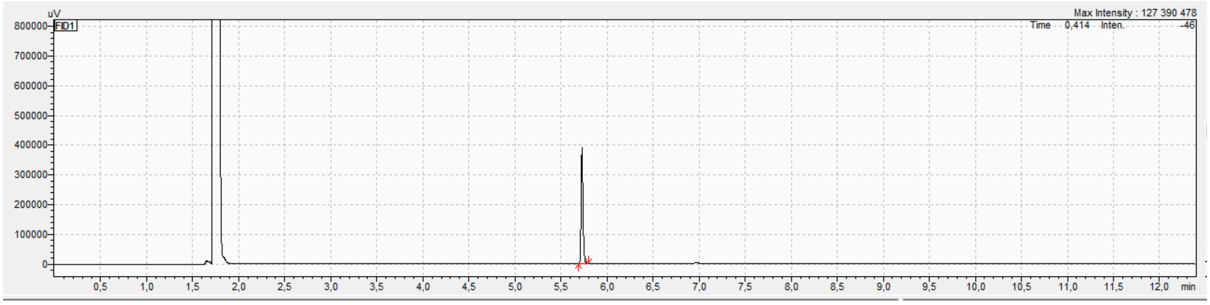

2-benzyl-4-((1,1,1,3,3,3-hexafluoropropan-2-yl)oxy)isoquinoline-1,3(2*H*,4*H*)-dione (6)

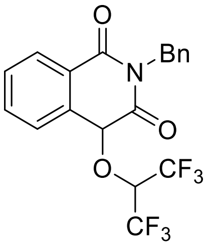

The title compound was synthesised according to General Procedure E from 2-benzyl-4-diazoisoquinoline-1,3(2*H*,4*H*)-dione (56 mg, 0.20 mmol) and HFIP (2 mL) in 63% yield (52 mg) as a white solid. Eluent for column chromatography (SiO_2): 10% EtOAc in hexane.

^{1}H NMR (500 MHz, $CDCl_3$) δ 8.24 (d, *J* = 7.9 Hz, 1H), 7.73 (td, *J* = 7.6, 1.4 Hz, 1H), 7.62 – 7.58 (m, 2H), 7.45 – 7.42 (m, 2H), 7.33 – 7.25 (m, 3H), 5.48 (hept, *J* = 5.9 Hz, 1H), 5.37 (s, 1H), 5.22 (d, *J* = 13.9 Hz, 1H), 5.10 (d, *J* = 13.9 Hz, 1H).

^{13}C{^{1}H} NMR (126 MHz, $CDCl_3$) δ 169.5, 163.2, 136.3, 134.6, 132.9, 130.4, 129.4, 129.1, 128.7, 128.1, 128.0, 125.2, 124.3 – 119.7 (m), 75.2 – 74.4 (m), 74.1 (m), 43.7.

^{19}F NMR (470 MHz, $CDCl_3$) δ -72.9 (qd, *J* = 8.7, 5.6 Hz, 3F), -73.3 (qd, *J* = 9.1, 5.7 Hz, 3F).

HRMS (ESI$^-$) *m/z* calc'd for $C_{19}H_{12}NO_3F_6^-$: 416.0721; found: 416.0728 $[M-H^+]^-$

m.p. 109 – 112 °C

GC (FID):

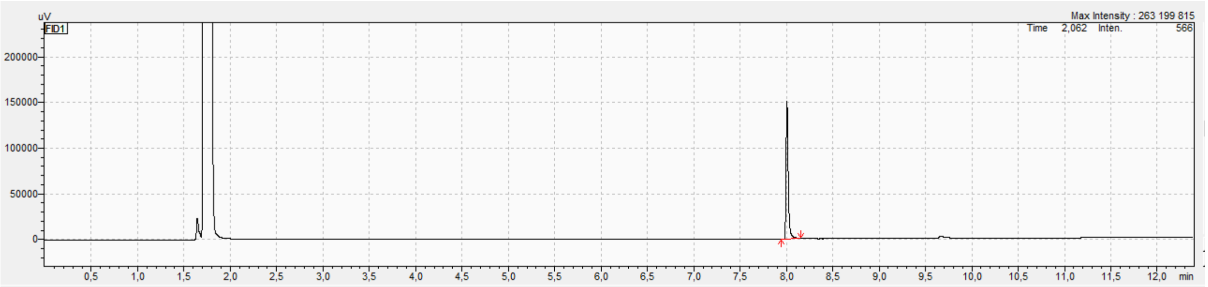

4-((1,1,1,3,3,3-hexafluoropropan-2-yl)oxy)-2-phenylisoquinoline-1,3(2*H*,4*H*)-dione (7)

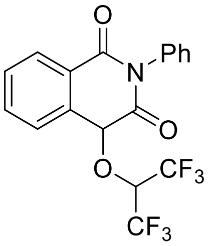

The title compound was synthesised according to General Procedure E from 4-diazo-2-phenylisoquinoline-1,3(2*H*,4*H*)-dione (53 mg, 0.20 mmol) and HFIP (2 mL) in 51% yield (21 mg) as a white solid. Eluent for column chromatography (SiO_2): 10% EtOAc in hexane.

^{1}H NMR (500 MHz, $CDCl_3$) δ 8.28 (d, *J* = 7.8 Hz, 1H), 7.80 (td, *J* = 7.6, 1.4 Hz, 1H), 7.70 (d, *J* = 7.7 Hz, 1H), 7.66 (t, *J* = 7.6 Hz, 1H), 7.55 – 7.51 (m, 2H), 7.50 – 7.46 (m, 1H), 7.22 – 7.19 (m, 2H), 5.52 (s, 1H), 5.39 (hept, *J* = 5.9 Hz, 1H).

^{13}C{^{1}H} NMR (126 MHz, $CDCl_3$) δ 169.4, 163.5, 134.9, 134.0, 132.9, 130.7, 129.7, 129.6, 129.3, 128.4, 128.3, 125.4, 125.6 – 117.5 (m), 74.9, 74.5 (obs. p, *J* = 32.5 Hz).

^{19}F NMR (470 MHz, $CDCl_3$) δ -73.0 (qd, *J* = 9.0, 5.8 Hz, 3F), -73.3 (qd, *J* = 9.0, 5.7 Hz, 3F).

HRMS (ESI$^-$) *m/z* calc'd for $C_{18}H_{10}NO_3F_6^-$: 402.0565; found: 402.0570 $[M-H^+]^-$

m.p. 119 – 121 °C

GC (FID):

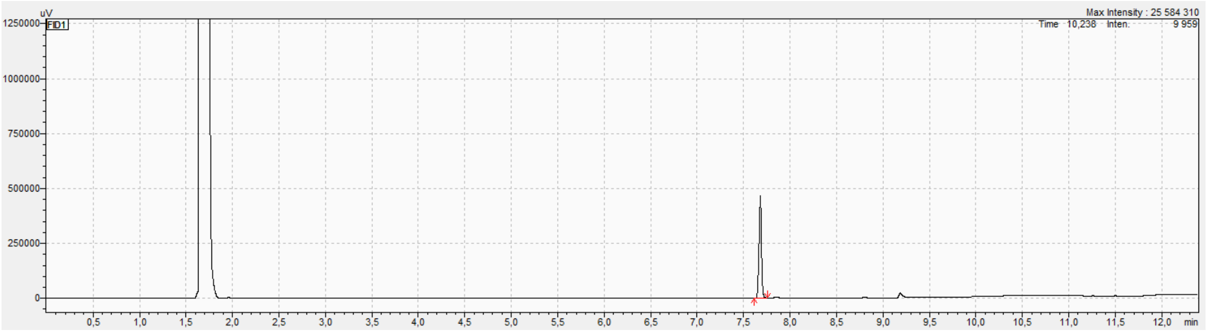

7-chloro-4-((1,1,1,3,3,3-hexafluoropropan-2-yl)oxy)-2-methylisoquinoline-1,3(2*H*,4*H*)-dione (8)

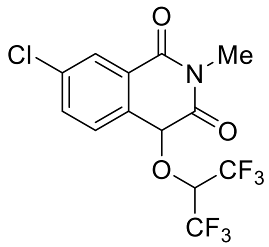

The title compound was synthesised according to General Procedure E from 7-chloro-4-diazo-2-methylisoquinoline-1,3(2*H*,4*H*)-dione (47 mg, 0.20 mmol) and HFIP (2.0 mL) in 58% yield (43 mg) as a white solid. Eluent for column chromatography (SiO_2): 10% EtOAc in hexane.

^{1}H NMR (500 MHz, $CDCl_3$) δ 8.21 (d, *J* = 2.2 Hz, 1H), 7.69 (dd, *J* = 8.3, 2.2 Hz, 1H), 7.56 (d, *J* = 8.3 Hz, 1H), 5.43 (hept, *J* = 6.0 Hz, 1H), 5.32 (s, 1H), 3.37 (s, 3H).

^{13}C{^{1}H} NMR (126 MHz, $CDCl_3$) δ 169.4, 162.4, 137.1, 134.7, 131.0, 129.6, 129.1, 126.7, 123.4 – 117.3 (m), 74.6 (obs. p, *J* = 32.5 Hz), 73.7, 27.3.

^{19}F NMR (470 MHz, $CDCl_3$) δ -73.0 – -73.1 (m, 3F), -73.4 – -73.5 (m, 3F).

HRMS (ESI$^-$) *m/z* calc'd for $C_{13}H_7NO_3F_6Cl^-$: 374.0019; found: 374.0020 $[M-H^+]^-$

m.p. 77 – 80 °C

GC (FID):

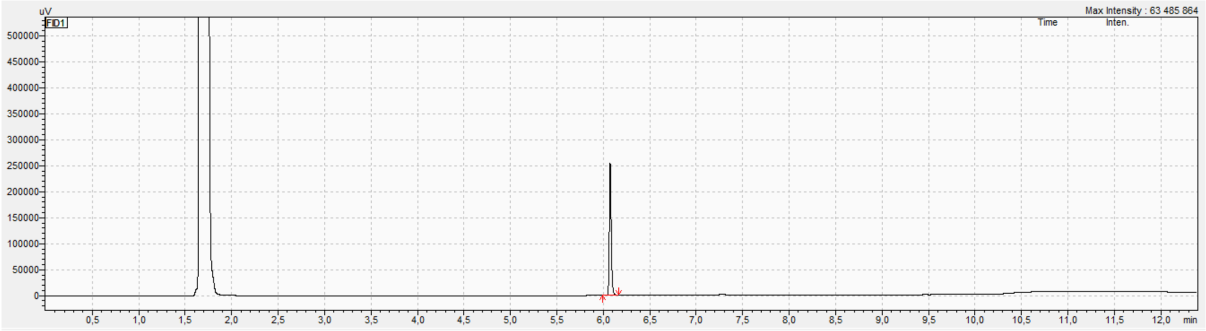

7-bromo-4-((1,1,1,3,3,3-hexafluoropropan-2-yl)oxy)-2-methylisoquinoline-1,3(2*H*,4*H*)-dione (9)

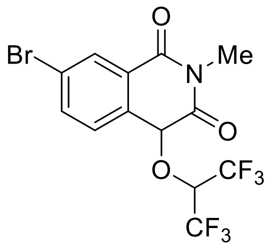

The title compound was synthesised according to General Procedure E from 7-bromo-4-diazo-2-methylisoquinoline-1,3(2*H*,4*H*)-dione (56 mg, 0.20 mmol) and HFIP (2.0 mL) in 58% yield (49 mg) as a white solid. Eluent for column chromatography (SiO_2): 10% EtOAc in hexane.

^{1}H NMR (500 MHz, $CDCl_3$) δ 8.37 (d, *J* = 2.1 Hz, 1H), 7.84 (dd, *J* = 8.3, 2.1 Hz, 1H), 7.49 (d, *J* = 8.2 Hz, 1H), 5.43 (hept, *J* = 5.9 Hz, 1H), 5.30 (s, 1H), 3.37 (s, 3H).

^{13}C{^{1}H} NMR (151 MHz, $CDCl_3$) δ 169.3, 162.3, 137.6, 132.1, 131.5, 129.7, 126.7, 124.9, 125.3 – 119.6 (m), 75.1 – 74.1 (m), 73.7, 27.3.

^{19}F NMR (470 MHz, $CDCl_3$) δ -73.0 (qd, *J* = 9.0, 5.9 Hz, 3F), -73.4 (qd, *J* = 9.0, 5.7 Hz, 3F).

HRMS (ESI$^-$) *m/z* calc'd for $C_{13}H_7NO_3F_6Br^-$: 417.9514; found: 417.9516 $[M-H^+]^-$

m.p. 96 – 99 °C

GC (FID):

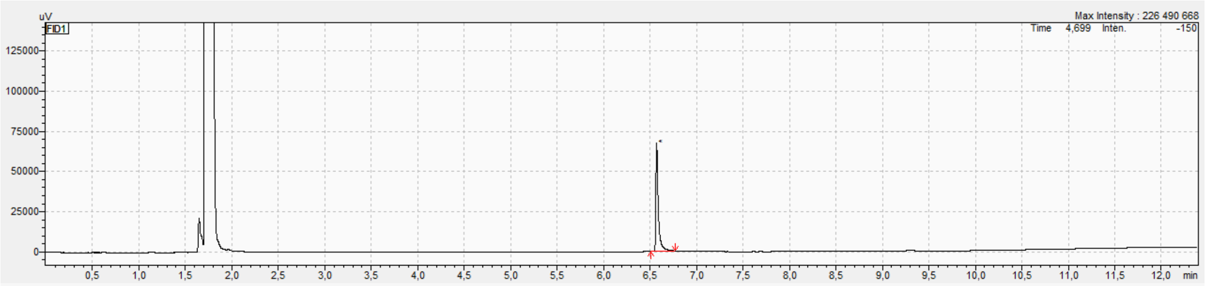

4-((1,1,1,3,3,3-hexafluoropropan-2-yl)oxy)-2,7-dimethylisoquinoline-1,3(2*H*,4*H*)-dione (10)

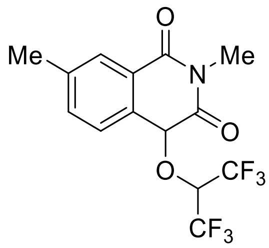

The title compound was synthesised according to General Procedure E from 4-diazo-2,7-dimethylisoquinoline-1,3(2*H*,4*H*)-dione (43 mg, 0.20 mmol) and HFIP (2 mL) in 63% yield (45 mg) as a white solid. Eluent for column chromatography (SiO_2): 10% EtOAc in hexane.

^{1}H NMR (500 MHz, $CDCl_3$) δ 8.03 (s, 1H), 7.53 – 7.48 (m, 2H), 5.41 (hept, *J* = 5.9 Hz, 1H), 5.29 (s, 1H), 3.36 (s, 3H), 2.46 (s, 3H).

^{13}C{^{1}H} NMR (126 MHz, $CDCl_3$) δ 169.9, 163.7, 141.0, 135.4, 129.9, 129.4, 128.2, 125.0, 123.6 – 119.6 (m), 74.4 (obs. p, *J* = 32.5 Hz), 74.2, 27.0, 21.4.

^{19}F NMR (470 MHz, $CDCl_3$) δ -73.1 (qd, *J* = 9.0, 5.9 Hz, 3F), -73.5 (qd, *J* = 9.0, 5.8 Hz, 3F).

HRMS (ESI$^-$) *m/z* calc'd for $C_{14}H_{10}NO_3F_6^-$: 354.0565; found: 354.0569 $[M-H^+]^-$

m.p. 117 – 119 °C

GC (FID):

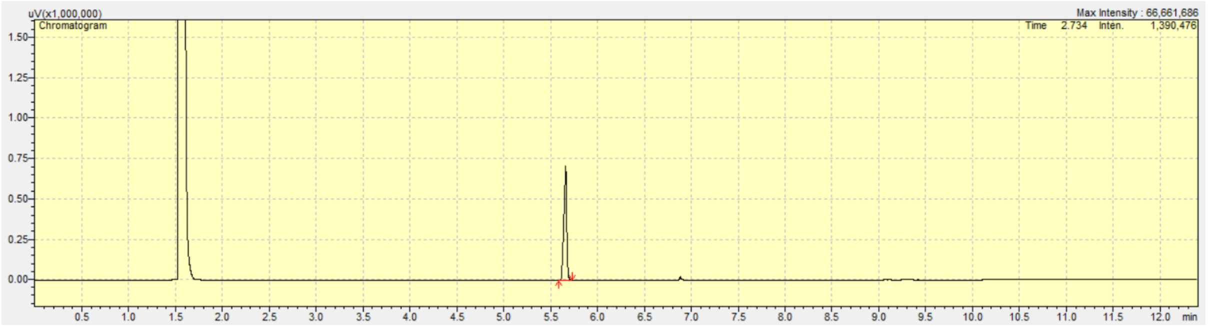

4-((1,1,1,3,3,3-hexafluoropropan-2-yl)oxy)-7-methoxy-2-methylisoquinoline-1,3(2*H*,4*H*)-dione (11)

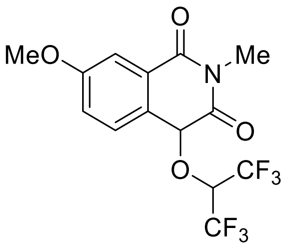

The title compound was synthesised according to General Procedure E from 4-diazo-7-methoxy-2-methylisoquinoline-1,3(2*H*,4*H*)-dione (46 mg, 0.20 mmol) and HFIP (2 mL) in 33% yield (25 mg) as a white solid. Extending the reaction time to 4.5 hours afforded the title compound in 56% yield (41 mg). Eluent for column chromatography (SiO_2): gradually from 10% of EtOAc in hexane to 15%.

^{1}H NMR (500 MHz, $CDCl_3$) δ 7.68 (d, *J* = 2.7 Hz, 1H), 7.49 (d, *J* = 8.5 Hz, 1H), 7.25 (dd, *J* = 8.5, 2.7 Hz, 1H), 5.39 (hept, *J* = 5.9 Hz, 1H), 5.25 (s, 1H), 3.90 (s, 3H), 3.36 (s, 3H).

^{13}C{^{1}H} NMR (126 MHz, $CDCl_3$) δ 169.8, 163.6, 161.3, 130.0, 126.7, 124.8, 122.2, 123.3 – 117.6 (m), 112.0, 74.3 (obs. p, *J* = 32.4 Hz), 74.1, 55.9, 27.1. (One carbon missing due to ^{19}F splitting).

^{19}F NMR (470 MHz, $CDCl_3$) δ -73.2 (qd, *J* = 9.1, 6.1 Hz, 3F), -73.6 (qd, *J* = 8.9, 5.5 Hz, 3F).

HRMS (ESI$^-$) *m/z* calc'd for $C_{14}H_{10}NO_4F_6^-$: 370.0514; found: 370.0519 $[M-H^+]^-$

m.p. 99 – 101 °C

GC (FID):

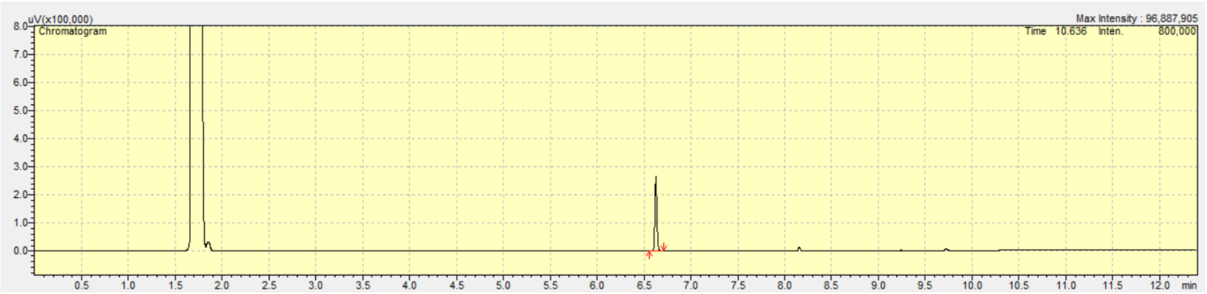

4-((1,1,1,3,3,3-hexafluoropropan-2-yl)oxy)-6-methoxy-2-methylisoquinoline-1,3(2*H*,4*H*)-dione (12)

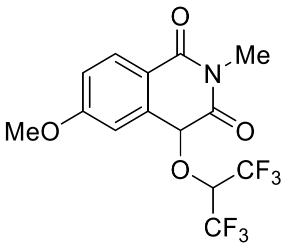

The title compound was synthesised according to General Procedure E from 4-diazo-6-methoxy-2-methylisoquinoline-1,3(2*H*,4*H*)-dione (46 mg, 0.20 mmol) and HFIP (2 mL) with an extended reaction time of 20 hours in 48% yield (36 mg) as a beige solid. Eluent for column chromatography (SiO_2): gradually from 10% of EtOAc in hexane to 15%.

^{1}H NMR (500 MHz, $CDCl_3$) δ 8.15 (d, *J* = 8.6 Hz, 1H), 7.09 – 7.05 (m, 2H), 5.46 (hept, *J* = 5.9 Hz, 1H), 5.31 (s, 1H), 3.91 (s, 3H), 3.34 (s, 3H).

^{13}C{^{1}H} NMR (126 MHz, $CDCl_3$) δ 170.0, 164.5, 163.1, 135.1, 131.5, 126.0 – 119.6 (m), 117.8, 116.6, 112.3, 75.5 – 74.0 (m), 74.2, 55.8, 26.9.

^{19}F NMR (470 MHz, $CDCl_3$) δ -73.0 (qd, *J* = 9.0, 5.9 Hz, 3F), -73.4 (qd, *J* = 9.1, 5.9 Hz, 3F).

HRMS (APCI$^+$) *m/z* calc'd for $C_{14}H_{12}NO_4F_6^+$: 372.0671; found: 372.0673 $[M+H]^+$

M.P 118 – 121 °C

GC (FID):

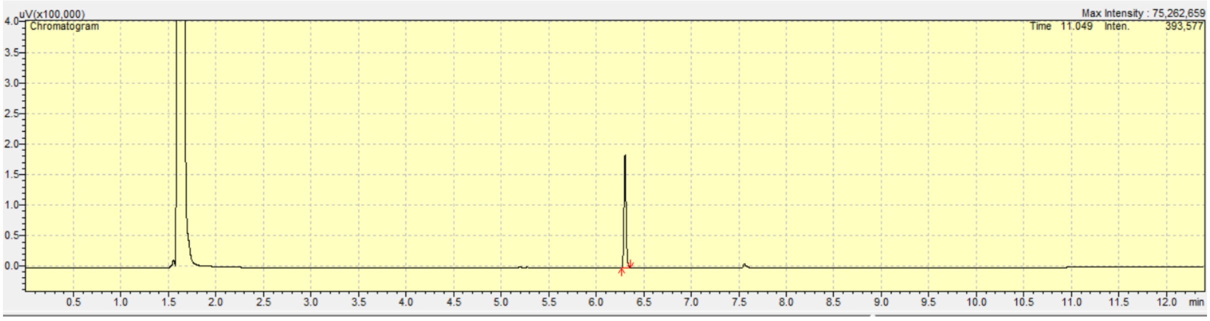

8-chloro-4-((1,1,1,3,3,3-hexafluoropropan-2-yl)oxy)-2-methylisoquinoline-1,3(2*H*,4*H*)-dione (15)

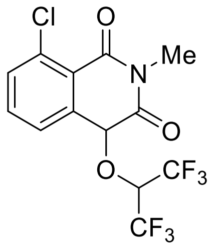

The title compound was synthesised according to General Procedure E from 8-chloro-4-diazo-2-methylisoquinoline-1,3(2*H*,4*H*)-dione (47 mg, 0.20 mmol) and HFIP (2.0 mL) in 39% yield (30 mg) as a white solid. Eluent for column chromatography (SiO_2): 10% EtOAc in hexane.

^{1}H NMR (500 MHz, $CDCl_3$) δ 7.65 (dd, *J* = 8.1, 1.5 Hz, 1H), 7.61 (t, *J* = 7.8 Hz, 1H), 7.56 – 7.54 (m, 1H), 5.36 – 5.31 (m, 2H), 3.36 (s, 3H).

^{13}C{^{1}H} NMR (126 MHz, $CDCl_3$) δ 168.5, 161.3, 136.7, 135.2, 134.3, 134.1, 127.0, 122.4, 124.2 – 119.7 (m), 74.5, 74.9 – 73.9 (m), 27.4.

^{19}F NMR (470 MHz, $CDCl_3$) δ -73.1 (qd, *J* = 9.0, 5.8 Hz, 3F), -73.5 (qd, *J* = 9.0, 5.7 Hz, 3F).

HRMS (ESI$^+$) *m/z* calc'd for $C_{13}H_8NO_3ClF_6Na^+$: 397.9995; found: 397.9991 $[M+Na]^+$

m.p. 137 – 140 °C

GC (FID):

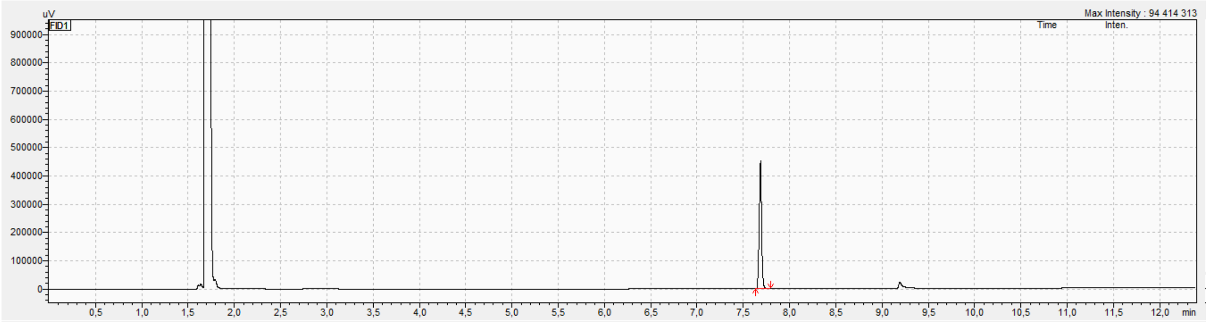

5-chloro-4-((1,1,1,3,3,3-hexafluoropropan-2-yl)oxy)-2-methylisoquinoline-1,3(2*H*,4*H*)-dione (16)

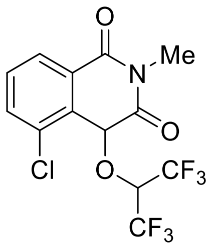

The title compound was synthesised according to General Procedure E from 5-chloro-4-diazo-2-methylisoquinoline-1,3(2*H*,4*H*)-dione (47 mg, 0.20 mmol) and HFIP (2.0 mL) in 51% yield (38 mg) as a white solid. Eluent for column chromatography (SiO_2): 10% EtOAc in hexane.

^{1}H NMR (500 MHz, $CDCl_3$) δ 8.19 (dd, *J* = 7.7, 1.3 Hz, 1H), 7.74 (dd, *J* = 8.0, 1.2 Hz, 1H), 7.60 (t, *J* = 8.0 Hz, 1H), 5.48 (s, 1H), 5.15 (hept, *J* = 6.0 Hz, 1H), 3.37 (s, 3H).

^{13}C{^{1}H} NMR (126 MHz, $CDCl_3$) δ 168.0, 162.9, 135.7, 135.3, 132.1, 129.7, 128.4, 128.3, 72.6, 72.4 (t, *J* = 33.1 Hz), 27.3. (One carbon missing due to ^{19}F splitting).

^{19}F NMR (470 MHz, $CDCl_3$) δ -72.7 (qd, *J* = 9.0, 5.9 Hz, 3F), -74.0 (qd, *J* = 9.0, 6.2 Hz, 3F).

HRMS (ESI$^-$) *m/z* calc'd for $C_{13}H_7NO_3F_6Cl^-$: 374.0019; found 374.0020 $[M-H^+]^-$

m.p. 82 – 84 °C

GC (FID):

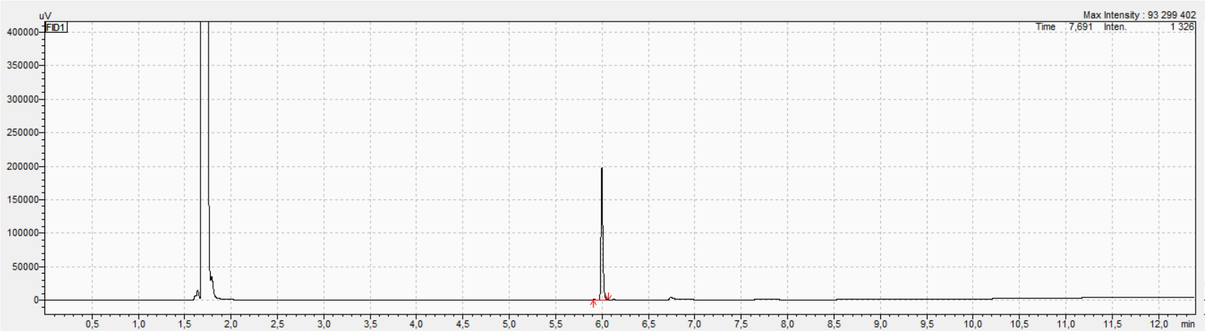

2-methyl-4-(2,2,2-trifluoroethoxy)isoquinoline-1,3(2*H*,4*H*)-dione (17)

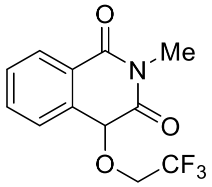

The title compound was synthesised according to General Procedure E from 4-diazo-2-methylisoquinoline-1,3(2*H*,4*H*)-dione (40 mg, 0.20 mmol) and 2,2,2-trifluoroethan-1-ol (2 mL) in 41% yield (23 mg) as a brown solid. Eluent for column chromatography (SiO_2): 10% EtOAc in hexane.

^{1}H NMR (600 MHz, $CDCl_3$) δ 8.21 – 8.19 (m, 1H), 7.72 – 7.69 (m, 1H), 7.68 – 7.67 (m, 1H), 7.57 – 7.54 (m, 1H), 5.24 (s, 1H), 4.50 (dq, *J* = 12.5, 8.7 Hz, 1H), 4.37 (dq, *J* = 12.6, 8.4 Hz, 1H), 3.36 (s, 3H).

^{13}C{^{1}H} NMR (151 MHz, $CDCl_3$) δ 171.0, 163.9, 134.4, 134.3, 129.7, 129.1, 127.0, 124.9, 122.0 (q, *J* = 278.9 Hz), 75.6, 68.9 (q, *J* = 34.3 Hz), 27.2.

^{19}F NMR (470 MHz, $CDCl_3$) δ -74.4 (t, *J* = 8.6 Hz, 3F).

HRMS (ESI$^-$) *m/z* calc'd for $C_{12}H_9NO_3F_3^-$: 272.0535; found 272.0540 $[M-H^+]^-$

m.p. 65 – 68 °C

GC (FID):

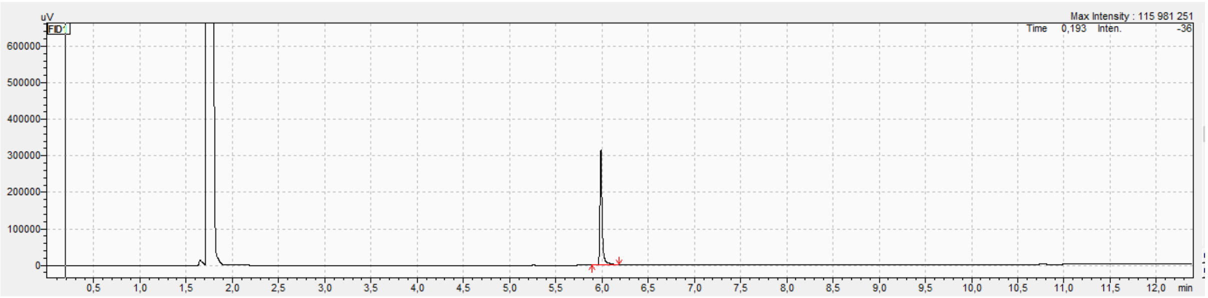

2-methyl-4-((1,1,1-trifluoropropan-2-yl)oxy)isoquinoline-1,3(2*H*,4*H*)-dione (18)

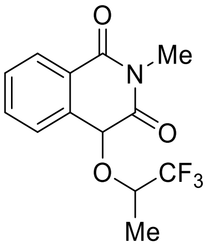

The title compound was synthesised according to General Procedure E from 4-diazo-2-methylisoquinoline-1,3(2*H*,4*H*)-dione (40 mg, 0.20 mmol) and 1,1,1-trifluoropropan-2-ol (2 mL) in 44% yield (1:10 dr, 25 mg) as a white solid. Only ^{1}H and ^{13}C peaks from the major product are reported as the intensity from the minor product is not sufficient to identify all peaks. Eluent for column chromatography (SiO_2): 10% EtOAc in hexane.

^{1}H NMR (600 MHz, $CDCl_3$) δ 8.18 (dt, *J* = 7.8, 1.0 Hz, 1H), 7.70 – 7.68 (m, 2H), 7.56 – 7.51 (m, 1H), 5.36 (s, 1H), 4.72 (hept, *J* = 6.5 Hz, 1H), 3.36 (s, 3H), 1.54 (d, *J* = 6.6, 3H).

^{13}C{^{1}H} NMR (151 MHz, $CDCl_3$) δ 171.8, 164.1, 134.9, 134.4, 129.4, 128.9, 126.9, 124.7, 76.0 (t, *J* = 30.1 Hz), 75.6, 27.1, 14.9 (q, *J* = 2.1 Hz). (One carbon missing due to ^{19}F splitting within the diastereomeric peaks).

^{19}F NMR (470 MHz, $CDCl_3$) δ -78.6 (Major, t, *J* = 6.4 Hz, 3F), -78.9 (Minor, t, *J* = 6.5 Hz, 3F).

HRMS (ESI$^-$) *m/z* calc'd for $C_{13}H_{11}NO_3F_3^-$: 286.0691; found: 286.0696 $[M-H^+]^-$

GC (FID):

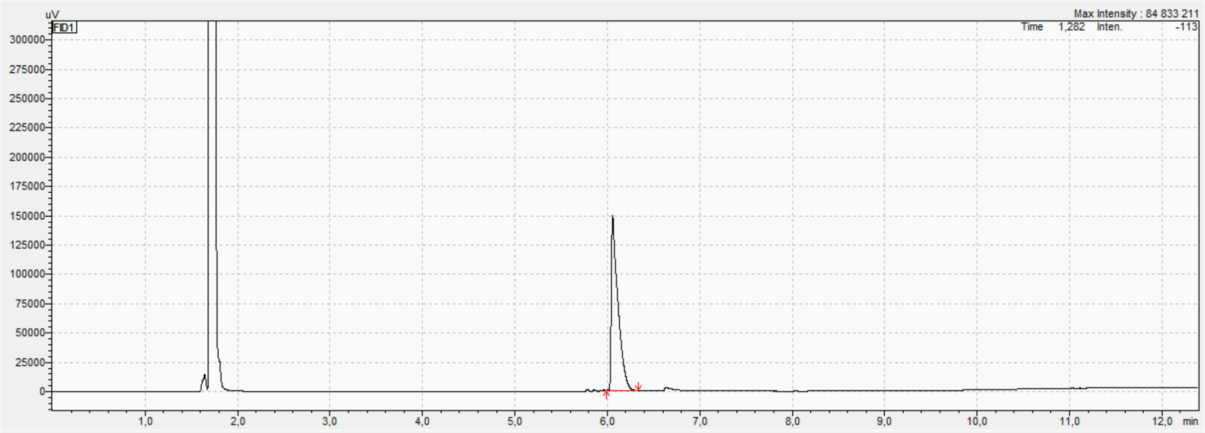

2-methyl-4-(2,2,3,3-tetrafluoropropoxy)isoquinoline-1,3(2*H*,4*H*)-dione (19)

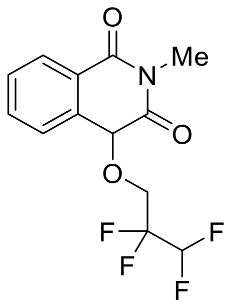

The title compound was synthesised according to General Procedure E from 4-diazo-2-methylisoquinoline-1,3(2*H*,4*H*)-dione (40 mg, 0.20 mmol) and 2,2,3,3-tetrafluoropropan-1-ol (2 mL) in 45% yield (28 mg) as a colourless oil. Eluent for column chromatography (SiO_2): 10% EtOAc in hexane.

^{1}H NMR (500 MHz, $CDCl_3$) δ 8.21 (d, *J* = 7.9 Hz, 1H), 7.71 (td, *J* = 7.5, 1.4 Hz, 1H), 7.64 – 7.62 (m, 1H), 7.58 – 7.54 (m, 1H), 6.01 (tdd, *J* = 53.2, 5.1, 4.1 Hz, 1H), 5.20 (s, 1H), 4.53 (dtd, *J* = 14.1, 11.8, 2.2 Hz, 1H), 4.27 – 4.19 (m, 1H), 3.37 (s, 3H).

^{13}C{^{1}H} NMR (126 MHz, $CDCl_3$) δ 170.7, 163.9, 134.4, 134.3, 129.8, 129.2, 126.9, 125.1, 115.0 (tt, *J* = 249.9, 27.4 Hz), 109.4 (tt, *J* = 249.9, 35.1 Hz), 75.7, 68.3 (t, *J* = 28.3 Hz), 27.2.

^{19}F NMR (470 MHz, $CDCl_3$) δ -124.2 – -125.8 (m, 2F), -137.8 – -139.7 (m, 2F).

HRMS (ESI$^-$) *m/z* calc'd for $C_{13}H_{10}NO_3F_4^-$: 304.0597; found: 304.0596 $[M-H^+]^-$

GC (FID):

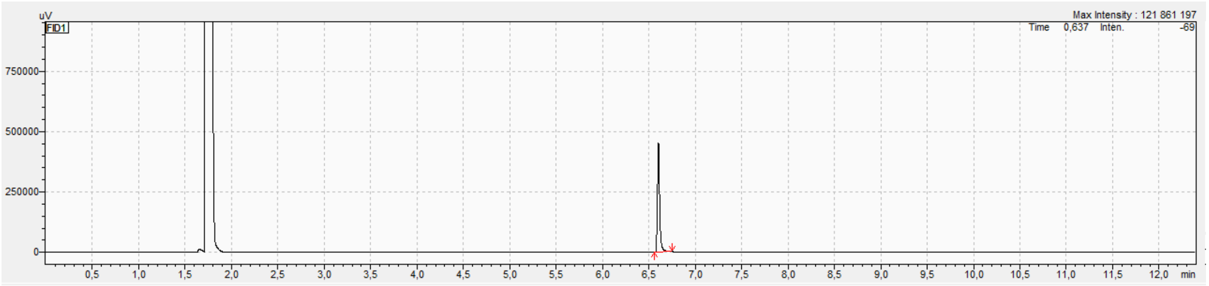

2-methyl-4-((2,2,3,3,4,4,5,5-octafluoropentyl)oxy)isoquinoline-1,3(2*H*,4*H*)-dione (20)

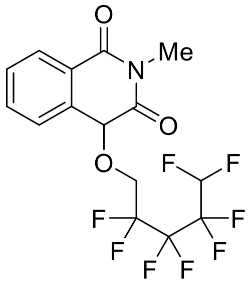

The title compound was synthesised according to General Procedure E from 4-diazo-2-methylisoquinoline-1,3(2*H*,4*H*)-dione (40 mg, 0.20 mmol) and 2,2,3,3,4,4,5,5-octafluoropentan-1-ol (2 mL) in 38% yield (31 mg) as a colourless oil. Eluent for column chromatography (SiO_2): 10% EtOAc in hexane.

^{1}H NMR (500 MHz, $CDCl_3$) δ 8.20 (d, *J* = 7.8 Hz, 1H), 7.72 – 7.67 (m, 2H), 7.57 – 7.54 (m, 1H), 6.07 (tt, *J* = 52.0, 5.4 Hz, 1H), 5.23 (s, 1H), 4.75 – 4.66 (m, 1H), 4.43 (q, *J* = 13.5 Hz, 1H), 3.37 (s, 3H).

^{13}C{^{1}H} NMR (126 MHz, $CDCl_3$) δ 171.0, 163.9, 134.4, 134.2, 129.7, 129.1, 127.0, 124.9, 118.1 – 105.4 (m), 76.0, 68.3 (t, *J* = 25.0 Hz), 27.2.

^{19}F NMR (470 MHz, $CDCl_3$) δ -120.4 (p, *J* = 12.7 Hz, 2F), -125.1 (t, *J* = 8.7 Hz, 2F), -129.9 – -130.1 (m, 2F), -137.1 – -137.3 (m, 2F).

HRMS (ESI$^-$) *m/z* calc'd for $C_{15}H_{10}NO_3F_8^-$: 404.0533; found: 404.0536 $[M-H^+]^-$

GC (FID):

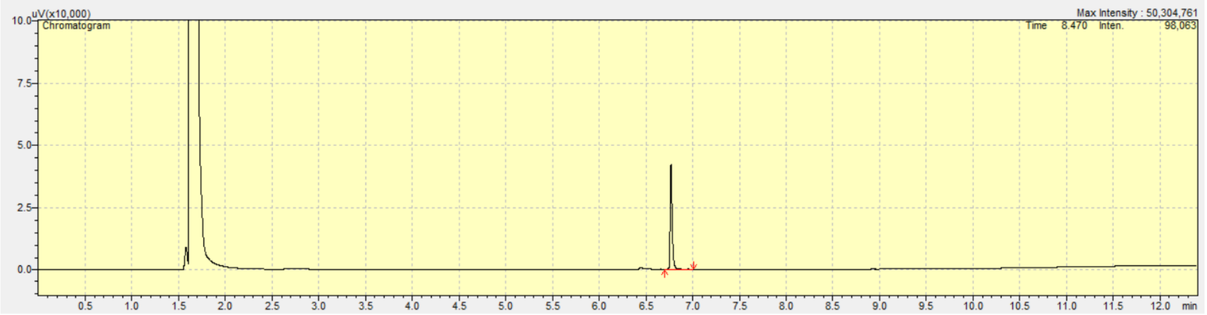

2-methylisoquinoline-1,3,4(2H)-trione (21b)

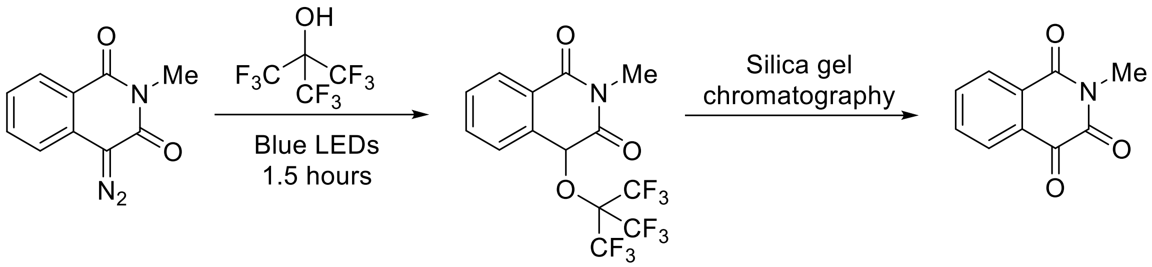

4-Diazo-2-methylisoquinoline-1,3(2*H*,4*H*)-dione (40 mg, 0.20 mmol) was charged in a vial and dissolved in nonafluoro-*tert*-butanol (2 ml) and irradiated with blue LEDs for 1.5 hours. After this time, the solution was transferred to a flask with DCM and all the volatiles are removed in vacuo. ^{1}H and ^{13}C**{^{1}H}** NMR of the crude material showed evidence for the formation of 4-((1,1,1,3,3,3-hexafluoro-2-(trifluoromethyl)propan-2-yl)oxy)-2-methylisoquinoline-1,3(2*H*,4*H*) -dione. During silica column chromatography (10% EtOAc in hexane) the compound rearranged to the title compound in 62% yield (24 mg).

^{1}H NMR (400 MHz, $CDCl_3$) δ 8.34 (dd, *J* = 7.7, 1.4 Hz, 1H), 8.21 (dd, *J* = 7.7, 1.4 Hz, 1H), 7.90 (td, *J* = 7.6, 1.4 Hz, 1H), 7.83 (td, *J* = 7.5, 1.4 Hz, 1H), 3.47 (s, 3H).

^{13}C{^{1}H} NMR (101 MHz, $CDCl_3$) δ 174.6, 162.5, 157.4, 136.2, 134.6, 130.8, 130.0, 129.9, 127.9, 27.7.

The spectroscopic data is consistent with that previously reported in the literature.[21]

11. S-H Insertion Products

2-methyl-4-(phenethylthio)isoquinoline-1,3(2*H*,4*H*)-dione (22)

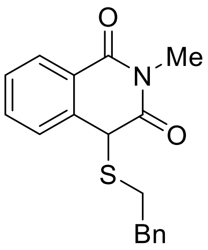

The title compound was synthesised according to General Procedure E from 4-diazo-2-methylisoquinoline-1,3(2*H*,4*H*)-dione (40 mg, 0.20 mmol) and 2-phenylethanethiol (2 mL) in 73% yield (45 mg) as a white solid. Eluent for column chromatography (SiO_2): 10% EtOAc in hexane.

^{1}H NMR (500 MHz, $CDCl_3$) δ 8.17 (d, *J* = 7.5 Hz, 1H), 7.60 (td, *J* = 7.5, 1.4 Hz, 1H), 7.46 (t, *J* = 7.7 Hz, 1H), 7.43 (d, *J* = 7.6 Hz, 1H), 7.31 (t, *J* = 7.6 Hz, 2H), 7.25 – 7.22 (m, 3H), 4.62 (s, 1H), 3.37 (s, 3H), 3.20 (ddd, *J* = 12.2, 8.9, 6.1 Hz, 1H), 3.00 – 2.84 (m, 3H).

^{13}C{^{1}H} NMR (126 MHz, $CDCl_3$) δ 171.0, 164.4, 139.8, 135.1, 133.9, 129.1, 128.83, 128.82, 128.71, 128.70, 126.8, 125.7, 44.9, 35.8, 33.8, 27.4.

HRMS (ESI$^-$) *m/z* calc'd for $C_{18}H_{16}NO_2S^-$: 310.0902; found: 310.0905 $[M\text{-}H^+]^-$

m.p. 75 – 77 °C

EA calc'd (%) for $C_{18}H_{17}NO_2S$: C – 69.43, H – 5.50, N – 4.50, S – 10.30; found: C – 69.24, H – 5.49, N – 4.39, S – 10.36

4-(cyclohexylthio)-2-methylisoquinoline-1,3(2*H*,4*H*)-dione (23)

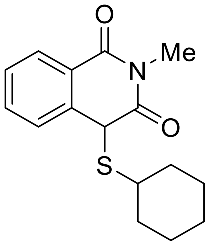

The title compound was synthesised according to General Procedure E from 4-diazo-2-methylisoquinoline-1,3(2*H*,4*H*)-dione (40 mg, 0.20 mmol) and cyclohexanethiol (2 mL) in 70% yield (41 mg) as a beige solid. Eluent for column chromatography (SiO_2): 10% EtOAc in hexane.

1H NMR (500 MHz, $CDCl_3$) δ 8.15 (dd, *J* = 7.9, 1.4 Hz, 1H), 7.59 (td, *J* = 7.5, 1.4 Hz, 1H), 7.46 – 7.42 (m, 2H), 4.76 (s, 1H), 3.36 (s, 3H), 3.15 (tt, *J* = 10.5, 3.7 Hz, 1H), 2.33 – 2.28 (m, 1H), 1.84 – 1.69 (m, 2H), 1.65 – 1.60 (m, 1H), 1.53 – 1.22 (m, 6H).

$^{13}C\{^1H\}$ NMR (126 MHz, $CDCl_3$) δ 171.5, 164.4, 135.6, 133.8, 129.0, 128.7, 128.6, 125.7, 44.6, 43.5, 33.8, 32.4, 27.3, 26.1, 25.9, 25.8.

HRMS (ESI^-) *m/z* calc'd for $C_{16}H_{18}NO_2S^-$: 288.1058; found: 288.1059 $[M-H^+]^-$

m.p. 107 - 111 °C

GC (FID)

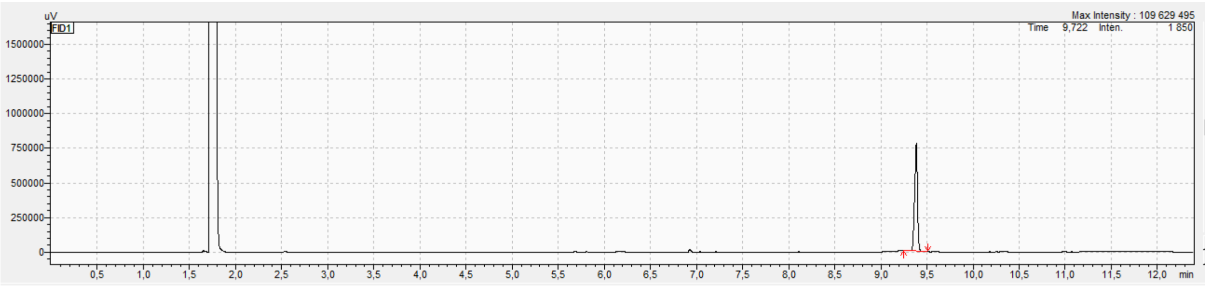

4-(benzylthio)-2-methylisoquinoline-1,3(2*H*,4*H*)-dione (24)

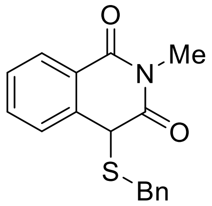

The title compound was synthesised according to General Procedure E from 4-diazo-2-methylisoquinoline-1,3(2*H*,4*H*)-dione (40 mg, 0.20 mmol) and benzyl mercaptan (2 mL) in 66% yield (39 mg) as a colourless oil. Eluent for column chromatography (SiO_2): 10% EtOAc in hexane.

^{1}H NMR (500 MHz, $CDCl_3$) δ 8.14 (dd, *J* = 7.9, 1.4 Hz, 1H), 7.55 (td, *J* = 7.6, 1.4 Hz, 1H), 7.45 – 7.42 (m, 3H), 7.37 – 7.34 (m, 2H), 7.30 – 7.25 (m, 2H), 4.48 (s, 1H), 4.08 (d, *J* = 13.7 Hz, 1H), 3.81 (d, *J* = 13.7 Hz, 1H), 3.31 (s, 3H).

^{13}C{^{1}H} NMR (126 MHz, $CDCl_3$) δ 171.1, 164.3, 136.9, 135.0, 133.9, 129.5, 128.9, 128.83, 128.79, 128.69, 127.7, 125.8, 43.5, 36.9, 27.2.

HRMS (ESI$^+$) *m/z* calc'd for $C_{17}H_{15}NO_2SNa^+$: 320.0721; found: 320.0723 $[M+Na]^+$

EA: calc'd (%) for $C_{17}H_{15}NO_2S$: C – 68.66, H – 5.08, N – 4.71, S – 10.78; found: C – 68.49, H – 5.14, N 4.94, S – 10.58

4-(dodecylthio)-2-methylisoquinoline-1,3(2*H*,4*H*)-dione (25)

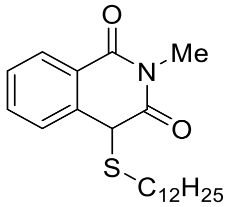

The title compound was synthesised according to General Procedure E from 4-diazo-2-methylisoquinoline-1,3(2*H*,4*H*)-dione (40 mg, 0.20 mmol) and 1-dodecanethiol (2 mL) in 50% yield (37 mg) as a white solid. Eluent for column chromatography (SiO_2): 10% EtOAc in hexane.

^{1}H NMR (500 MHz, $CDCl_3$) δ 8.17 (dd, *J* = 8.0, 1.4 Hz, 1H), 7.62 (td, *J* = 7.6, 1.4 Hz, 1H), 7.51 – 7.45 (m, 2H), 4.67 (s, 1H), 3.38 (s, 3H), 2.86 (ddd, *J* = 12.6, 8.4, 5.8 Hz, 1H), 2.57 (ddd, *J* = 12.6, 8.6, 6.7 Hz, 1H), 1.67 – 1.54 (m, 3H), 1.30 – 1.25 (m, 16H), 0.88 (t, *J* = 6.9 Hz, 3H).

^{13}C{^{1}H} NMR (126 MHz, $CDCl_3$) δ 171.2, 164.4, 135.5, 133.9, 129.0, 128.7, 128.7, 125.7, 45.1, 32.5, 32.1, 29.8, 29.8, 29.7, 29.6, 29.5, 29.3, 29.0, 28.9, 27.4, 22.8, 14.3.

HRMS (ESI$^-$) *m/z* calc'd for $C_{22}H_{32}NO_2S^-$: 374.2154; found: 374.2155 $[M-H^+]^-$

m.p. 60 – 62 °C

EA calc'd (%) for $C_{22}H_{33}NO_2S$: C – 70.36, H – 8.86, N – 3.73, S – 8.54; found: C – 70.30, H – 8.90, N – 3.83, S – 8.31

4-((2-hydroxyethyl)thio)-2-methylisoquinoline-1,3(2*H*,4*H*)-dione (26)

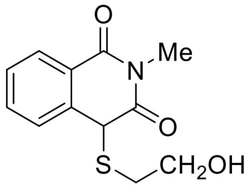

The title compound was synthesised according to General Procedure E from 4-diazo-2-methylisoquinoline-1,3(2*H*,4*H*)-dione (40 mg, 0.20 mmol) and 2-mercaptoethanol (2 mL) in 45% yield (23 mg) as a colourless oil. Eluent for column chromatography (SiO_2): 20% -> 40% EtOAc in hexane.

^{1}H NMR (500 MHz, $CDCl_3$) δ 8.19 – 8.17 (m, 1H), 7.63 (td, *J* = 7.5, 1.4 Hz, 1H), 7.55 – 7.53 (m, 1H), 7.50 – 7.47 (m, 1H), 4.90 (s, 1H), 3.94 – 3.84 (m, 2H), 3.38 (s, 3H), 3.14 (ddd, *J* = 14.3, 6.6, 4.5 Hz, 1H), 2.84 (ddd, *J* = 14.3, 6.7, 4.7 Hz, 1H), 2.47 (s, 1H).

^{13}C{^{1}H} NMR (126 MHz, $CDCl_3$) δ 171.6, 164.2, 135.2, 134.1, 129.2, 129.0, 128.8, 125.5, 62.0, 44.8, 35.9, 27.5.

HRMS (ESI$^+$) *m/z* calc'd for $C_{12}H_{13}NO_3SNa^+$: 274.0514; found: 274.0516 $[M+Na]^+$

EA calc'd (%) for $C_{12}H_{13}NO_3S$: C – 57.35, H – 5.21, N – 5.57, S – 12.76; found: C – 57.25, H – 5.38, N – 5.66, S – 13.03

12. Arene Insertions

2-methyl-4-phenylisoquinoline-1,3(2*H*,4*H*)-dione (28)

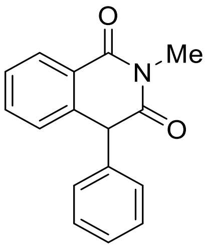

The title compound was synthesised according to General Procedure F from 4-diazo-2-methylisoquinoline-1,3(2*H*,4*H*)-dione (40 mg, 0.20 mmol) and benzene (2 mL) in 37% yield (19 mg) as a white solid. Eluent for column chromatography (SiO_2): 10% EtOAc in hexane.

^{1}H NMR (600 MHz, $CDCl_3$) δ 8.29 (dd, *J* = 7.9, 1.4 Hz, 1H), 7.55 (td, *J* = 7.5, 1.5 Hz, 1H), 7.49 – 7.46 (m, 1H), 7.32 – 7.26 (m, 3H), 7.14 – 7.11 (m, 3H), 5.09 (s, 1H), 3.35 (s, 3H).

^{13}C{^{1}H} NMR (151 MHz, $CDCl_3$) δ 172.1, 165.1, 139.2, 138.4, 134.0, 129.2, 128.9, 128.7, 128.5, 128.1, 128.1, 125.5, 52.6, 27.4.

The spectroscopic data is consistent with that previously reported in the literature.[22]

4-(2,5-dimethylphenyl)-2-methylisoquinoline-1,3(2*H*,4*H*)-dione (29)

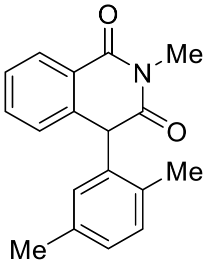

The title compound was synthesised according to General Procedure F from 4-diazo-2-methylisoquinoline-1,3(2*H*,4*H*)-dione (40 mg, 0.20 mmol) and *p*-xylene (2 mL) in 43% yield (24 mg) as a white solid. Eluent for column chromatography (SiO_2): 10% EtOAc in hexane.

^{1}H NMR (500 MHz, $CDCl_3$) δ 8.29 (dd, *J* = 7.8, 1.5 Hz, 1H), 7.49 (td, *J* = 7.5, 1.6 Hz, 1H), 7.46 – 7.42 (m, 1H), 7.11 (d, *J* = 7.7 Hz, 1H), 7.02 (d, *J* = 7.7 Hz, 1H), 6.97 (d, *J* = 7.9 Hz, 1H), 6.71 (s, 1H), 5.24 (s, 1H), 3.40 (s, 3H), 2.23 (s, 6H).

^{13}C{^{1}H} NMR (151 MHz, $CDCl_3$) δ 172.2, 165.1, 139.1, 137.4, 136.2, 134.0, 133.5, 131.4, 130.7, 129.0, 128.8, 127.9, 127.8, 125.3, 49.9, 27.3, 21.0, 19.6.

The spectroscopic data is consistent with that previously reported in the literature.[22]

4-mesityl-2-methylisoquinoline-1,3(*2H,4H*)-dione (30)

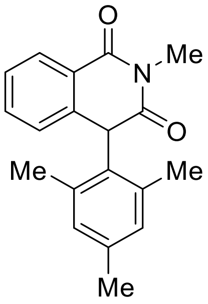

The title compound was synthesised according to General Procedure F from 4-diazo-2-methylisoquinoline-1,3(2*H*,4*H*)-dione (40 mg, 0.20 mmol) and mesitylene (2 mL) in 50% yield (30 mg) as a white solid. Eluent for column chromatography (SiO_2): 10% EtOAc in hexane.

^{1}H NMR (500 MHz, $CDCl_3$) δ 8.29 (dd, *J* = 7.6, 1.7 Hz, 1H), 7.48 – 7.40 (m, 2H), 7.01 (s, 1H), 6.83 (dt, *J* = 7.6, 1.5 Hz, 1H), 6.80 (s, 1H), 5.44 (s, 1H), 3.45 (s, 3H), 2.49 (s, 3H), 2.29 (s, 3H), 1.62 (s, 3H).

^{13}C{^{1}H} NMR (126 MHz, $CDCl_3$) δ 172.2, 165.0, 138.9, 137.8, 137.7, 136.3, 134.1, 133.0, 130.8, 129.2, 129.0, 127.6, 126.5, 125.3, 47.1, 27.2, 21.15, 21.08, 20.0.

HRMS (ESI$^+$) m/z calc'd for $C_{19}H_{19}NO_2Na^+$: 316.1313; found: 316.1316 $[M+Na]^+$

m.p. 148 – 151 °C

GC (FID):

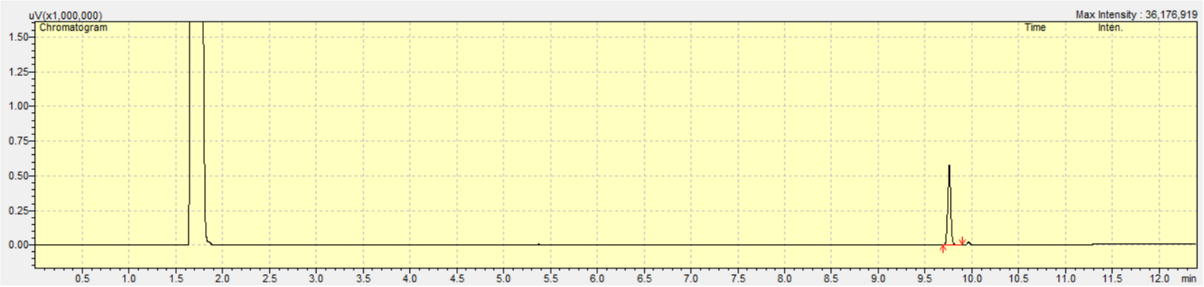

4-(5-bromo-2-methoxyphenyl)-2-methylisoquinoline-1,3(2H,4H)-dione (31)

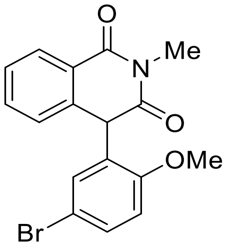

The title compounds were synthesised according to General Procedure F from 4-diazo-2-methylisoquinoline-1,3(2*H*,4*H*)-dione (40 mg, 0.20 mmol) and 4-bromoanisole (2 mL) in 20% yield (14 mg) as an off-white solid. Eluent for column chromatography (SiO_2): gradually from 20% of EtOAc in hexane to 35%.

^{1}H NMR (500 MHz, $CDCl_3$) δ 8.25 (dd, *J* = 7.8, 1.5 Hz, 1H), 7.48 (td, *J* = 7.5, 1.5 Hz, 1H), 7.43 – 7.38 (m, 3H), 7.03 (d, *J* = 7.7 Hz, 1H), 6.73 (d, *J* = 8.6 Hz, 1H), 5.07 (s, 1H), 3.58 (s, 3H), 3.42 (s, 3H).

^{13}C{^{1}H} NMR (126 MHz, $CDCl_3$) δ 171.8, 165.2, 156.0, 138.1, 134.0, 133.7, 132.3, 130.4, 128.7, 127.7, 127.3, 125.2, 113.6, 113.3, 56.1, 48.3, 27.2.

HRMS (ESI$^+$) *m/z* calc'd for $C_{17}H_{14}NO_3NaBr^+$: 382.0055; found: 382.0056 $[M+Na]^+$

m.p. 170 – 173 °C

GC (FID):

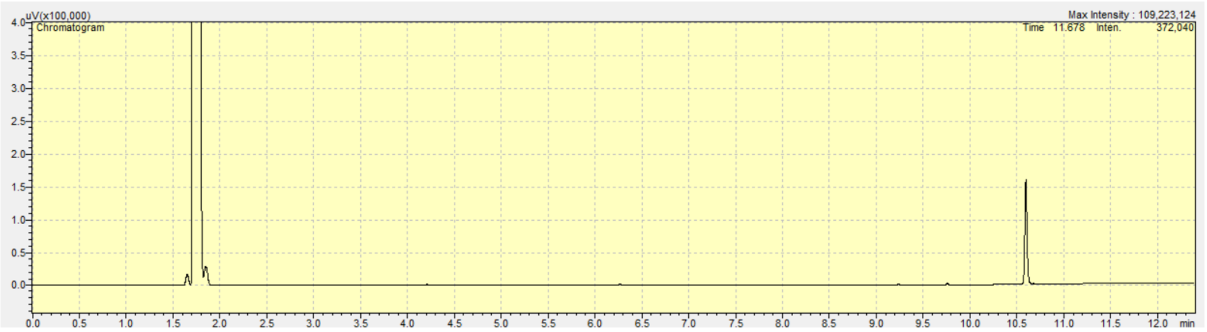

4-(2,4-dimethoxyphenyl)-2-methylisoquinoline-1,3(2*H*,4*H*)-dione (32) and

4-(2,6-dimethoxyphenyl)-2-methylisoquinoline-1,3(2*H*,4*H*)-dione (33)

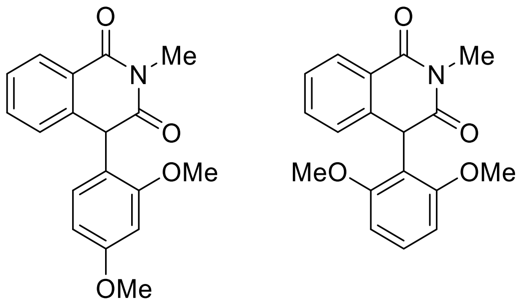

The title compounds were synthesised according to General Procedure F from 4-diazo-2-methylisoquinoline-1,3(2*H*,4*H*)-dione (40 mg, 0.20 mmol) and 1,3-dimethoxybenzene (2 mL) in 74% yield (46 mg) as a white foam. The product was isolated as an inseparable 2:1 (**32**:**33**) mixture of regioisomers. Eluent for column chromatography (SiO_2): gradually from 30% of EtOAc in hexane to 40%.

^{1}H NMR (500 MHz, $CDCl_3$) δ 8.36 (dd, *J* = 7.9, 1.4 Hz, 1H), 8.22 (td, *J* = 7.5, 1.5 Hz, 2H + 33), 7.90 (td, *J* = 7.6, 1.4 Hz, 1H), 7.83 (td, *J* = 7.5, 1.3 Hz, 1H), 7.47 – 7.32 (m, 4H + 33), 7.27 – 7.22 (m, 1H + 33), 7.14 (d, *J* = 8.2 Hz, 1H), 7.05 (d, *J* = 7.7 Hz, 1H), 7.02 (d, *J* = 7.7 Hz, 1H), 6.50 (dd, *J* = 8.3, 2.4 Hz, 1H), 6.42 (d, *J* = 2.4 Hz, 1H), 5.67 (s, 1H), 5.06 (s, 1H), 3.96 (s, 3H), 3.79 (s, 3H), 3.58 (s, 3H), 3.49 (s, 3H), 3.43 (s, 3H), 3.41 (s, 3H).

^{13}C{^{1}H} NMR (126 MHz, $CDCl_3$) Unable to unambiguously assign the ^{13}C peaks due to the low rr.

HRMS (ESI$^+$) *m/z* calc'd for $C_{18}H_{17}NO_4Na^+$: 334.1055; found: 334.1052 [M+Na]$^+$

GC (FID): 2:1 mixture.

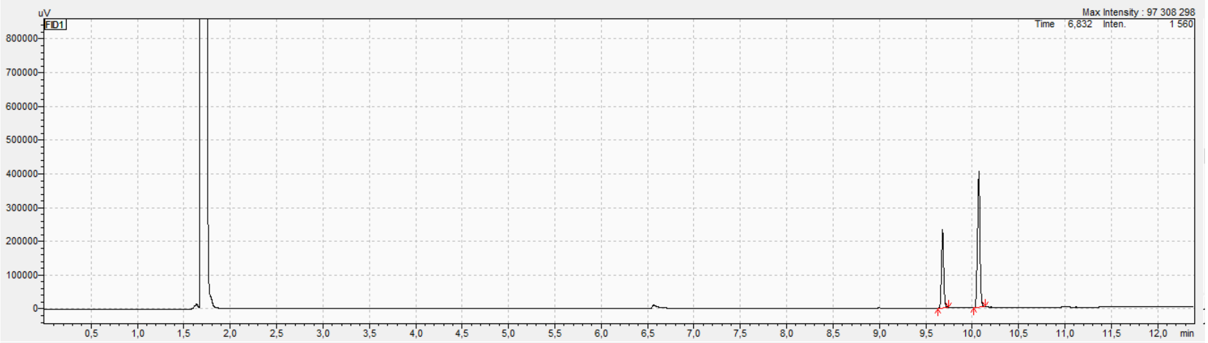

13. Reactions of 4-diazo-2*H*-benzo[*e*][1,2]thiazin-3(4*H*)-one 1,1-dioxides

4-((1,1,1,3,3,3-hexafluoropropan-2-yl)oxy)-2-methyl-2H-benzo[*e*][1,2]thiazin-3(4*H*)-one 1,1-dioxide (35)

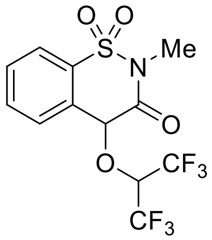

The title compound was synthesised according to General Procedure G from 4-diazo-2-methyl-2*H*-benzo[*e*][1,2]thiazin-3(4*H*)-one 1,1-dioxide (36 mg, 0.15 mmol) and HFIP (1.5 mL) in 53% yield (30 mg) as a white solid. Performing the reaction on the same scale with blue LEDs for 2.5 hours yielded the title compound in 52% yield (29 mg). Eluent for column chromatography (SiO_2): 10% EtOAc in hexane.

^{1}H NMR (500 MHz, $CDCl_3$) δ 7.94 (d, *J* = 7.7 Hz, 1H), 7.84 (dt, *J* = 7.8, 1.3 Hz, 1H), 7.75 (td, *J* = 7.7, 1.3 Hz, 1H), 7.61 (t, *J* = 7.7 Hz, 1H), 5.92 (s, 1H), 5.02 (hept, *J* = 5.6 Hz, 1H), 3.32 (s, 3H).

^{13}C{^{1}H} NMR (126 MHz, $CDCl_3$) δ 167.6, 134.2, 134.1, 131.4, 129.3, 125.5, 123.2, 123.0 – 118.3 (m), 76.1, 75.5 (obs. p, *J* = 33.6 Hz), 27.8.

^{19}F NMR (470 MHz, $CDCl_3$) δ -72.6 – -72.6 (m, 3F), -72.7 – -72.8 (m, 3F).

HRMS (ESI$^-$) *m/z* calc'd for $C_{12}H_8NO_4F_6S^-$: 376.0078; found: 376.0080 $[M-H^+]^-$

m.p. 92 – 96 °C

GC (FID):

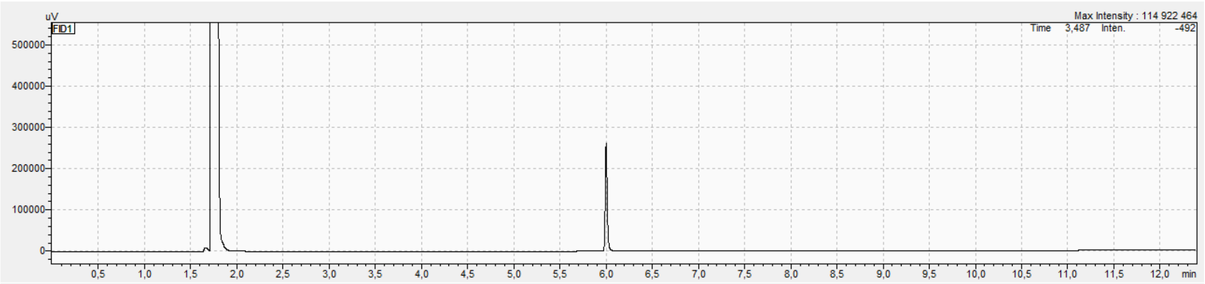

2-benzyl-4-((1,1,1,3,3,3-hexafluoropropan-2-yl)oxy)-2*H*-benzo[*e*][1,2]thiazin-3(4*H*)-one 1,1-dioxide (36)

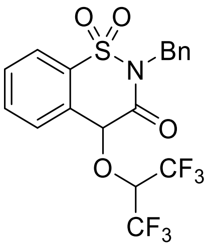

The title compound was synthesised according to General Procedure G from 2-benzyl-4-diazo-2*H*-benzo[e][1,2]thiazin-3(4*H*)-one 1,1-dioxide (47 mg, 0.15 mmol) and HFIP (1.5 mL) in 51% yield (35 mg) as a white solid. Eluent for column chromatography (SiO_2): 10% EtOAc in hexane.

^{1}H NMR (500 MHz, $CDCl_3$) δ 7.95 (d, *J* = 7.7 Hz, 1H), 7.84 (d, *J* = 7.8 Hz, 1H), 7.74 (td, *J* = 7.7, 1.3 Hz, 1H), 7.61 (t, *J* = 7.7 Hz, 1H), 7.44 – 7.42 (m, 2H), 7.36 – 7.28 (m, 3H), 5.97 (s, 1H), 5.04 (d, *J* = 15.2 Hz, 1H), 5.00 – 4.95 (m, 2H).

^{13}C{^{1}H} NMR (126 MHz, $CDCl_3$) δ 167.4, 135.3, 134.5, 134.2, 131.6, 129.3, 128.9, 128.8, 128.4, 125.5, 123.0, 122.8 – 118.5 (m), 76.3, 75.5 (obs. p, *J* = 33.0 Hz), 46.3.

^{19}F NMR (470 MHz, $CDCl_3$) δ -72.5 – -72.6 (m, 3F), -72.6 – -72.7 (m, 3F).

HRMS (ESI$^-$) *m/z* calc'd for $C_{18}H_{12}NO_4SF_6^-$:452.0391; found: 452.0399 $[M-H^+]^-$

m.p. 140 – 143 °C

GC (FID):

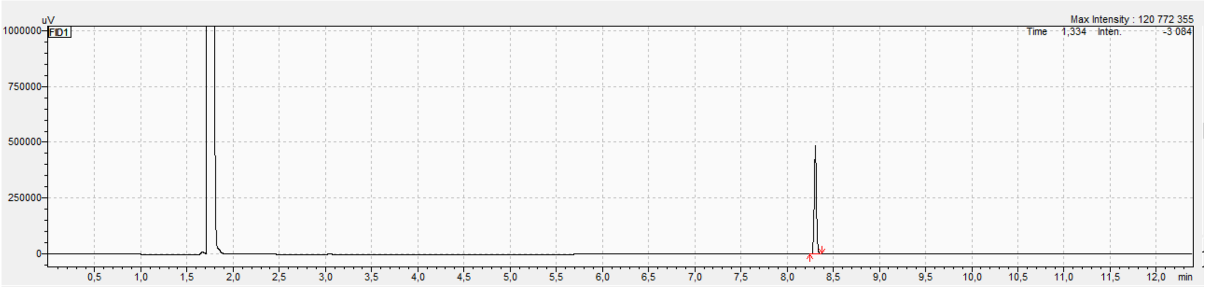

2-allyl-4-((1,1,1,3,3,3-hexafluoropropan-2-yl)oxy)-2*H*-benzo[*e*][1,2]thiazin-3(4*H*)-one 1,1-dioxide (37)

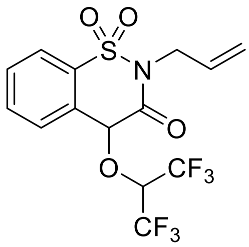

The title compound was synthesised according to General Procedure G from 2-allyl-4-diazo-2*H*-benzo[*e*][1,2]thiazin-3(4*H*)-one 1,1-dioxide (39 mg, 0.15 mmol) and HFIP (1.5 mL) in 44% yield (27 mg) as a white solid. Eluent for column chromatography (SiO_2): 10% EtOAc in hexane.

^{1}H NMR (500 MHz, $CDCl_3$) δ 7.93 (dd, *J* = 7.7, 0.7 Hz, 1H), 7.84 (d, *J* = 7.8 Hz, 1H), 7.74 (td, *J* = 7.7, 1.3 Hz, 1H), 7.61 (t, *J* = 7.7 Hz, 1H), 5.97 (s, 1H), 5.87 (ddt, *J* = 17.1, 10.2, 5.8 Hz, 1H), 5.33 (dq, *J* = 17.0, 1.3 Hz, 1H), 5.25 (dq, *J* = 10.3, 1.1 Hz, 1H), 5.00 (hept, *J* = 5.5 Hz, 1H), 4.51 (ddt, *J* = 15.8, 5.8, 1.4 Hz, 1H), 4.41 (ddt, *J* = 15.7, 6.0, 1.4 Hz, 1H).

^{13}C{^{1}H} NMR (126 MHz, $CDCl_3$) δ 167.2, 134.5, 134.2, 131.5, 130.9, 129.3, 125.5, 123.0, 119.6, 76.3, 75.5 (obs. p, *J* = 32.2 Hz), 44.9. (One carbon is missing due to ^{19}F splitting).

^{19}F NMR (470 MHz, $CDCl_3$) δ -72.5 – -72.6 (m, 3F), -72.6 – -72.7 (m, 3F).

HRMS (APCI$^+$) *m/z* calc'd for $C_{14}H_{12}NO_4F_6S^+$: 404.0391; found: 404.0390. $[M+H]^+$

m.p. 65 – 67 °C

GC (FID):

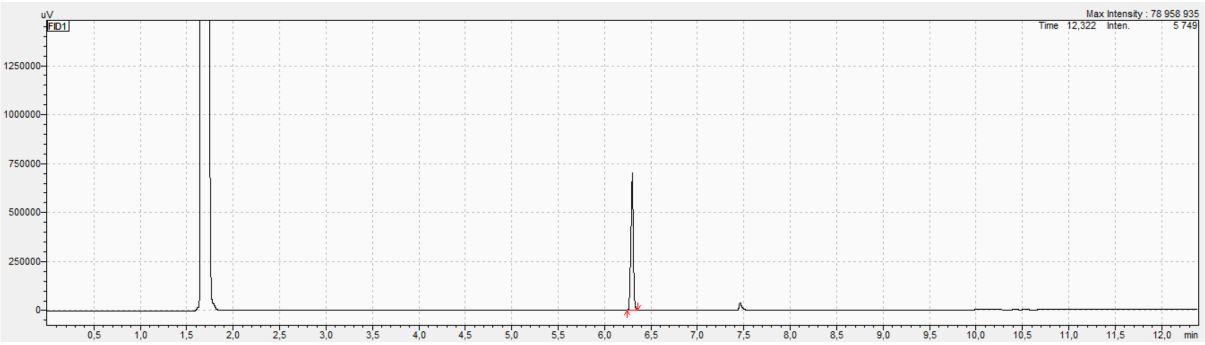

2-methyl-4-(2,2,3,3-tetrafluoropropoxy)-2H-benzo[*e*][1,2]thiazin-3(4*H*)-one 1,1-dioxide (38)

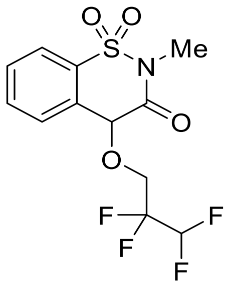

The title compound was synthesised according to General Procedure G from 4-diazo-2-methyl-2*H*-benzo[*e*][1,2]thiazin-3(4*H*)-one 1,1-dioxide (36 mg, 0.15 mmol) and 2,2,3,3-tetrafluoropropan-1-ol (1.5 mL) with an extended reaction time of 4 hours in 46% yield (24 mg) as a colourless oil. Eluent for column chromatography (SiO_2): 10% EtOAc in hexane.

^{1}H NMR (500 MHz, $CDCl_3$) δ 7.91 (d, *J* = 7.7 Hz, 1H), 7.79 (d, *J* = 7.8 Hz, 1H), 7.71 (td, *J* = 7.7, 1.2 Hz, 1H), 7.57 (t, *J* = 7.7 Hz, 1H), 6.09 (tdd, *J* = 53.2, 5.2, 3.2 Hz, 1H), 5.64 (s, 1H), 4.72 – 4.64 (m, 1H), 4.15 (qd, *J* = 11.8, 2.3 Hz, 1H), 3.30 (s, 3H).

^{13}C{^{1}H} NMR (126 MHz, $CDCl_3$) δ 168.7, 134.2, 134.0, 132.7, 128.9, 125.3, 123.0, 114.8 (tt, *J* = 249.9, 27.7 Hz), 109.5 (tdd, *J* = 250.0, 36.8, 34.8 Hz), 68.9 (dd, *J* = 29.7, 27.1 Hz), 27.9. (One peak is missing and overlaps with the $CDCl_3$ peak).

^{19}F NMR (470 MHz, $CDCl_3$) δ -123.5 – -125.6 (m, 2F), -136.7 – -139.3 (m, 2F).

HRMS (APCI$^+$) *m/z* calc'd for $C_{12}H_{12}NO_4F_4S^+$: 342.0423; found: 342.0425 $[M+H]^+$

GC (FID):

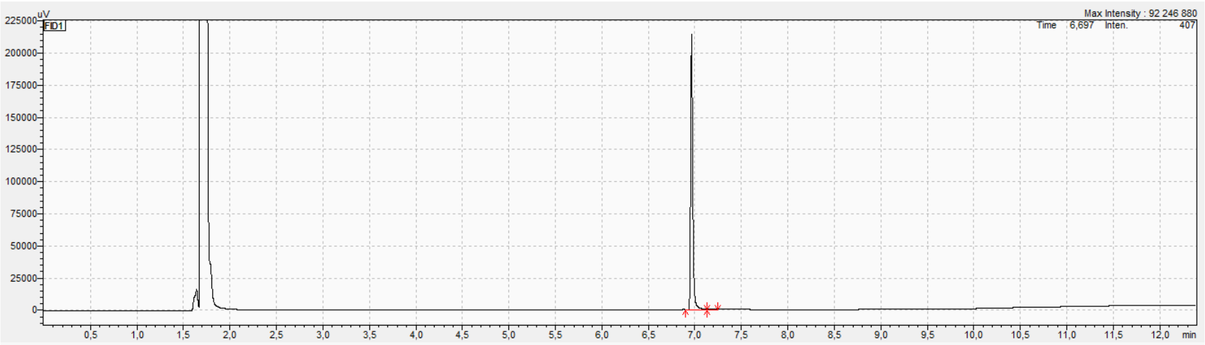

4-(cyclohexylthio)-2-methyl-2H-benzo[*e*][1,2]thiazin-3(4*H*)-one 1,1-dioxide (39)

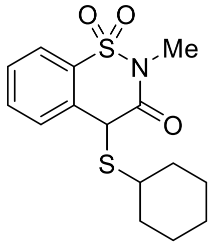

The title compound was synthesised according to General Procedure G from 4-diazo-2-methyl-2*H*-benzo[*e*][1,2]thiazin-3(4*H*)-one 1,1-dioxide (36 mg, 0.15 mmol) and cyclohexanethiol (2 mL) with an extended reaction time of 7 hours in 50% yield (24 mg) as a white solid. Eluent for column chromatography (SiO_2): 10% EtOAc in hexane.

^{1}H NMR (500 MHz, $CDCl_3$) δ 7.95 (dd, *J* = 7.9, 1.3 Hz, 1H), 7.65 (td, *J* = 7.6, 1.3 Hz, 1H), 7.54 – 7.50 (m, 2H), 4.85 (s, 1H), 3.35 (s, 3H), 3.17 – 3.11 (m, 1H), 2.26 (d, *J* = 12.6 Hz, 1H), 2.02 – 1.98 (m, 1H), 1.85 – 1.76 (m, 2H), 1.66 – 1.62 (m, 1H), 1.53 – 1.24f (m, 5H).

^{13}C{^{1}H} NMR (126 MHz, $CDCl_3$) δ 167.9, 135.1, 134.1, 132.8, 129.5, 128.8, 123.8, 45.9, 45.8, 33.6, 32.8, 27.1, 26.1, 25.9, 25.8.

HRMS (APCI$^-$) *m/z* calc'd for $C_{15}H_{18}NO_3S_2^-$: 324.0728; found: 324.0730 $[M-H]^+$

m.p. 122 – 124 °C

GC (FID):

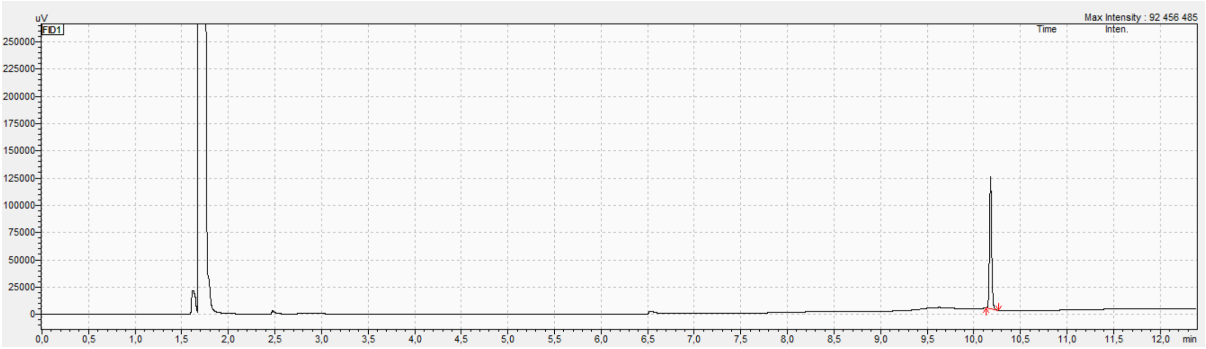

14. References

(1) Tsou, H. R.; Otteng, M.; Tran, T.; Floyd, M. B.; Reich, M.; Birnberg, G.; Kutterer, K.; Ayral-Kaloustian, S.; Ravi, M.; Nilakantan, R.; Grillo, M.; McGinnis, J. P.; Rabindran, S. K. 4-(Phenylaminomethylene)Isoquinoline-1,3(2H,4H)-Diones as Potent and Selective Inhibitors of the Cyclin-Dependent Kinase 4 (CDK4). *J. Med. Chem.* **2008**, *51* (12), 3507–3525.

(2) Liu, Z.; Shibata, N.; Takeuchi, Y. Novel Methods for the Facile Construction of 3,3-Disubstituted and 3,3-Spiro-2H,4H-Benzo[e]1,2-Thiazine-1,1-Diones: Synthesis of (11S,12R,14R)-2-Fluoro-14-Methyl-11-(Methylethyl)Spiro[4H]Benzo[e]-1,2-Thiazine-3,2'-Cyclohexane]-1,1-Dione, an Agent for The . *J. Org. Chem.* **2000**, *65* (22), 7583–7587.

(3) Barbazanges, M.; Augé, M.; Moussa, J.; Amouri, H.; Aubert, C.; Desmarets, C.; Fensterbank, L.; Gandon, V.; Malacria, M.; Ollivier, C. Enantioselective IrI-Catalyzed Carbocyclization of 1,6-Enynes by the Chiral Counterion Strategy. *Chem. - A Eur. J.* **2011**, *17* (49), 13789–13794.

(4) Lombardino, J. G.; Wiseman, E. H. Antiinflammatory 3,4-Dihydro-2-Alkyl-3-Oxo-2H-1,2-Benzothiazine-4-Carboxamide 1,1-Dioxides. *J. Med. Chem.* **1971**, *14* (10), 973–977.

(5) Kantin, G.; Dar'in, D.; Krasavin, M. RhII-Catalyzed Cycloaddition of α-Diazo Homophthalimides and Nitriles Delivers Oxazolo[5,4-c]Isoquinolin-5(4H)-One Scaffold. *Eur. J. Org. Chem.* **2018**, *2018* (35), 4857–4859.

(6) Eom, S.; Kwon, T.; Lee, D. Y.; Park, C. H.; Kim, H. J. Copper-Mediated Three-Component Reaction for the Synthesis of N-Acylsulfonamide on DNA. *Org. Lett.* **2022**, *24* (27), 4881–4885.

(7) Lobb, K. L.; Hipskind, P. A.; Aikins, J. A.; Alvarez, E.; Cheung, Y. Y.; Considine, E. L.; De Dios, A.; Durst, G. L.; Ferritto, R.; Grossman, C. S.; Giera, D. D.; Hollister, B. A.; Huang, Z.; Iversen, P. W.; Law, K. L.; Li, T.; Lin, H. S.; Lopez, B.; Lopez, J. E.; Martin Cabrejas, L. M.; McCann, D. J.; Molero, V.; Reilly, J. E.; Richett, M. E.; Shih, C.; Teicher, B.; Wikel, J. H.; White, W. T.; Mader, M. M. Acyl Sulfonamide Anti-Proliferatives: Benzene Substituent Structure-Activity Relationships for a Novel Class of Antitumor Agents. *J. Med. Chem.* **2004**, *47* (22), 5367–5380.

(8) Venkateswarlu, V.; Aravinda Kumar, K. A.; Gupta, S.; Singh, D.; Vishwakarma, R. A.; Sawant, S. D. DMSO/I_2 Mediated C-C Bond Cleavage of α-Ketoaldehydes Followed by C-O Bond Formation: A Metal-Free Approach for One-Pot Esterification. *Org. Biomol. Chem.* **2015**, *13* (29), 7973–7978.

(9) Deady, L. W.; Rodemann, T. Reduced Benzimidazo[2,1-a]Isoquinolines. Synthesis and Cytotoxicity Studies. *Aust. J. Chem.* **2001**, *54* (8), 529.

(10) Dey, A. S.; Rosowsky, A.; Modest, E. J. Direction of Cyclization of l,2-Bis-(Cyanomethyl)Benzenes. *J. Org. Chem.* **1970**, *35* (2), 536–539.

(11) Billamboz, M.; Bailly, F.; Barreca, M. L.; De Luca, L.; Mouscadet, J. F.; Calmels, C.; Andréola, M. L.; Witvrouw, M.; Christ, F.; Debyser, Z.; Cotelle, P. Design, Synthesis, and Biological Evaluation of a Series of 2-Hydroxyisoquinoline-1,3(2H,4H)-Diones as Dual Inhibitors of Human Immunodeficiency Virus Type 1 Integrase and the Reverse Transcriptase RNase H Domain. *J. Med. Chem.* **2008**, *51* (24), 7717–7730.

(12) Glennon, R. A.; Raghupathi, R.; Bartyzel, P.; Teitler, M.; Leonhardt, S. Binding of Phenylalkylamine Derivatives at 5-HT1C and 5-HT2 Serotonin Receptors: Evidence for a

Lack of Selectivity. *J. Med. Chem.* **1992**, *35* (4), 734–740.

(13) Trost, B. M.; Kalnmals, C. A. Sulfones as Synthetic Linchpins: Transition-Metal-Free Sp3–Sp2 and Sp2–Sp2 Cross-Couplings Between Geminal Bis(Sulfones) and Organolithium Compounds. *Chem. - A Eur. J.* **2018**, *24* (36), 9066–9074.

(14) Jangir Narshinha P., R. A. Total Synthesis of Tetrahydroisoquinoline-Based Bioactive Natural Products Laudanosine, Romneine, Glaucine, Dicentrine, and Their Unnatural Analogues Isolaudanosine and Isoromneine. *Synthesis* **2017**, *49* (07), 1655–1663.

(15) Horning, D. E.; Lacasse, G.; Muchowski, J. M. Spiroalkylation of Some Homophthalimides and Oxindoles with 1-Bromo-2-Chloroethane. *Can. J. Chem.* **1971**, *49* (2), 246–254.

(16) Deliömeroğlu, M. K.; Özcan, S.; Balcia, M. A Short and Efficient Construction of the Dibenzo[c,h]Chromen-6-One Skeleton. *Arkivoc* **2010**, *2010* (2), 148–160.

(17) Jaiswal, A. K.; Kushawaha, A. K.; Pandey, S.; Kumar, A.; Sashidhara, K. V. Halogen-Free Oxidation of Aryl Ketones and Benzyl Nitrile Derivatives to Corresponding Carboxylic Acids by Using NaOH/ TBHP in Aqueous Medium. *Tetrahedron* **2023**, *136*, 133359.

(18) Yang, Y.; Li, Y.; Cheng, C.; Yang, G.; Zhang, J.; Zhang, Y.; Zhao, Y.; Zhang, L.; Li, C.; Tang, L. Synthesis of 4-Aryl Isoquinolinedione Derivatives by a Palladium-Catalyzed Coupling Reaction of Aryl Halides with Isoquinoline-1,3(2 H,4 H)-Diones. *J. Org. Chem.* **2018**, *83* (6), 3348–3353.

(19) Doyle, M. P.; Siegfried, B.; Dellaria, J. F. Alkyl Nitrite-Metal Halide Deamination Reactions. 2. Substitutive Deamination of Arylamines by Alkyl Nitrites and Copper(II) Halides. A Direct and Remarkably Efficient Conversion of Arylamines to Aryl Halides. *J. Org. Chem.* **1977**, *42* (14), 2426–2431.

(20) Pfizer Patent - DE1943265, 1970, A1.

(21) Di Mola, A.; Tedesco, C.; Massa, A. Metal-Free Air Oxidation in a Convenient Cascade Approach for the Access to Isoquinoline-1,3,4(2H)-Triones. *Molecules* **2019**, *24* (11), 2177.

(22) Golushko, A.; Dar'in, D.; Kantin, G.; Guranova, N.; Vasilyev, A. V.; Krasavin, M. Medicinally Relevant Modification of the Isoquinoline-1,3-Dione Scaffold via Metal-Free Arylation and Fluorination of Diazo Homophthalimides in Brønsted Acids. *Synthesis* **2019**, *51* (20), 3815–3824.

15. NMR Spectra

4-methoxy-2-methylbenzoic acid (**S1**)

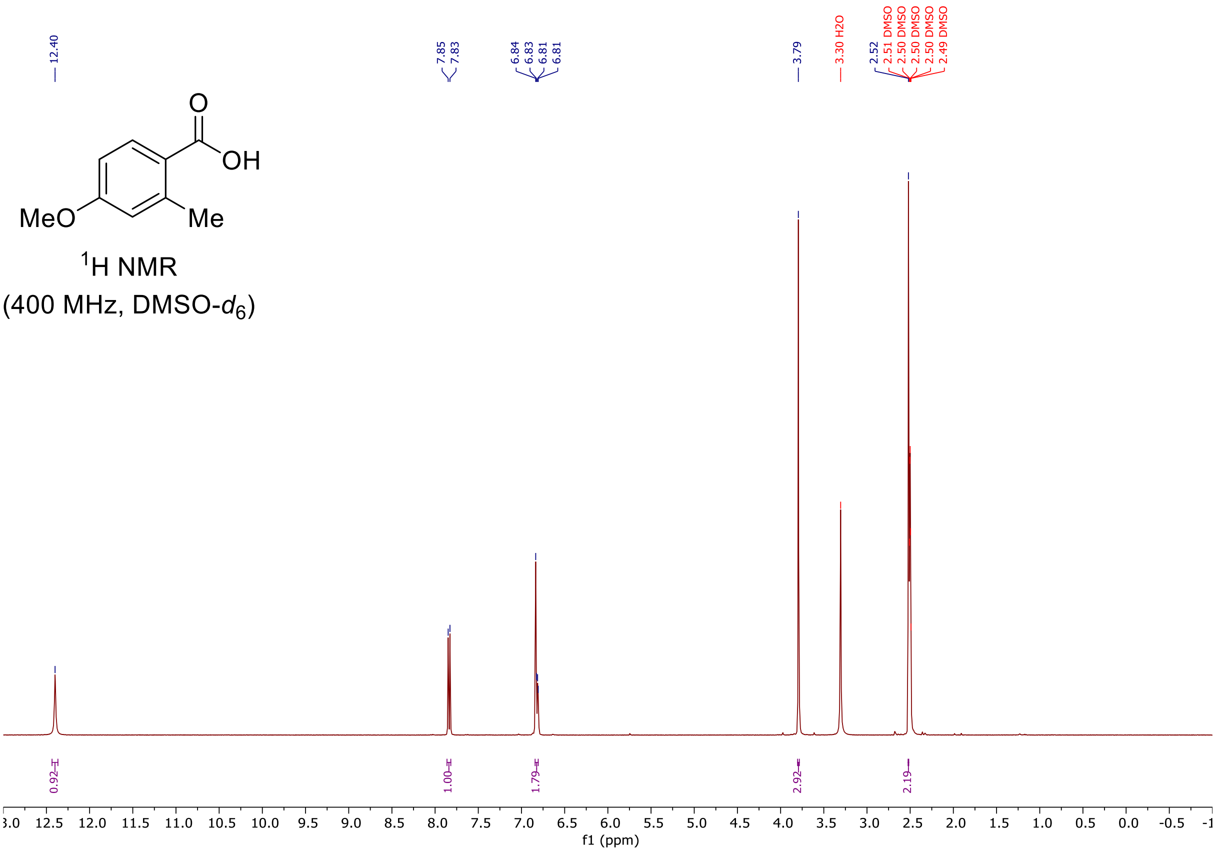

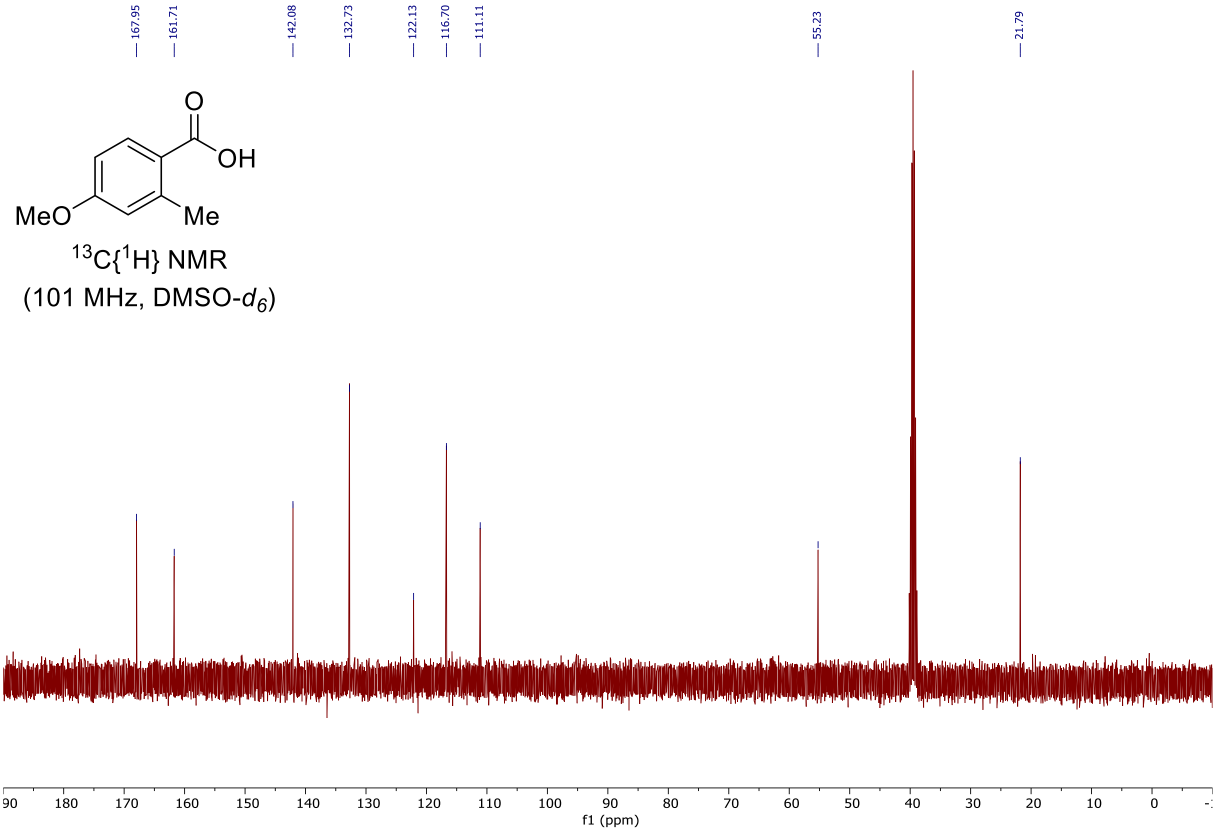

2-(carboxymethyl)-4-methoxybenzoic acid (**S2**)

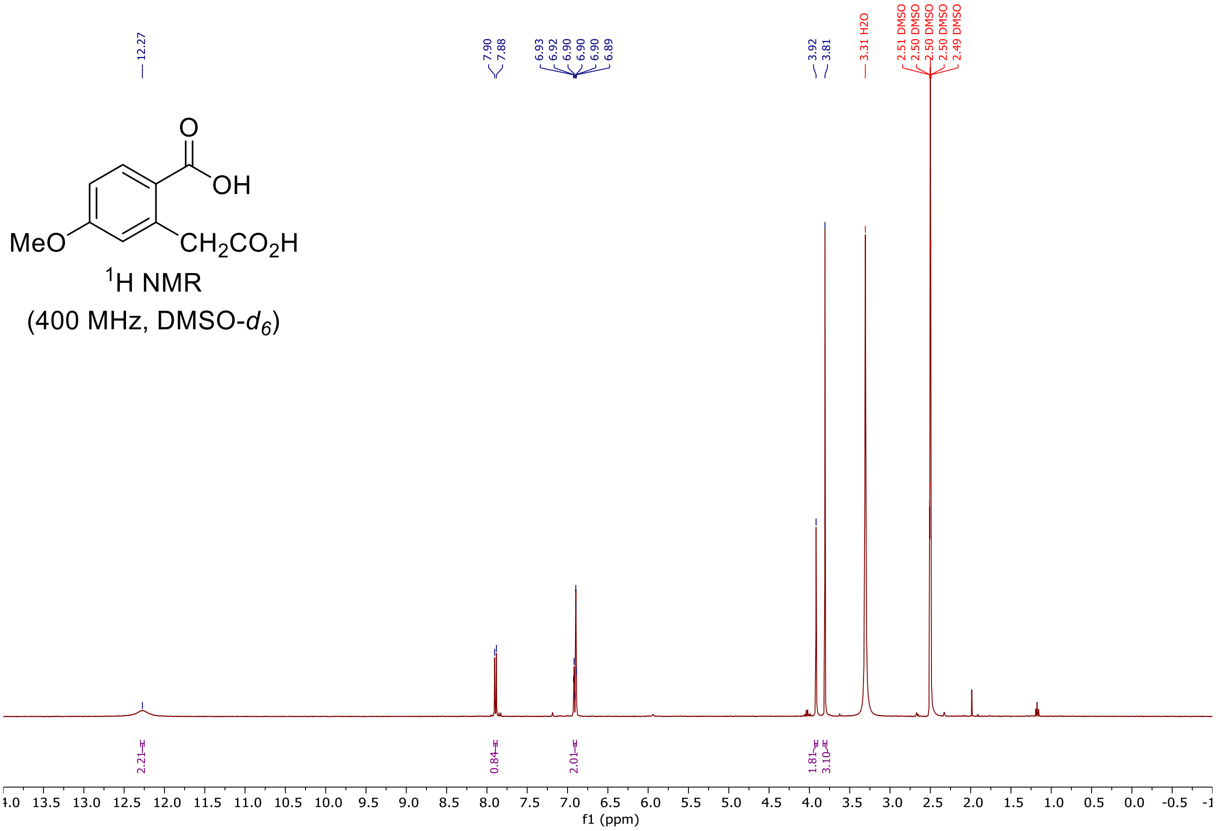

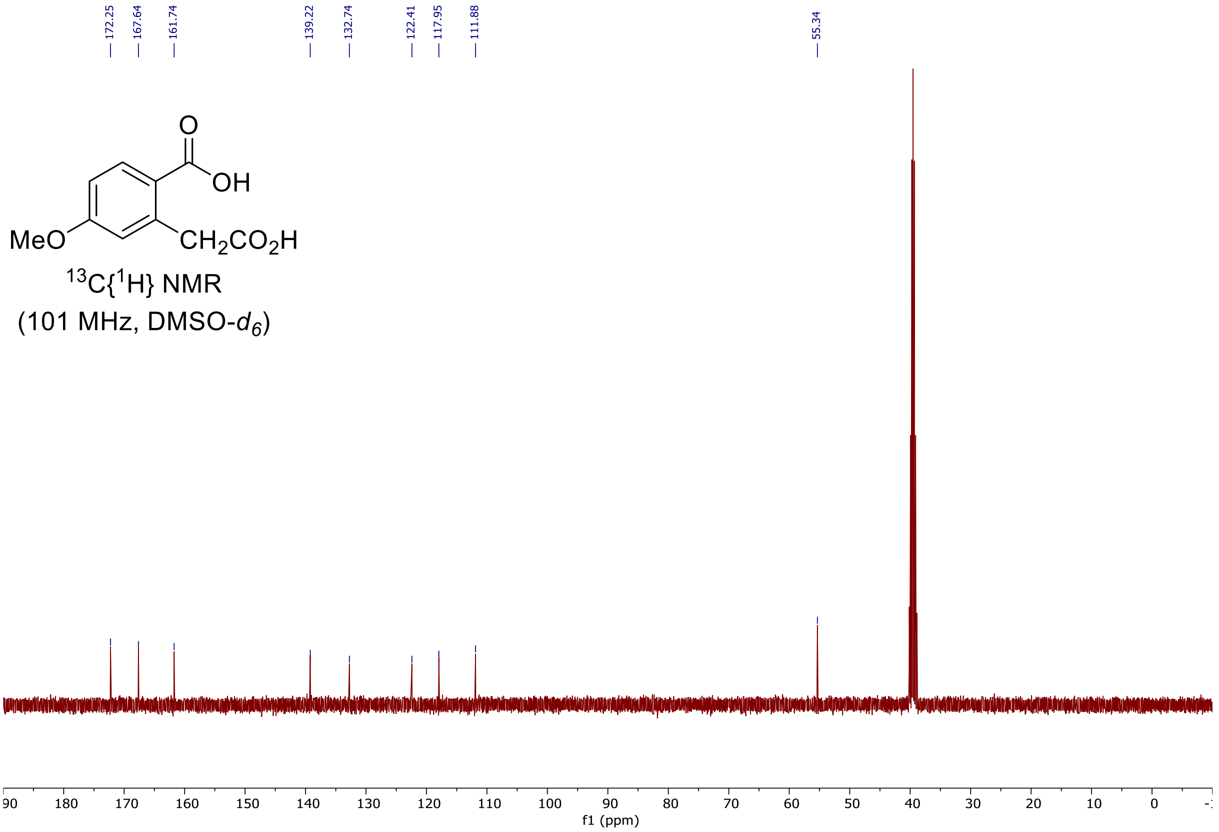

2-(carboxymethyl)-5-chlorobenzoic acid (**S3**)

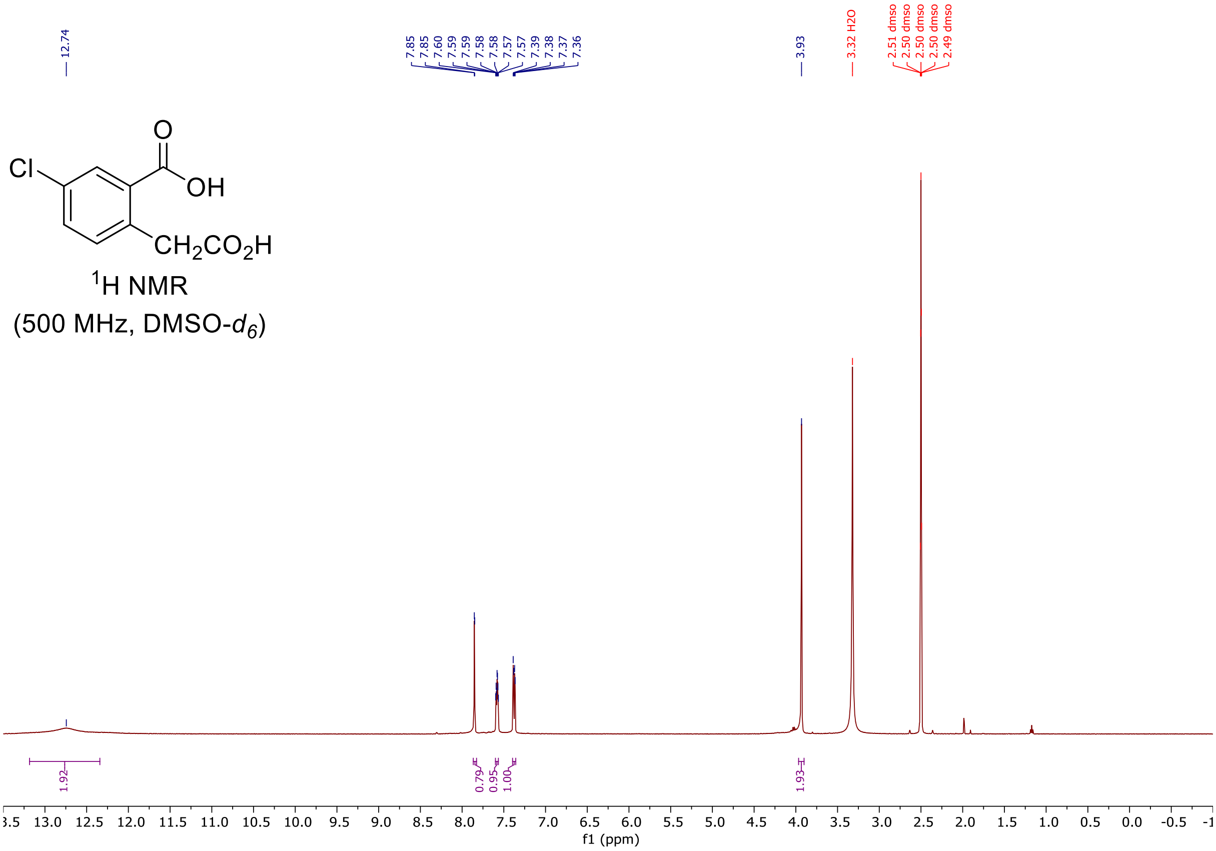

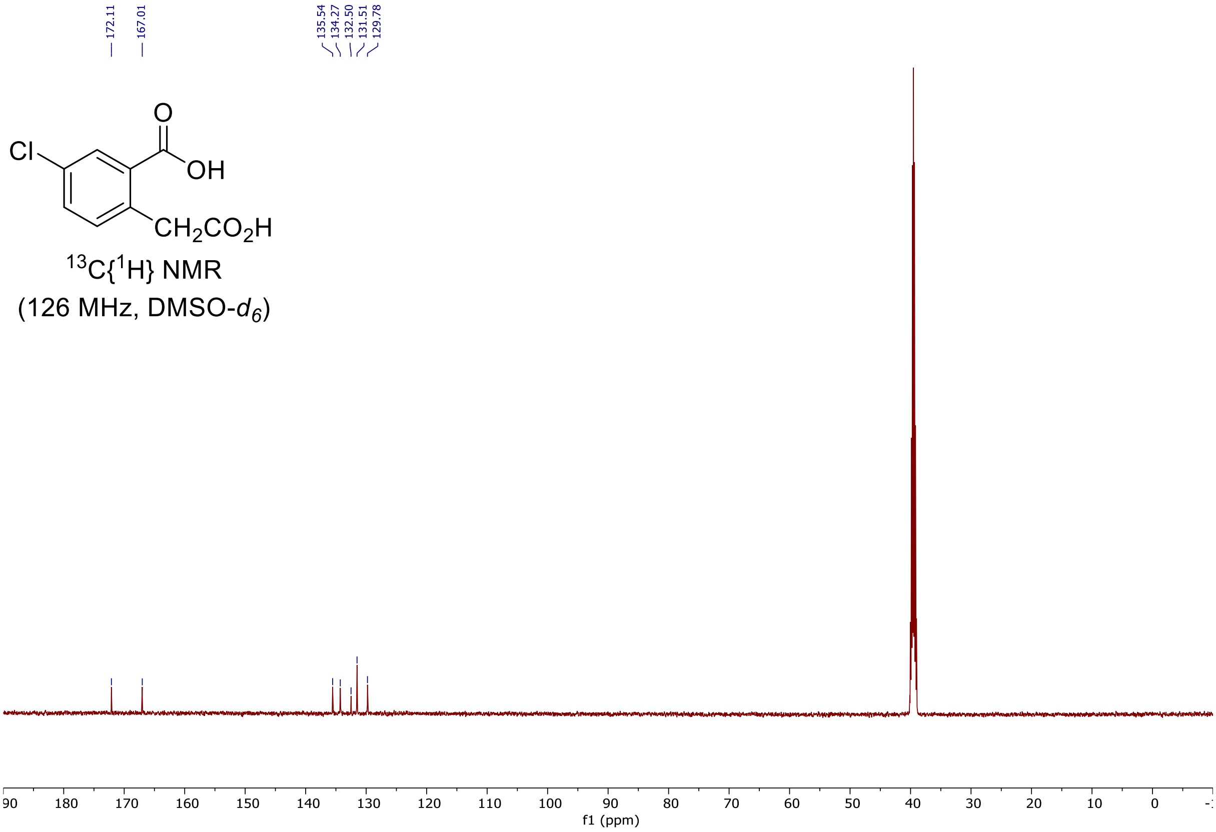

2-(carboxymethyl)-3-chlorobenzoic acid (**S4**)

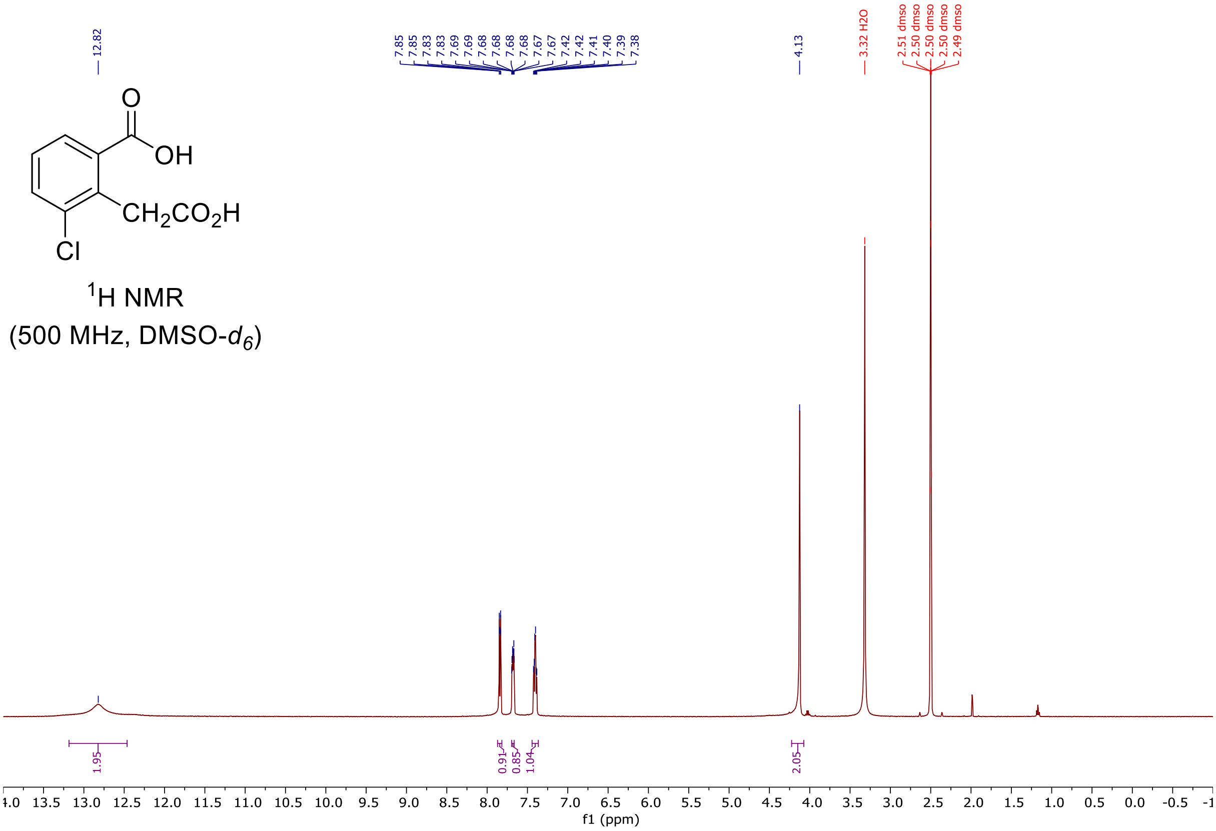

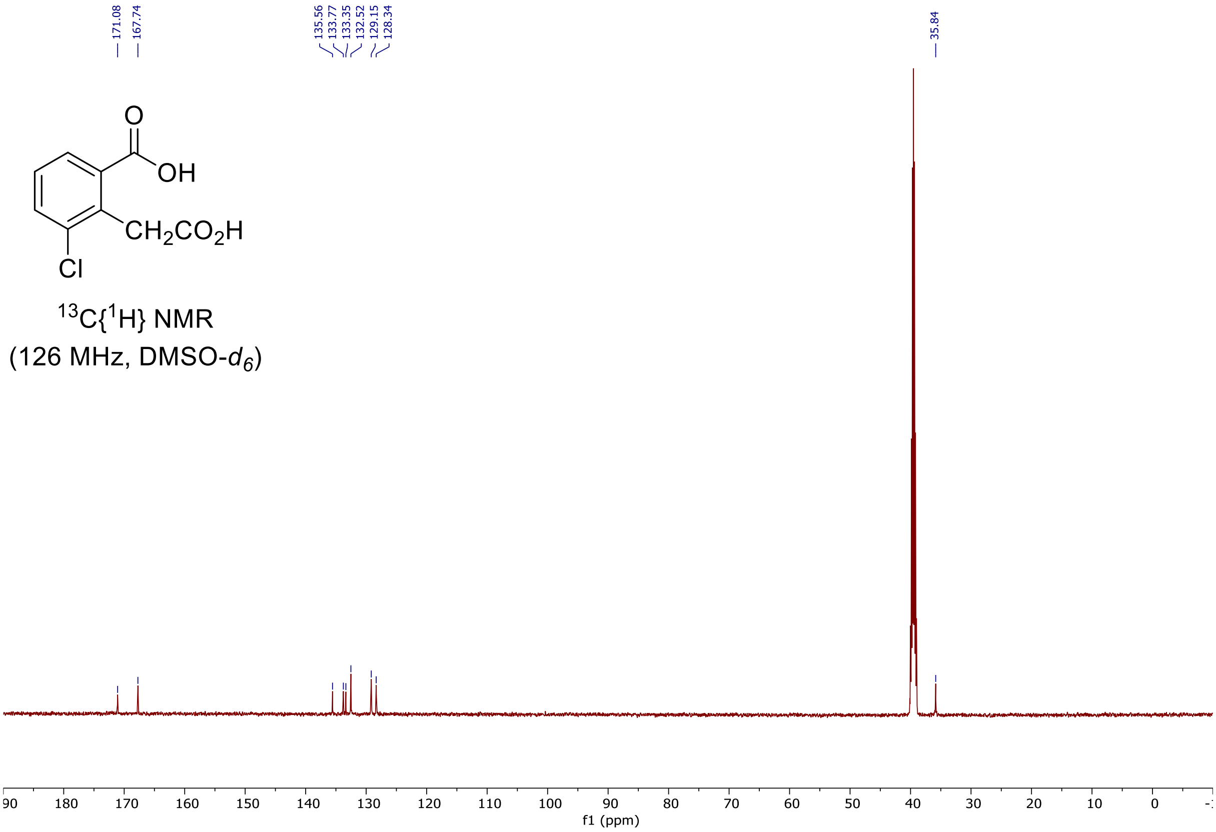

4-bromo-2-(carboxymethyl)benzoic acid (**S5**)

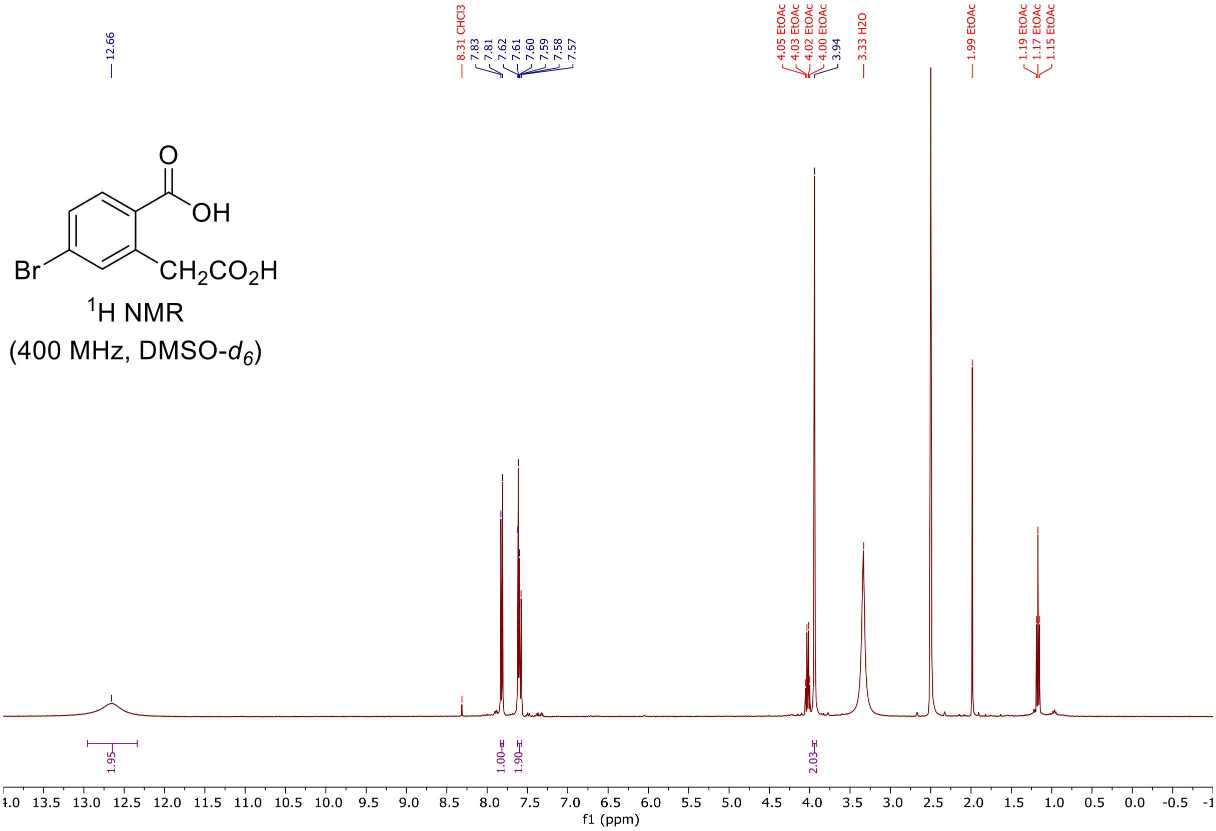

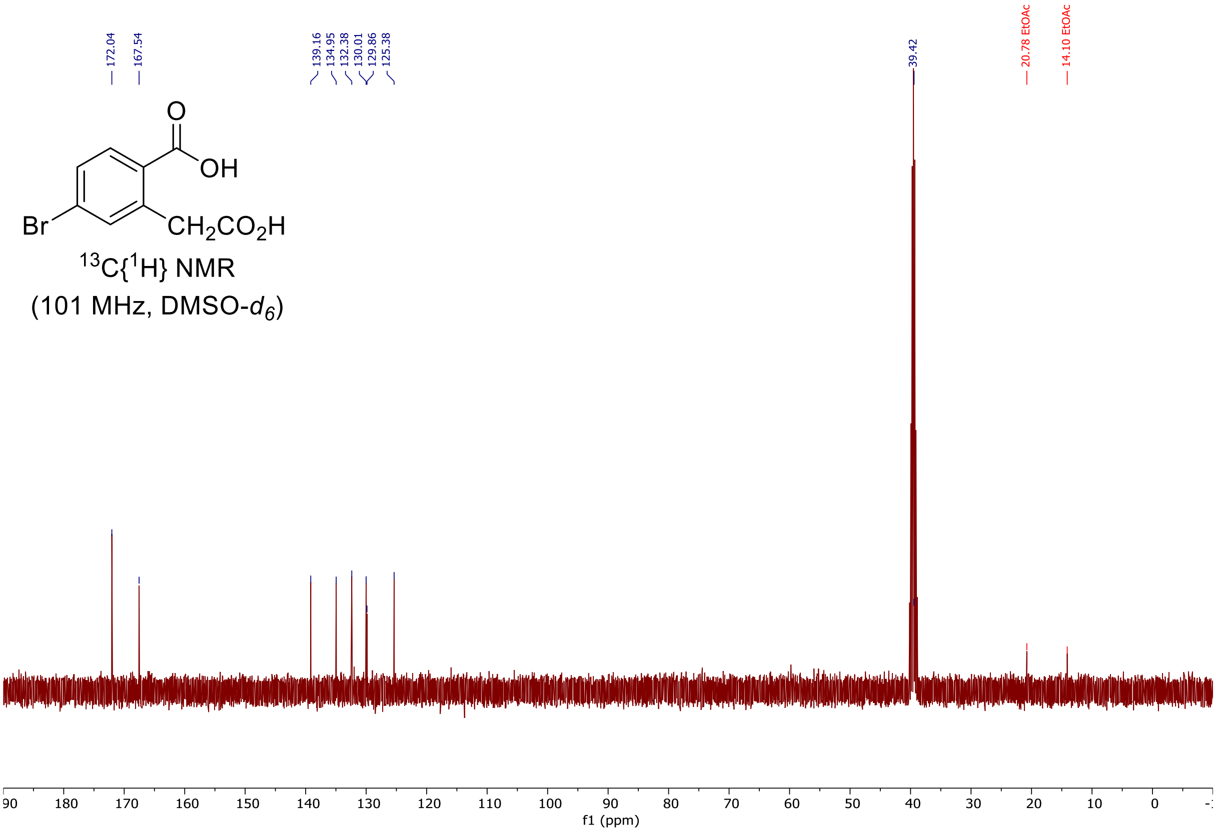

7-chloro-2-methylisoquinoline-1,3(2*H*,4*H*)-dione (**S6**)

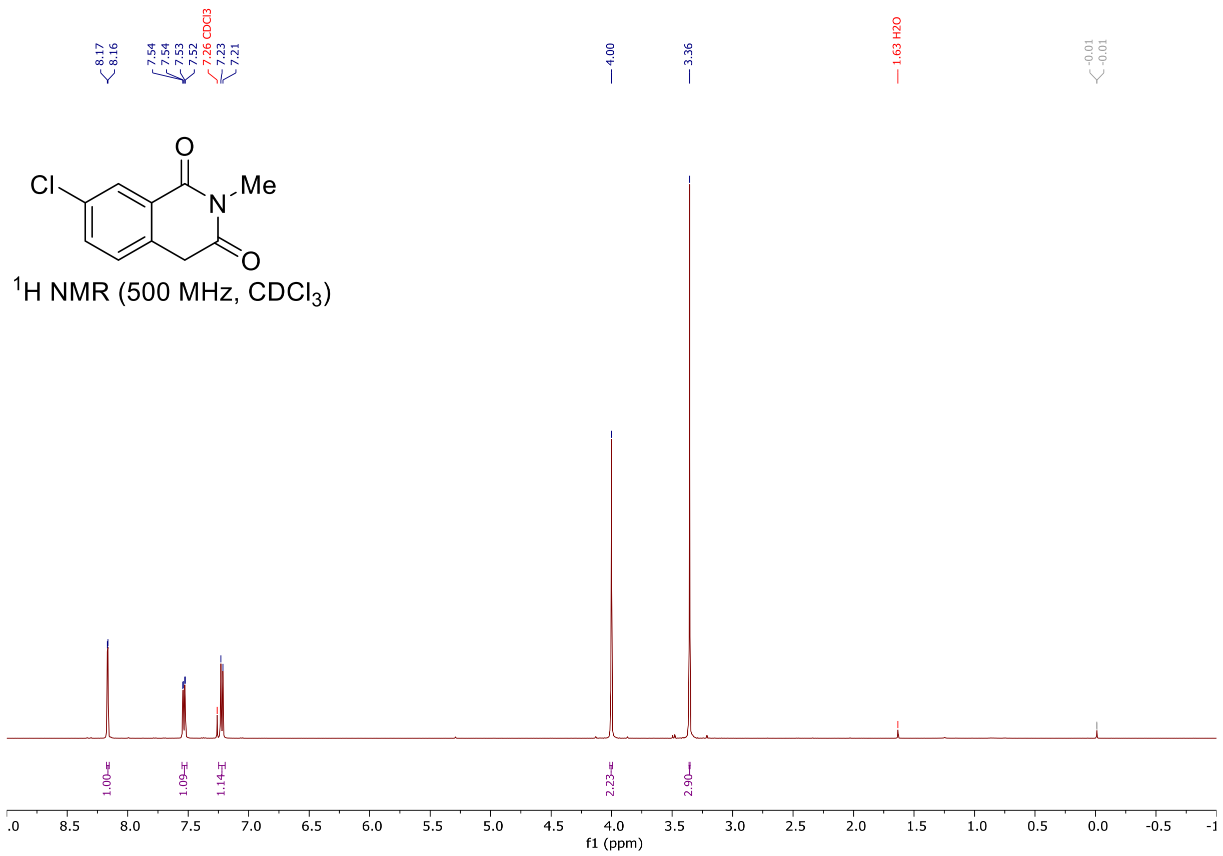

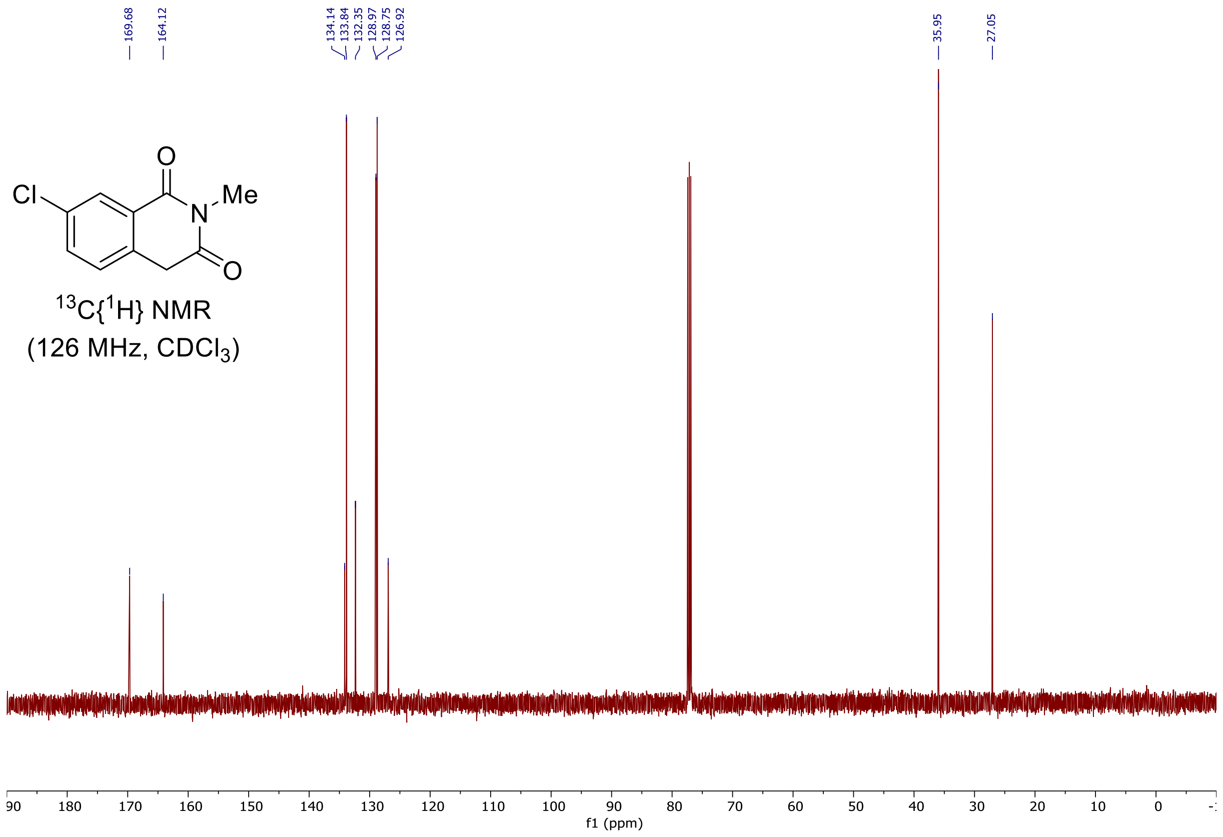

7-bromo-2-methylisoquinoline-1,3(2*H*,4*H*)-dione (**S7**)

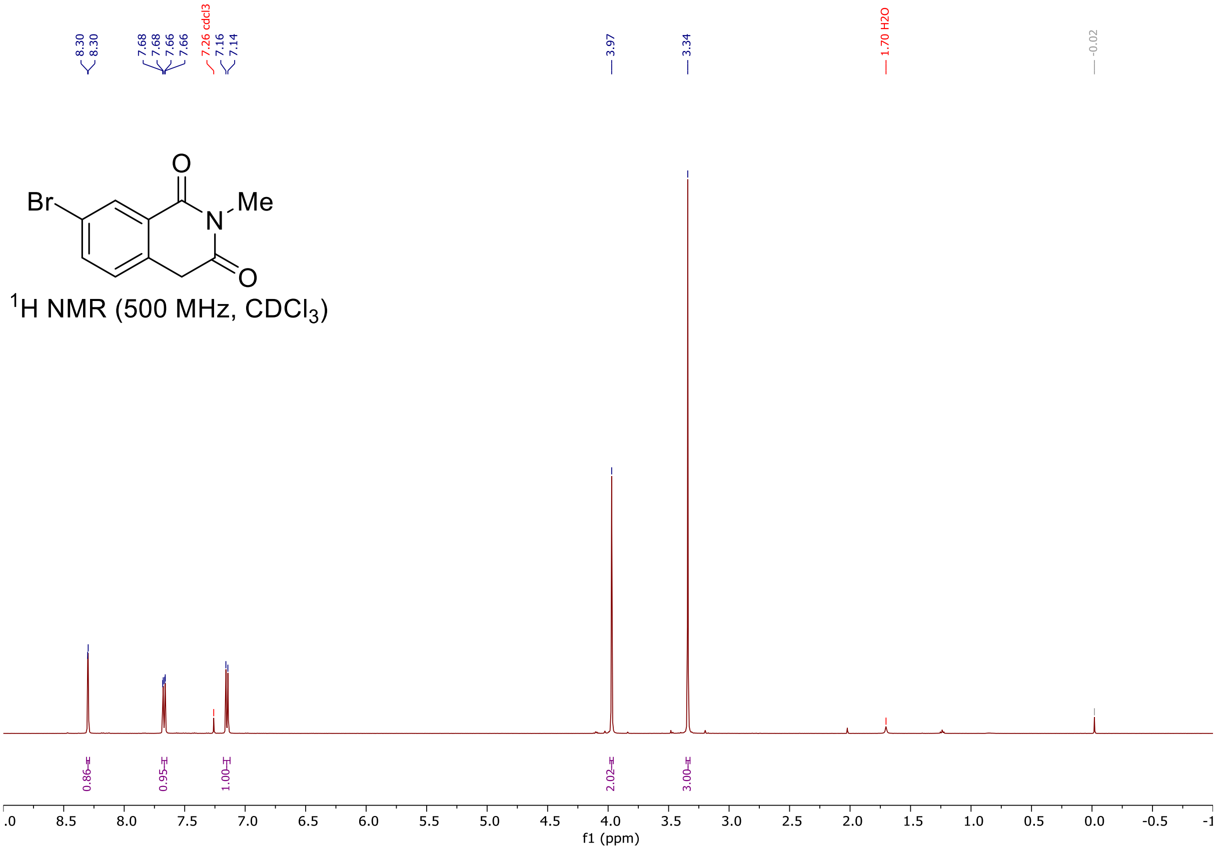

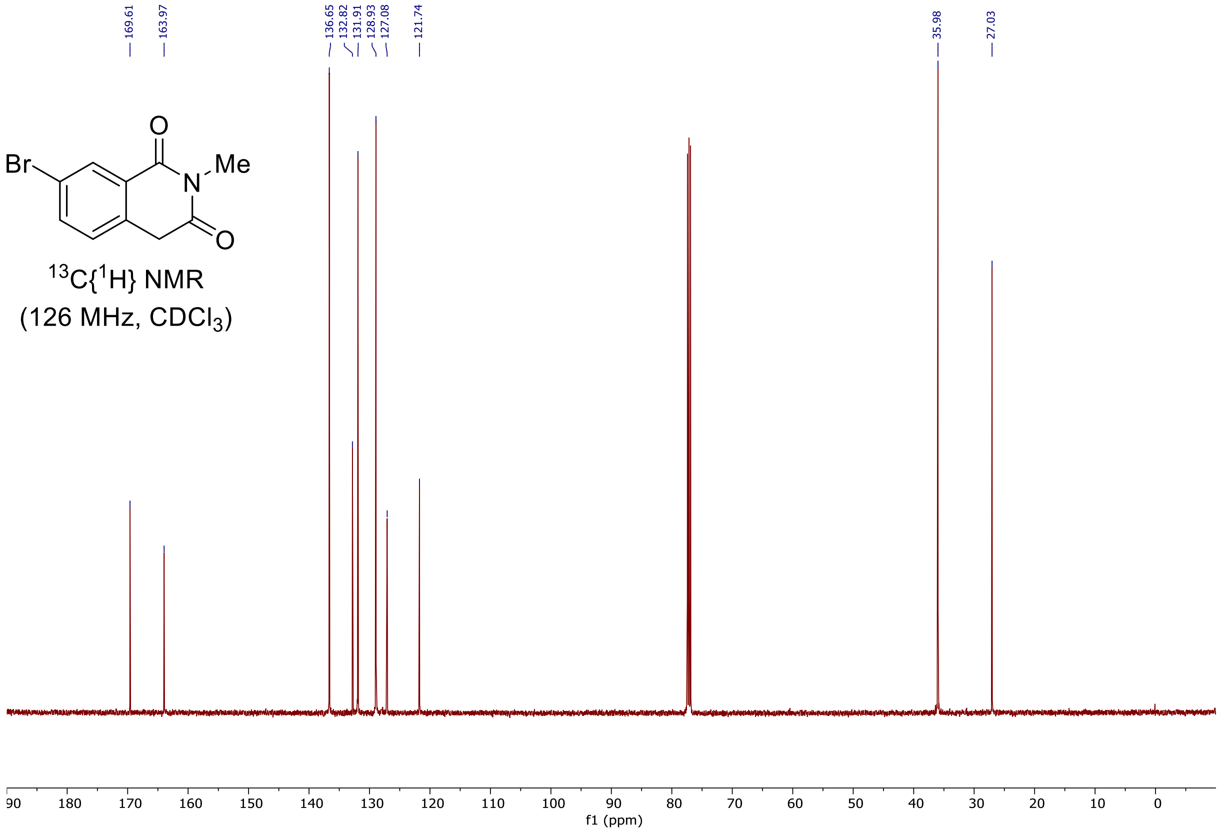

2,7-dimethylisoquinoline-1,3(2*H*,4*H*)-dione (**S8**)

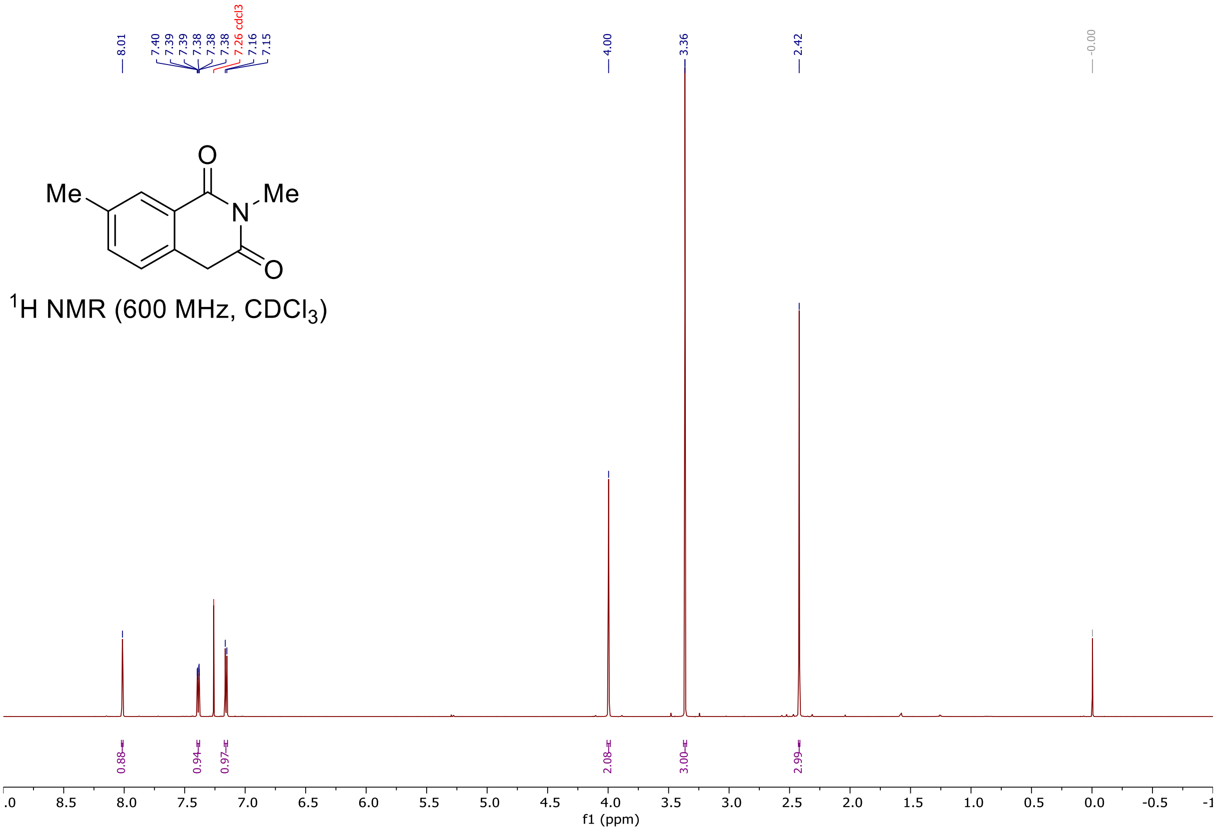

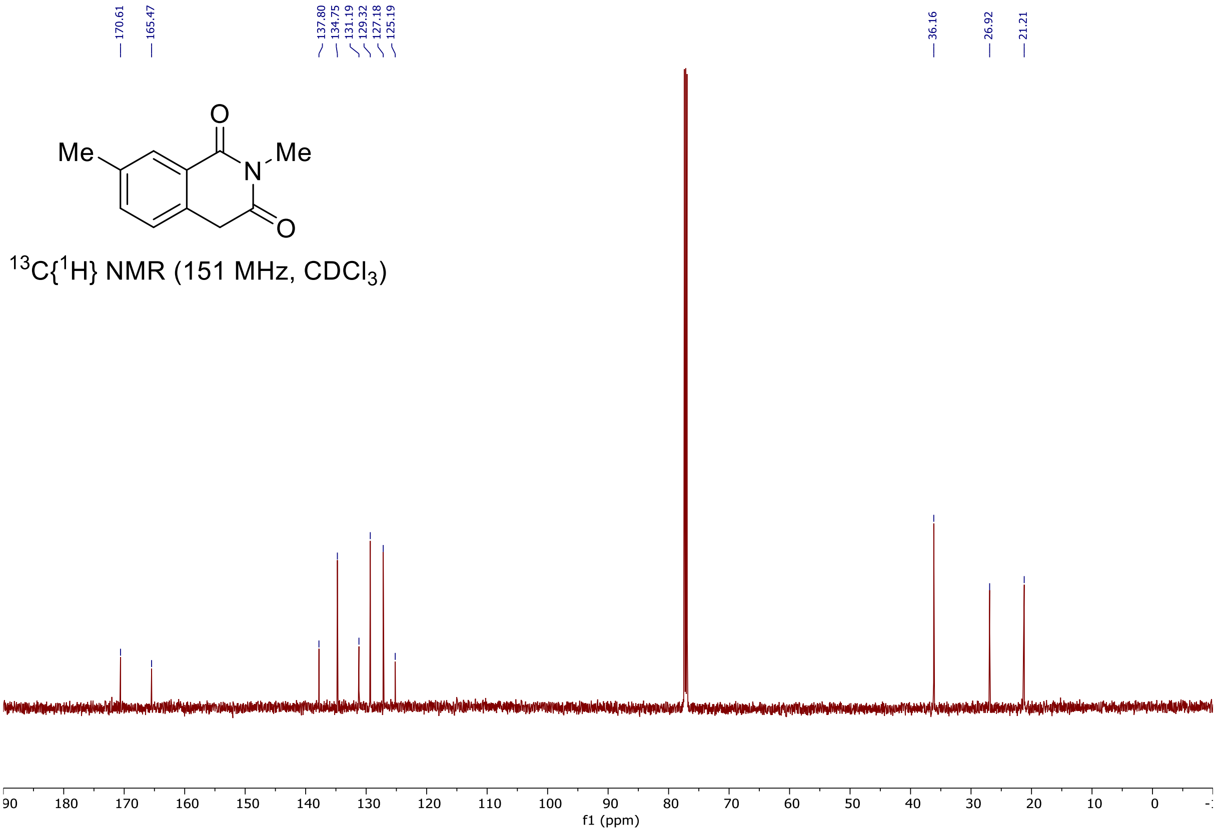

7-methoxy-2-methylisoquinoline-1,3(2*H*,4*H*)-dione (**S9**)

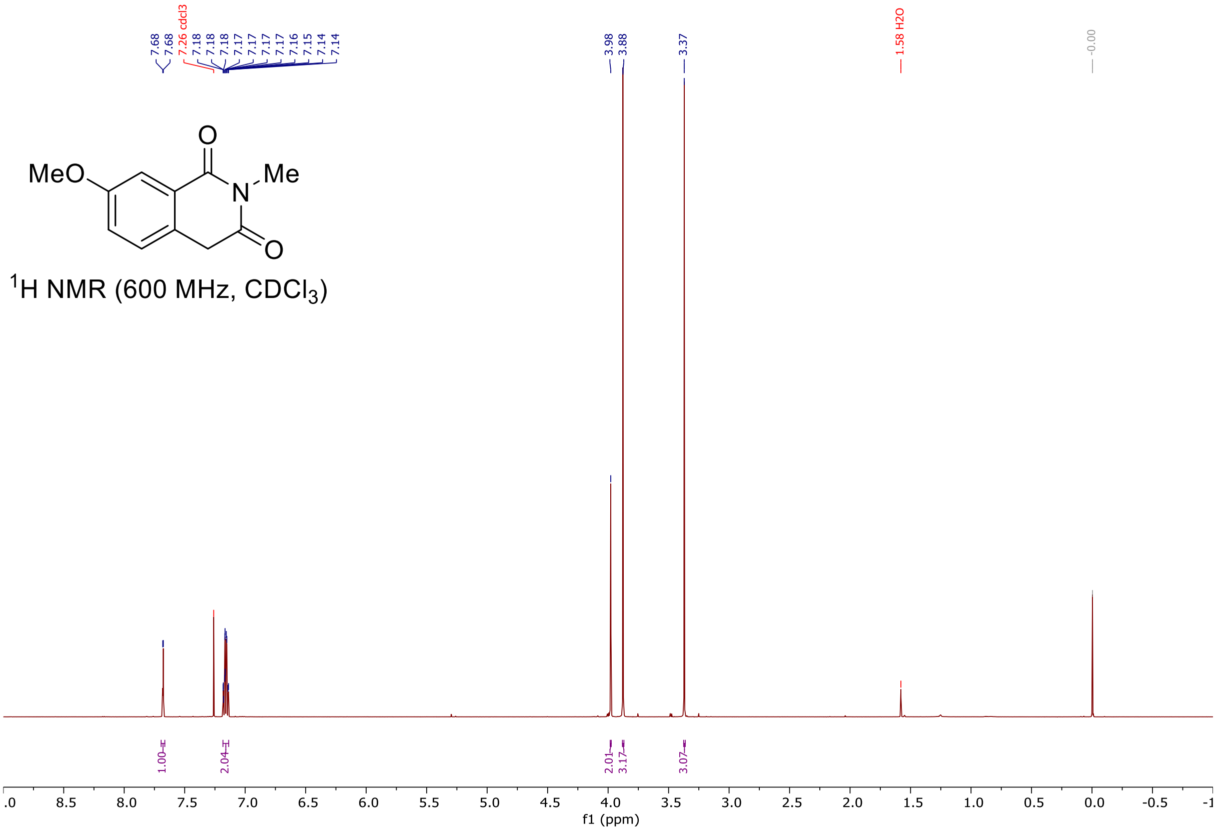

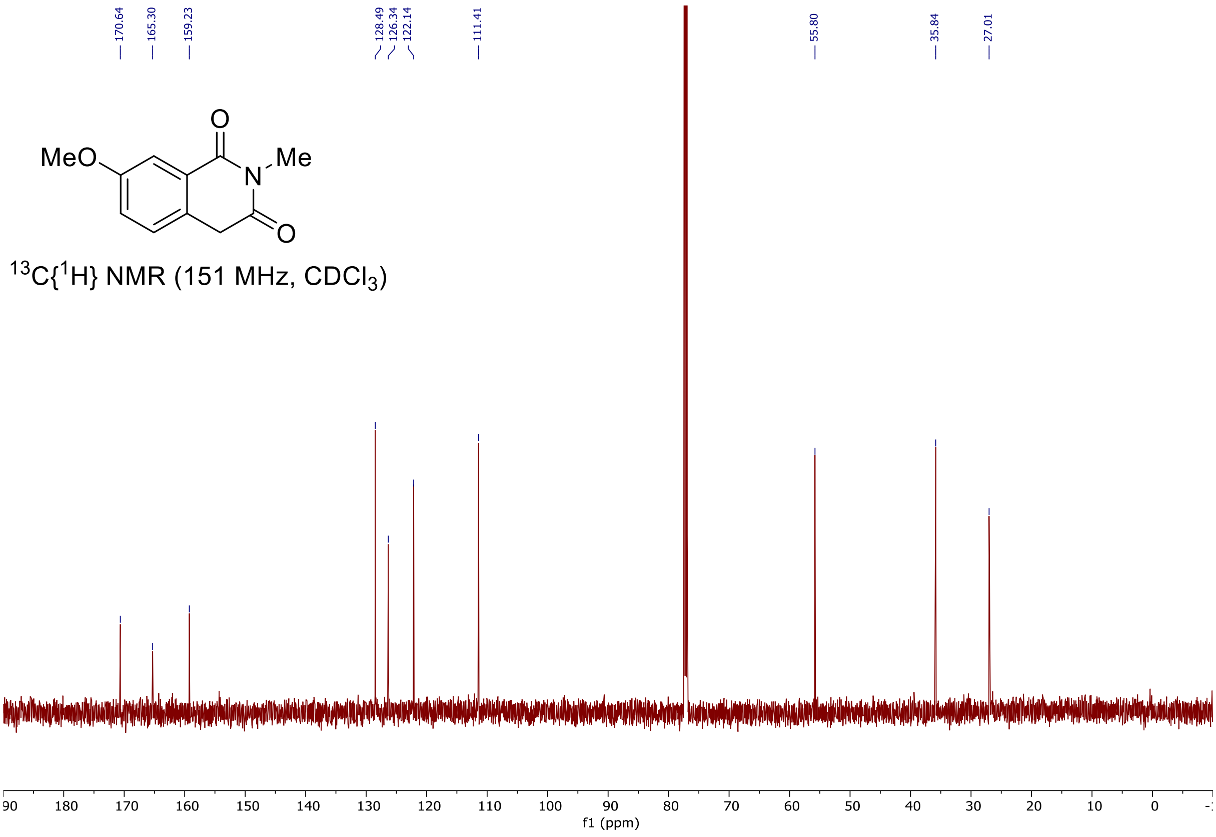

6-methoxy-2-methylisoquinoline-1,3(2*H*,4*H*)-dione (**S10**)

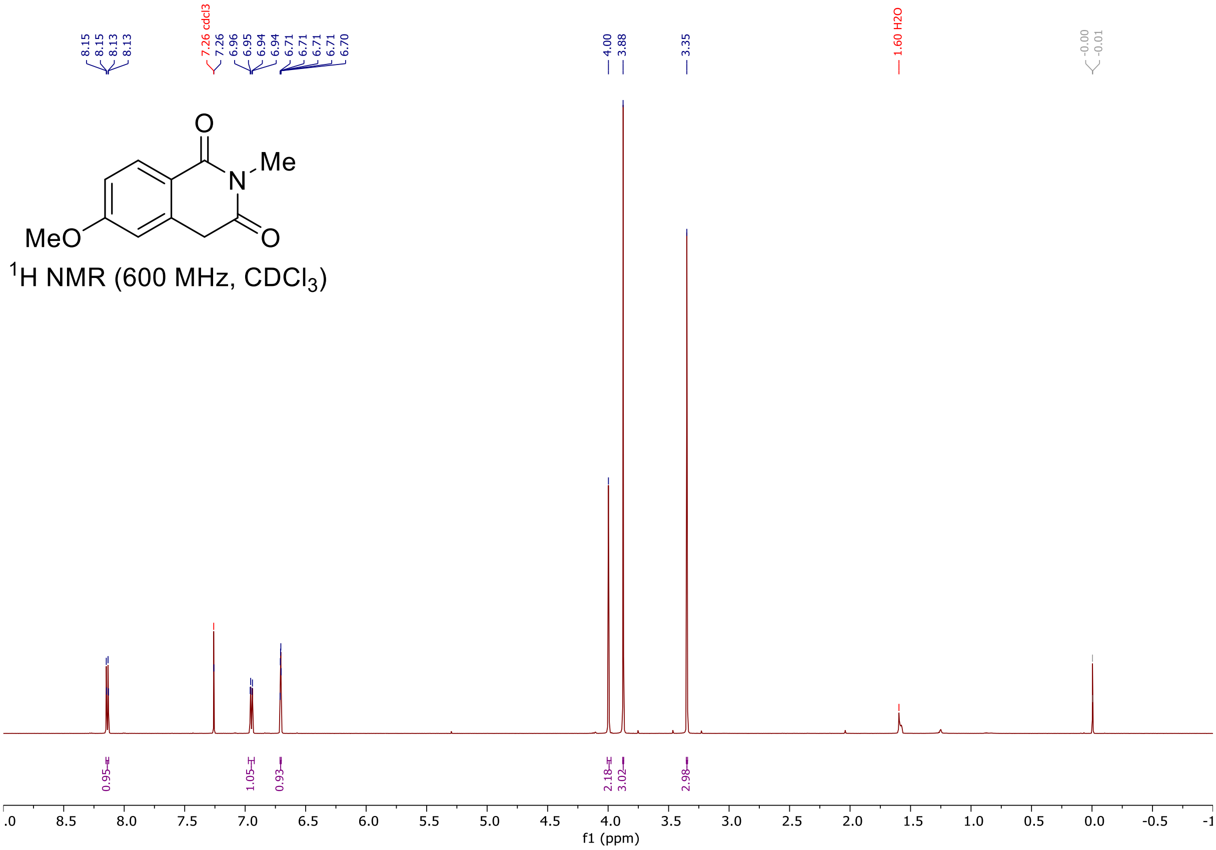

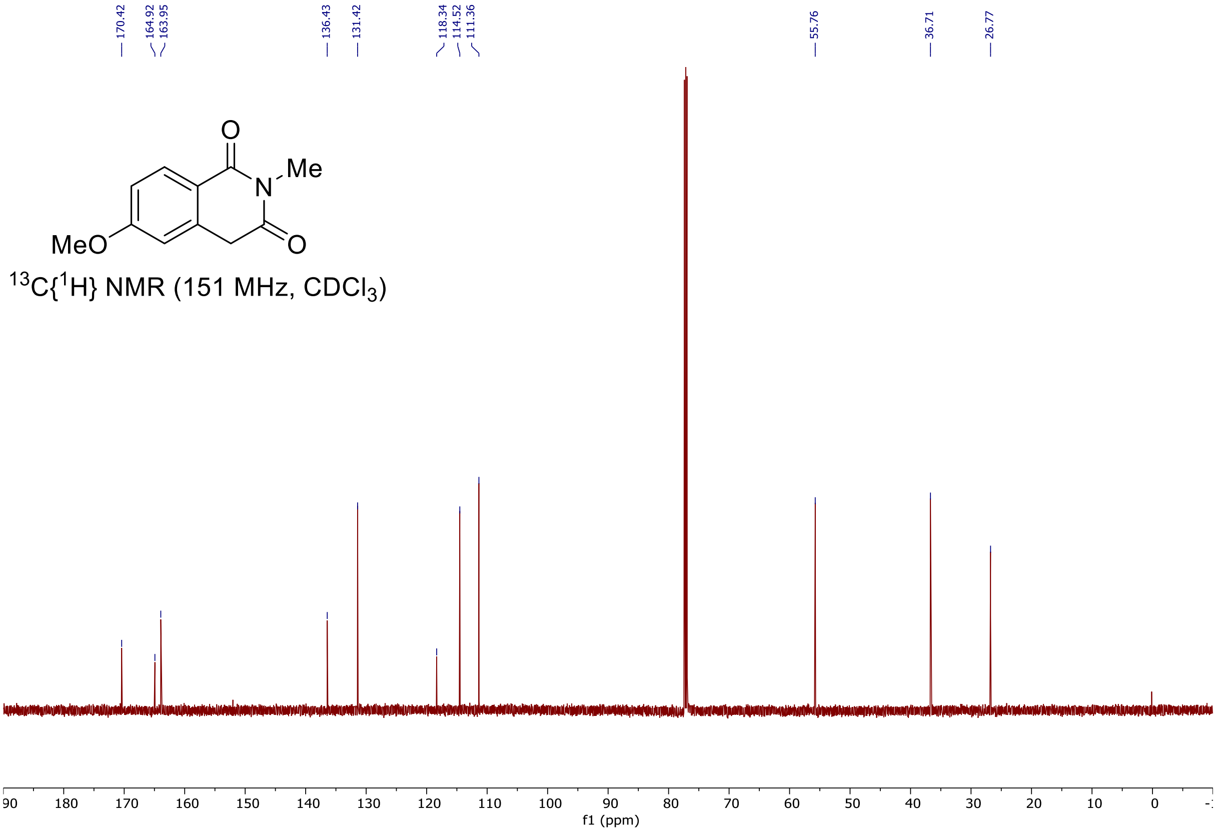

6-chloro-2-methylisoquinoline-1,3(2*H*,4*H*)-dione (**S11**)

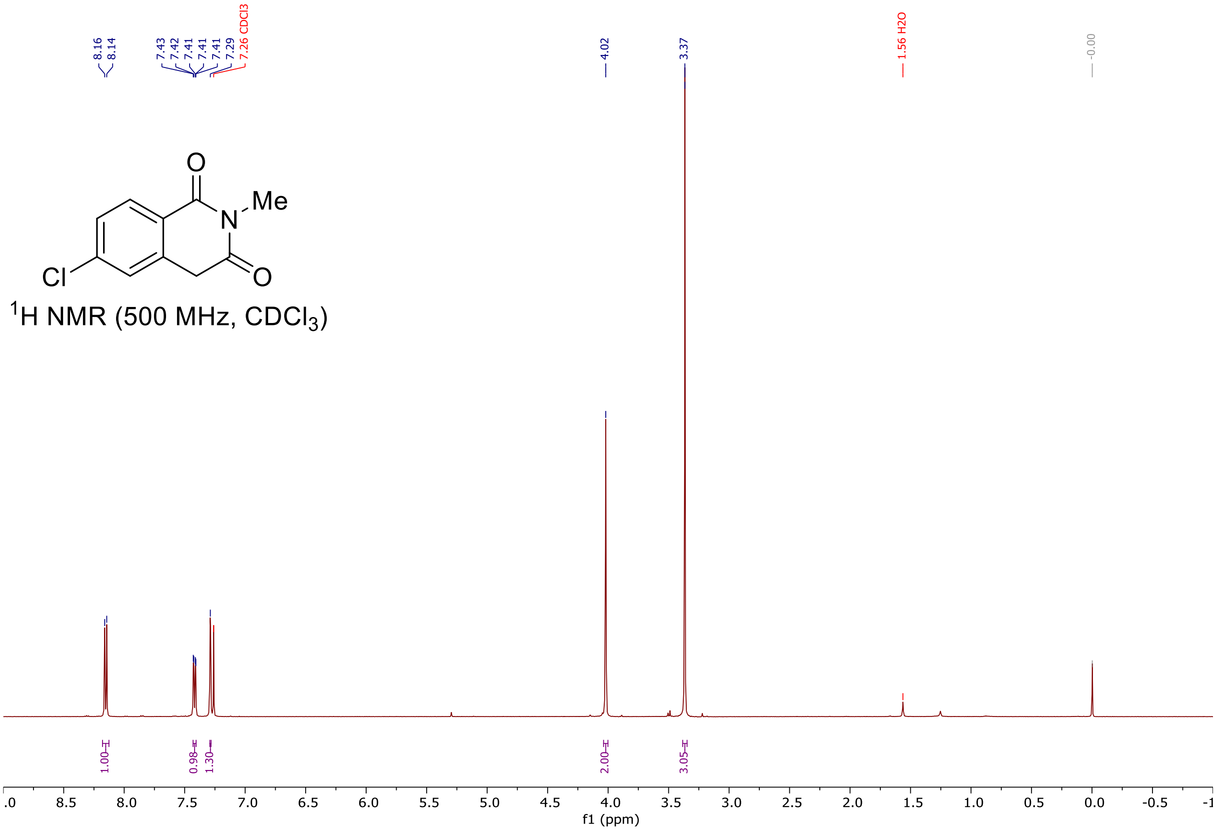

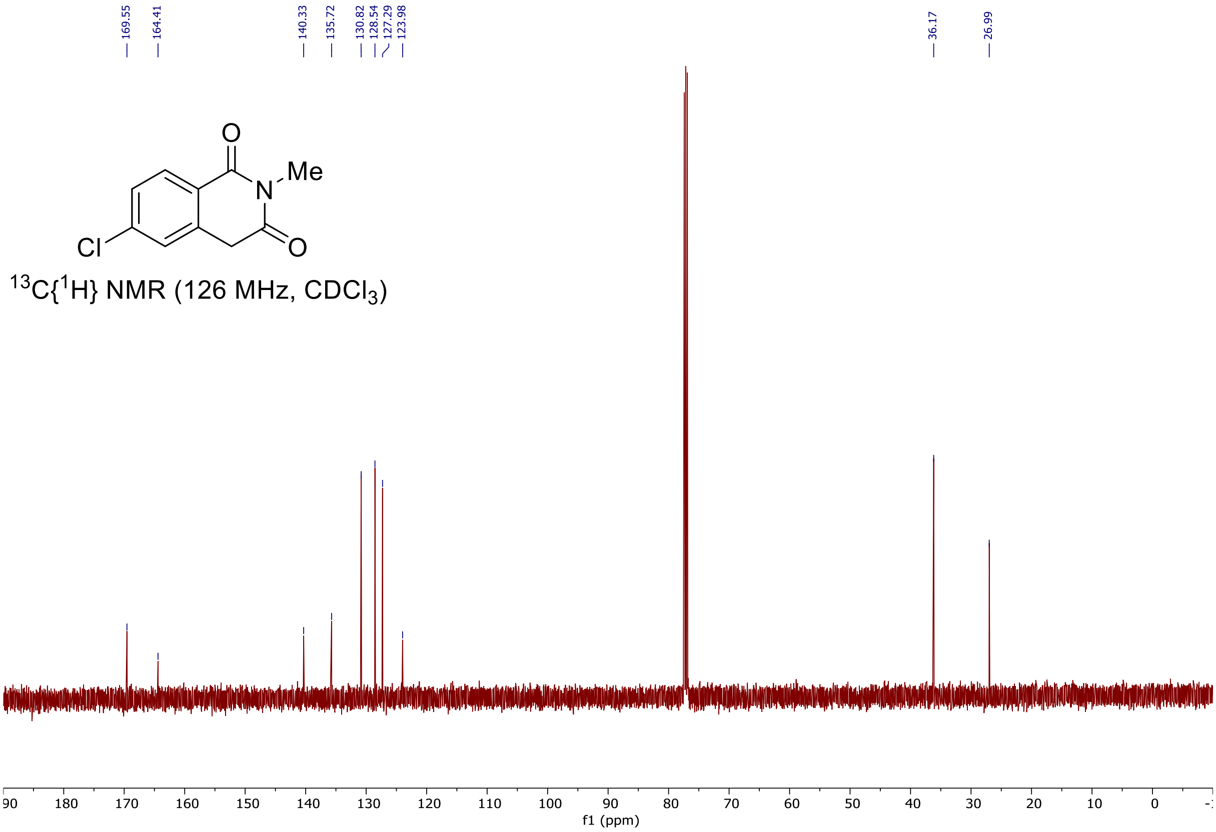

6-bromo-2-methylisoquinoline-1,3(2*H*,4*H*)-dione (**S12**)

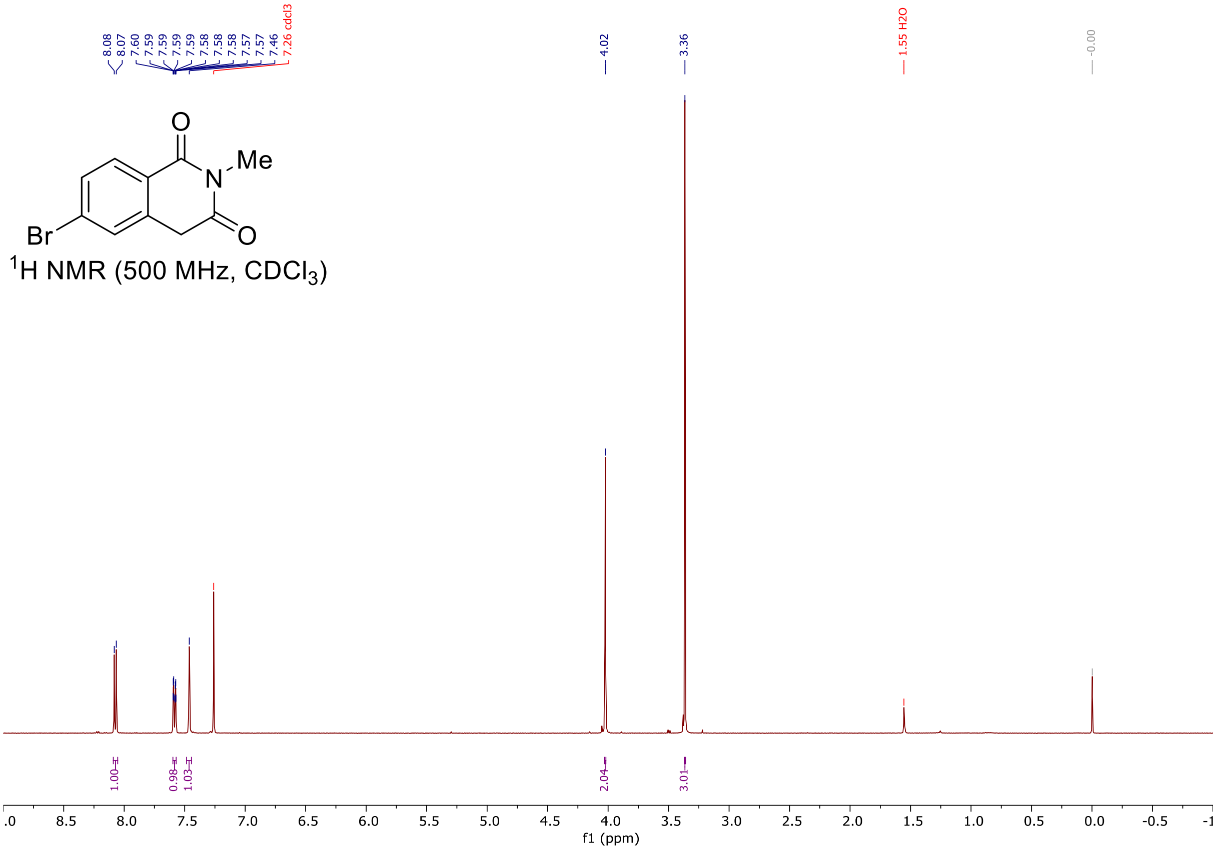

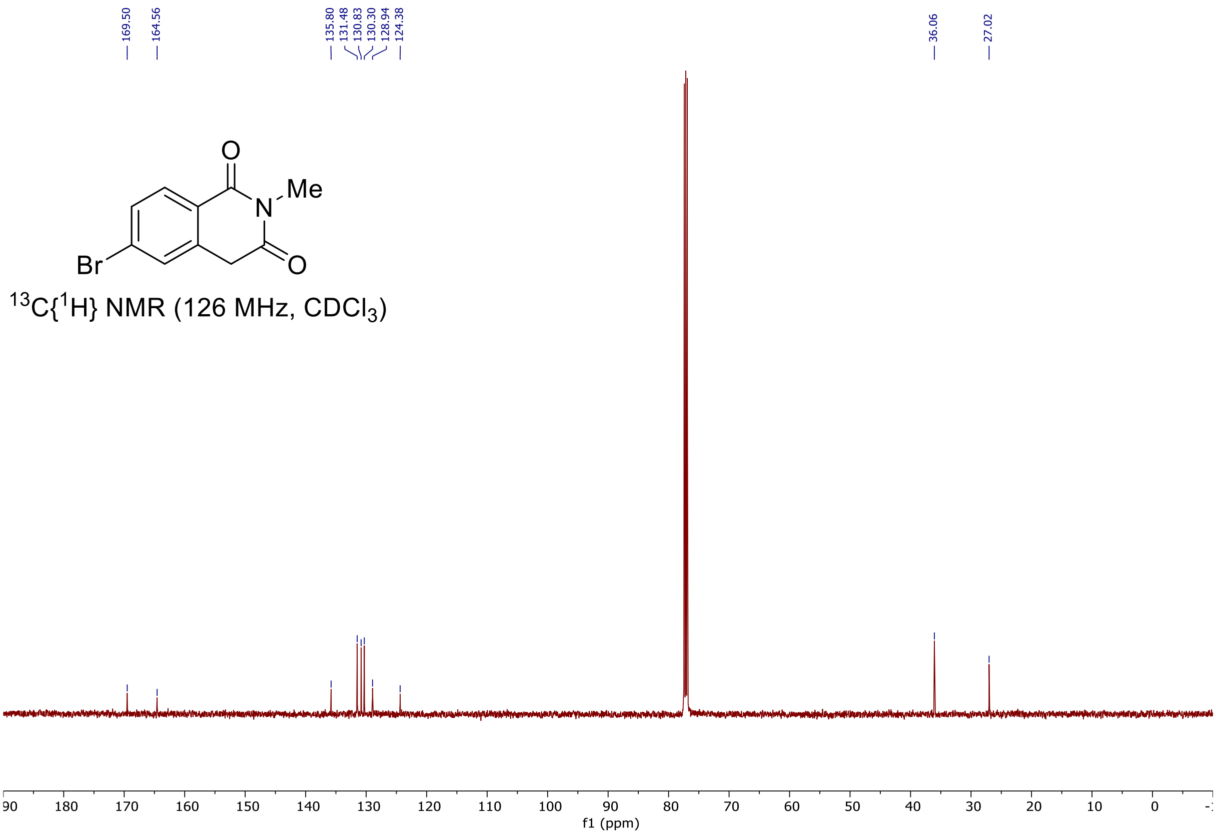

8-chloro-2-methylisoquinoline-1,3(2*H*,4*H*)-dione (**S13**)

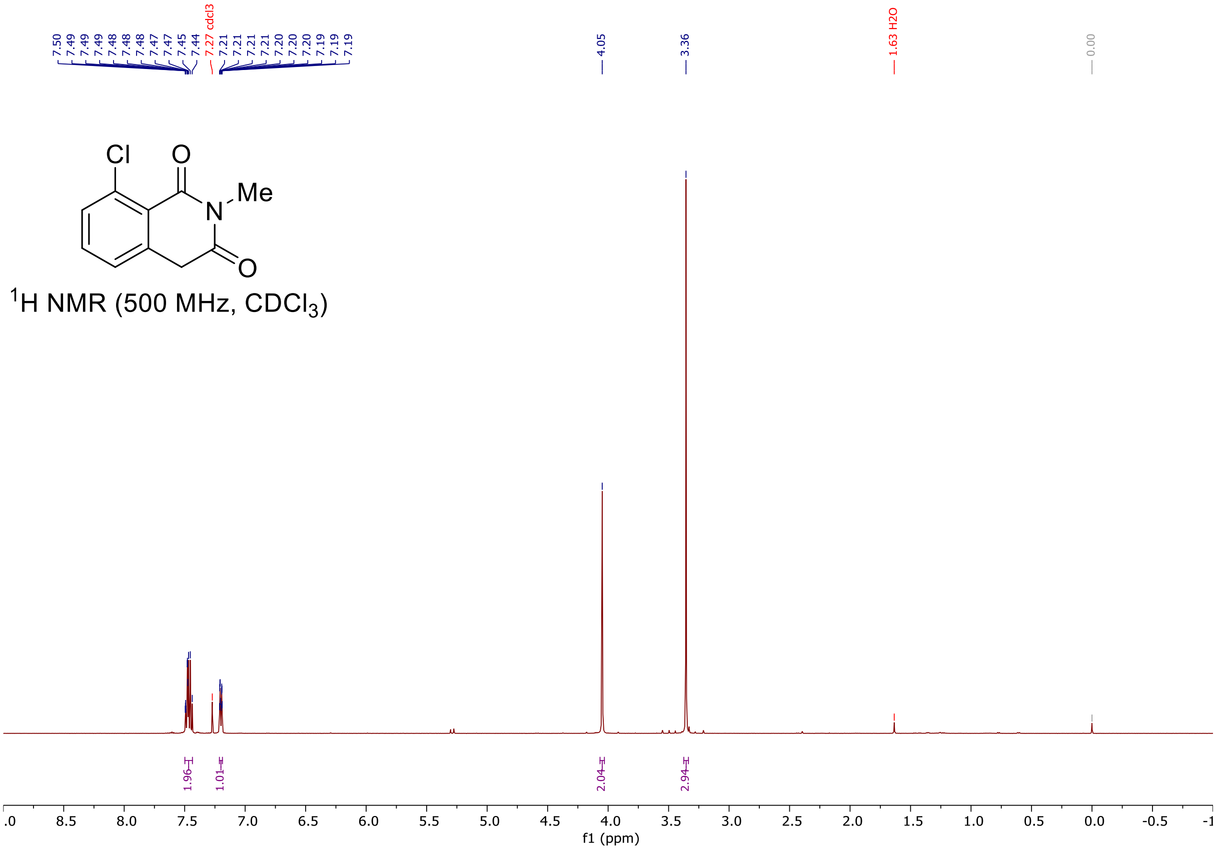

^{1}H NMR (500 MHz, $CDCl_3$)

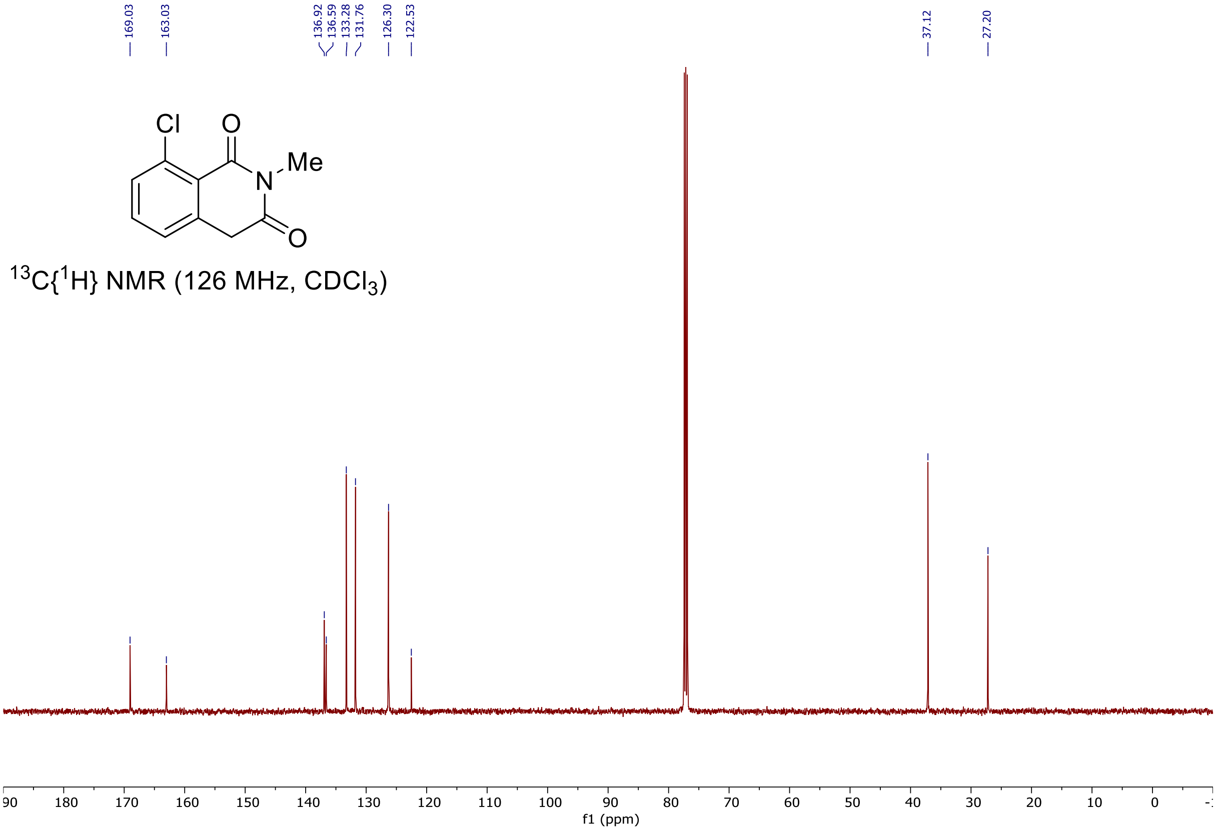

$^{13}C\{^{1}H\}$ NMR (126 MHz, $CDCl_3$)

5-chloro-2-methylisoquinoline-1,3(2*H*,4*H*)-dione (**S14**)

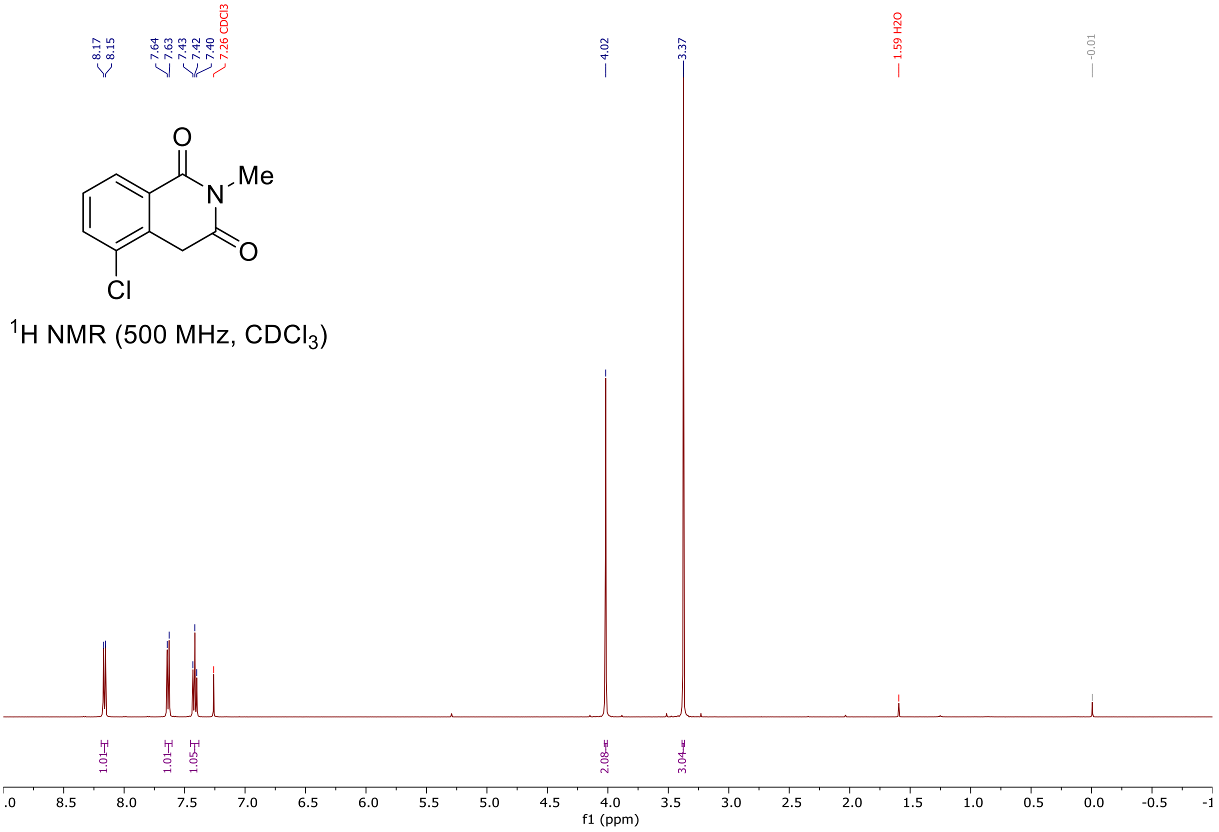

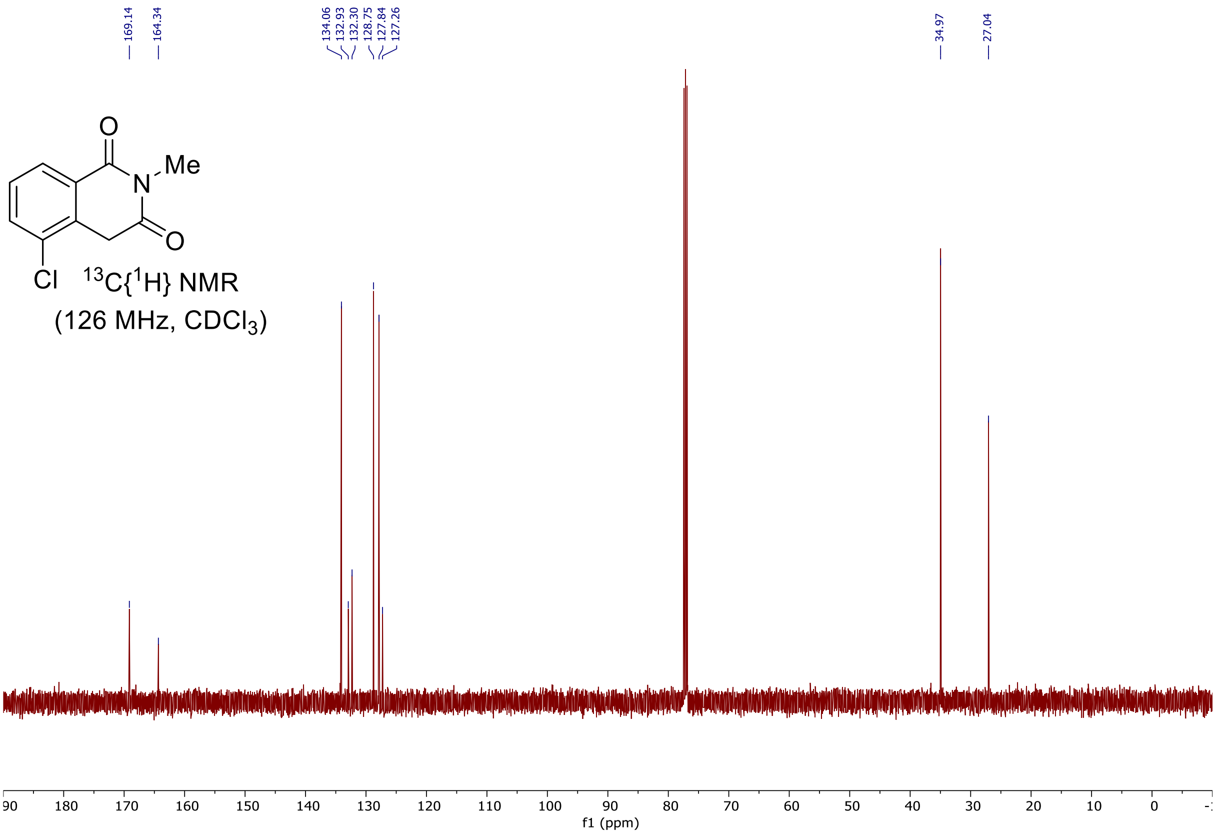

2-methyl-2*H*-benzo[*e*][1,2]thiazin-3(4*H*)-one 1,1-dioxide (**S15**)

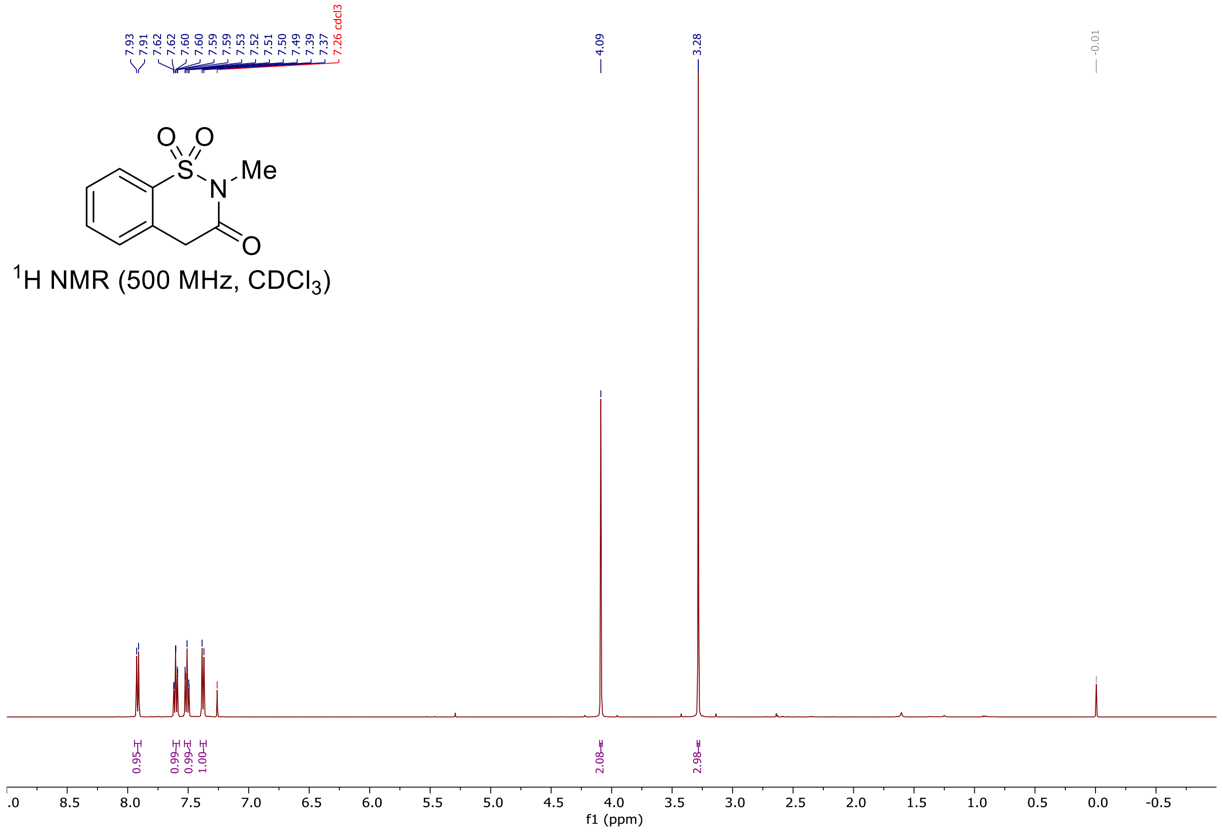

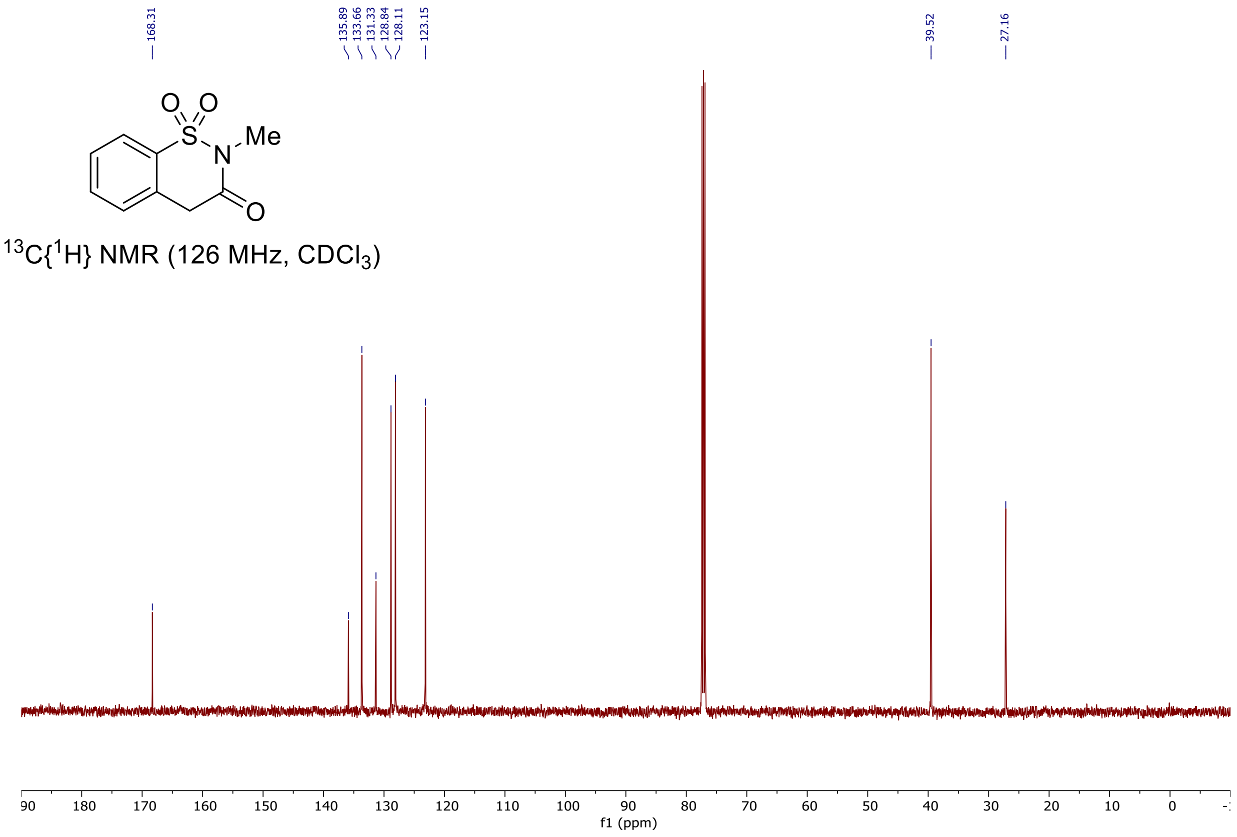

2-benzyl-2*H*-benzo[*e*][1,2]thiazin-3(4*H*)-one 1,1-dioxide (**S16**)

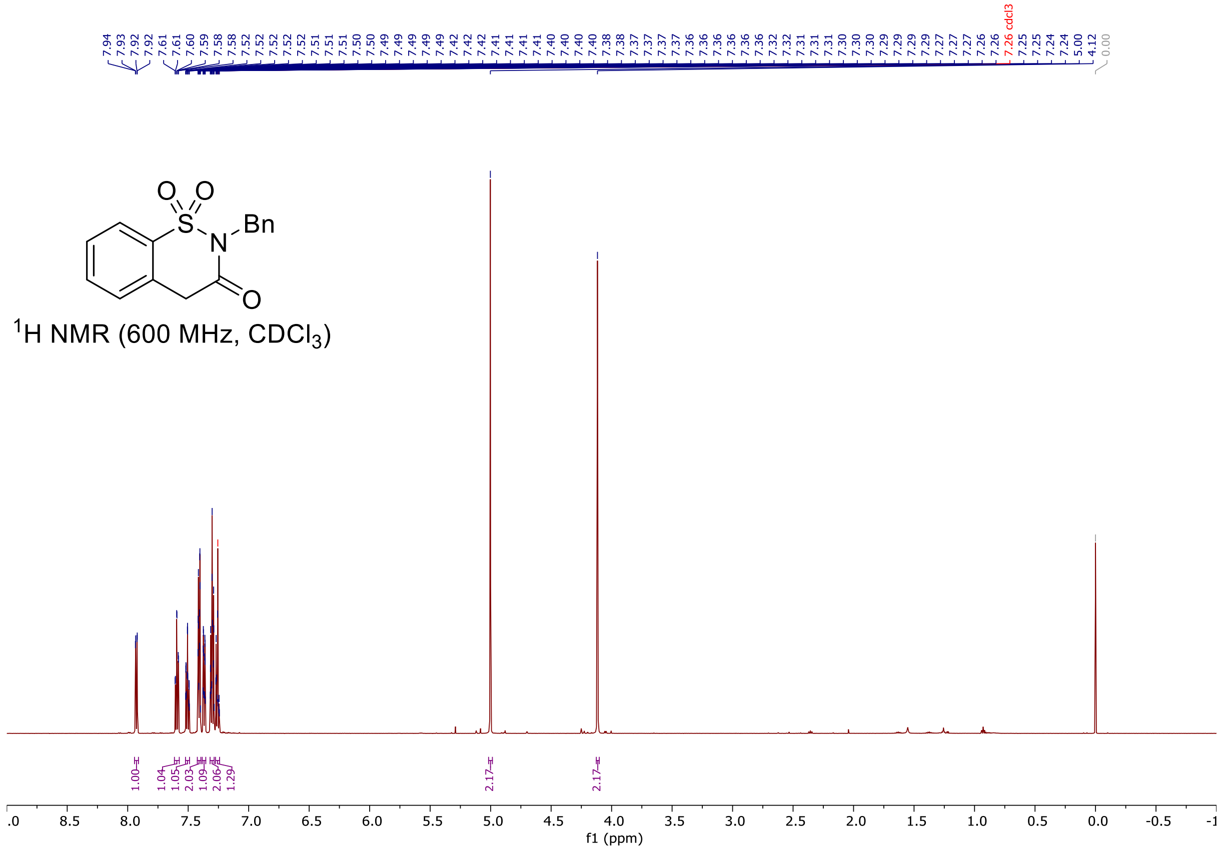

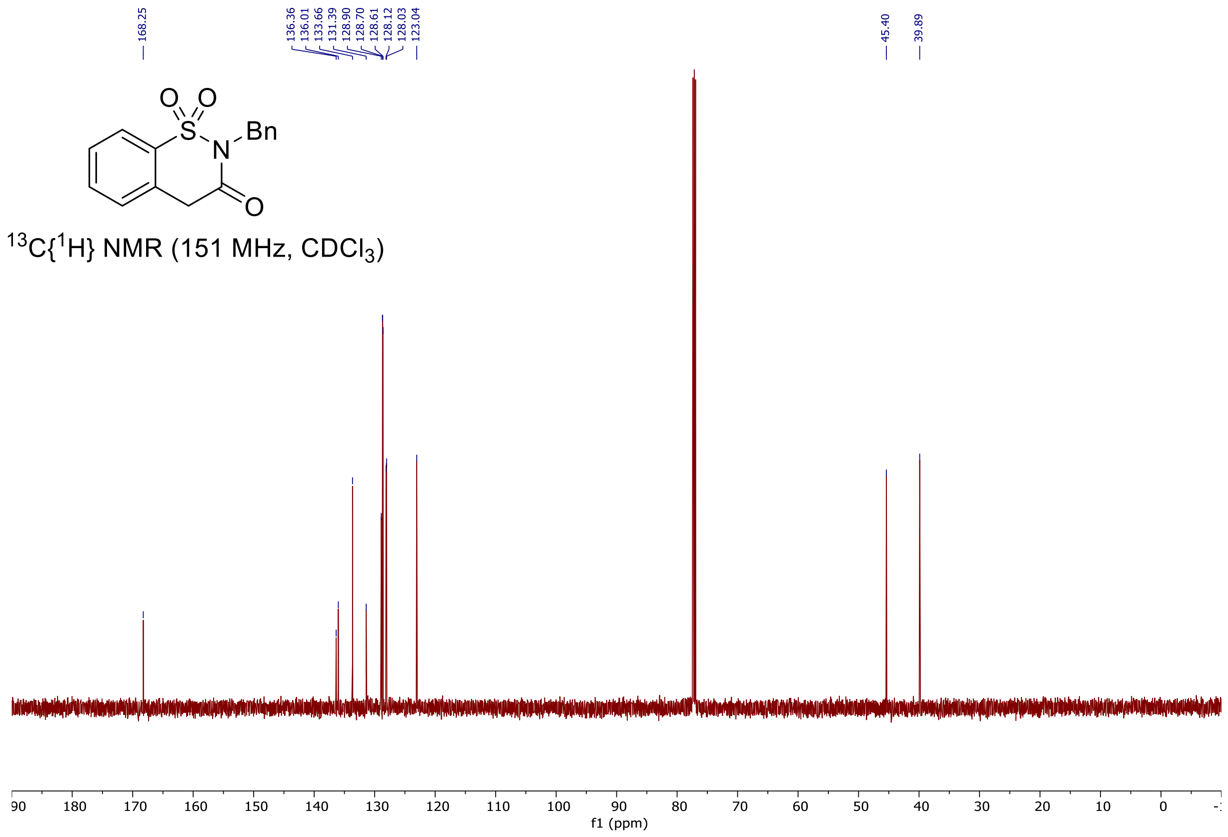

2-allyl-2*H*-benzo[*e*][1,2]thiazin-3(4*H*)-one 1,1-dioxide (**S17**)

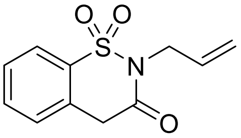

^{1}H NMR (500 MHz, $CDCl_3$)

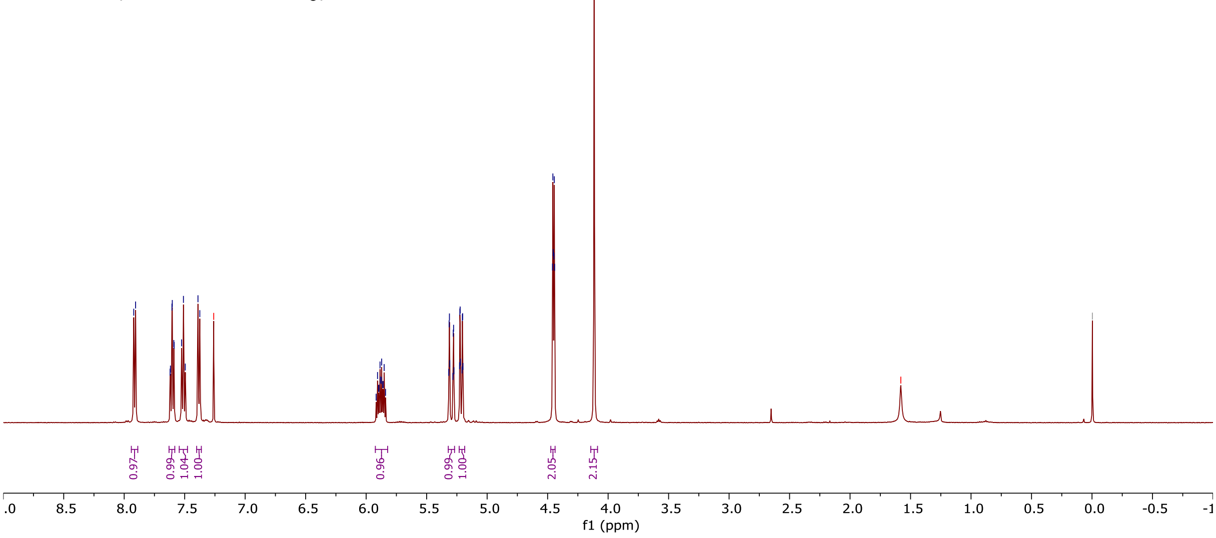

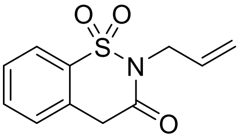

$^{13}C\{^1H\}$ NMR (126 MHz, $CDCl_3$)

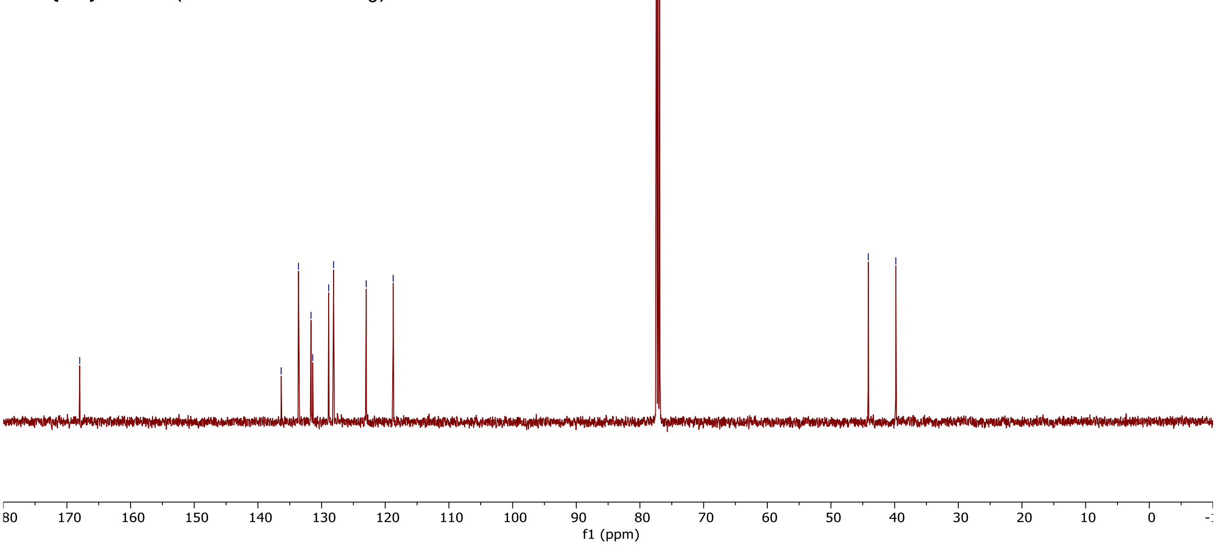

7-chloro-4-diazo-2-methylisoquinoline-1,3(2*H*,4*H*)-dione (**1g**)

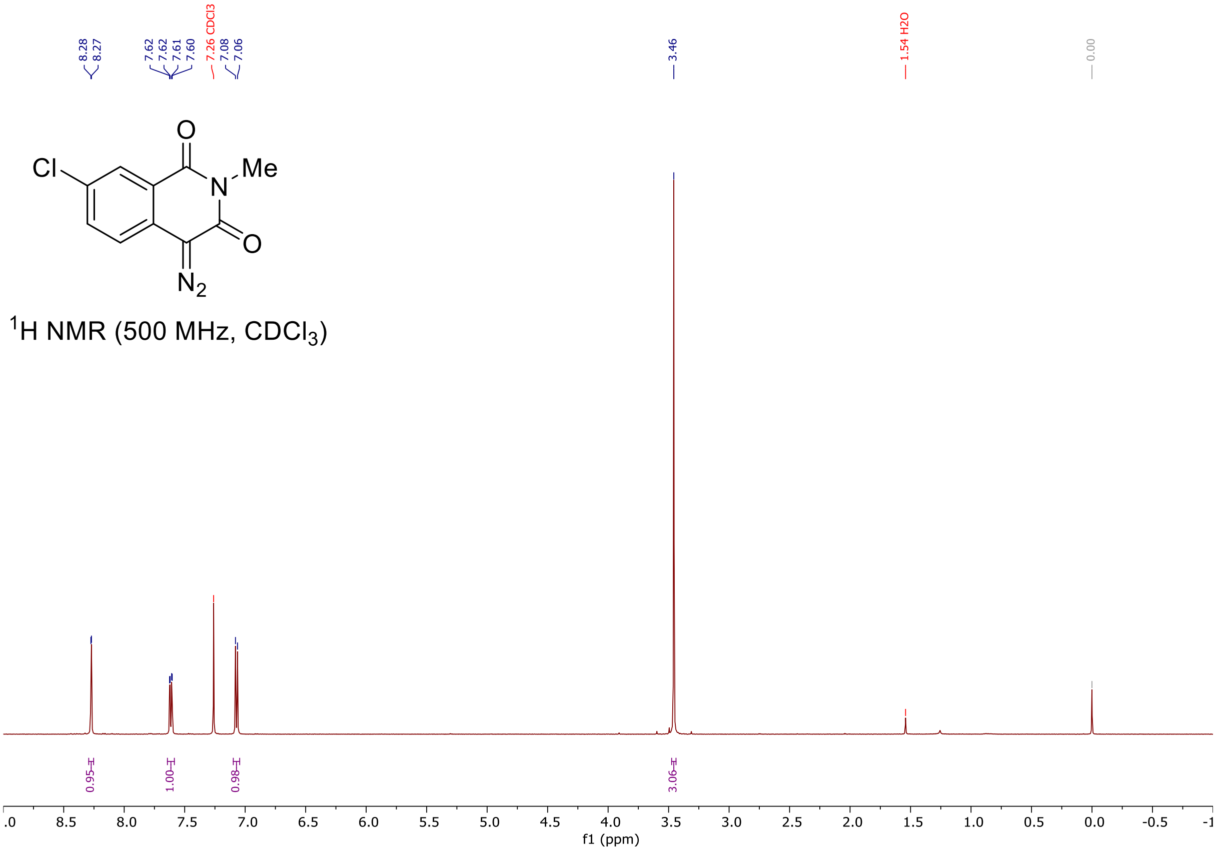

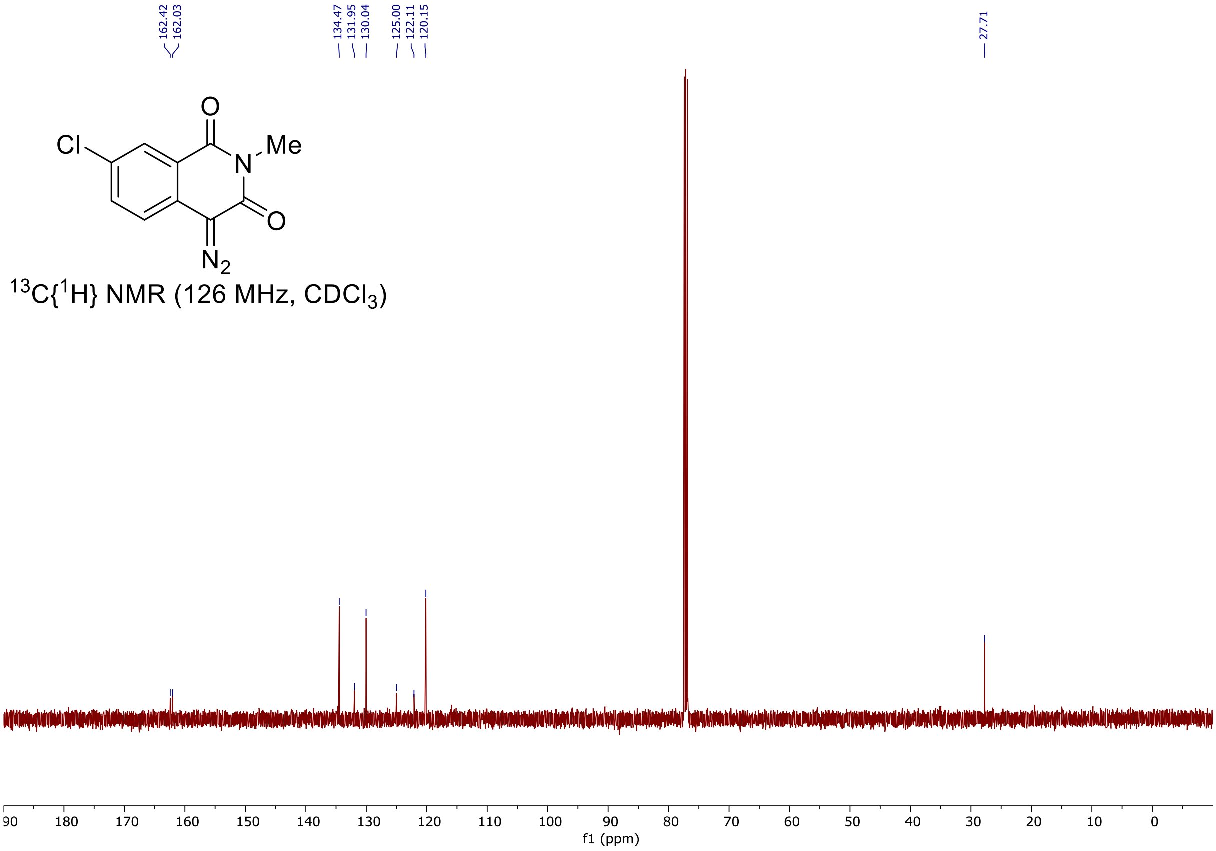

7-bromo-4-diazo-2-methylisoquinoline-1,3(2*H*,4*H*)-dione (**1h**)

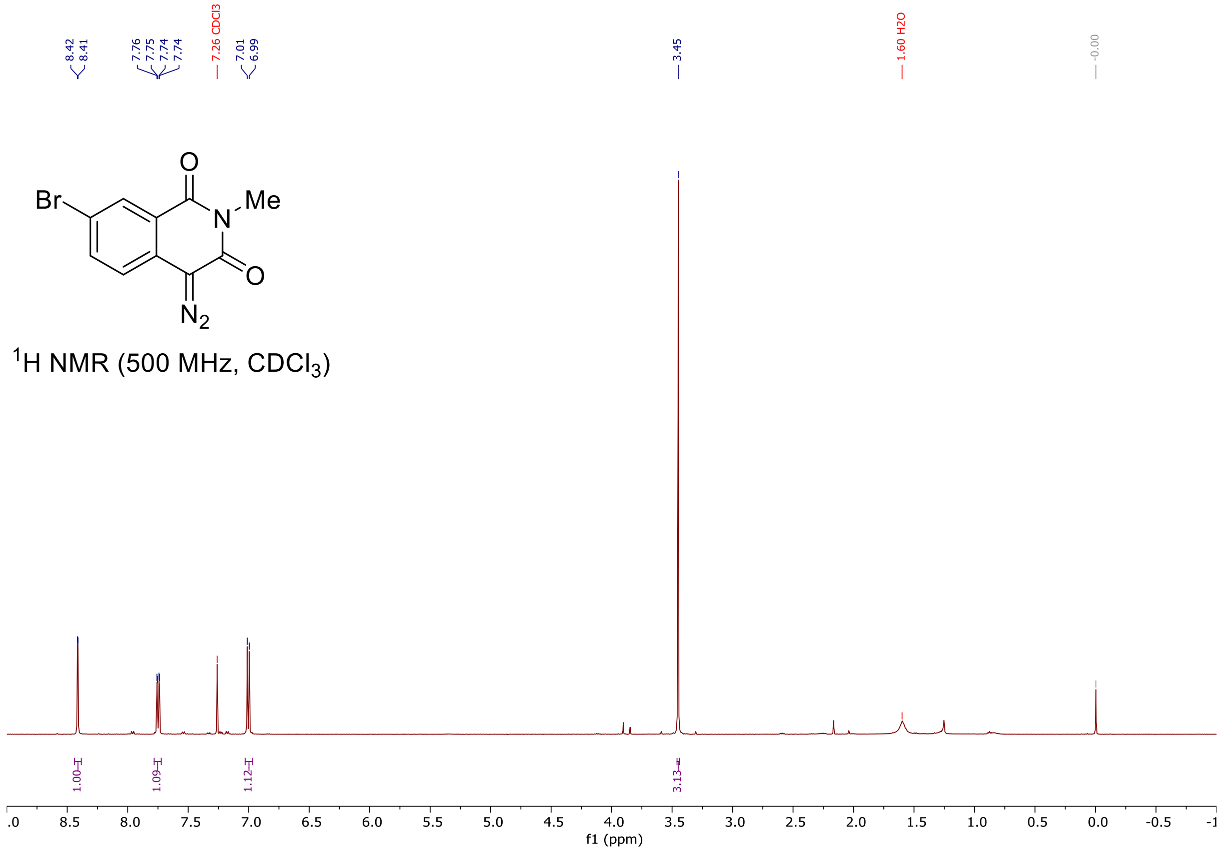

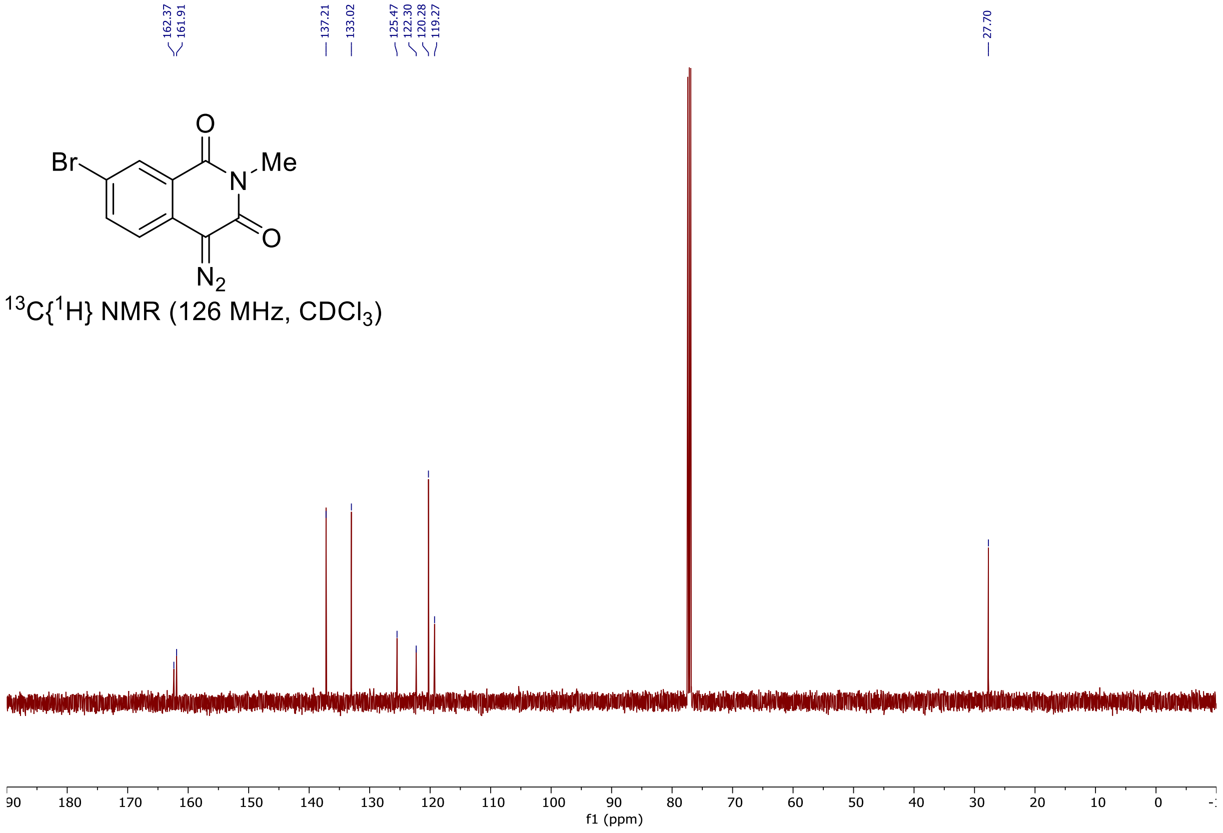

4-diazo-2,7-dimethylisoquinoline-1,3(2*H*,4*H*)-dione (**1i**)

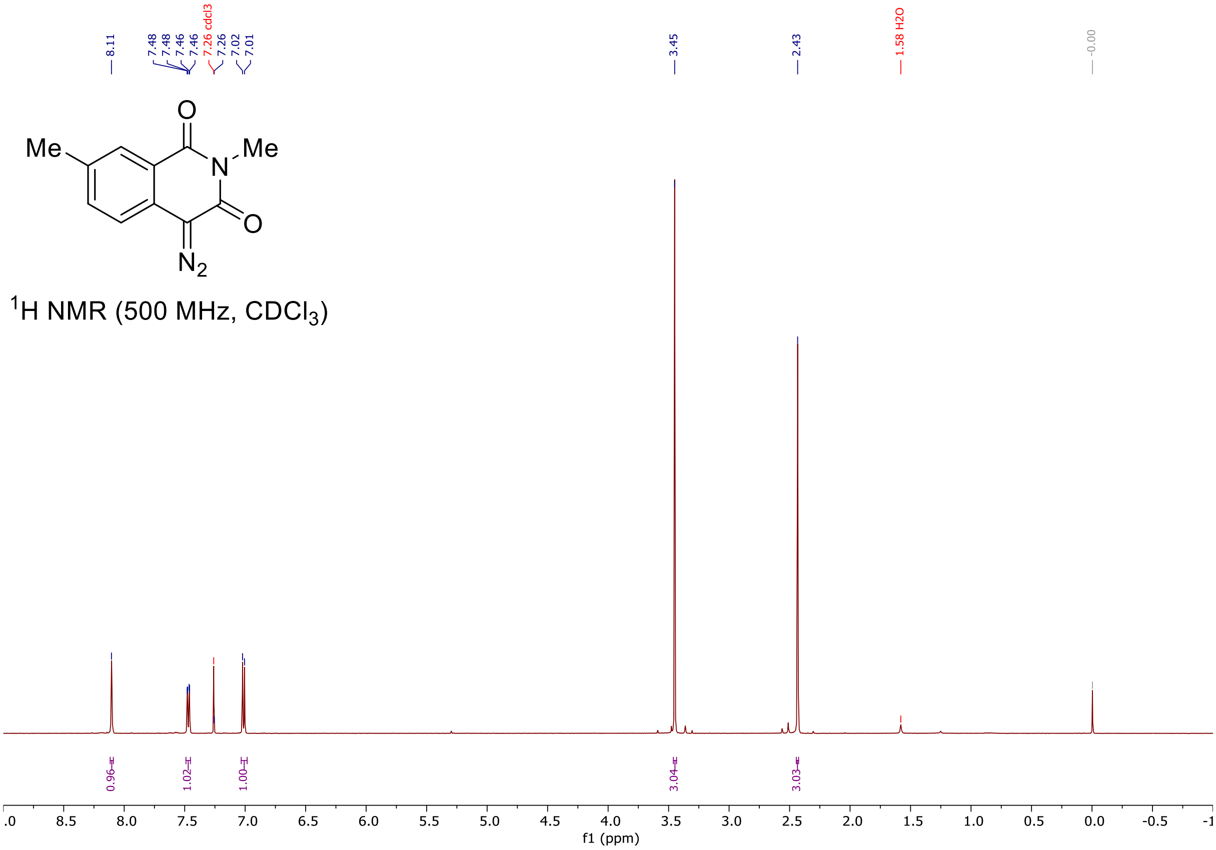

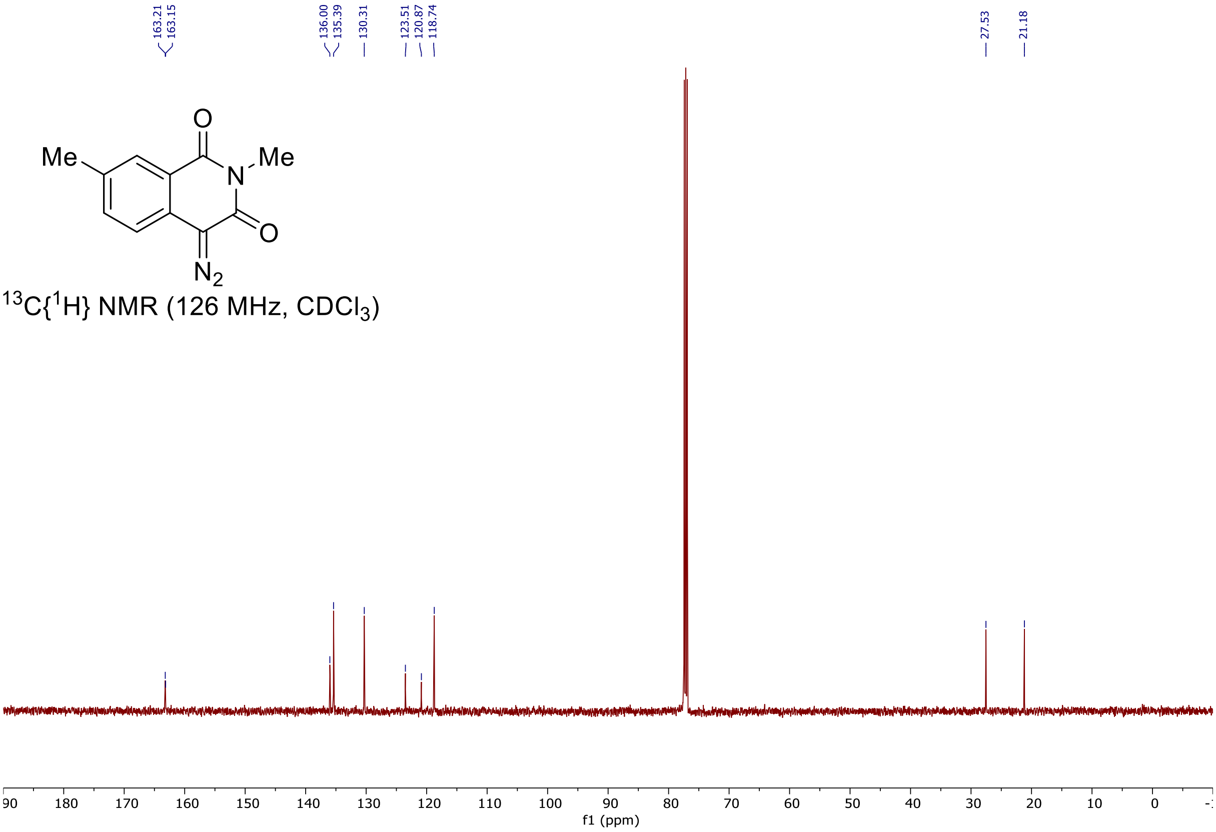

4-diazo-7-methoxy-2-methylisoquinoline-1,3(2*H*,4*H*)-dione (**1j**)

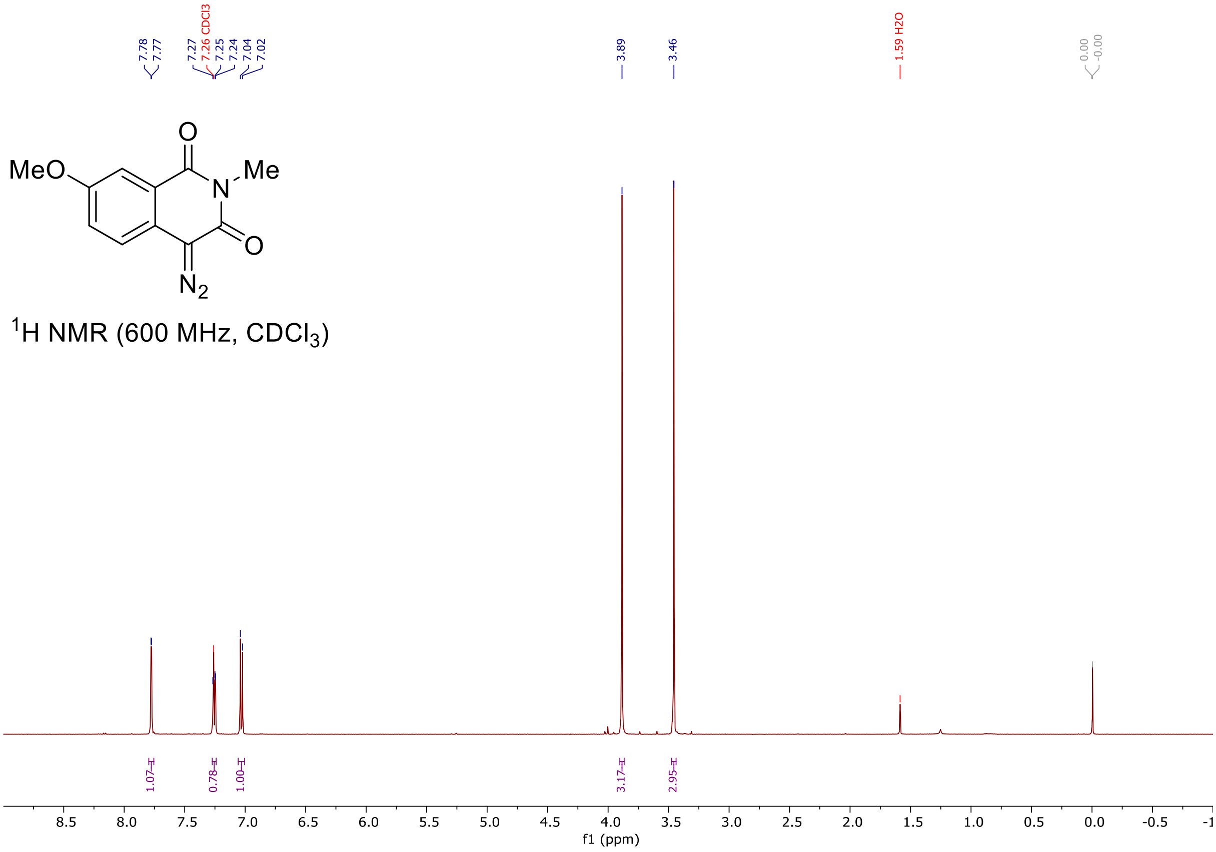

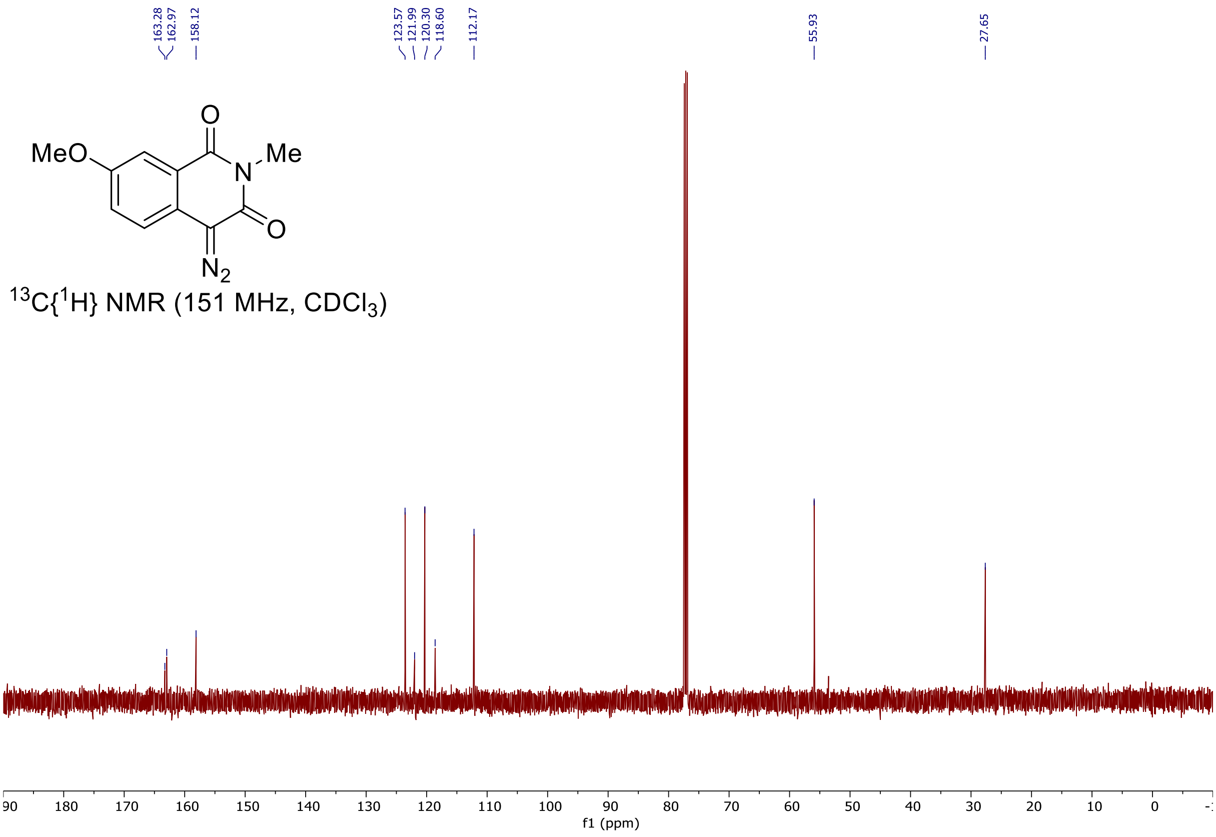

4-diazo-6-methoxy-2-methylisoquinoline-1,3(2*H*,4*H*)-dione (**1k**)

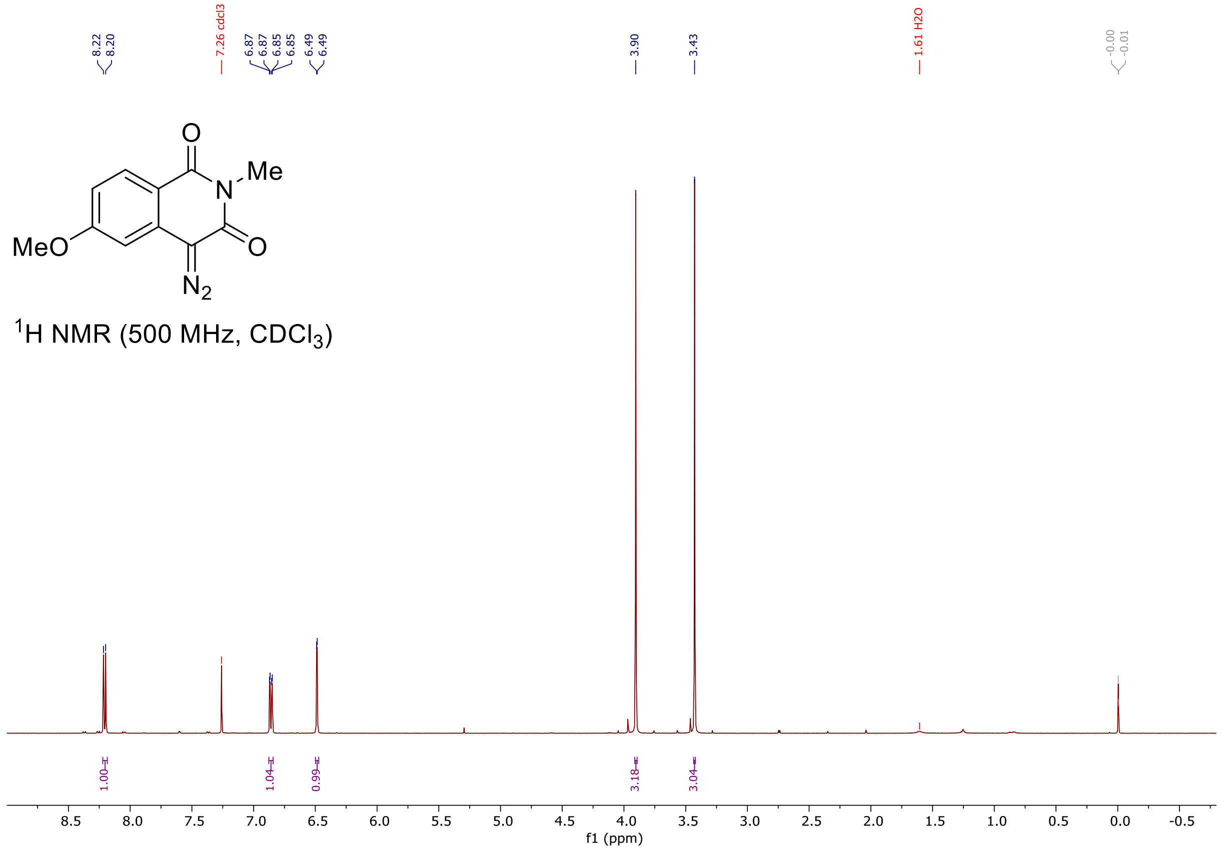

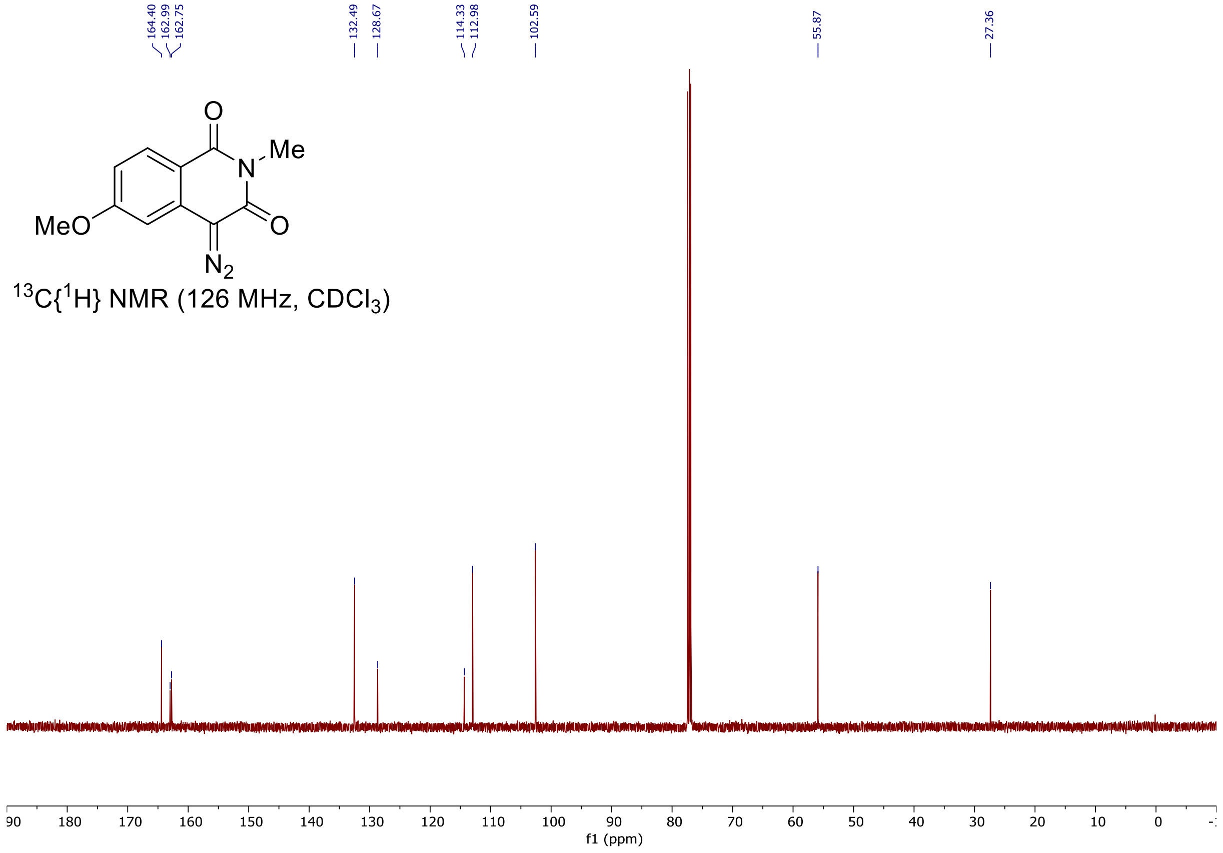

6-chloro-4-diazo-2-methylisoquinoline-1,3(2*H*,4*H*)-dione (**1l**)

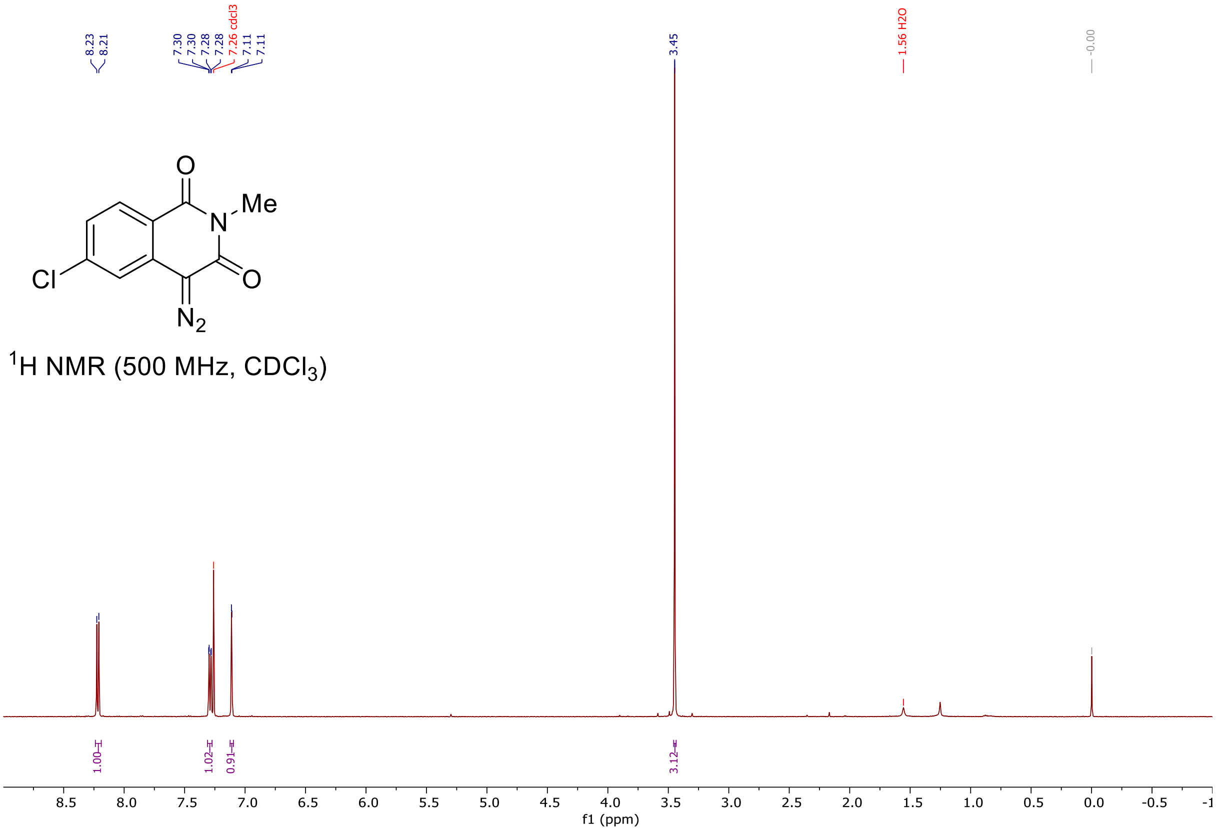

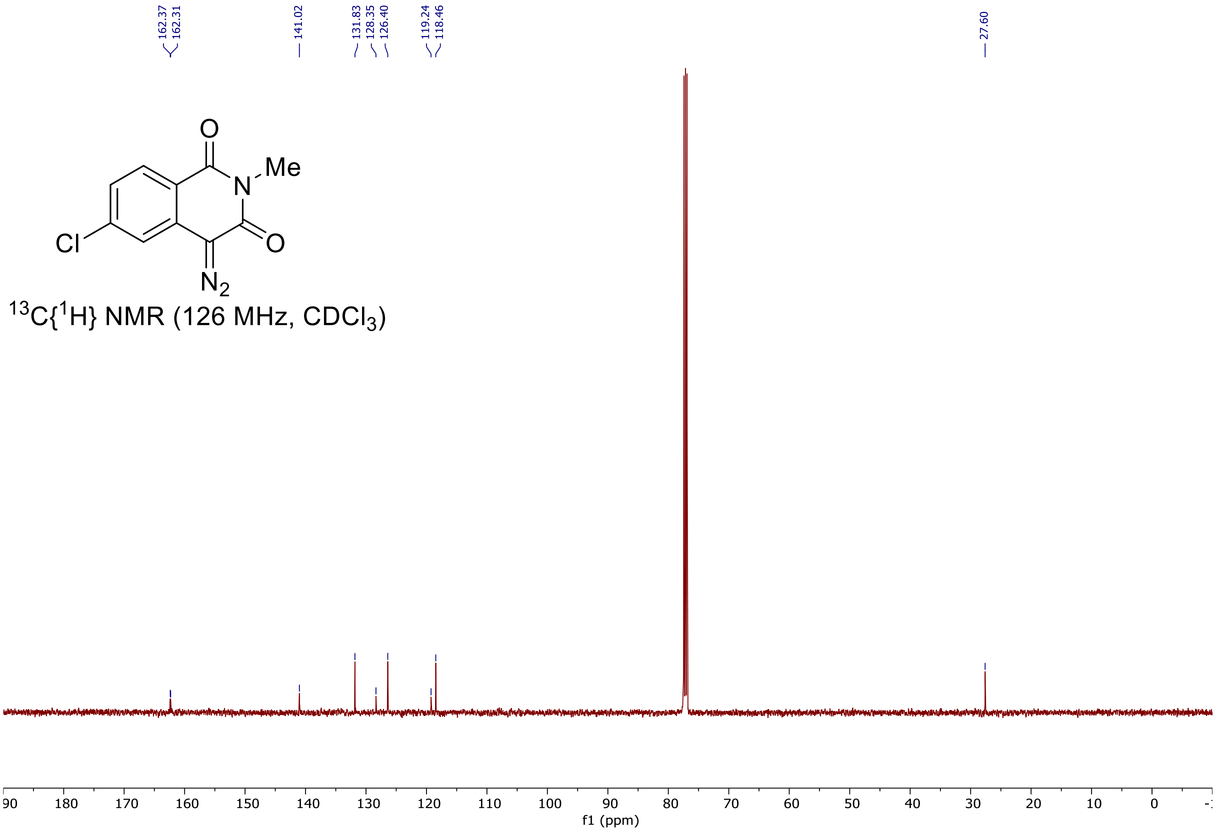

6-bromo-4-diazo-2-methylisoquinoline-1,3(2*H*,4*H*)-dione (**1m**)

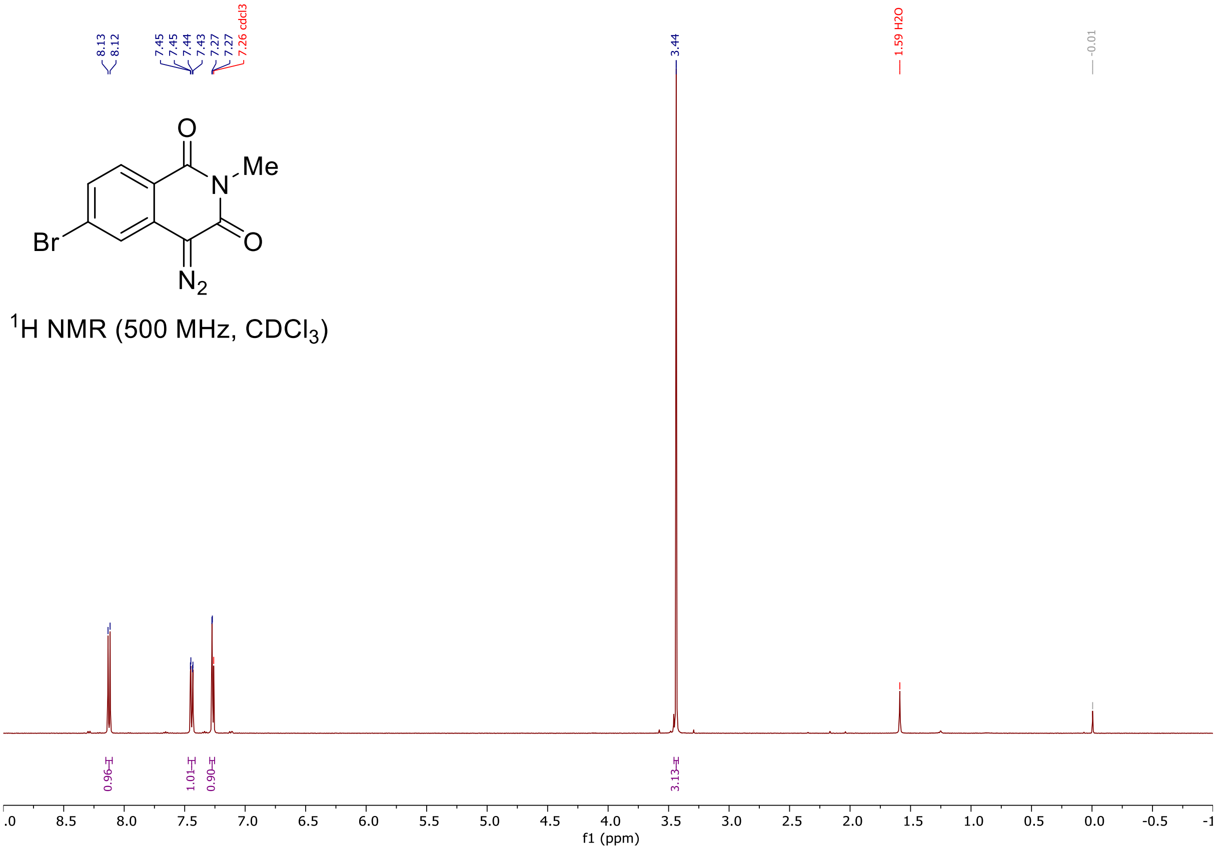

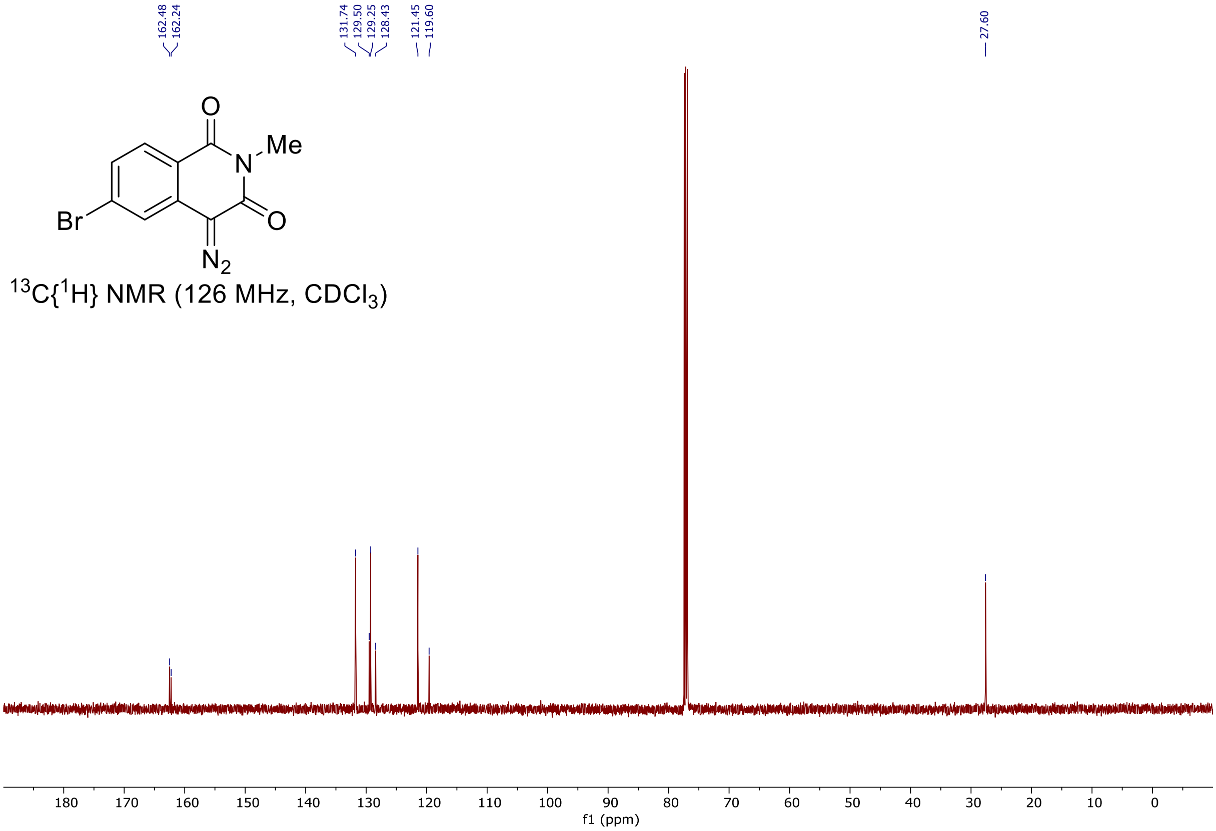

8-chloro-4-diazo-2-methylisoquinoline-1,3(2*H*,4*H*)-dione (**1n**)

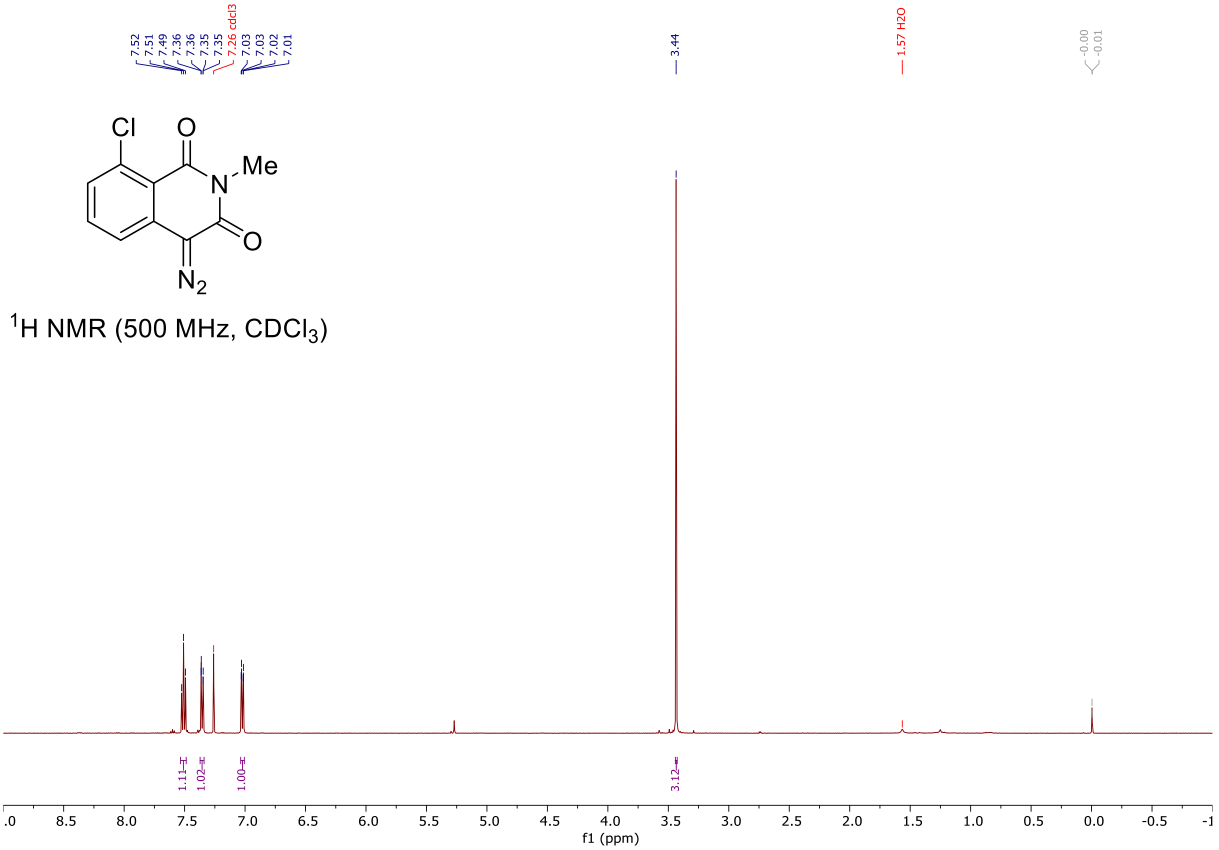

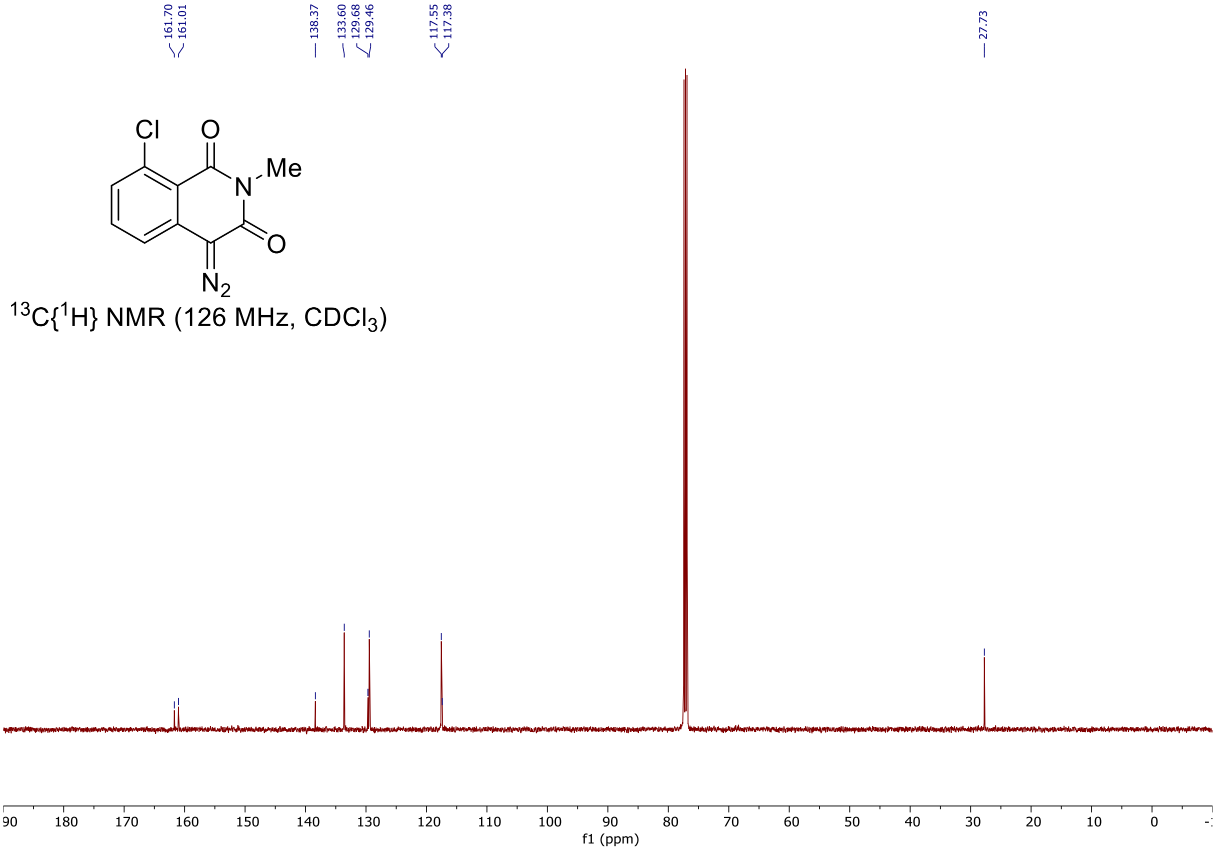

5-chloro-4-diazo-2-methylisoquinoline-1,3(2*H*,4*H*)-dione (**1o**)

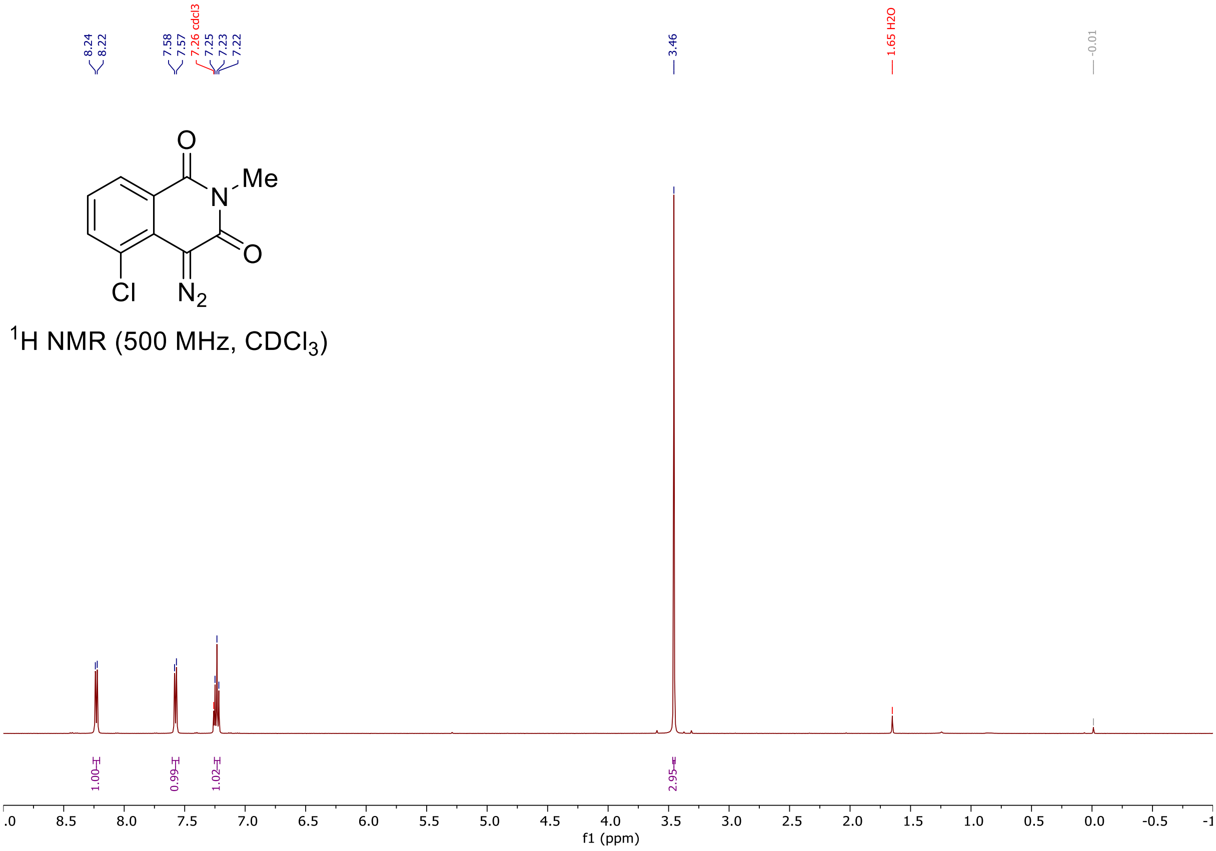

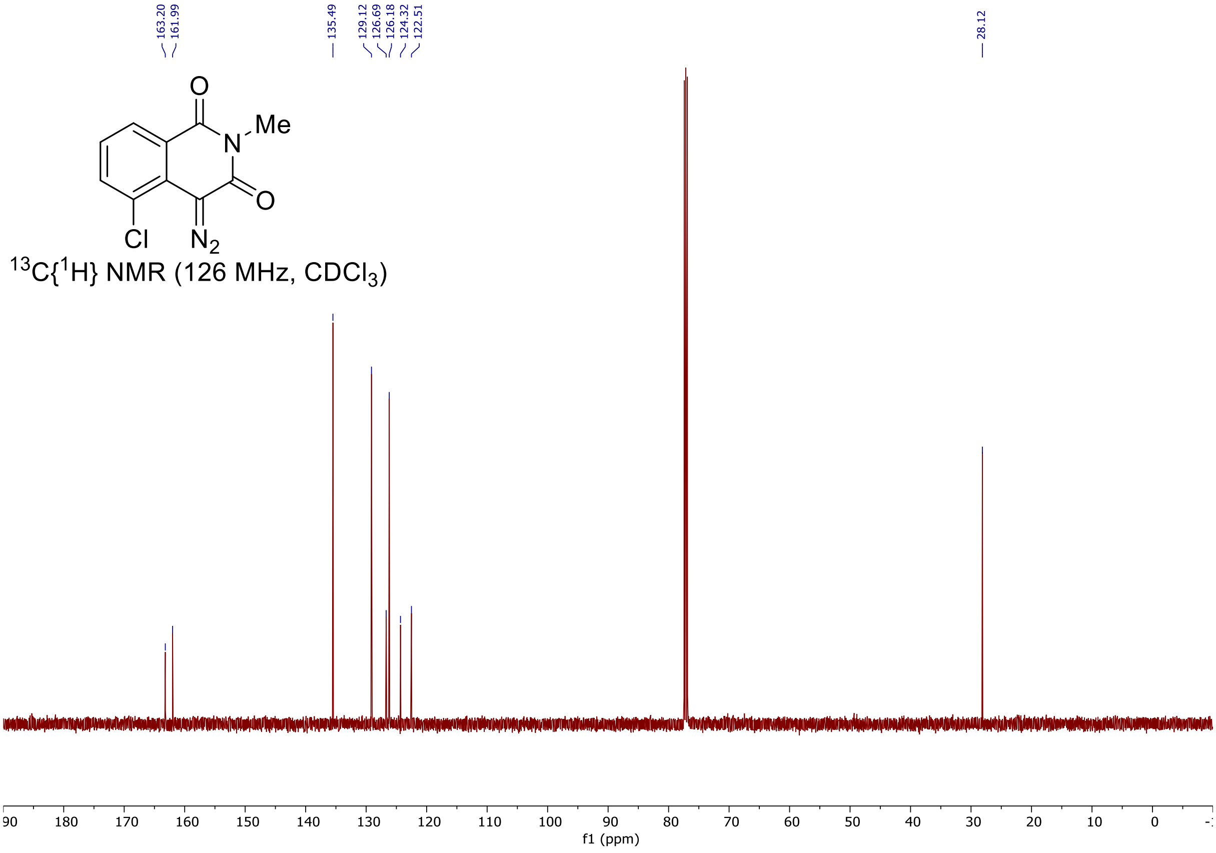

2-methyl-4-diazo-2H-benzo[*e*][1,2]thiazin-3(4*H*)-one 1,1-dioxide (**34a**)

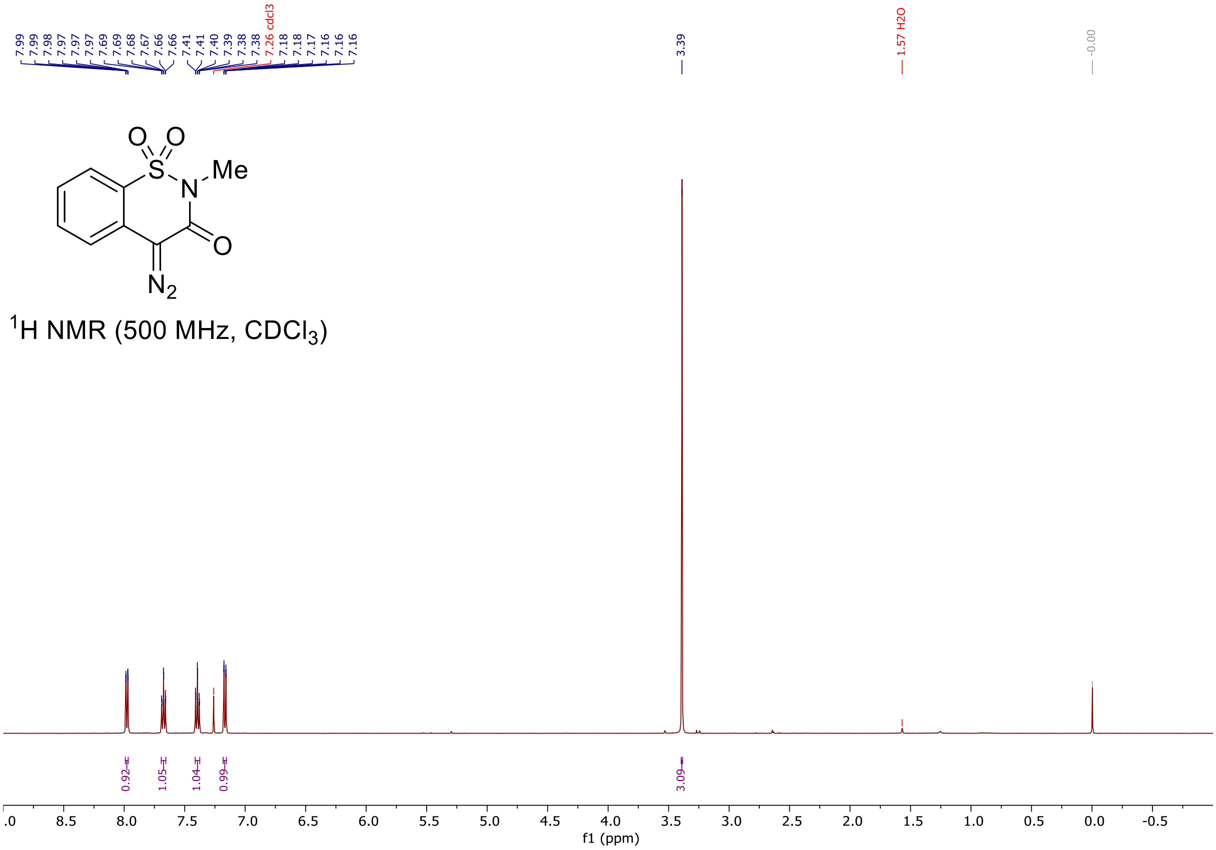

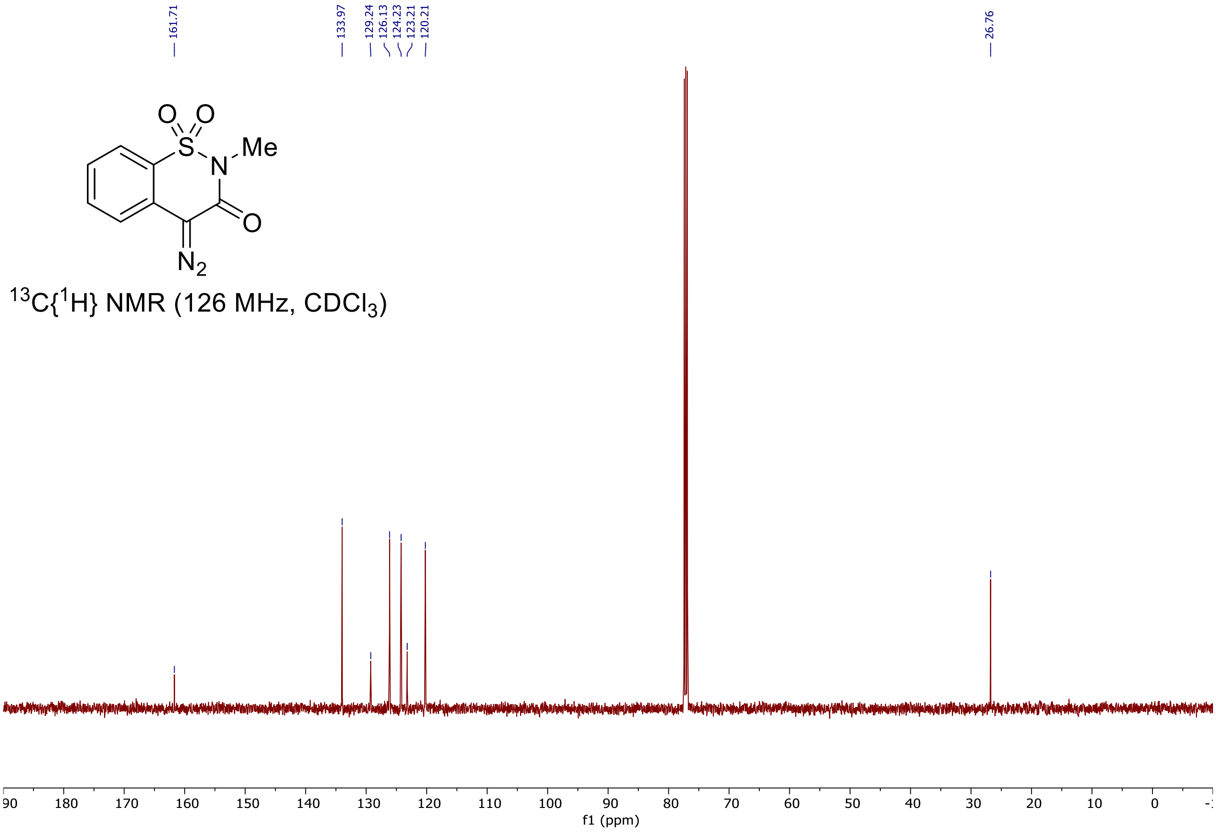

2-benzyl-4-diazo-2H-benzo[*e*][1,2]thiazin-3(4*H*)-one 1,1-dioxide (**34b**)

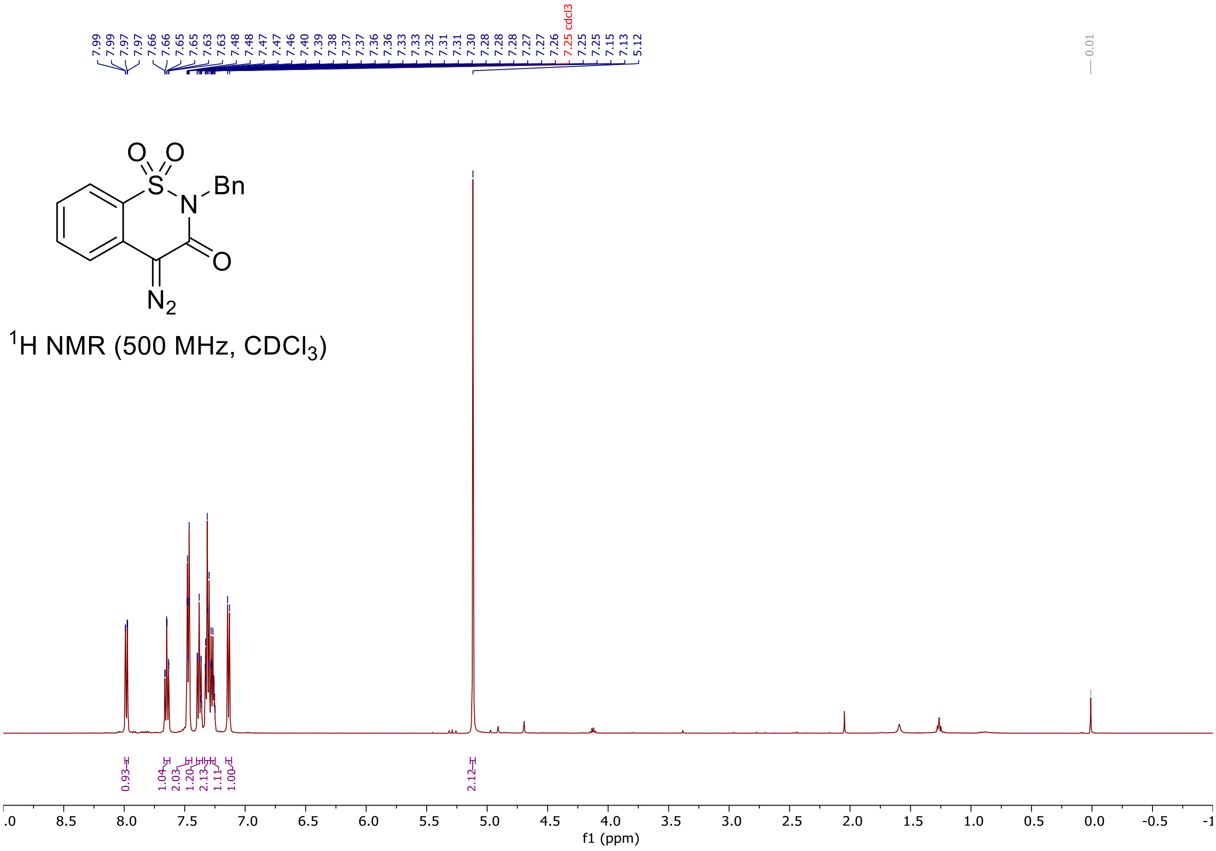

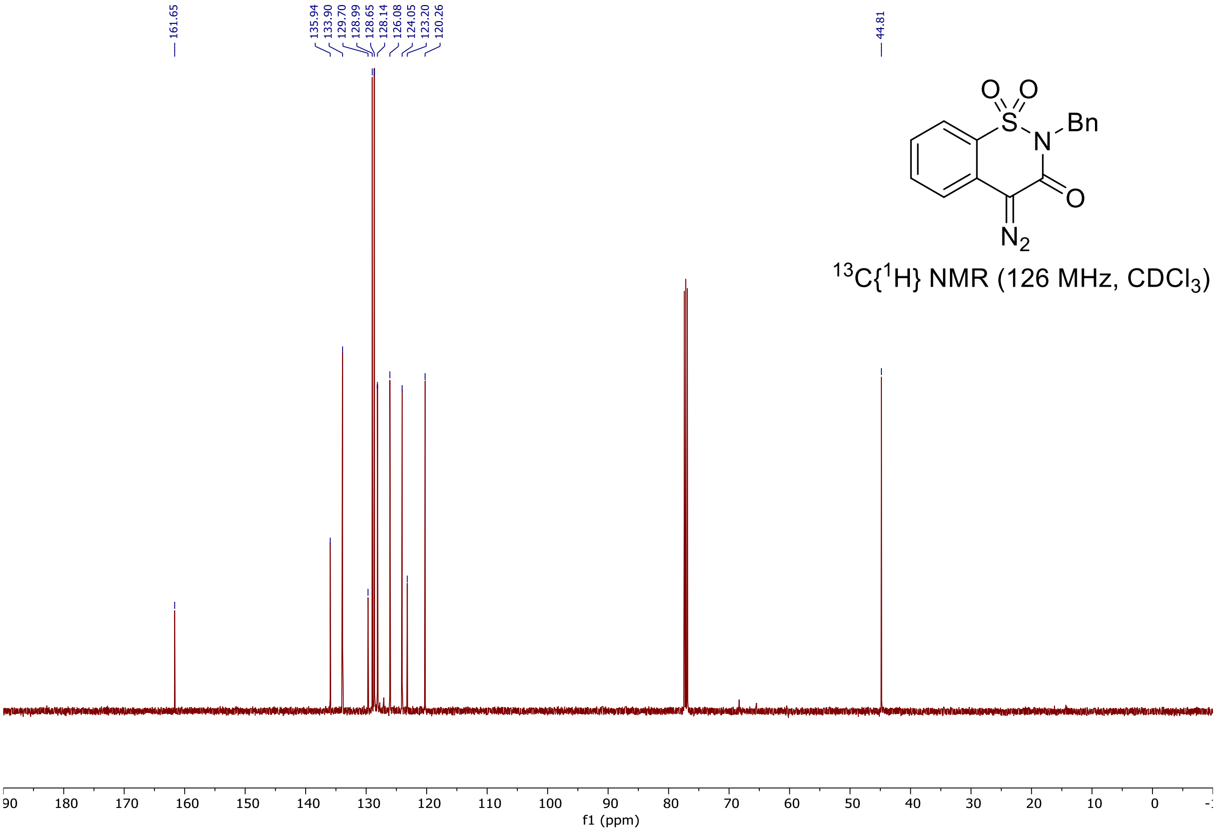

4-diazo-2-allyl-2*H*-benzo[*e*][1,2]thiazin-3(4*H*)-one 1,1-dioxide (**34c**)

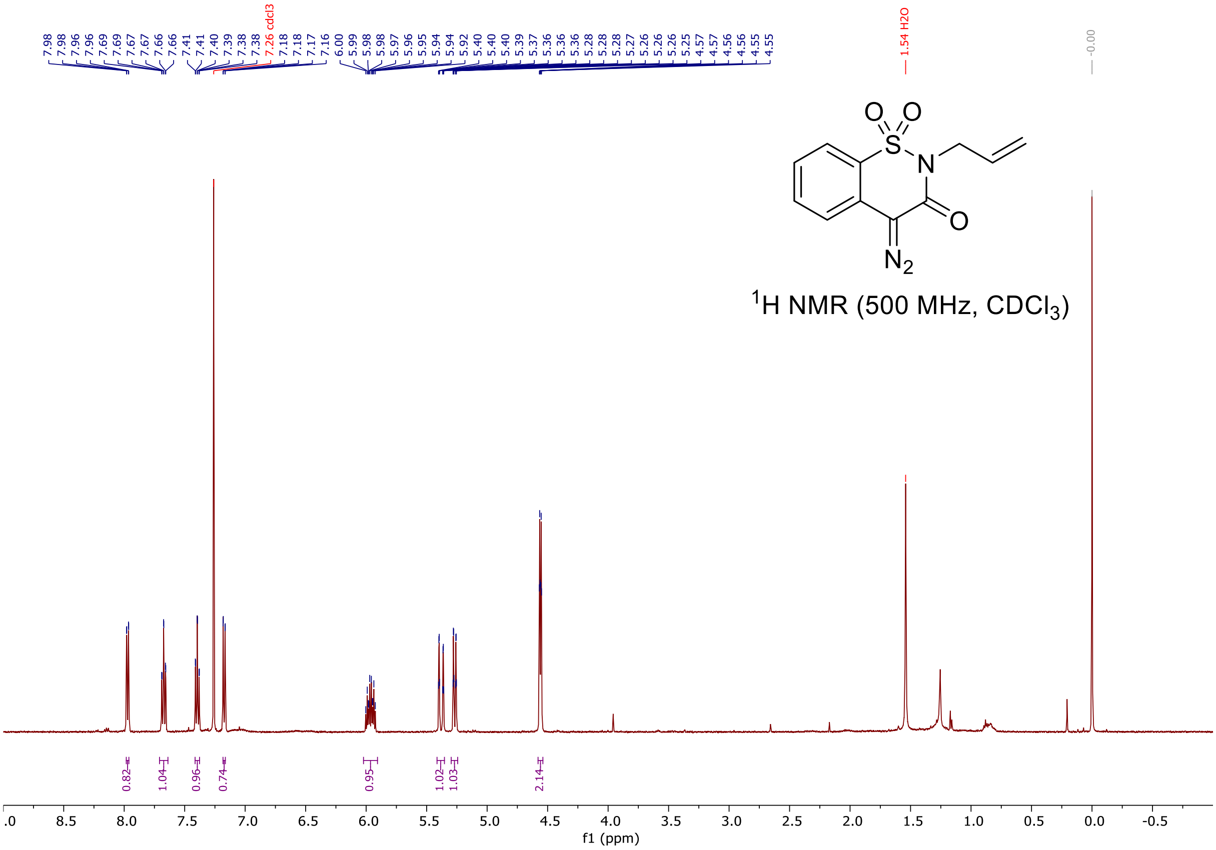

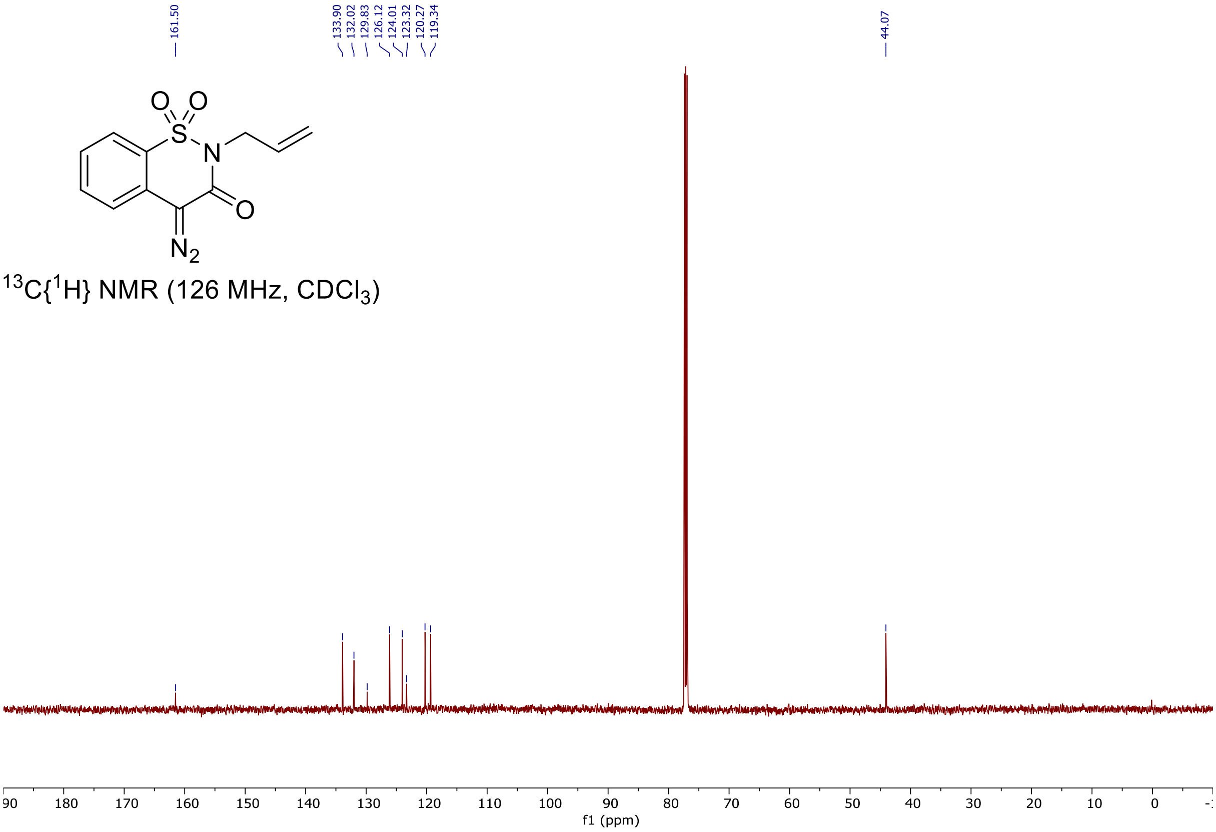

4-((1,1,1,3,3,3-hexafluoropropan-2-yl)oxy)-2-methylisoquinoline-1,3(2*H*,4*H*)-dione (**2**)

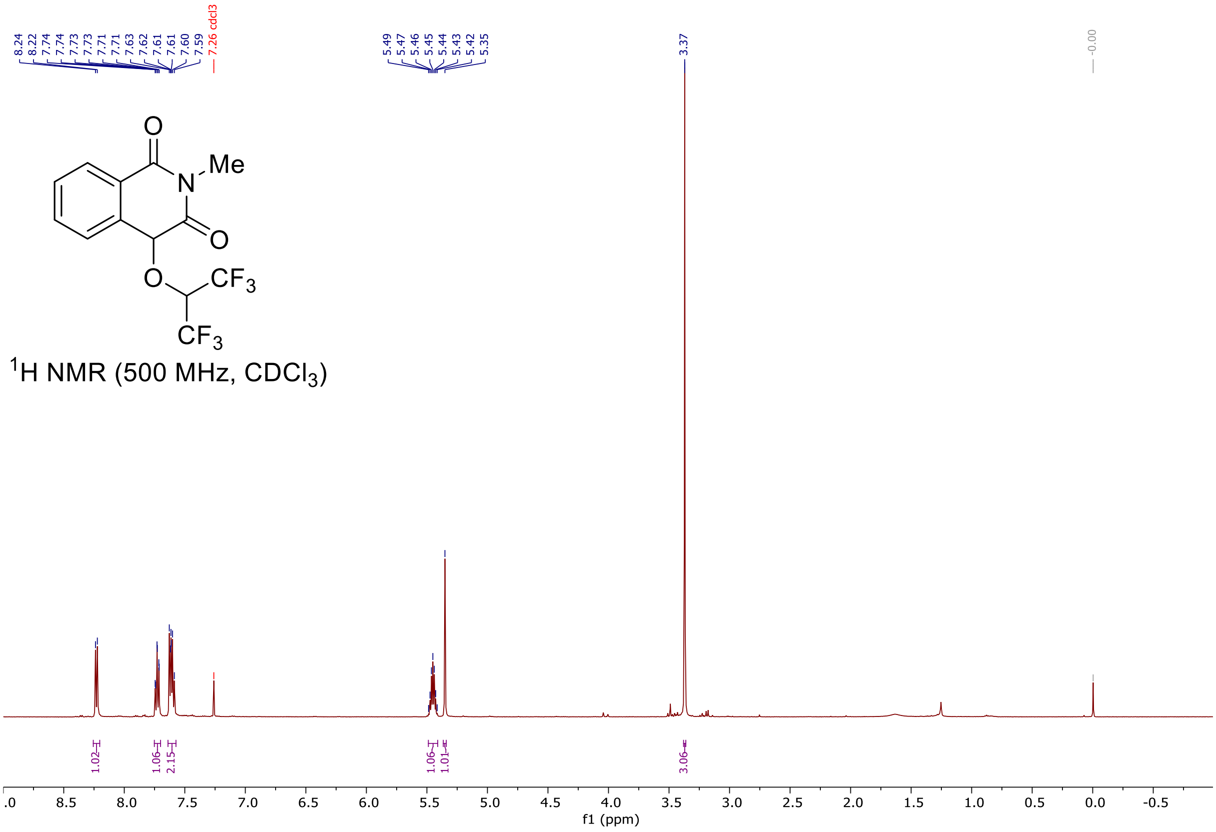

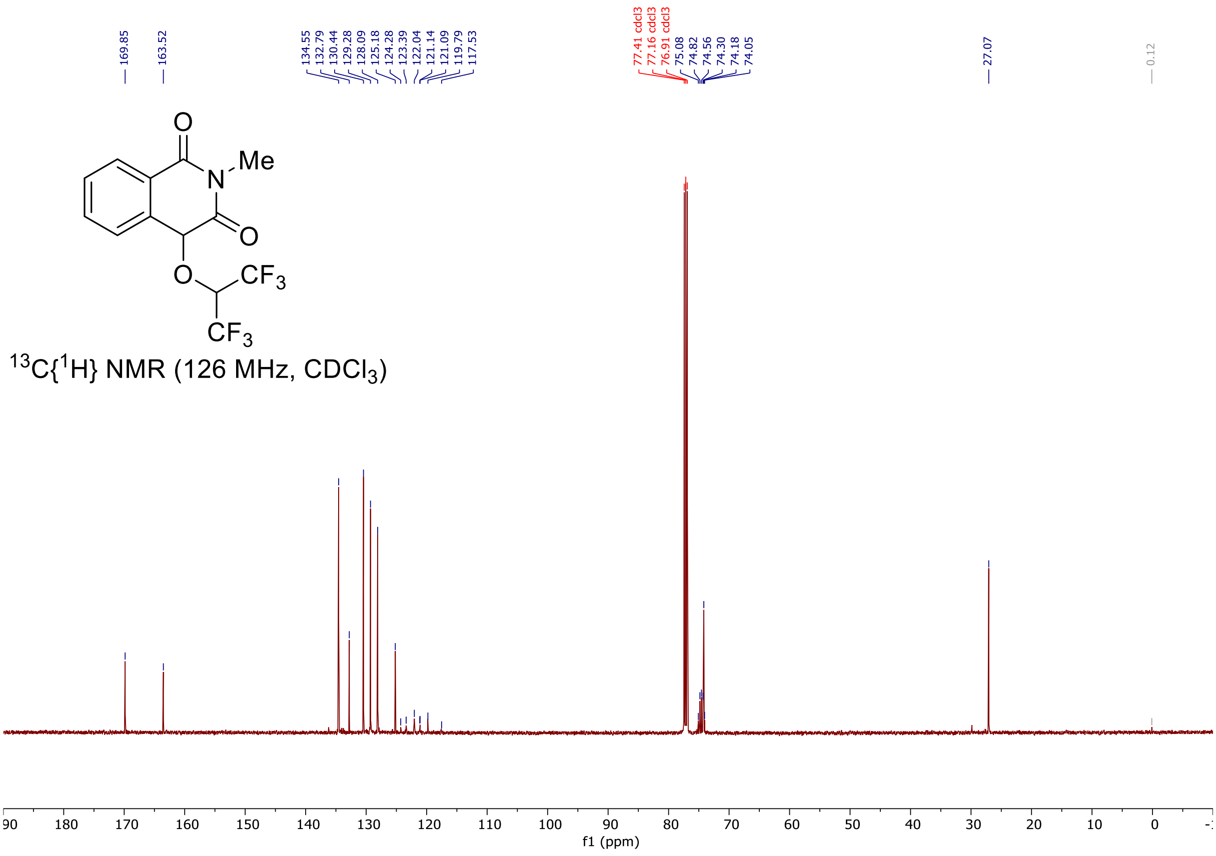

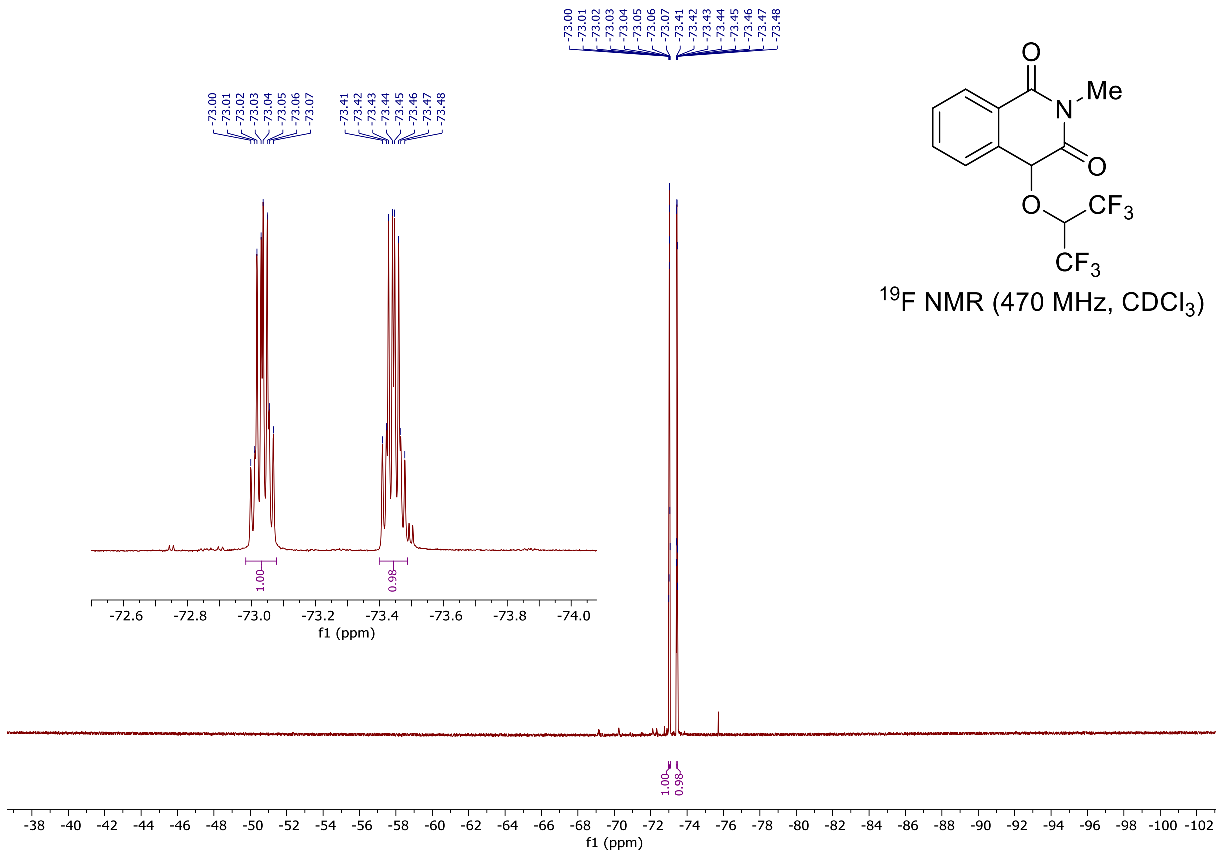

2-ethyl-4-((1,1,1,3,3,3-hexafluoropropan-2-yl)oxy)isoquinoline-1,3(2*H*,4*H*)-dione (**3**)

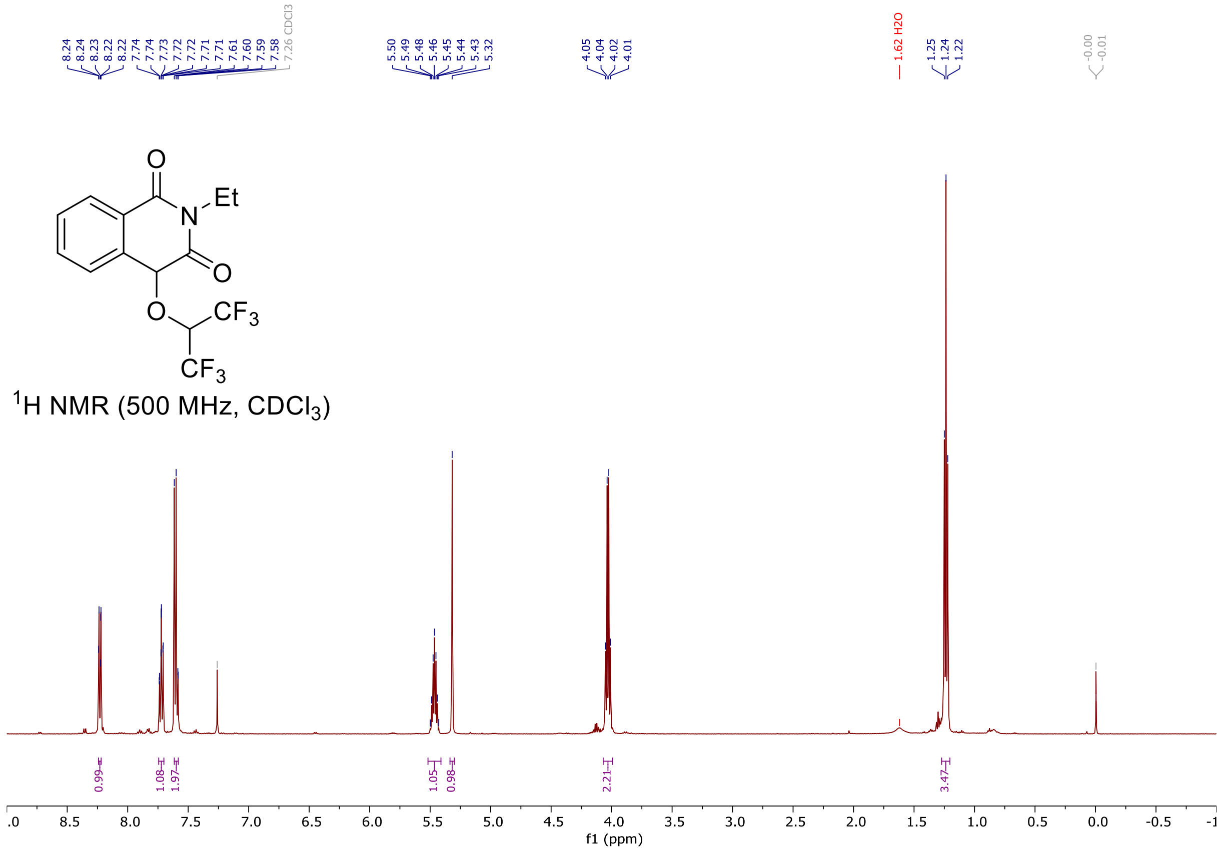

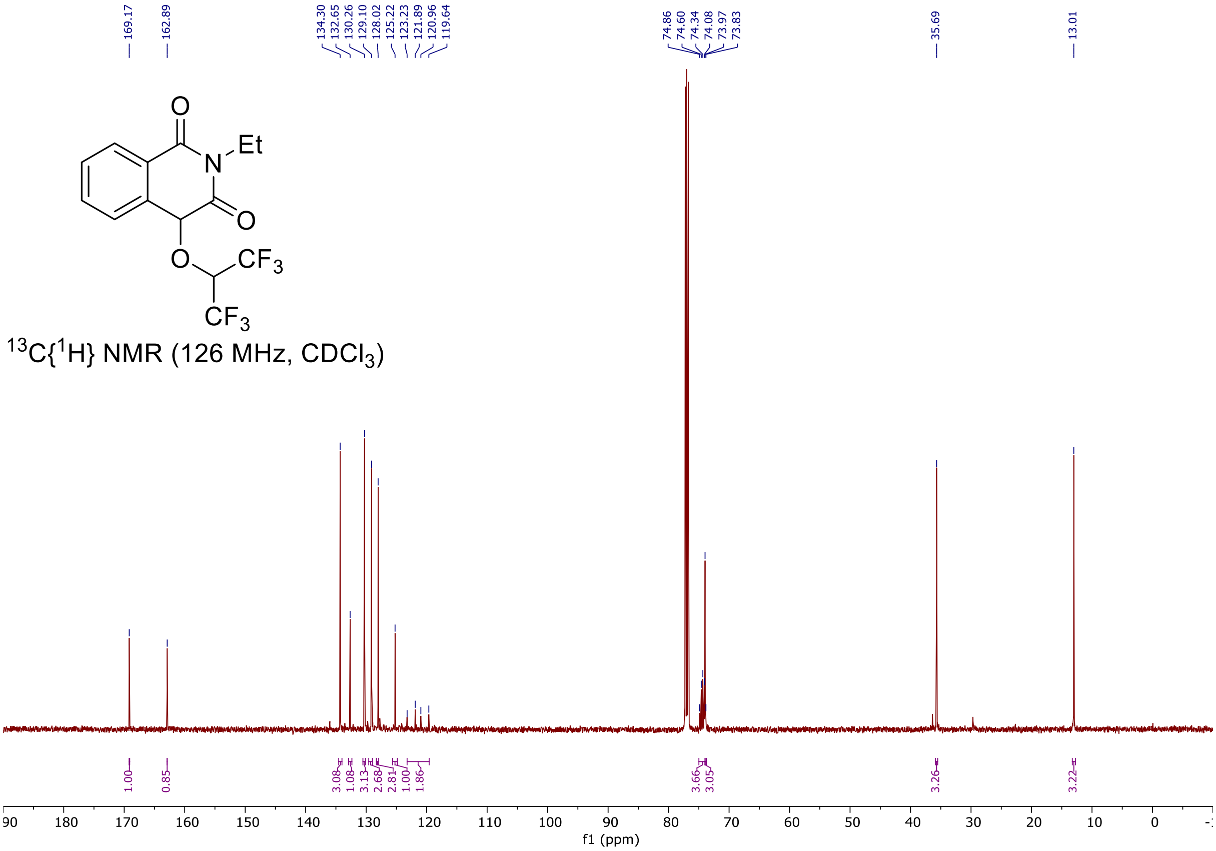
13C{1H} NMR (126 MHz, CDCl3)
f1 (ppm)

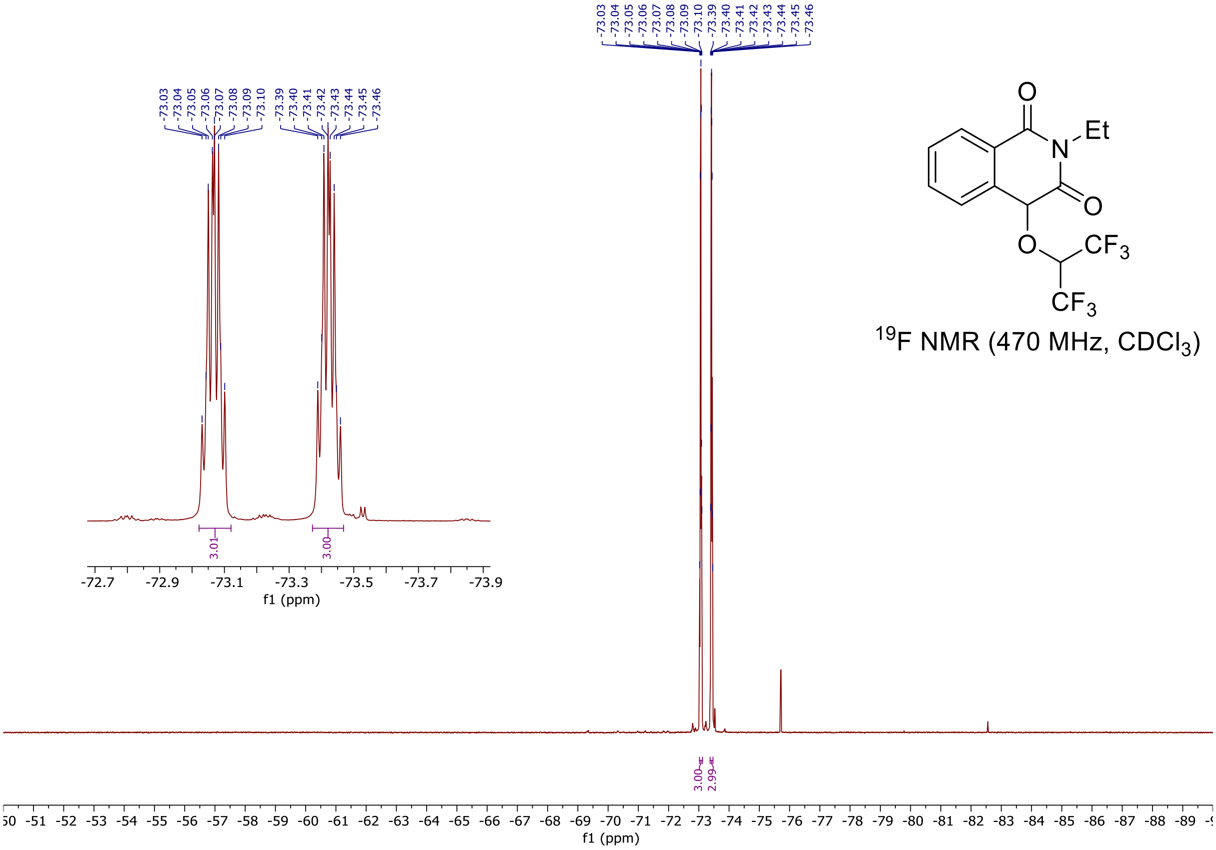
19F NMR (470 MHz, CDCl3)
f1 (ppm)

4-((1,1,1,3,3,3-hexafluoropropan-2-yl)oxy)-2-propylisoquinoline-1,3(2*H*,4*H*)-dione (**4**)

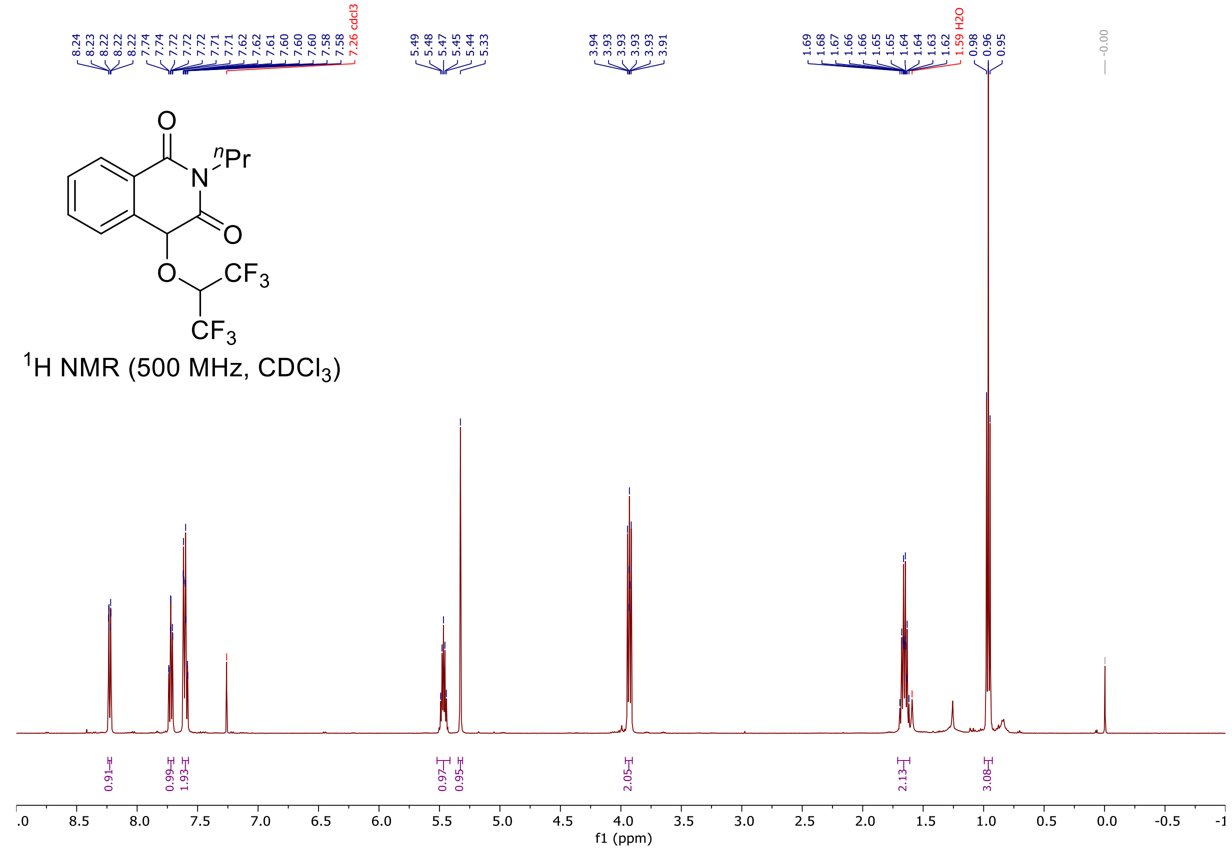

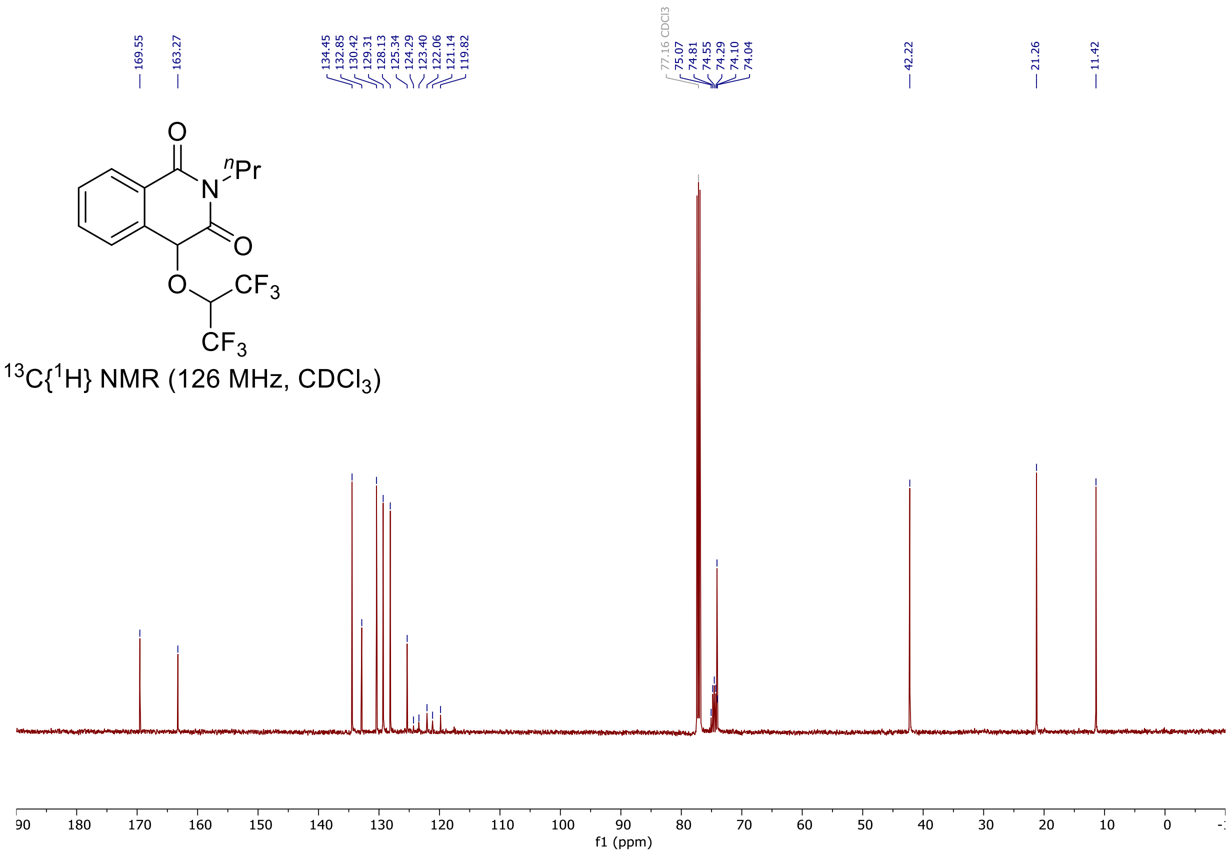

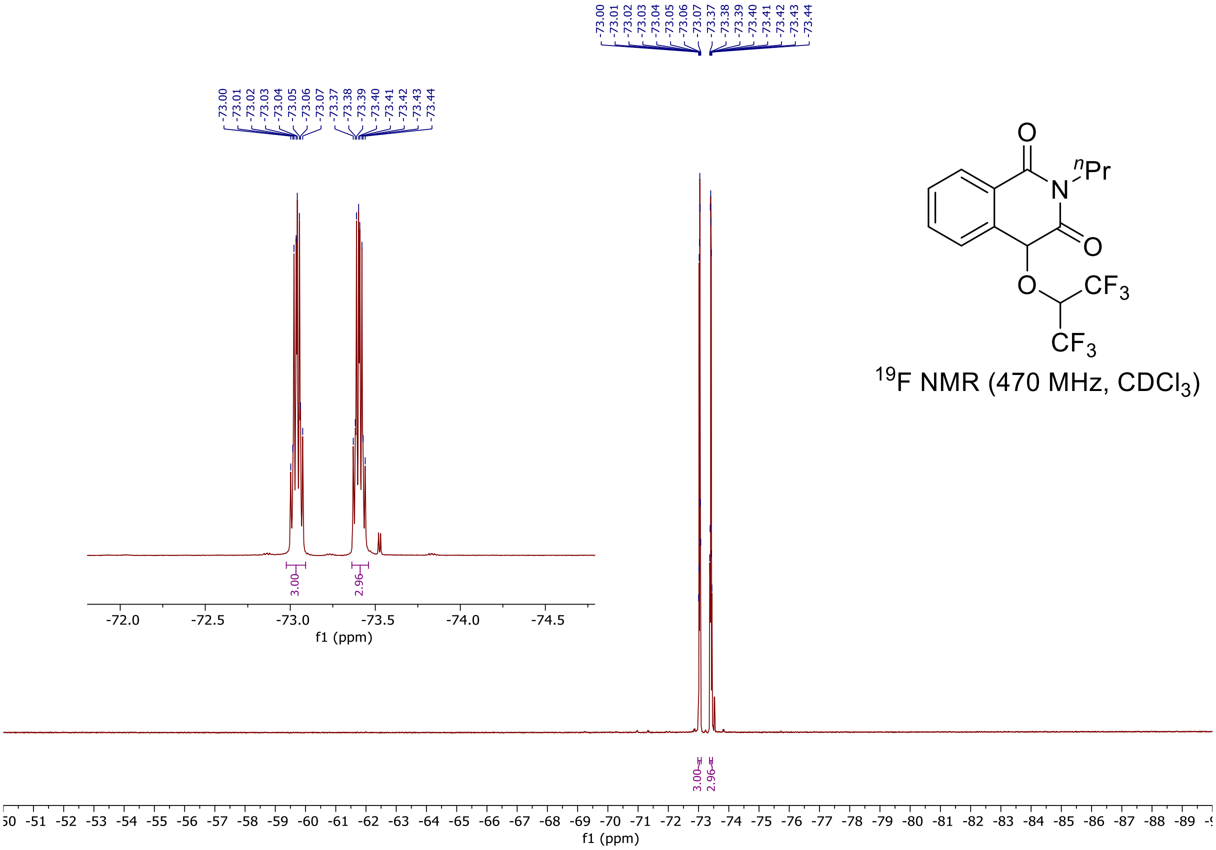

4-((1,1,1,3,3,3-hexafluoropropan-2-yl)oxy)-2-isopropylisoquinoline-1,3(2*H*,4*H*)-dione (**5**)

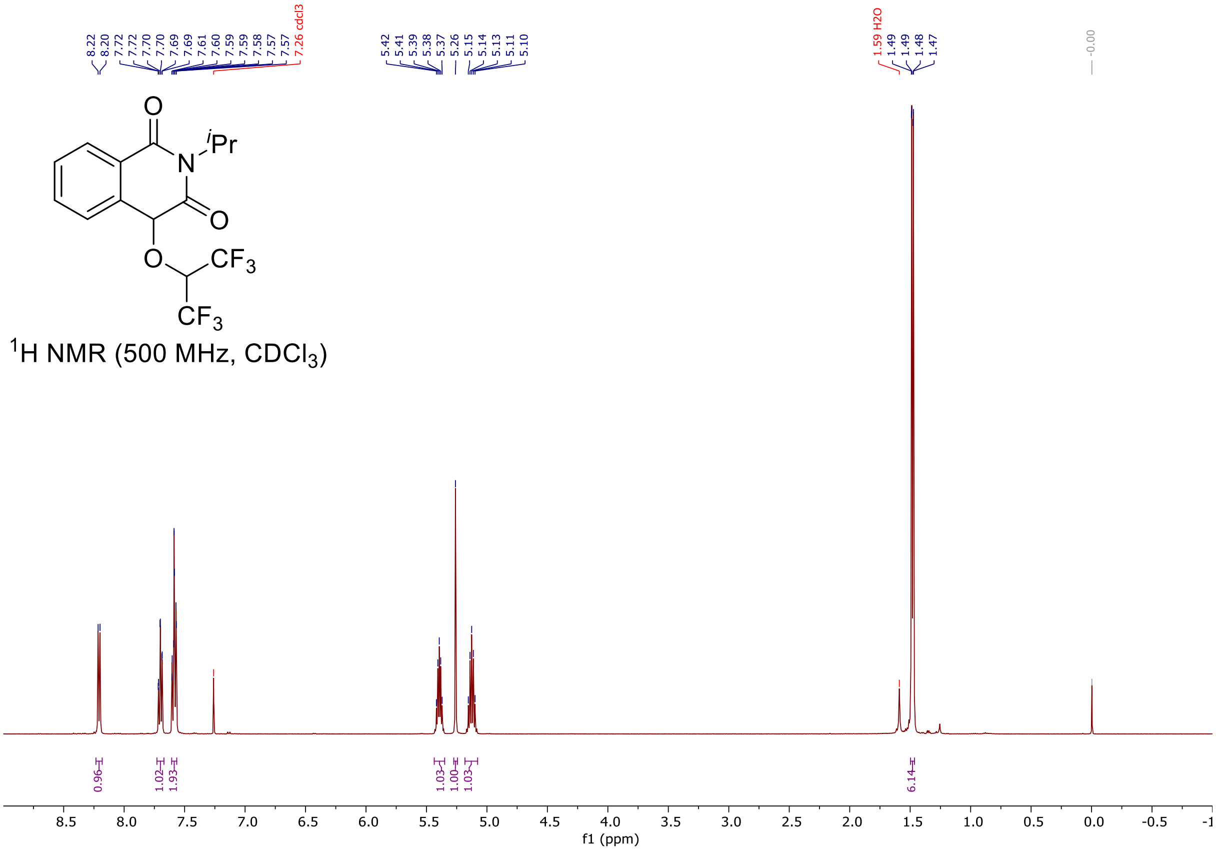

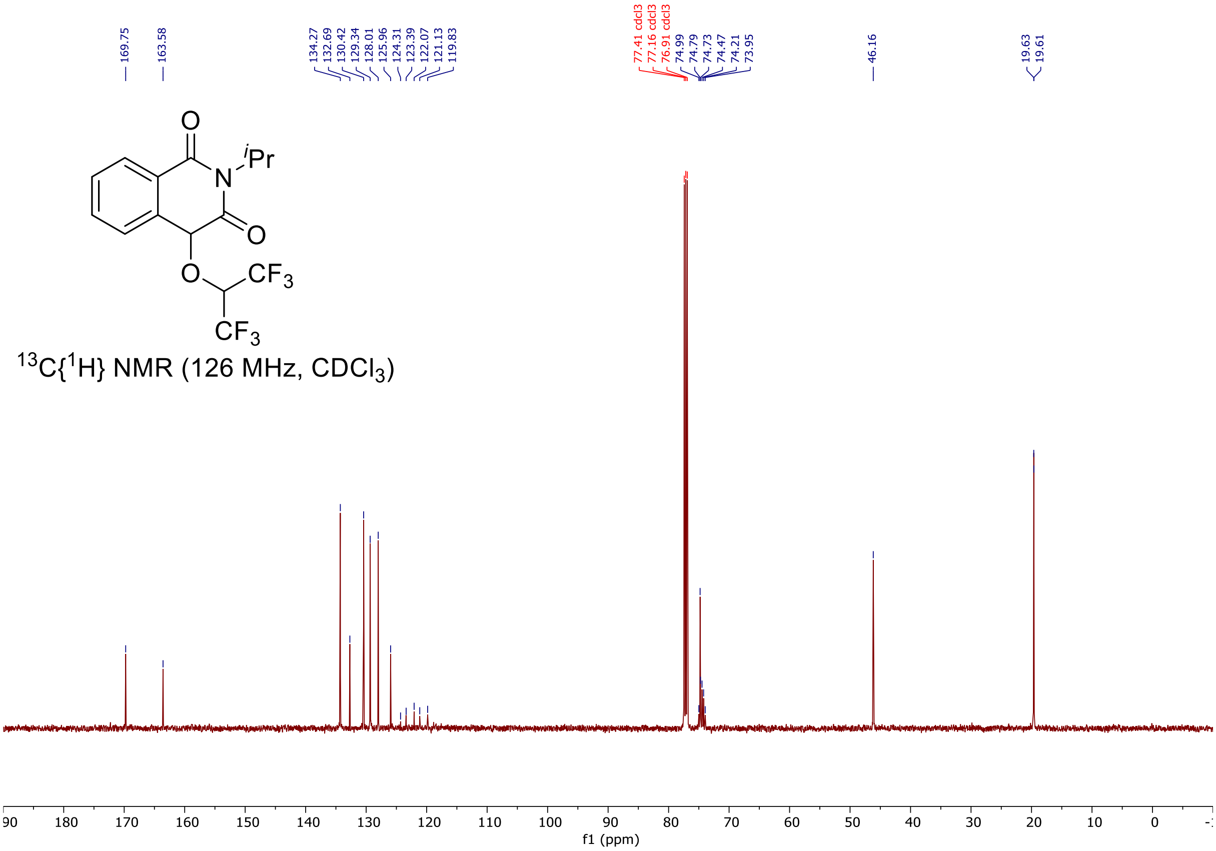
169.75
163.58
134.27
132.69
130.42
129.34
128.01
125.96
124.31
123.39
122.07
121.13
119.83
77.41 cdcl3
77.16 cdcl3
76.91 cdcl3
74.99
74.79
74.73
74.47
74.21
73.95
46.16
19.63
19.61
$^{13}C\{^{1}H\}$ NMR (126 MHz, $CDCl_3$)
f1 (ppm)

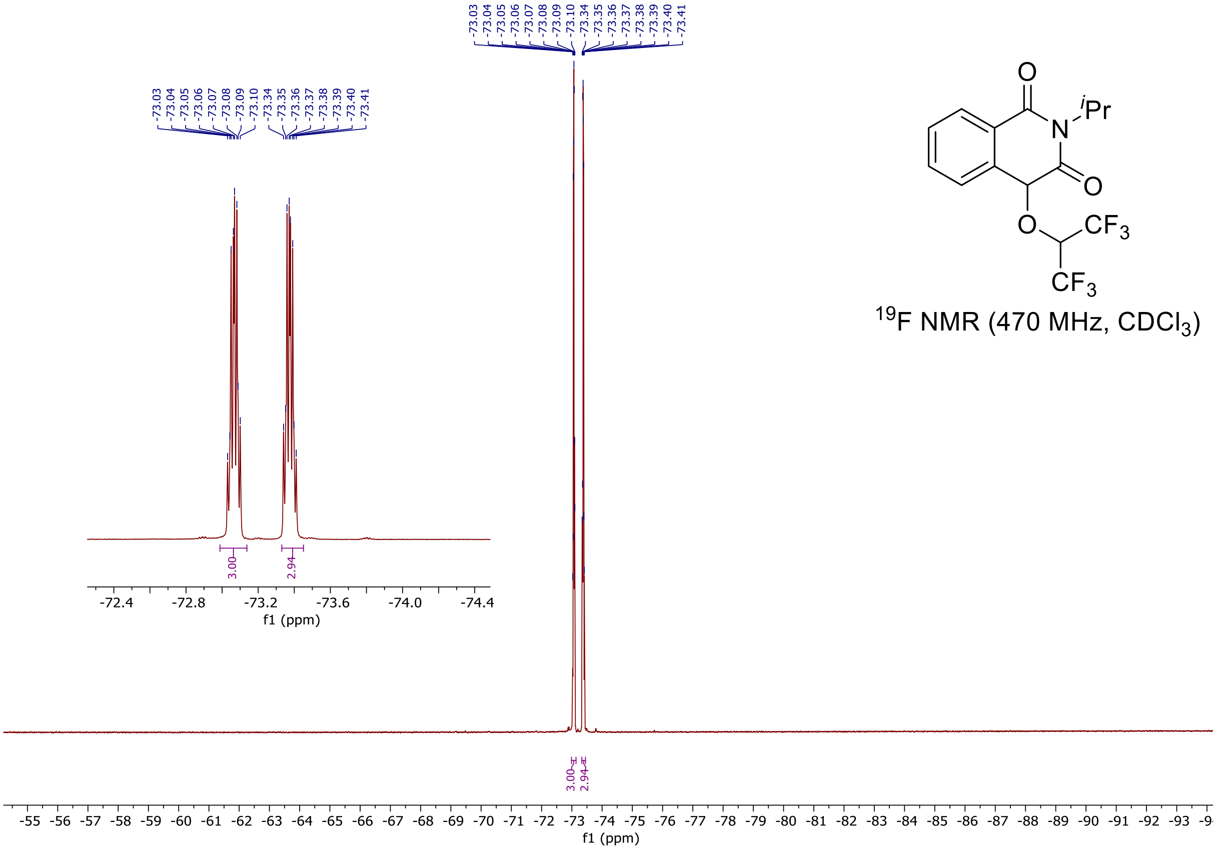
-73.03
-73.04
-73.05
-73.06
-73.07
-73.08
-73.09
-73.10
-73.34
-73.35
-73.36
-73.37
-73.38
-73.39
-73.40
-73.41
3.00
2.94
^{19}F NMR (470 MHz, $CDCl_3$)
f1 (ppm)

2-benzyl-4-((1,1,1,3,3,3-hexafluoropropan-2-yl)oxy)isoquinoline-1,3(2*H*,4*H*)-dione (**6**)

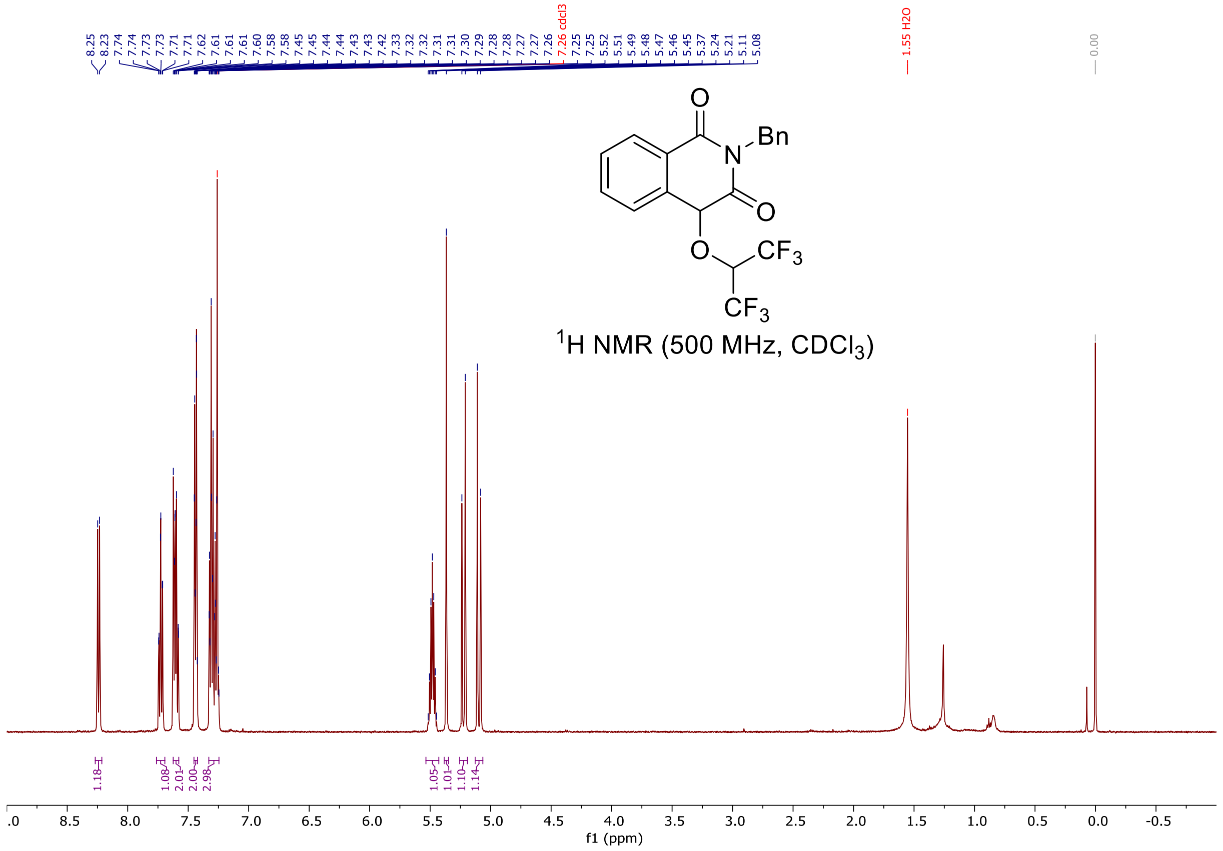

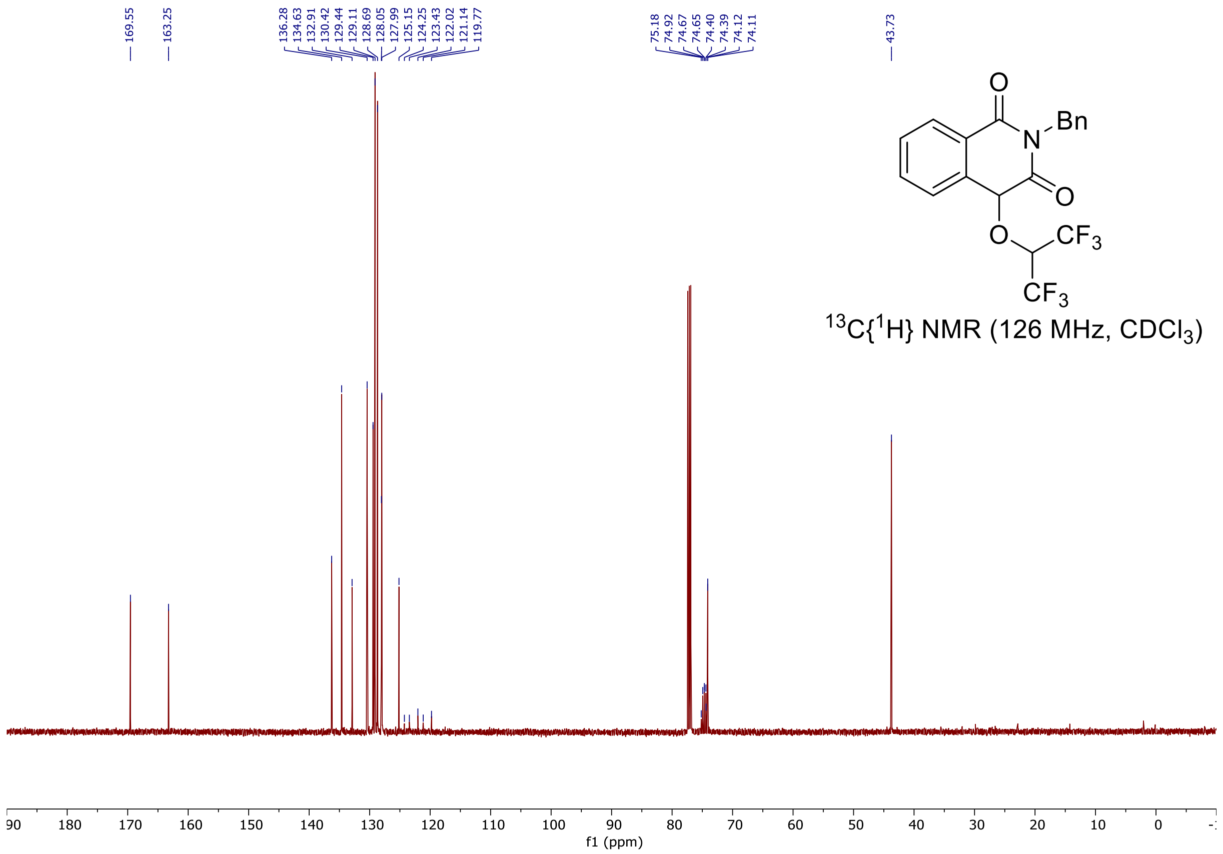

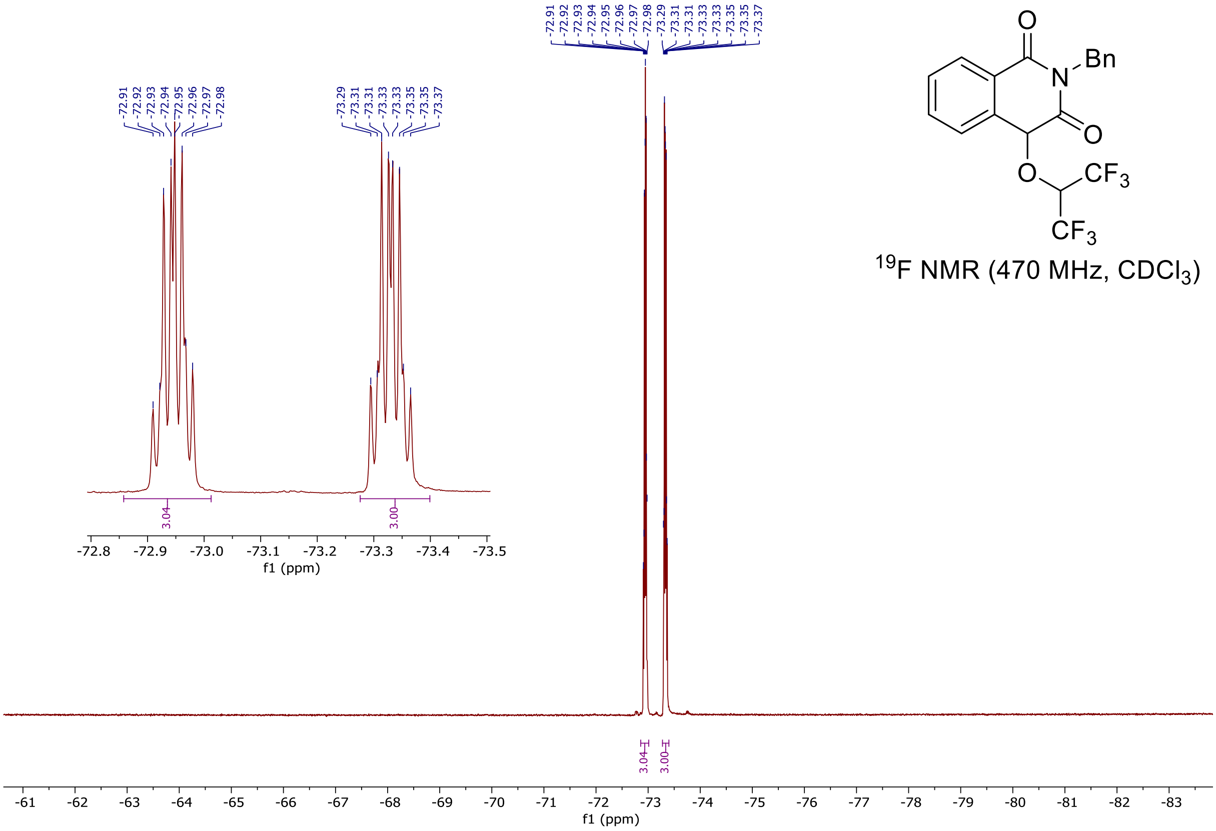

4-((1,1,1,3,3,3-hexafluoropropan-2-yl)oxy)-2-phenylisoquinoline-1,3(2*H*,4*H*)-dione (**7**)

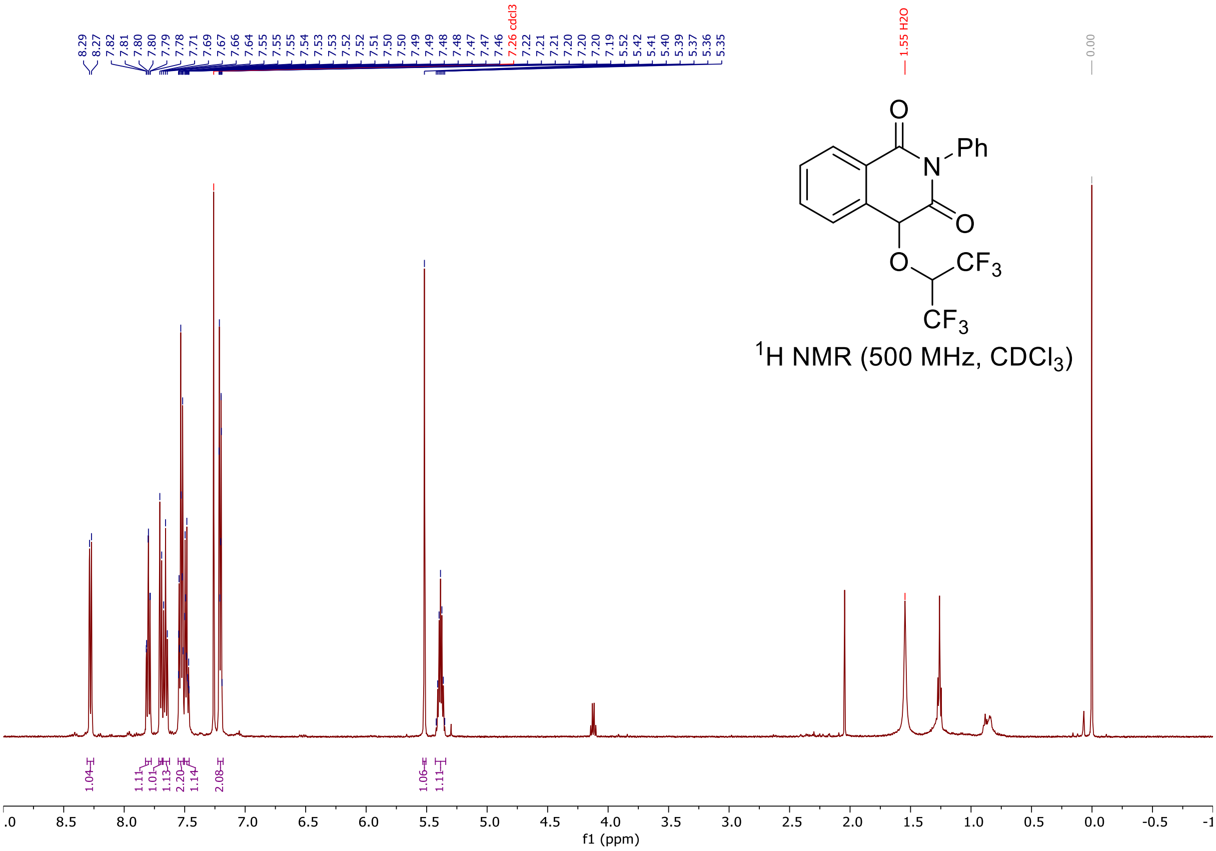

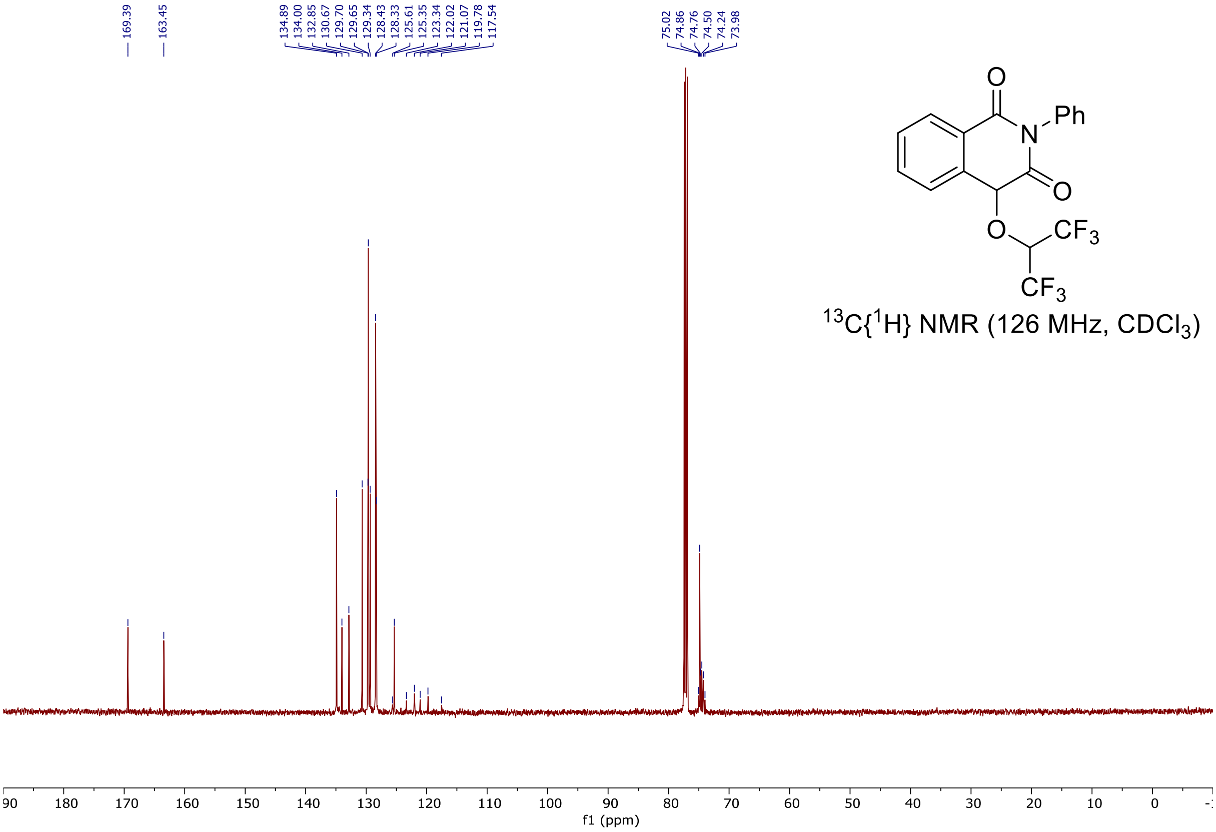

169.39
163.45
134.89 134.00 132.85 130.67 129.70 129.65 129.34 128.43 128.33 125.61 125.35 123.34 122.02 121.07 119.78 117.54
75.02 74.86 74.76 74.50 74.24 73.98
O
N
Ph
O
O
CF3
CF3
13C{1H} NMR (126 MHz, CDCl3)
f1 (ppm)

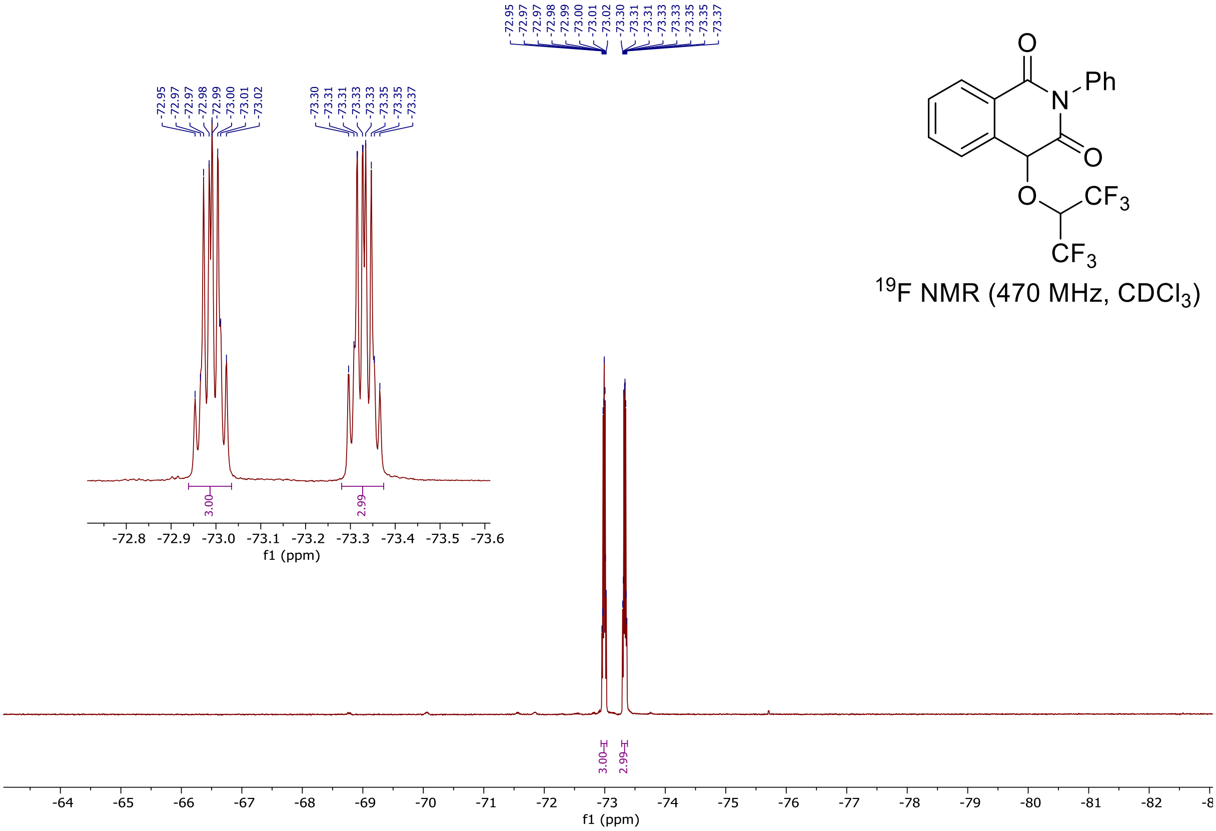

-72.95 -72.97 -72.97 -72.98 -72.99 -73.00 -73.01 -73.02 -73.30 -73.31 -73.31 -73.33 -73.33 -73.35 -73.35 -73.37
O
N
Ph
O
O
CF3
CF3
19F NMR (470 MHz, CDCl3)
3.00
2.99
f1 (ppm)

7-chloro-4-((1,1,1,3,3,3-hexafluoropropan-2-yl)oxy)-2-methylisoquinoline-1,3(2*H*,4*H*)-dione (**8**)

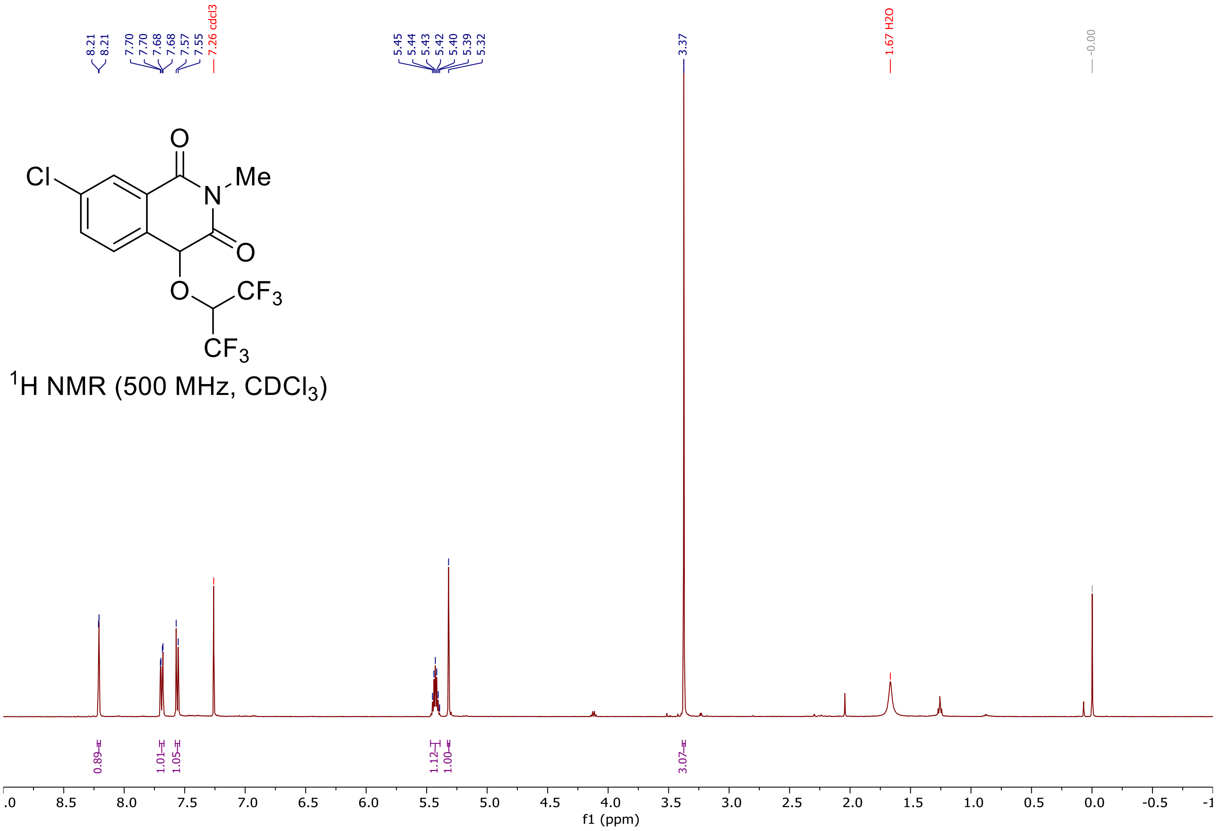

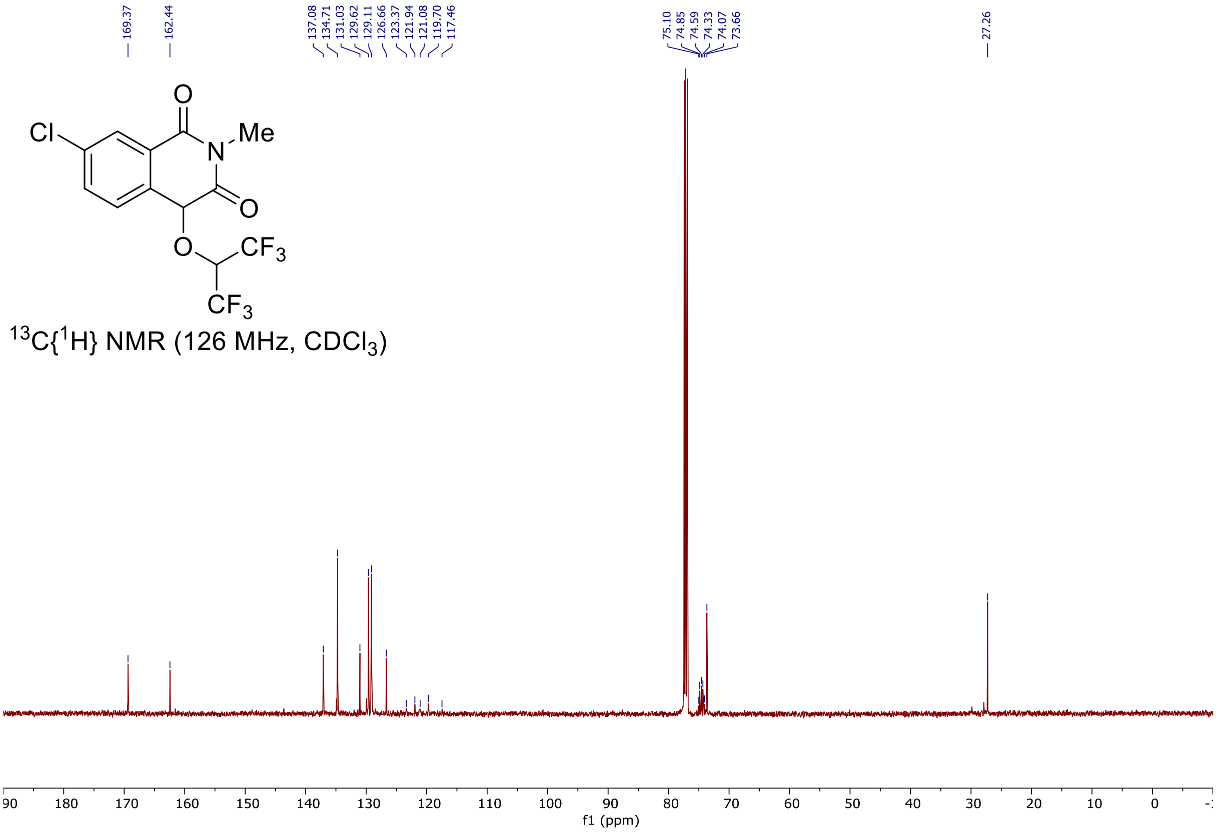

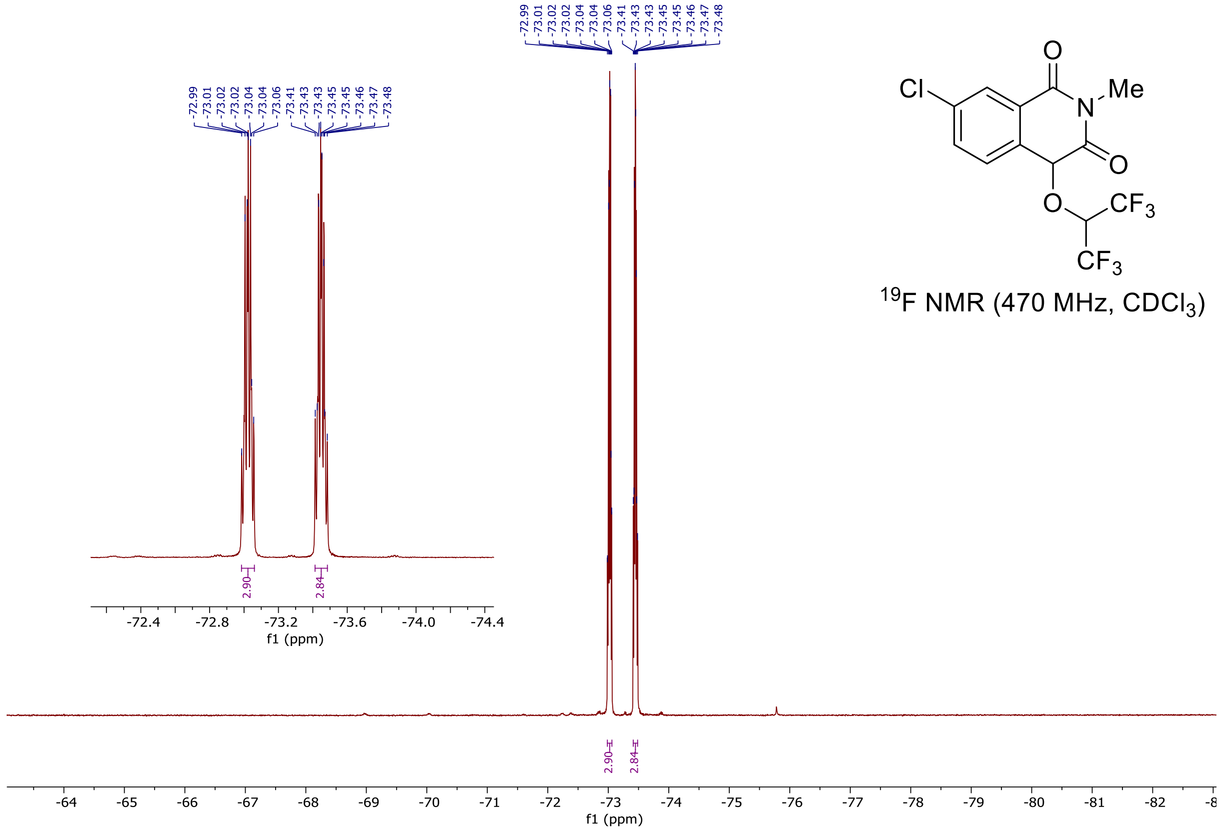

7-bromo-4-((1,1,1,3,3,3-hexafluoropropan-2-yl)oxy)-2-methylisoquinoline-1,3(2*H*,4*H*)-dione (**9**)

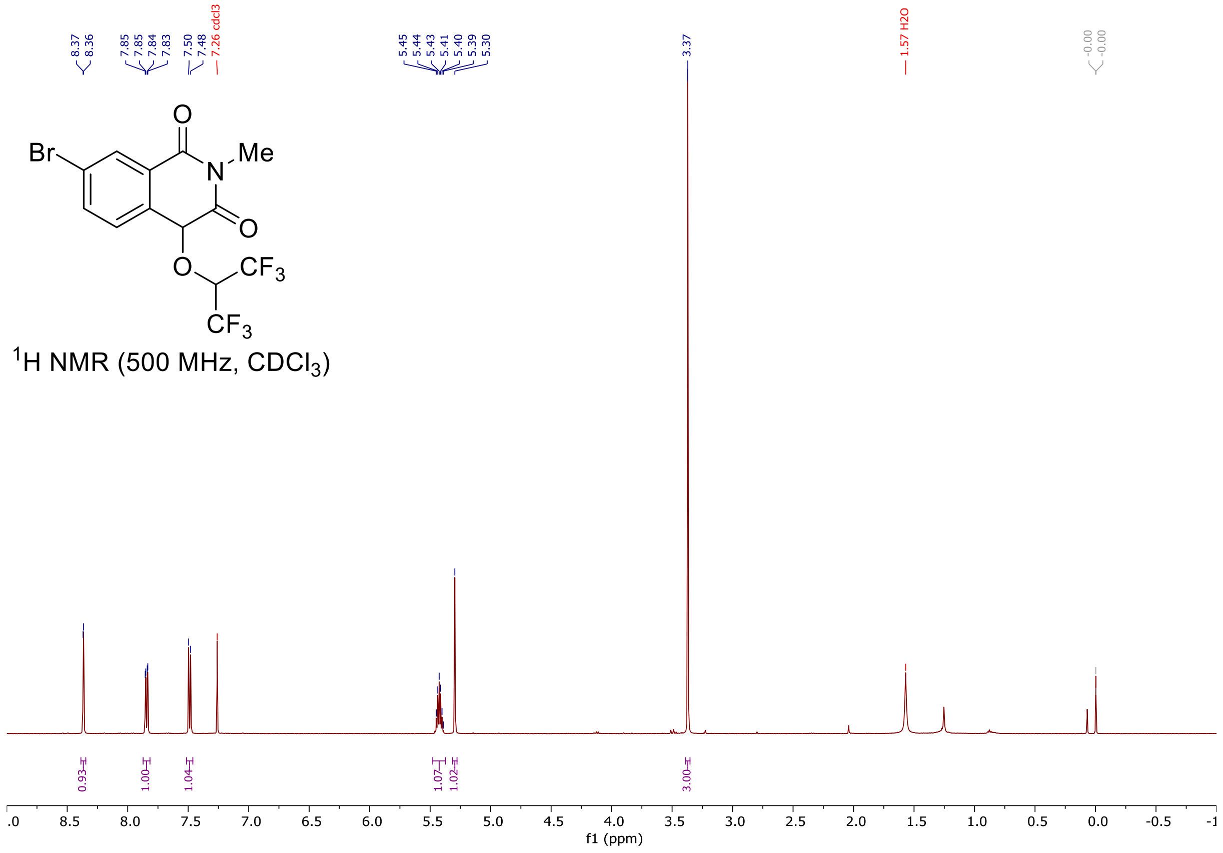

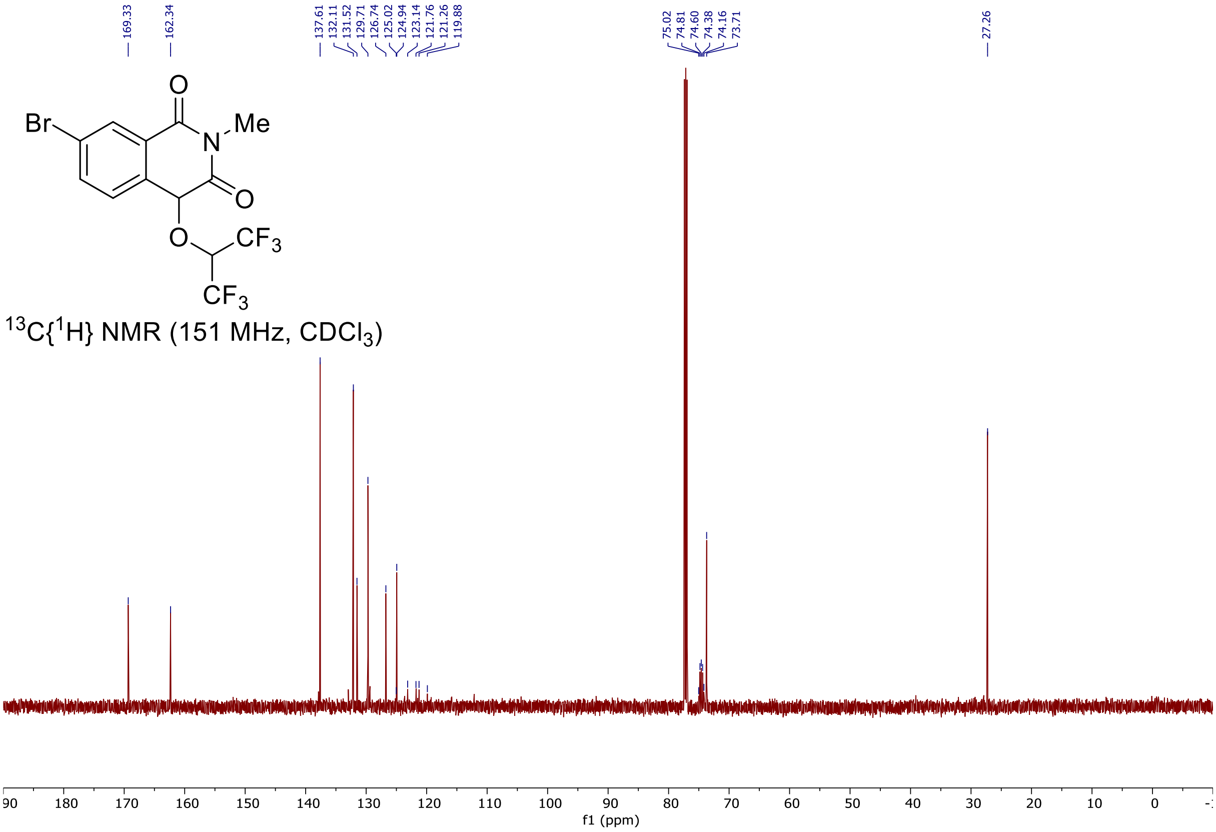
169.33
162.34
137.61
132.11
131.52
129.71
126.74
125.02
124.94
123.14
121.76
121.26
119.88
75.02
74.81
74.60
74.38
74.16
73.71
27.26
Br
O
N
Me
O
O
CF3
CF3
13C{1H} NMR (151 MHz, CDCl3)
f1 (ppm)

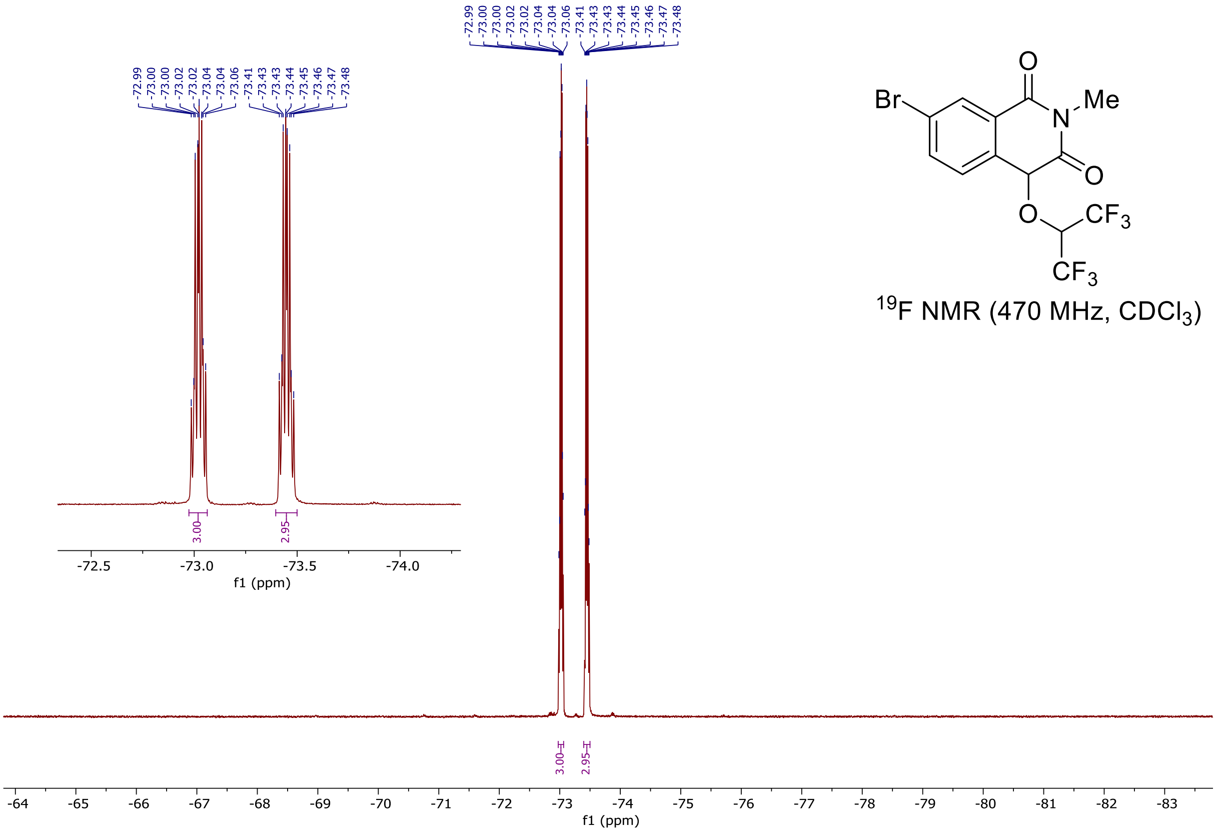
-72.99
-73.00
-73.00
-73.02
-73.02
-73.04
-73.04
-73.06
-73.41
-73.43
-73.43
-73.44
-73.45
-73.46
-73.47
-73.48
3.00
2.95
-72.5
-73.0
-73.5
-74.0
f1 (ppm)
Br
O
N
Me
O
O
CF3
CF3
19F NMR (470 MHz, CDCl3)

4-((1,1,1,3,3,3-hexafluoropropan-2-yl)oxy)-2,7-dimethylisoquinoline-1,3(2*H*,4*H*)-dione (**10**)

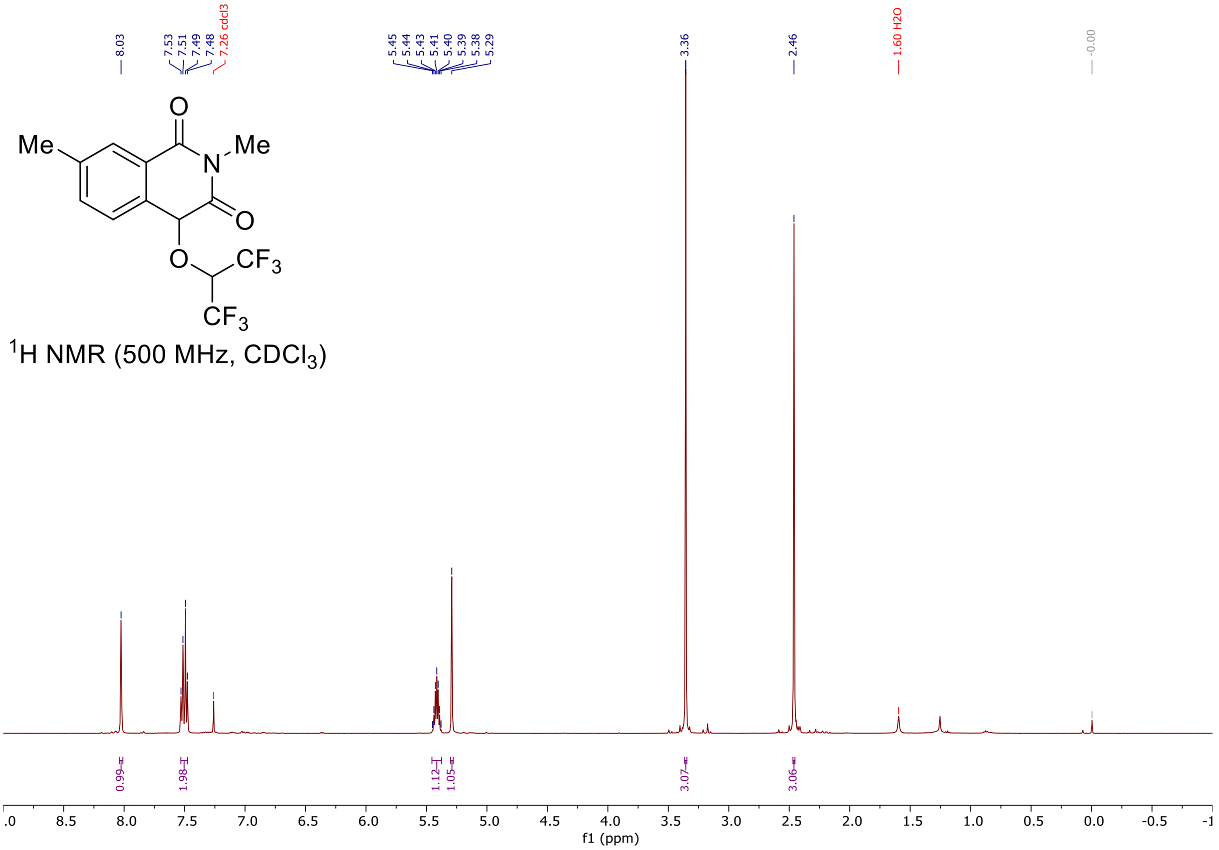

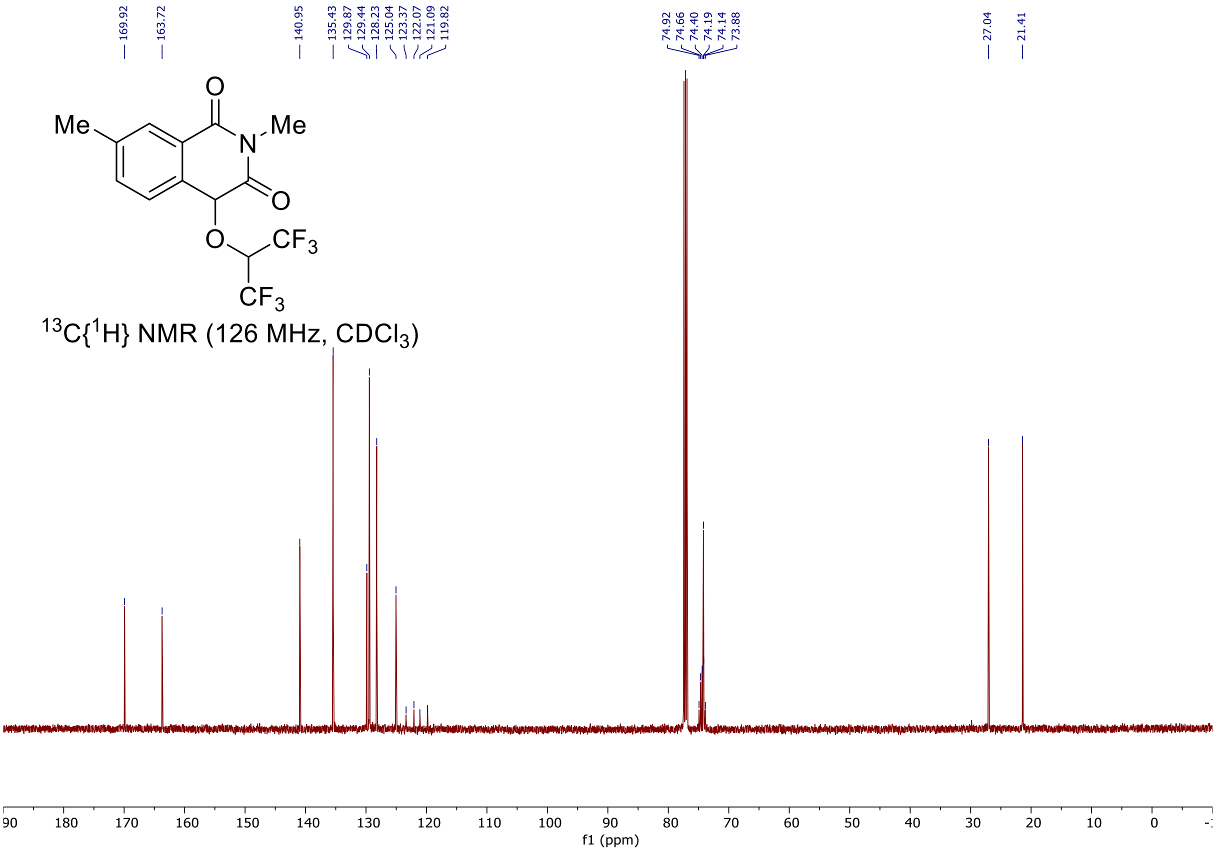

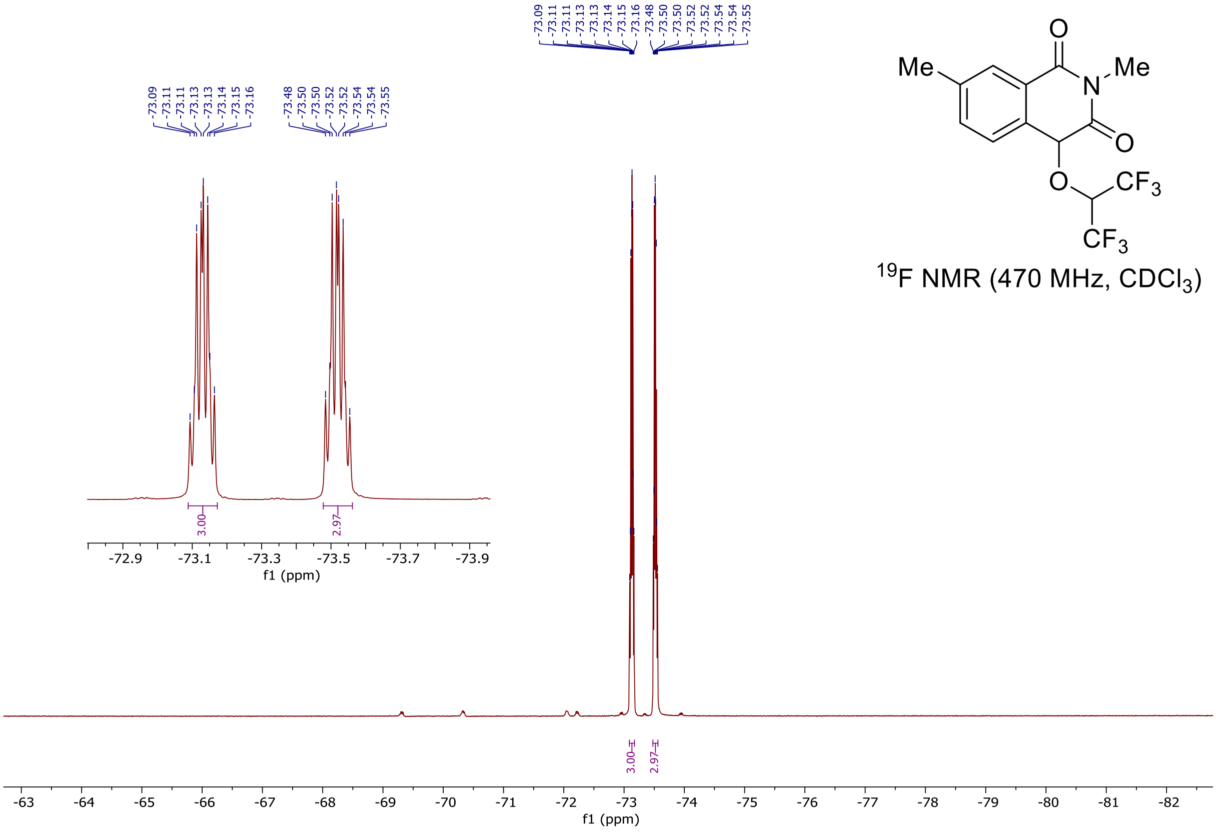

4-((1,1,1,3,3,3-hexafluoropropan-2-yl)oxy)-7-methoxy-2-methylisoquinoline-1,3(2*H*,4*H*)-dione (**11**)

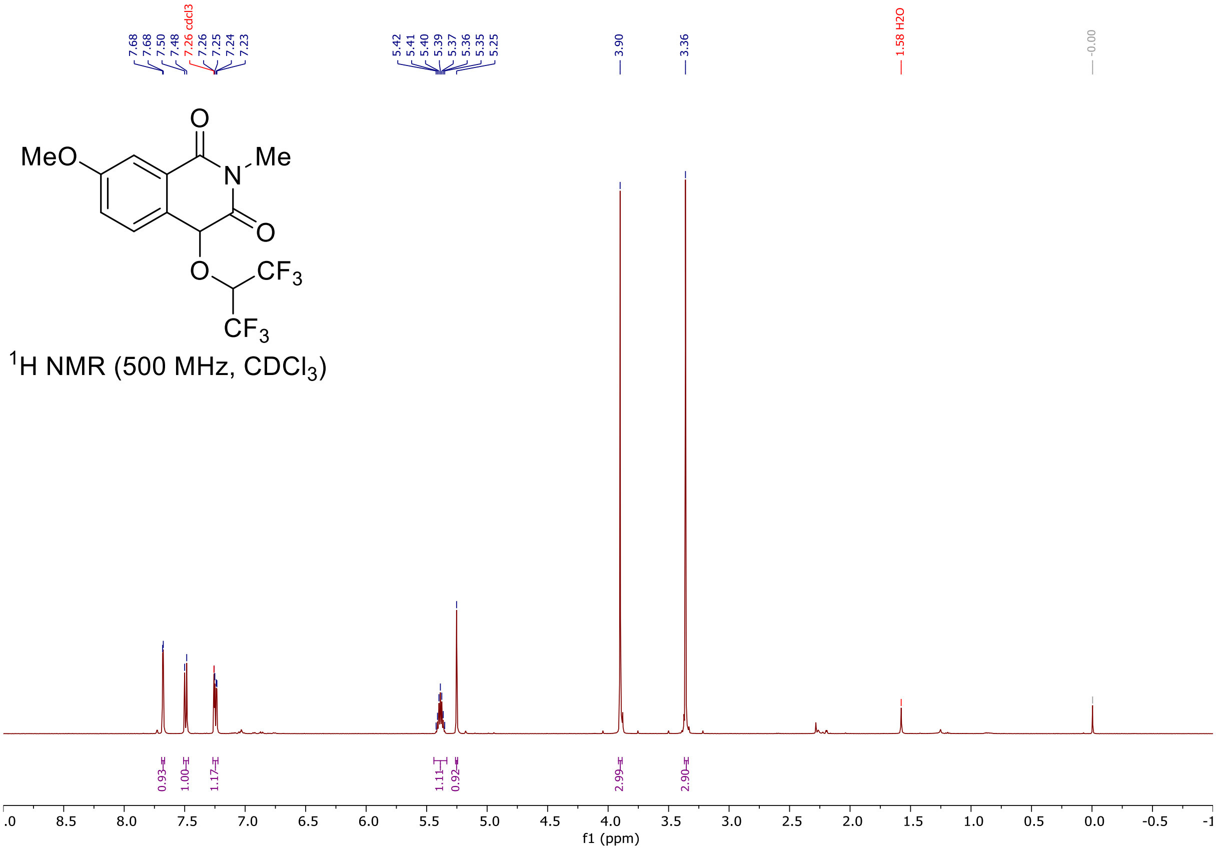

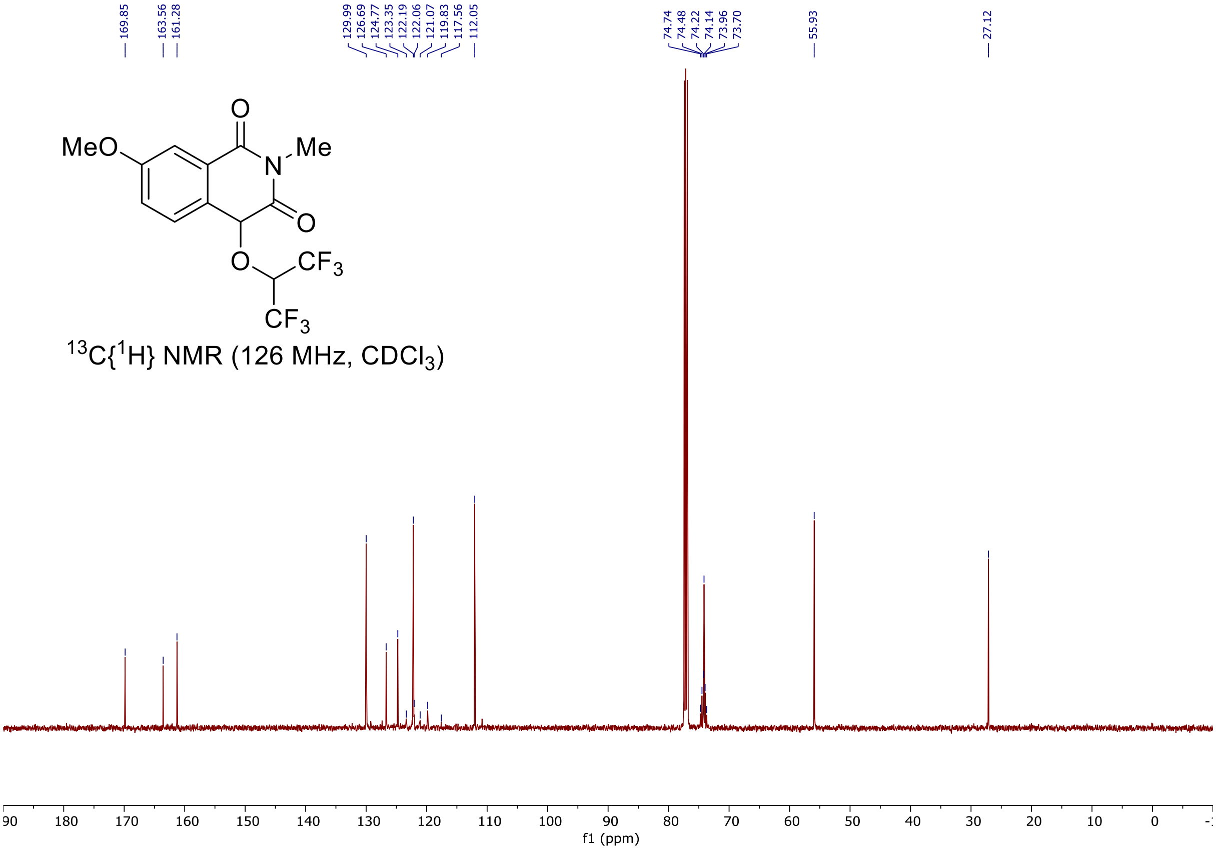

169.85
163.56
161.28
129.99
126.69
124.77
123.35
122.19
122.06
121.07
119.83
117.56
112.05
74.74
74.48
74.22
74.14
73.96
73.70
55.93
27.12
MeO
O
N
Me
O
O
CF3
CF3
$^{13}C\{^{1}H\}$ NMR (126 MHz, $CDCl_3$)
f1 (ppm)

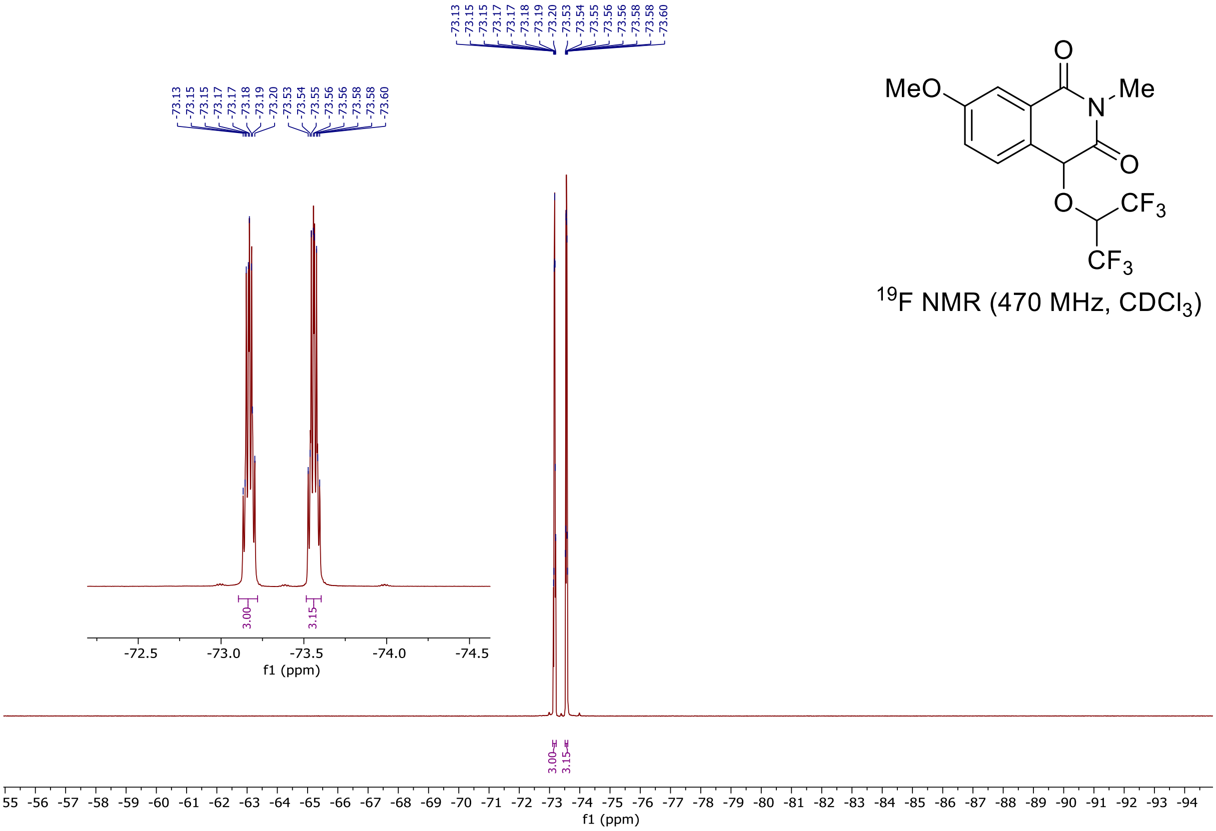

-73.13
-73.15
-73.15
-73.17
-73.17
-73.18
-73.19
-73.20
-73.53
-73.54
-73.55
-73.56
-73.56
-73.58
-73.58
-73.60
MeO
O
N
Me
O
O
CF3
CF3
^{19}F NMR (470 MHz, $CDCl_3$)
3.00
3.15
f1 (ppm)

4-((1,1,1,3,3,3-hexafluoropropan-2-yl)oxy)-6-methoxy-2-methylisoquinoline-1,3(2*H*,4*H*)-dione (**12**)

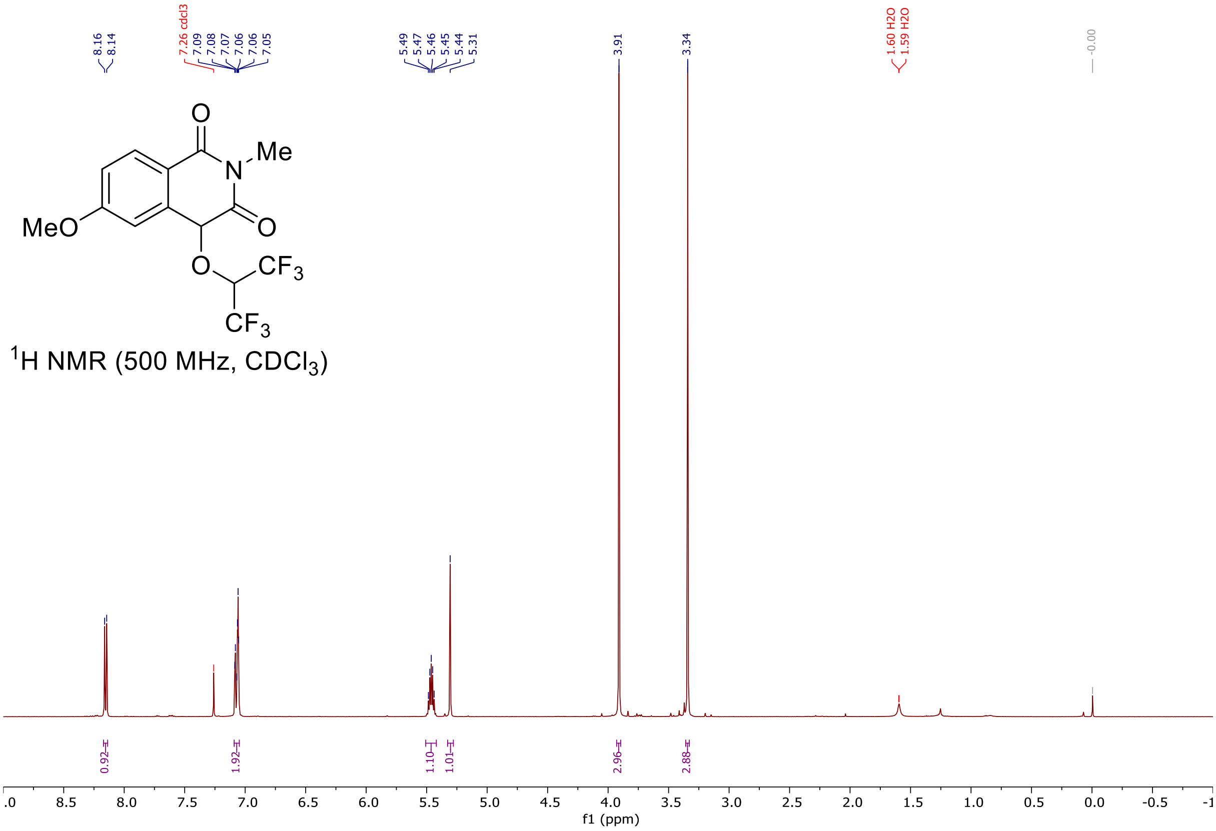

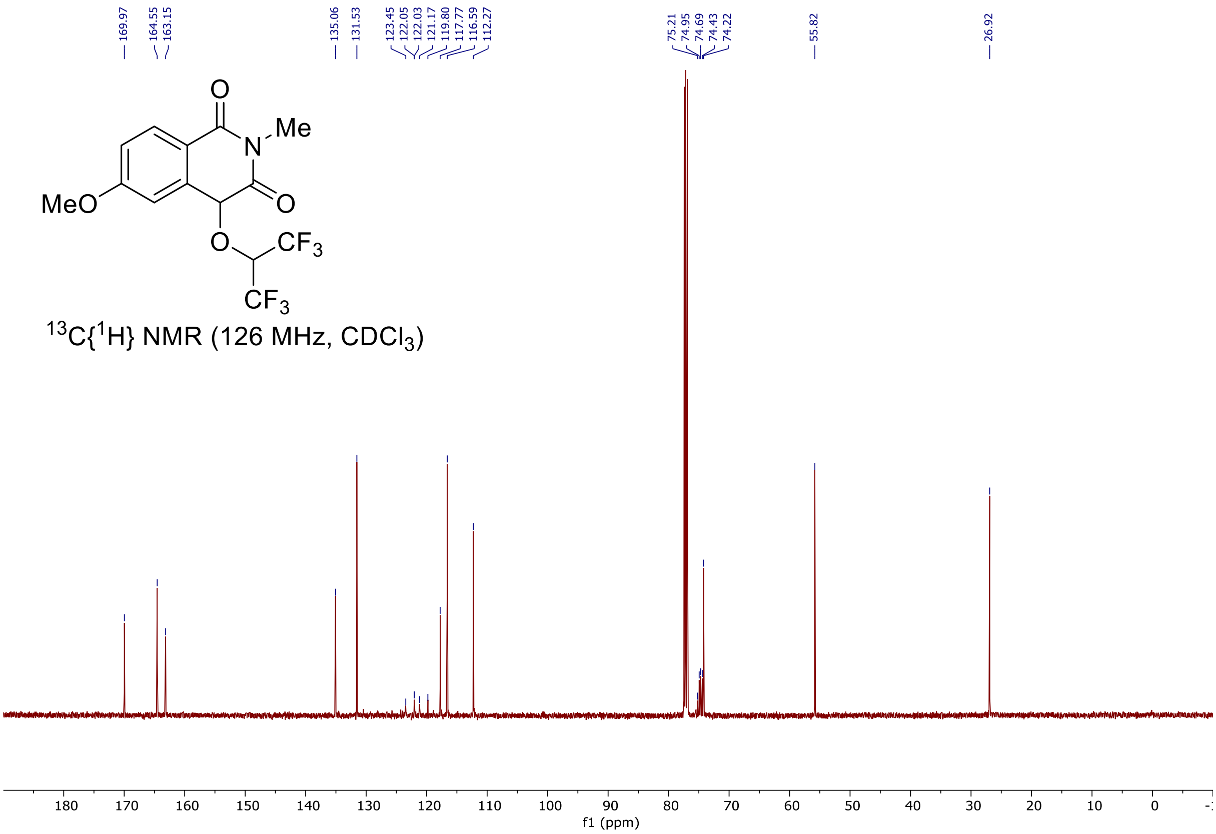

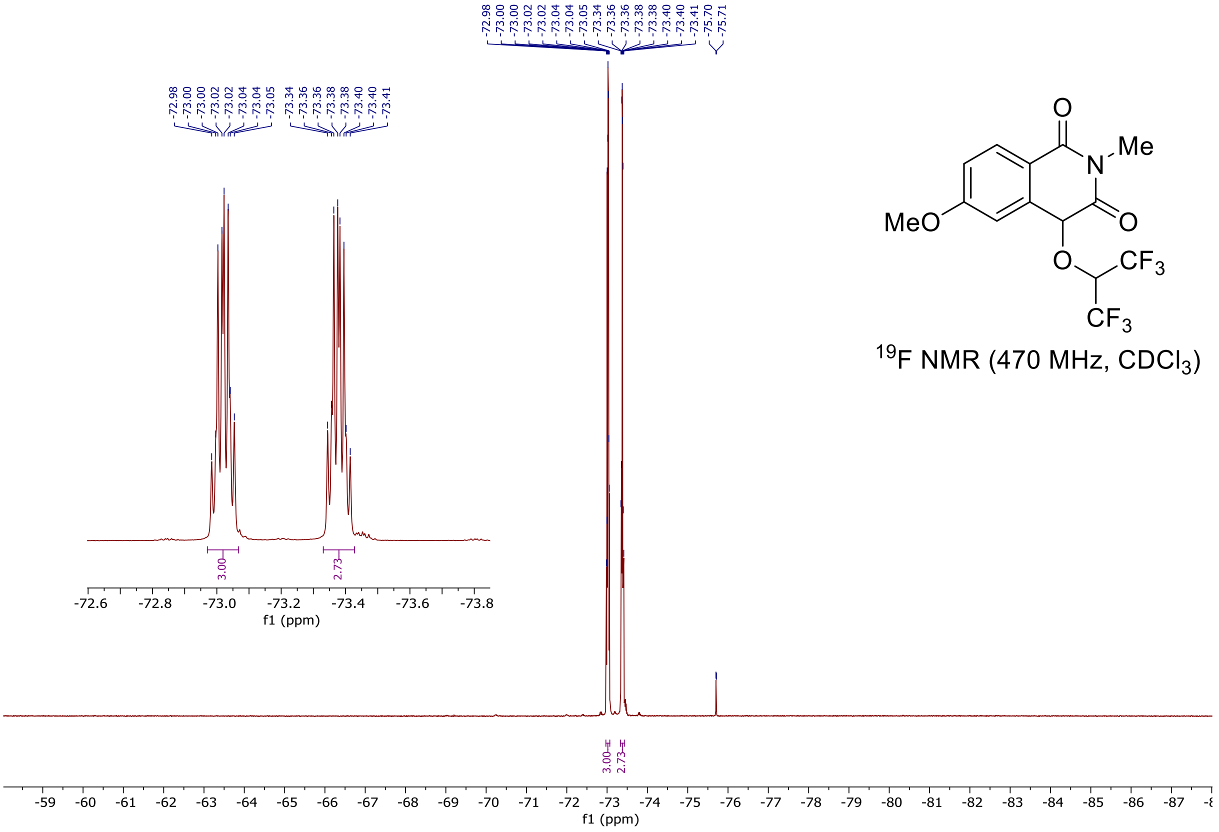

8-chloro-4-((1,1,1,3,3,3-hexafluoropropan-2-yl)oxy)-2-methylisoquinoline-1,3(2*H*,4*H*)-dione (**15**)

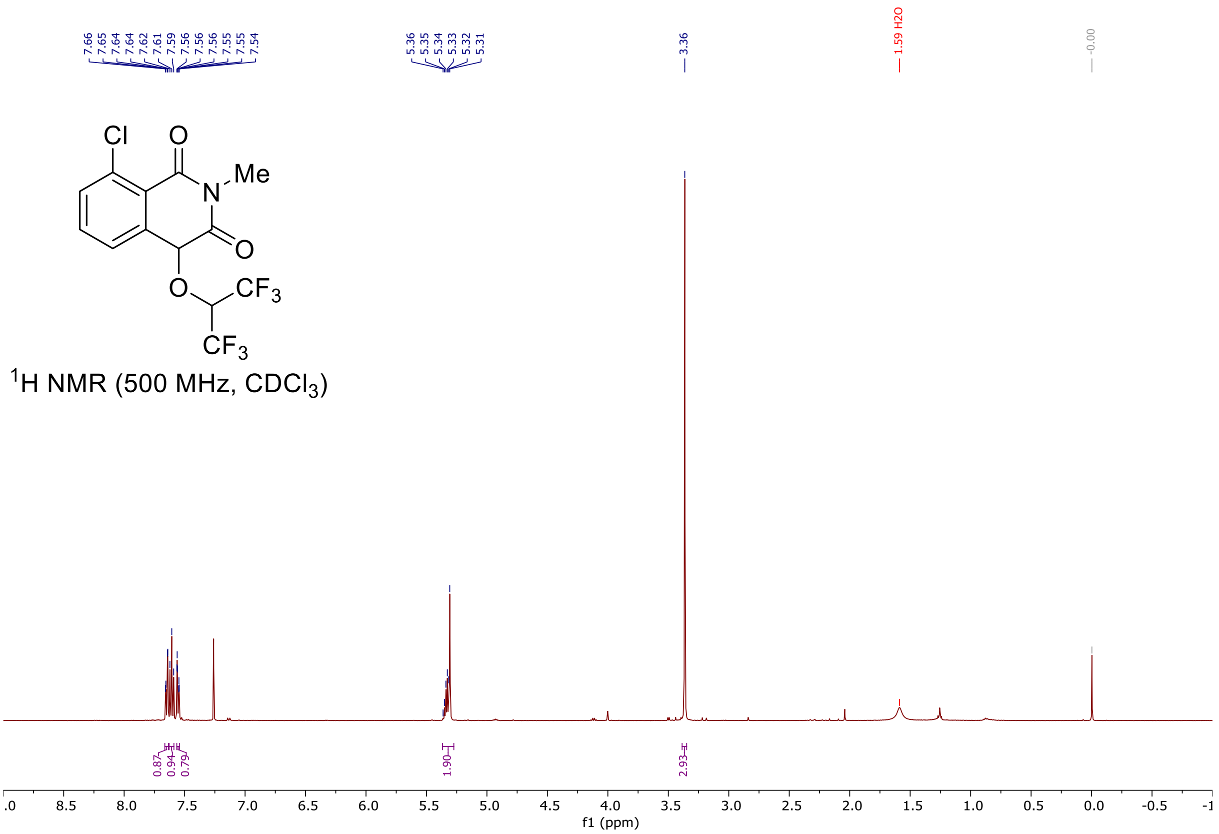

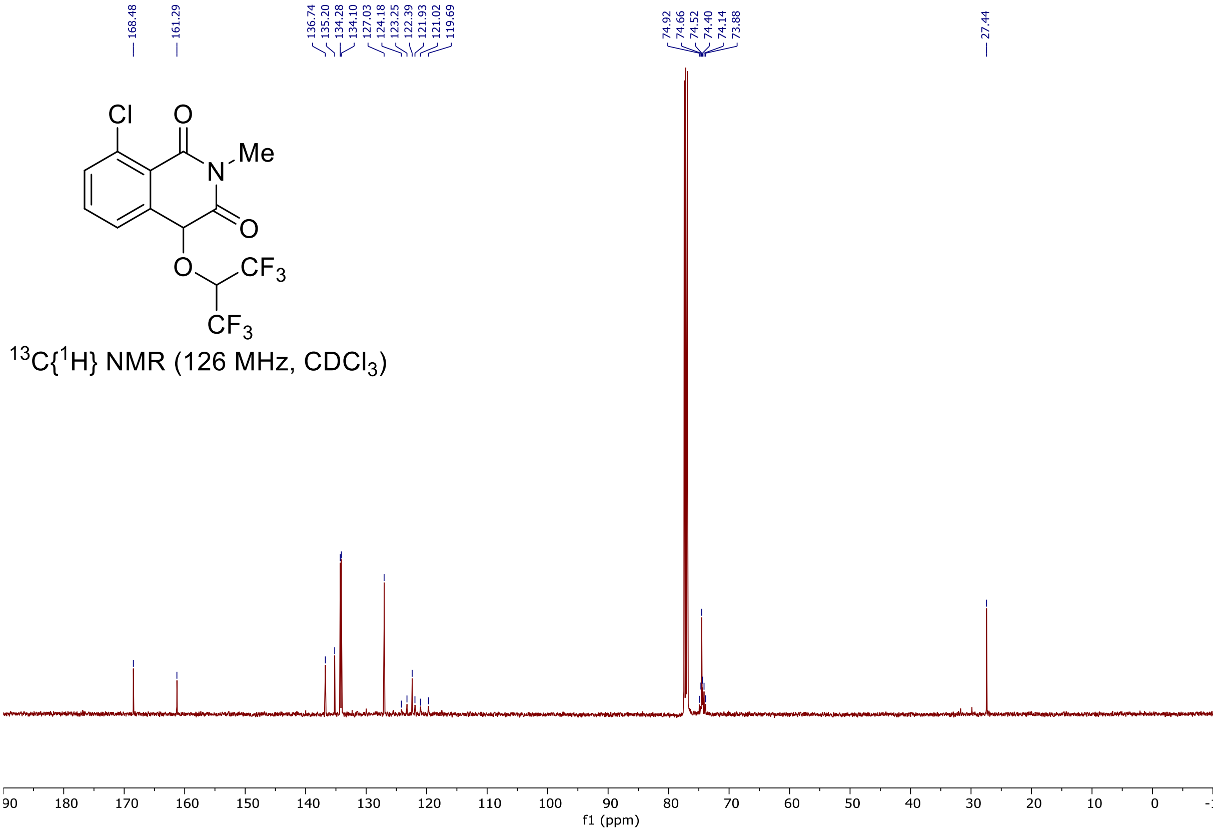
168.48
161.29
136.74
135.20
134.28
134.10
127.03
124.18
123.25
122.39
121.93
121.02
119.69
74.92
74.66
74.52
74.40
74.14
73.88
27.44
Cl
O
N
Me
O
O
CF3
CF3
13C{1H} NMR (126 MHz, CDCl3)
f1 (ppm)

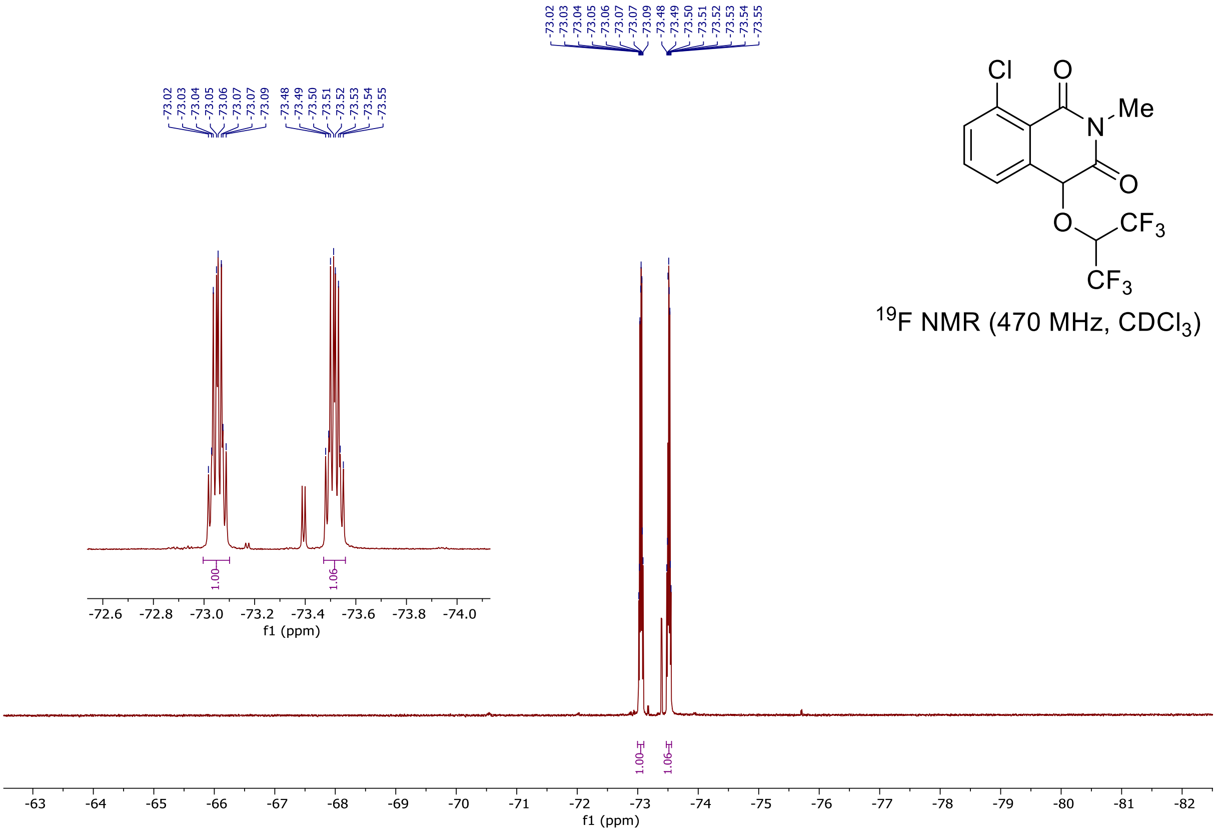
-73.02
-73.03
-73.04
-73.05
-73.06
-73.07
-73.07
-73.09
-73.48
-73.49
-73.50
-73.51
-73.52
-73.53
-73.54
-73.55
Cl
O
N
Me
O
O
CF3
CF3
19F NMR (470 MHz, CDCl3)
1.00
1.06
f1 (ppm)

5-chloro-4-((1,1,1,3,3,3-hexafluoropropan-2-yl)oxy)-2-methylisoquinoline-1,3(2*H*,4*H*)-dione (**16**)

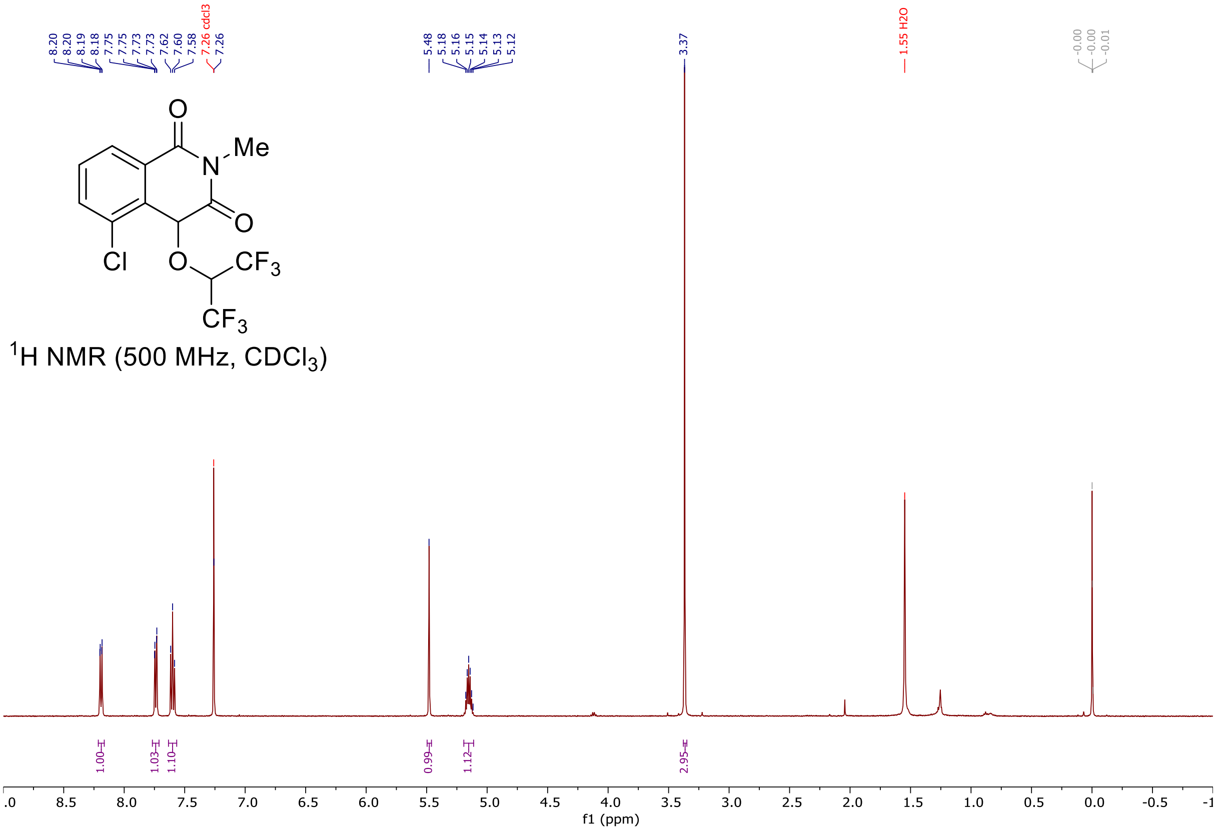

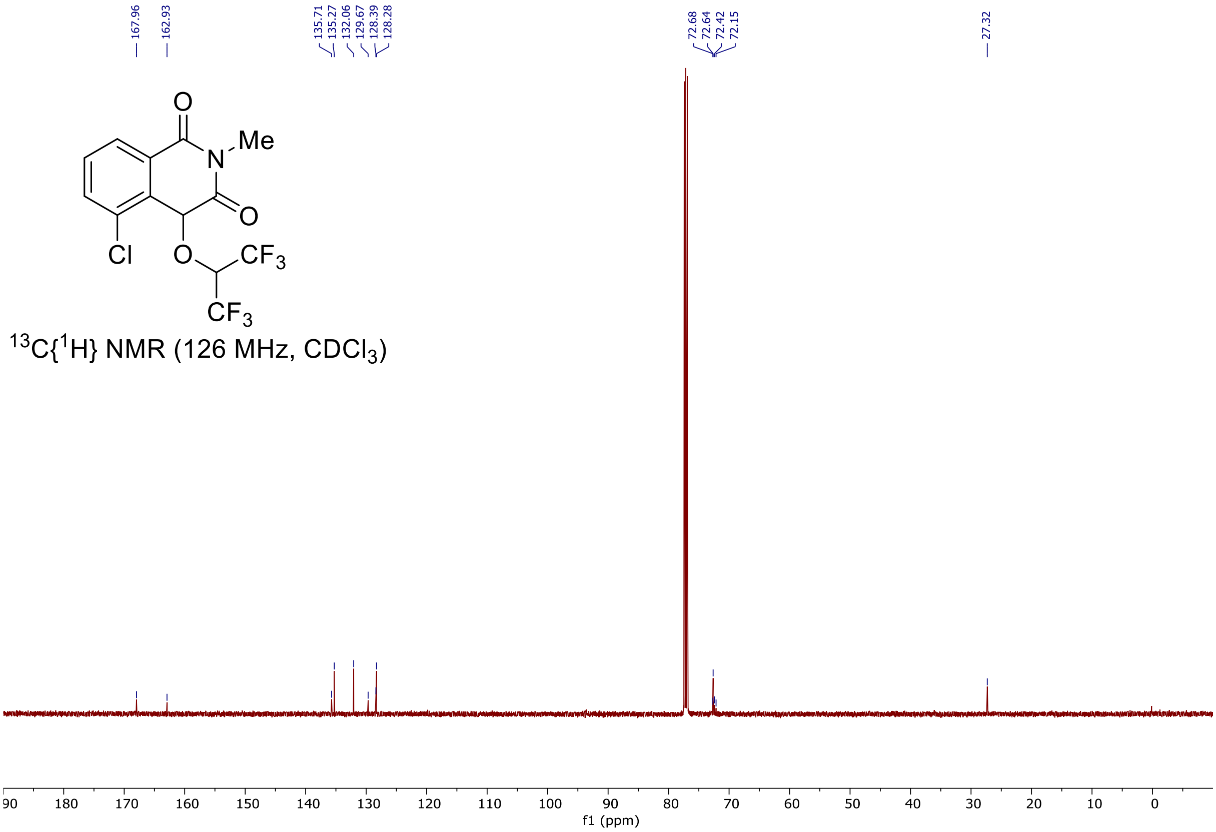

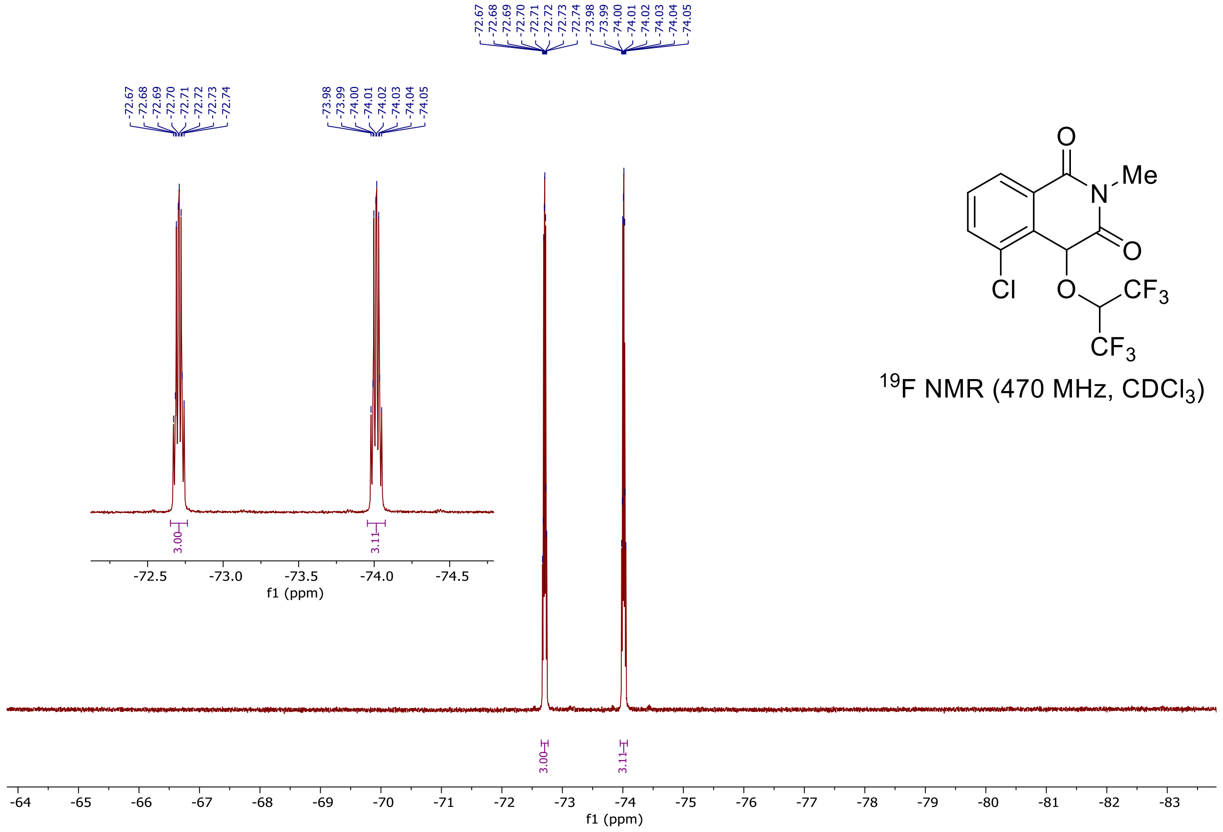

2-methyl-4-(2,2,2-trifluoroethoxy)isoquinoline-1,3(2*H*,4*H*)-dione)(**17**)

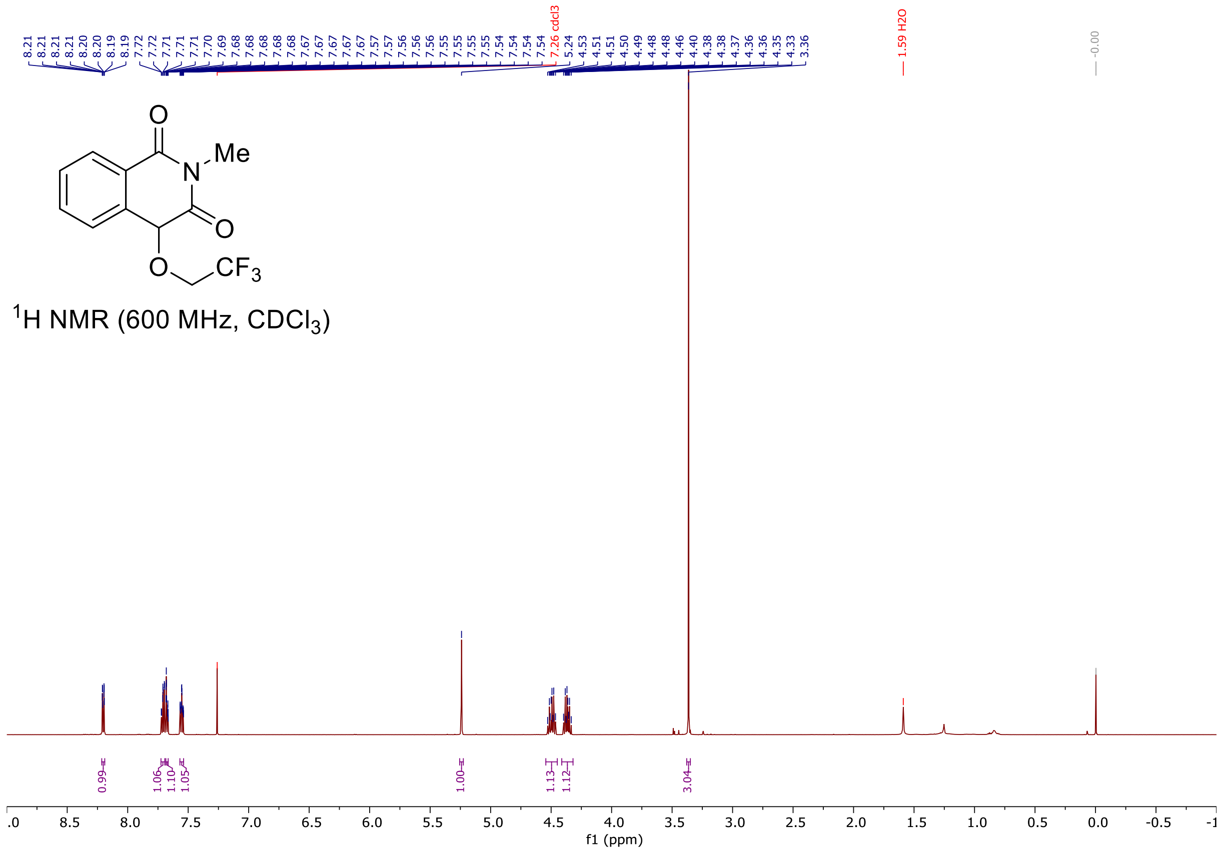

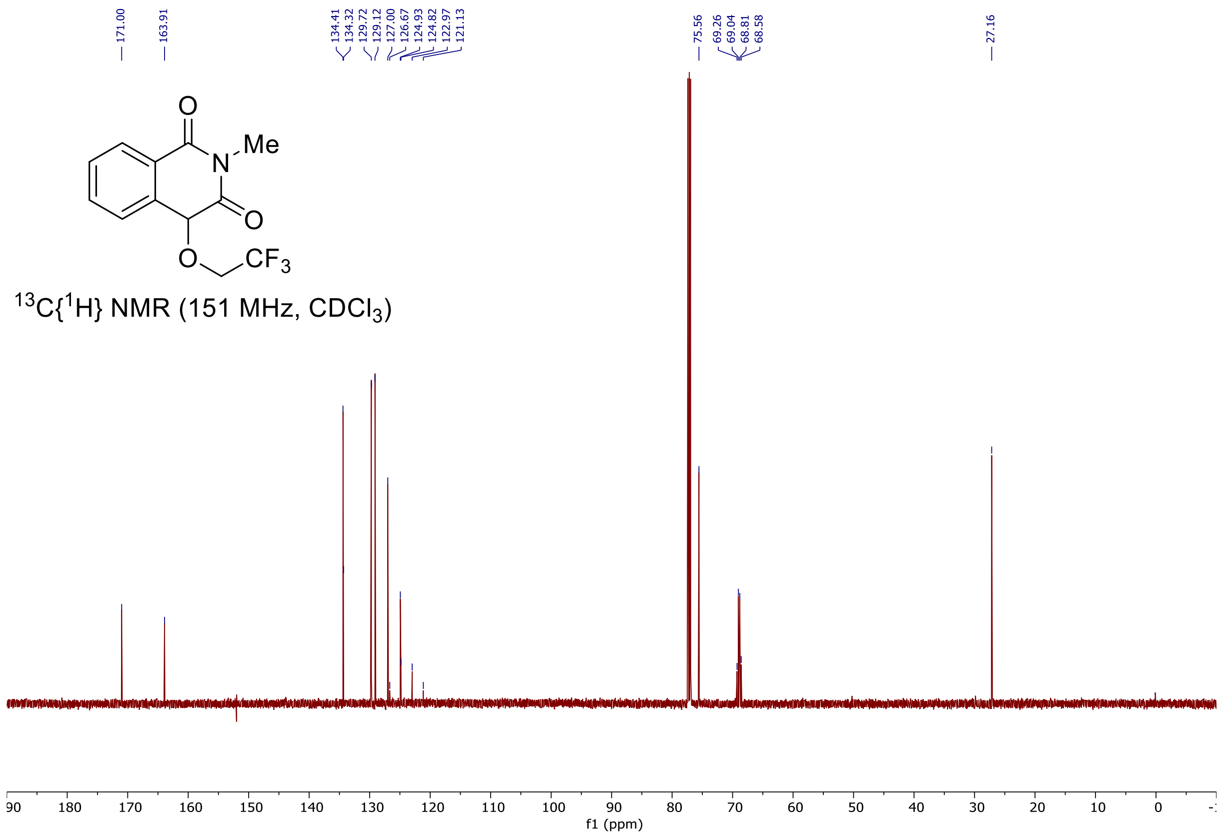
171.00
163.91
134.41
134.32
129.72
129.12
127.00
126.67
124.93
124.82
122.97
121.13
75.56
69.26
69.04
68.81
68.58
27.16
O
N
Me
O
O
CF3
13C{1H} NMR (151 MHz, CDCl3)
f1 (ppm)

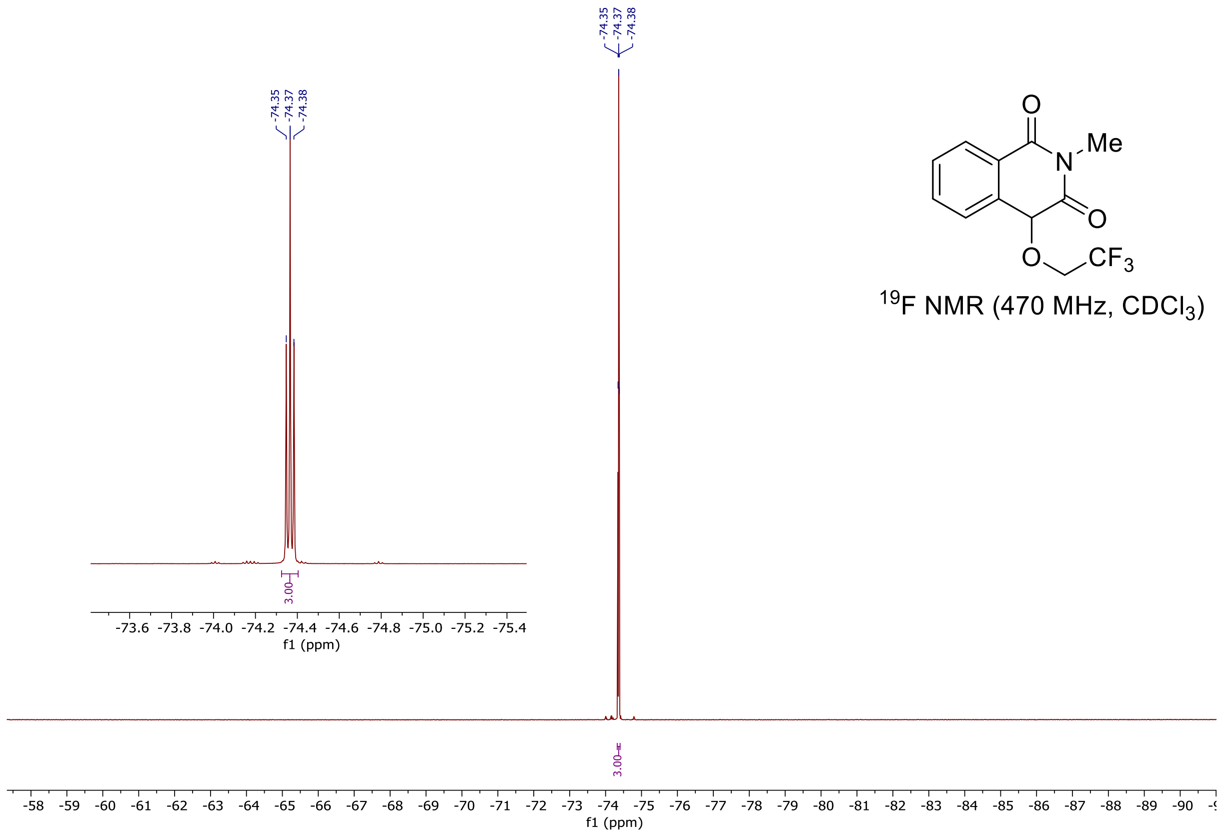
-74.35
-74.37
-74.38
3.00
-73.6 -73.8 -74.0 -74.2 -74.4 -74.6 -74.8 -75.0 -75.2 -75.4
f1 (ppm)
O
N
Me
O
O
CF3
19F NMR (470 MHz, CDCl3)
f1 (ppm)

2-methyl-4-((1,1,1-trifluoropropan-2-yl)oxy)isoquinoline-1,3(2*H*,4*H*)-dione (**18**) (dr 1:10)

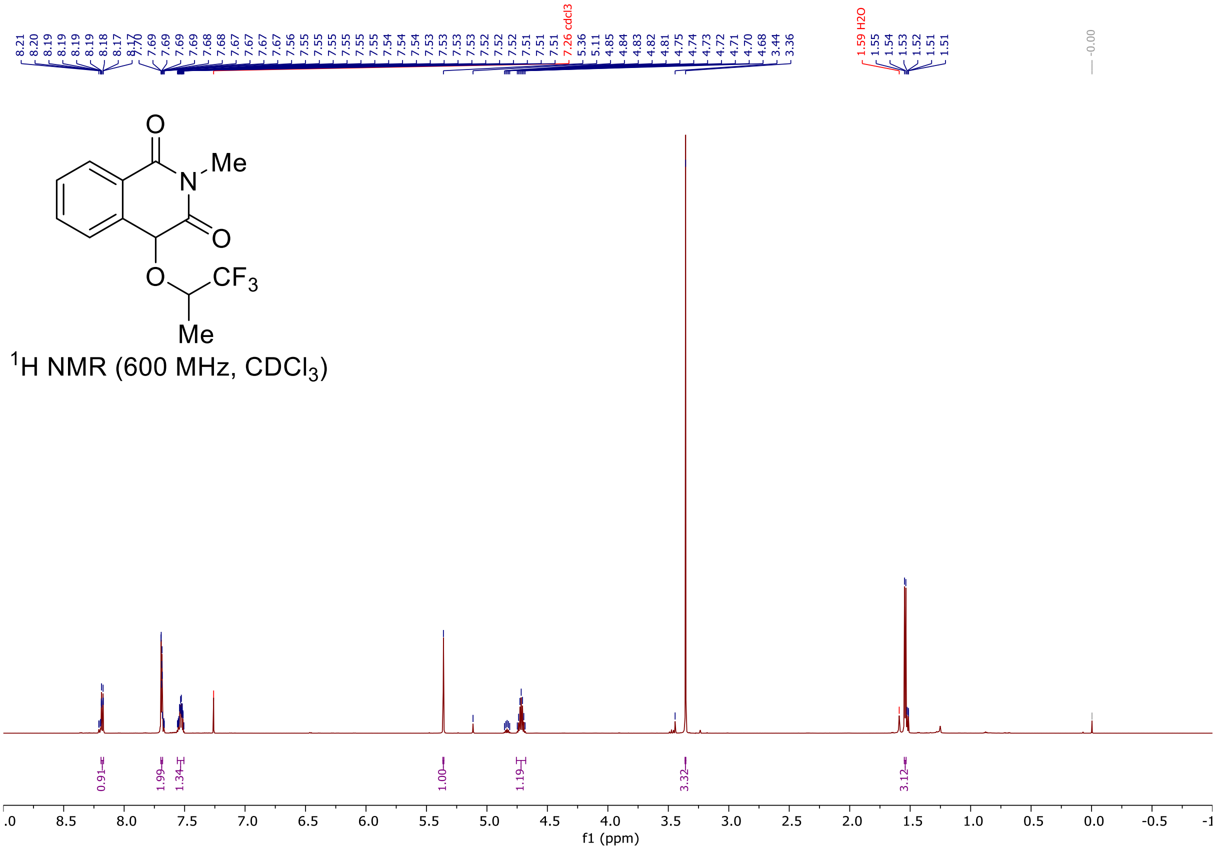

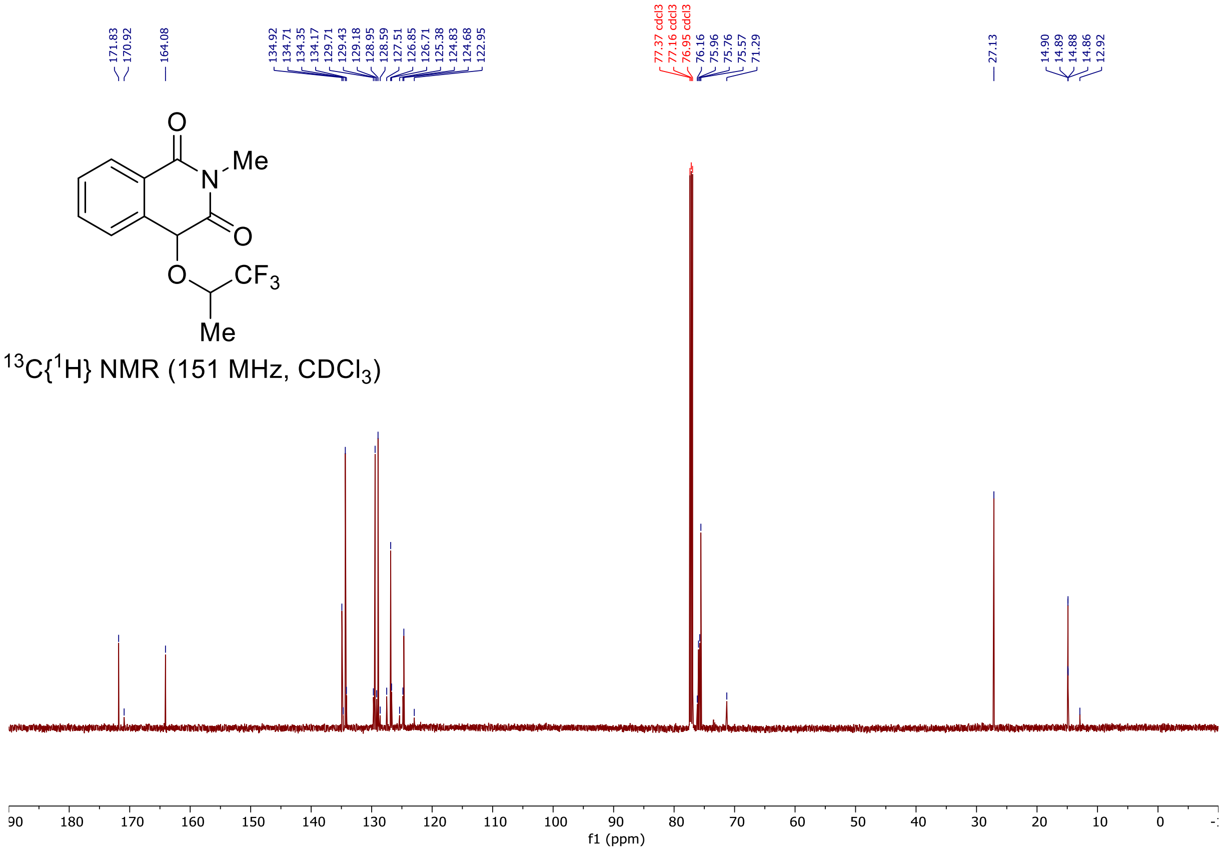

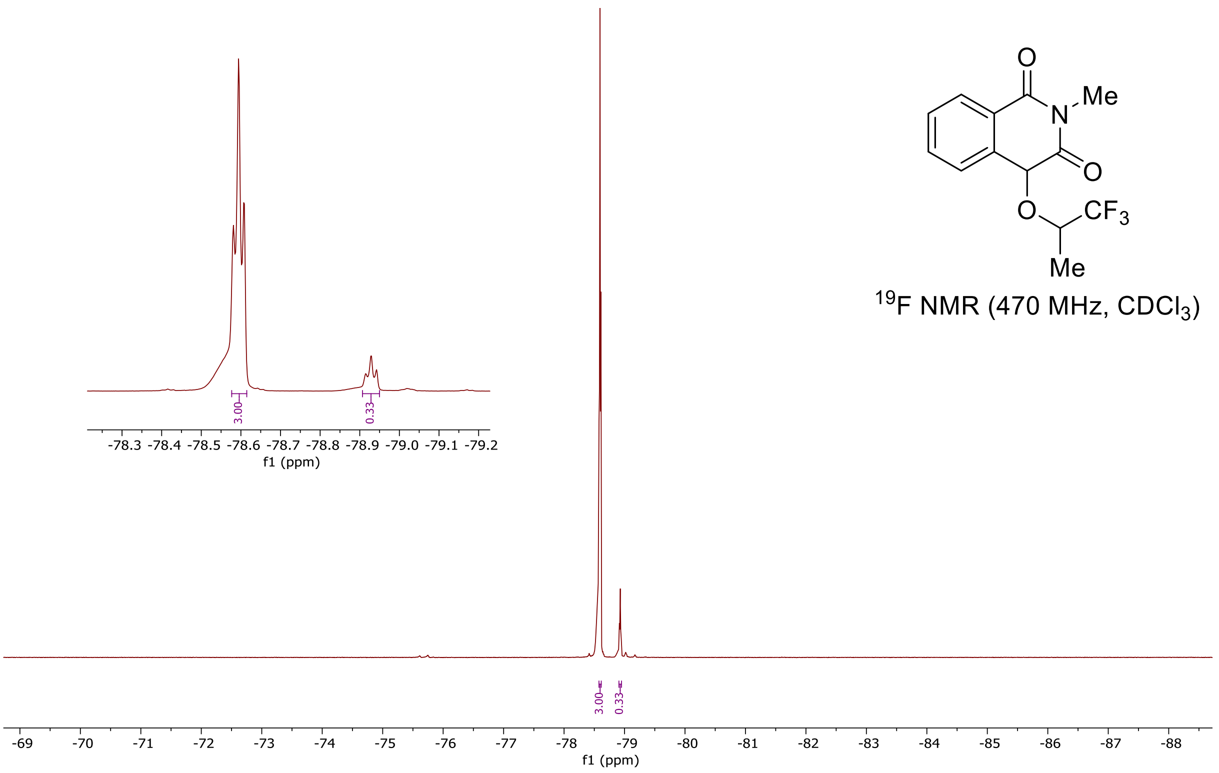

^{19}F NMR (470 MHz, $CDCl_3$)

2-methyl-4-(2,2,3,3-tetrafluoropropoxy)isoquinoline-1,3(2*H*,4*H*)-dione (**19**)

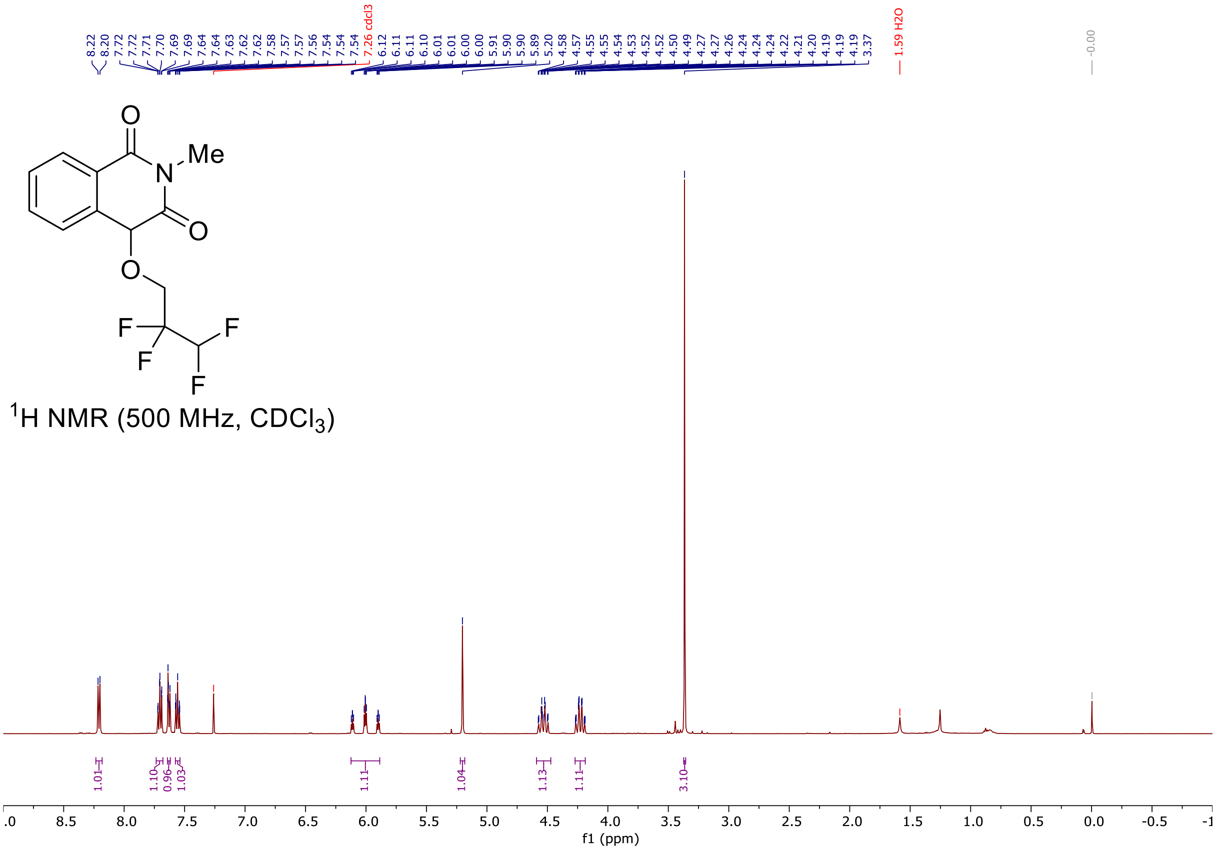

^{1}H NMR (500 MHz, $CDCl_3$)

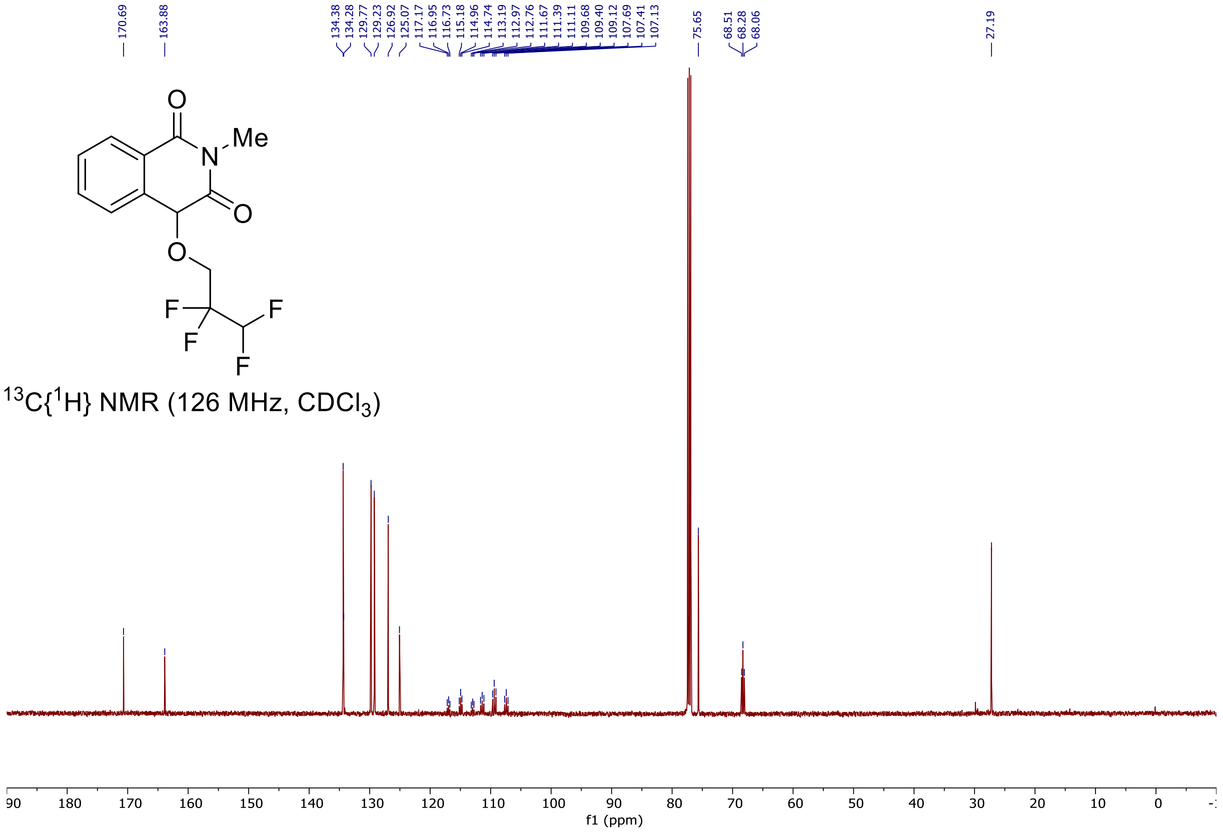

$^{13}C\{^1H\}$ NMR (126 MHz, $CDCl_3$)

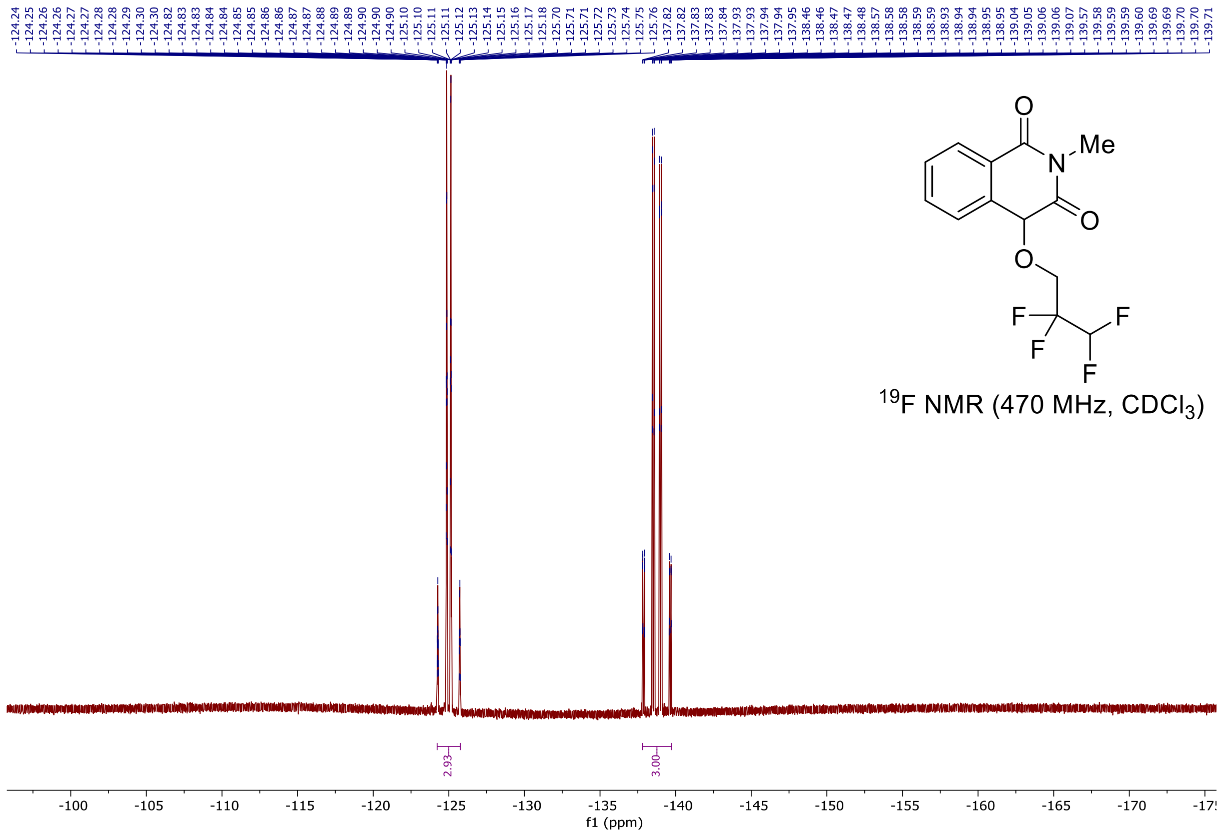

^{19}F NMR (470 MHz, $CDCl_3$)

2-methyl-4-((2,2,3,3,4,4,5,5-octafluoropentyl)oxy)isoquinoline-1,3(2*H*,4*H*)-dione (**20**)

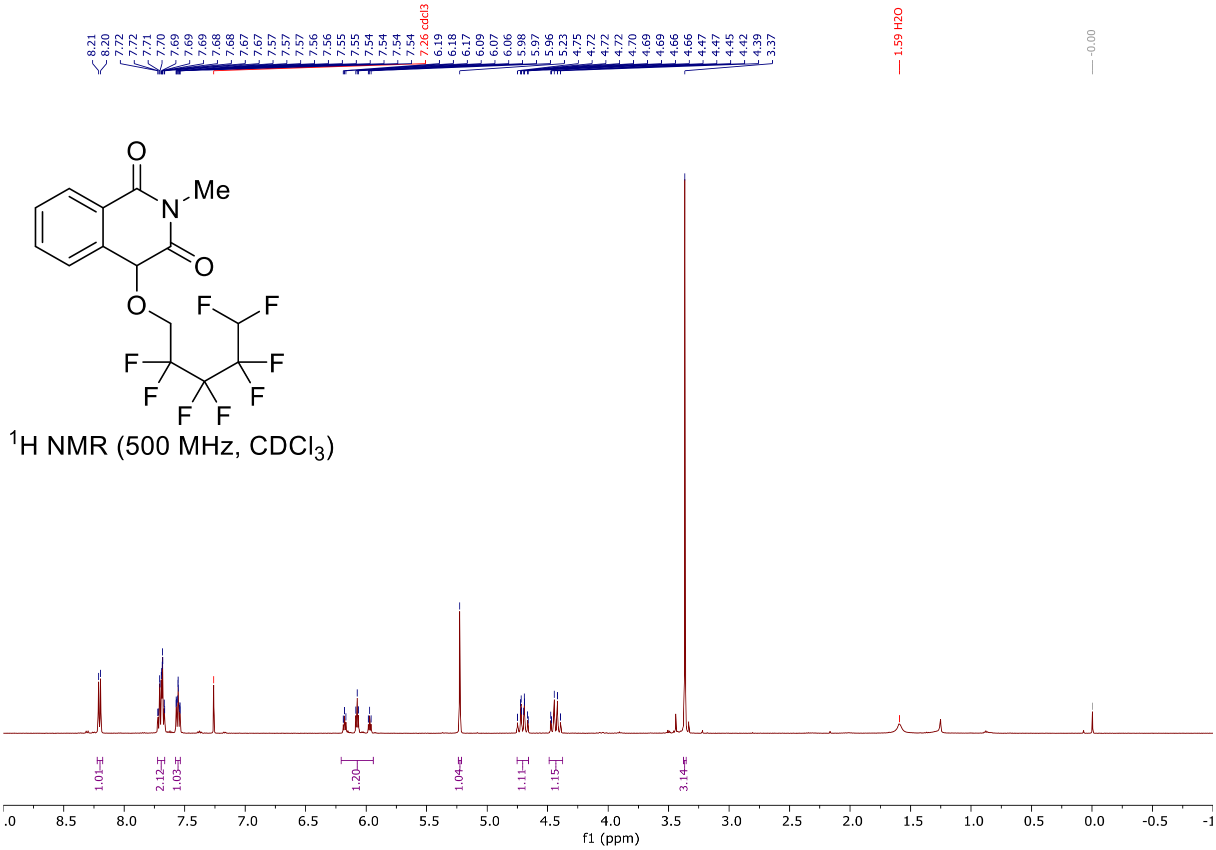

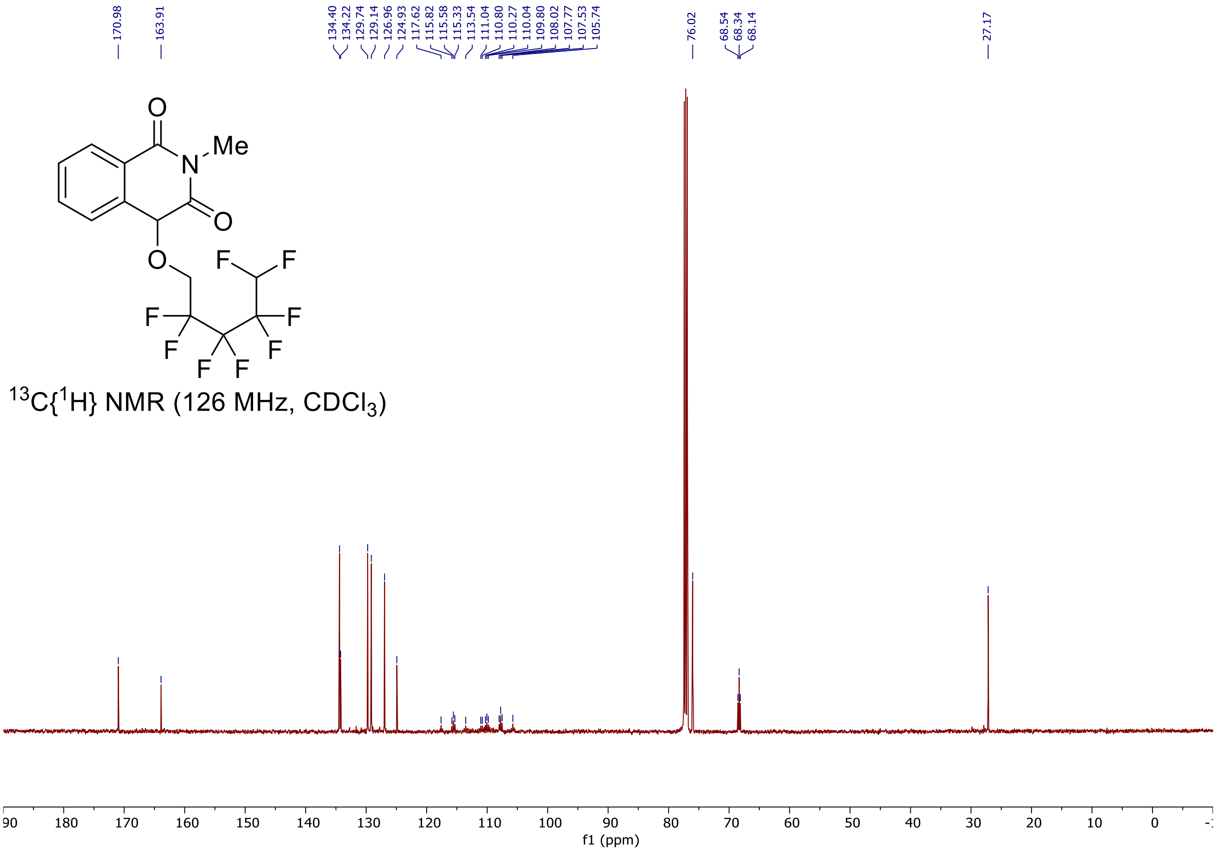

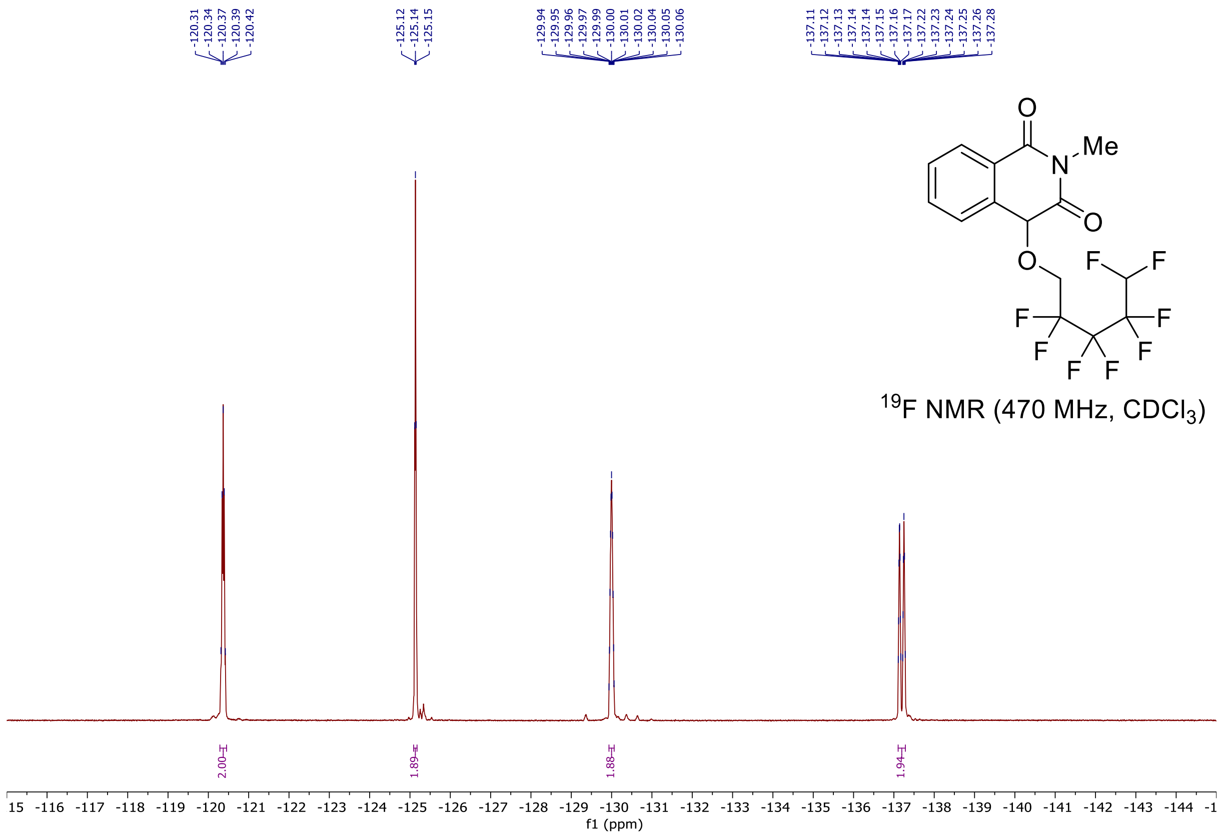
-120.31
-120.34
-120.37
-120.39
-120.42
-125.12
-125.14
-125.15
-129.94
-129.95
-129.96
-129.97
-129.99
-130.00
-130.01
-130.02
-130.04
-130.05
-130.06
-137.11
-137.12
-137.13
-137.14
-137.15
-137.16
-137.17
-137.22
-137.23
-137.24
-137.25
-137.26
-137.28
O
N
Me
O
O
F
F
F
F
F
F
F
F
19F NMR (470 MHz, CDCl3)
2.00
1.89
1.88
1.94
f1 (ppm)

2-methylisoquinoline-1,3,4(2*H*)-trione (**21b**)

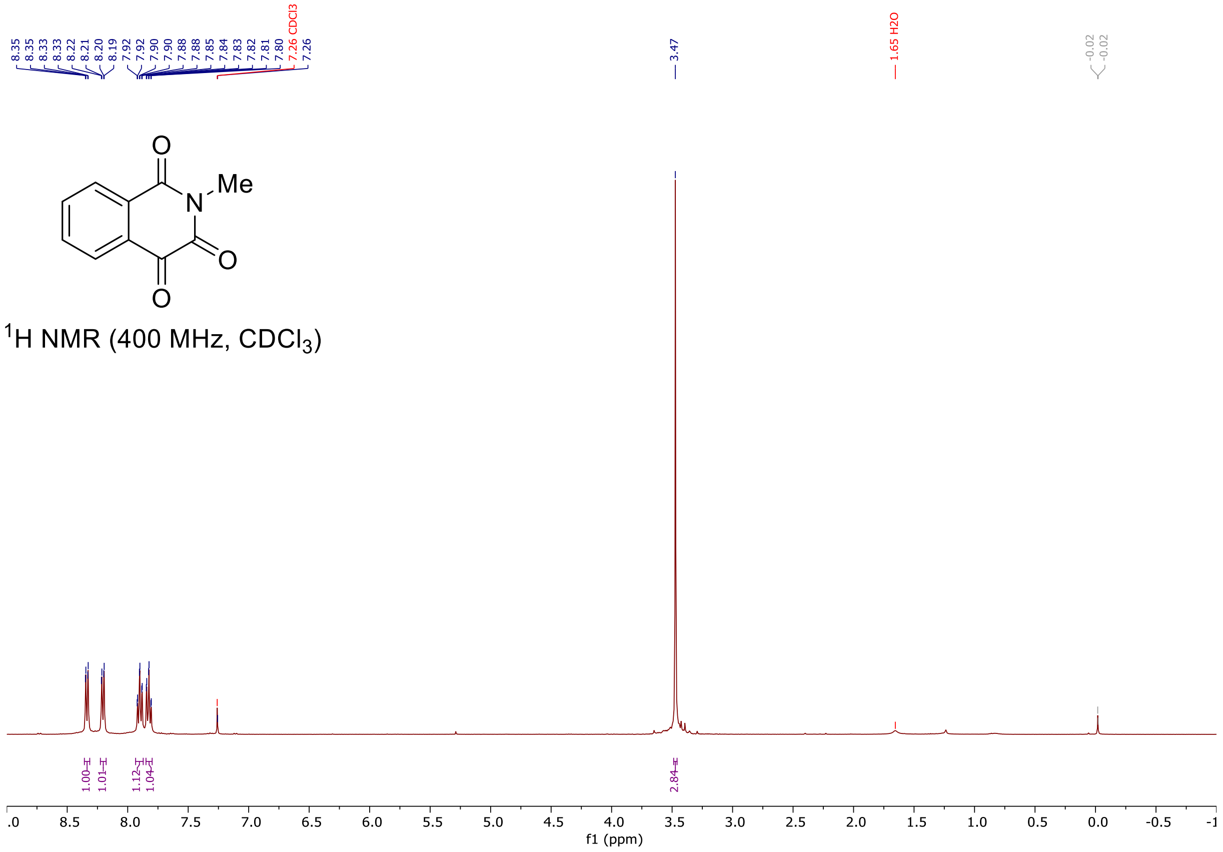

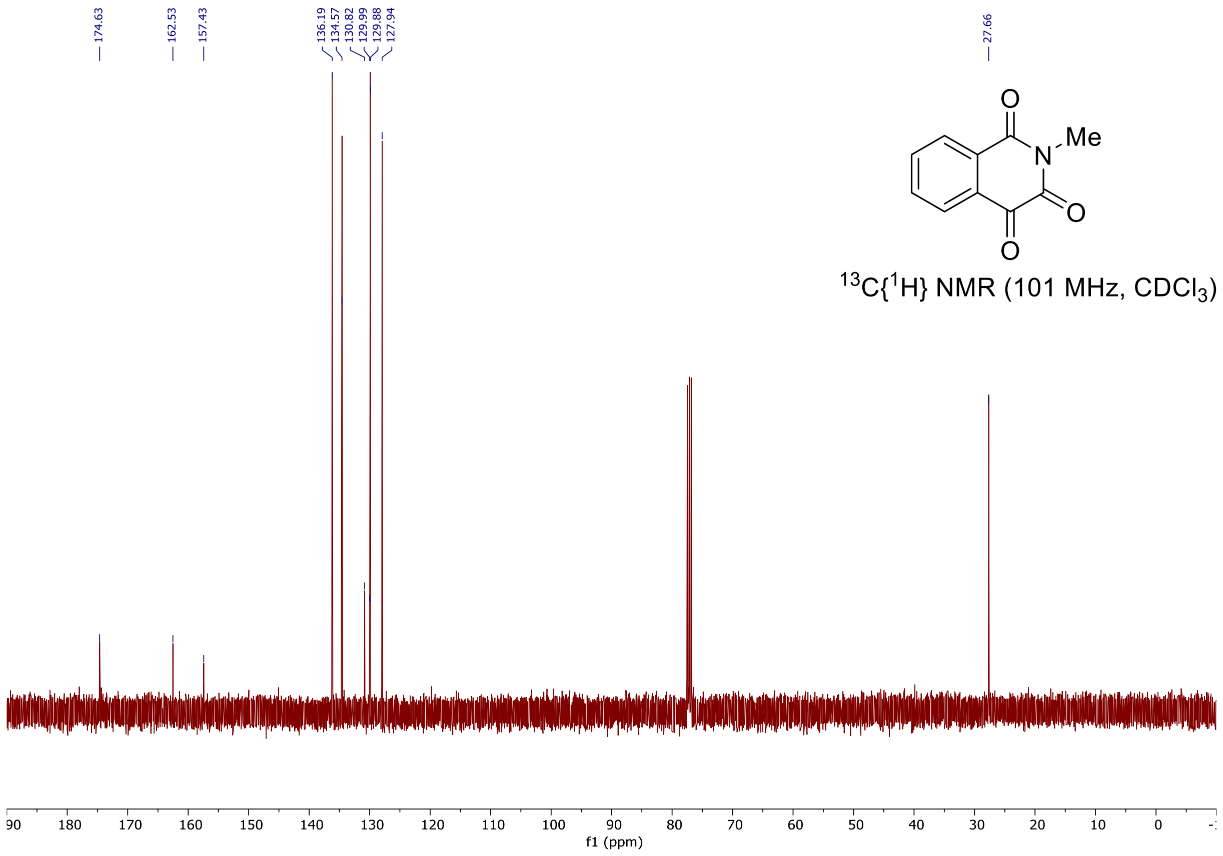

2-methyl-4-(phenethylthio)isoquinoline-1,3(2*H*,4*H*)-dione (**22**)

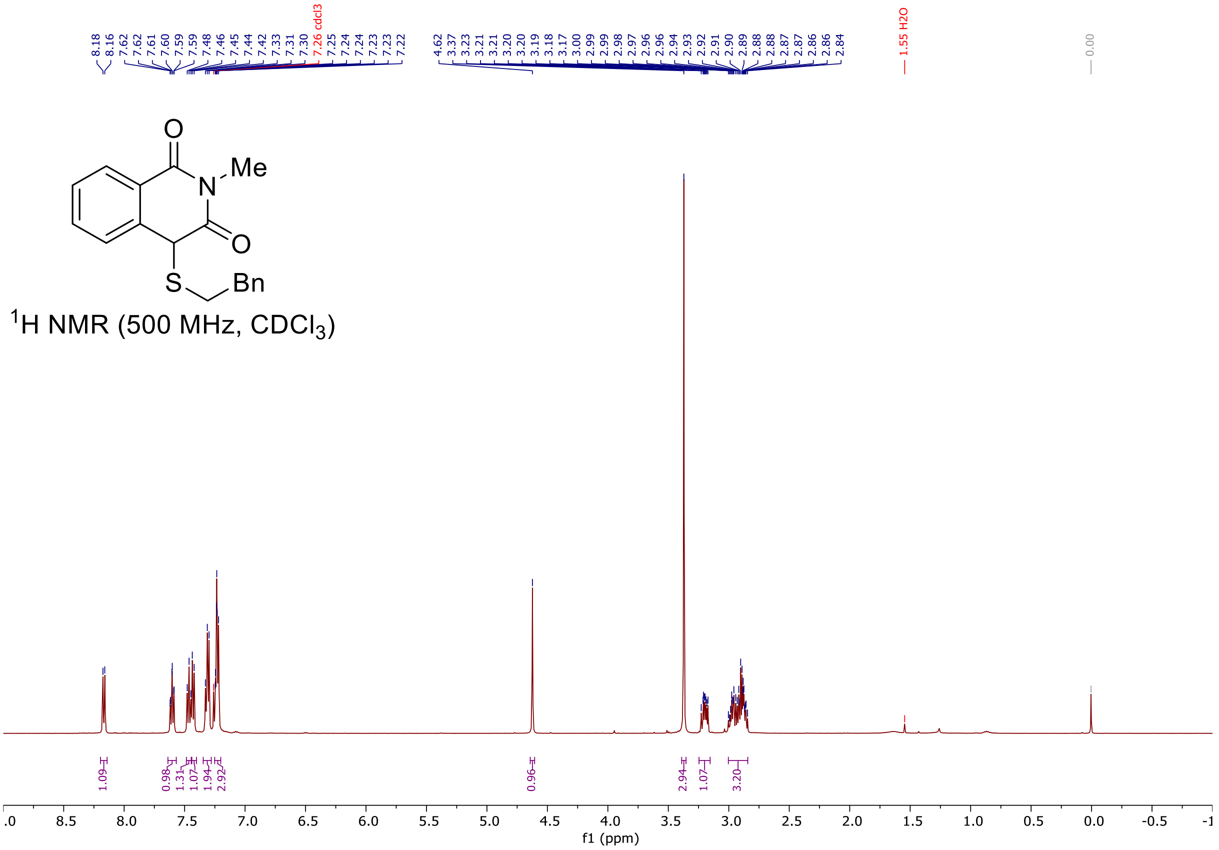

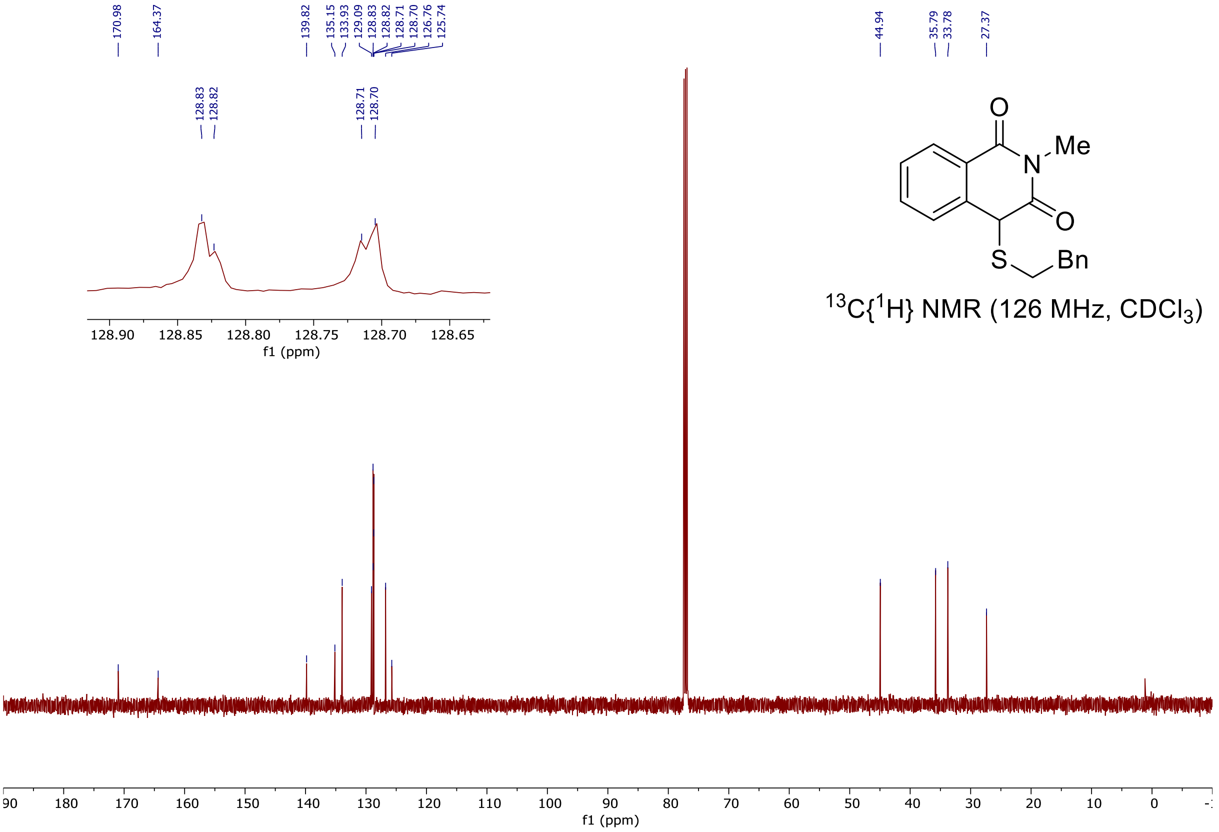

4-(cyclohexylthio)-2-methylisoquinoline-1,3(2*H*,4*H*)-dione (**23**)

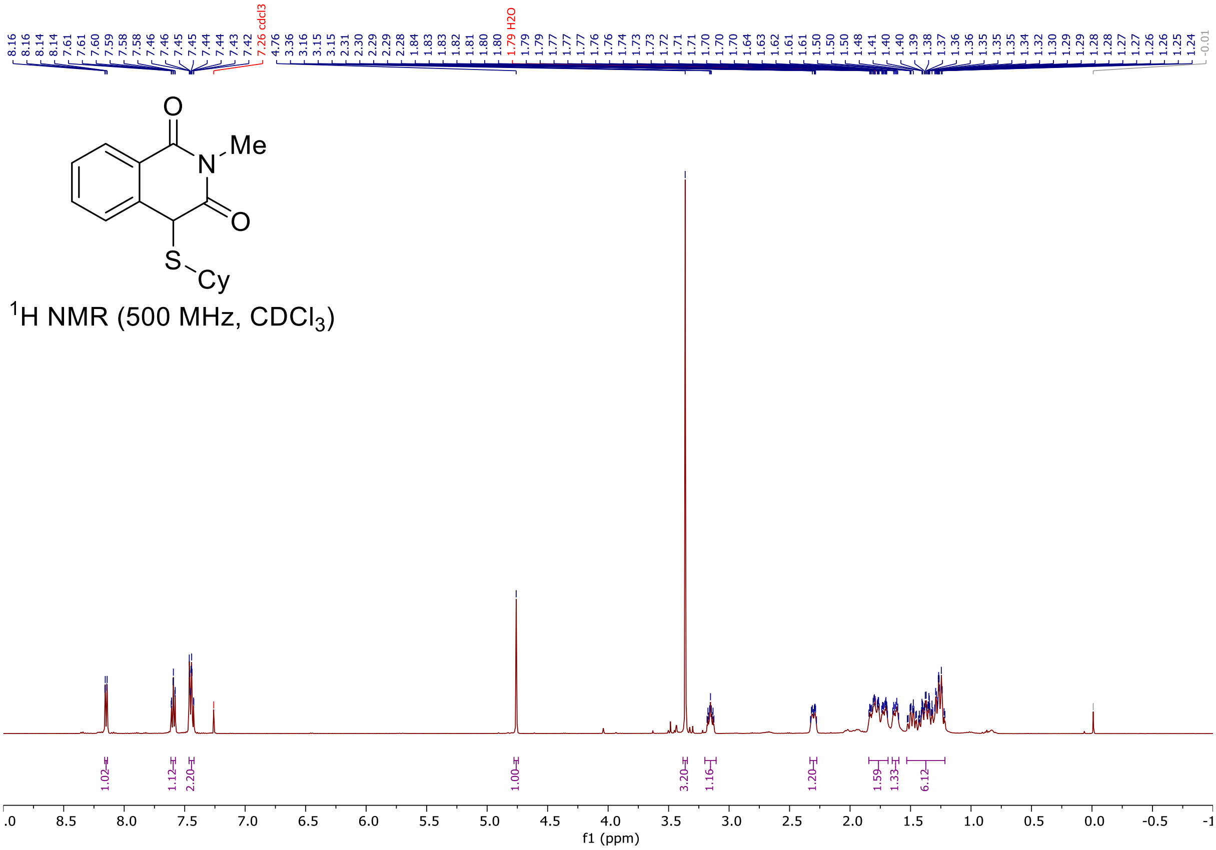

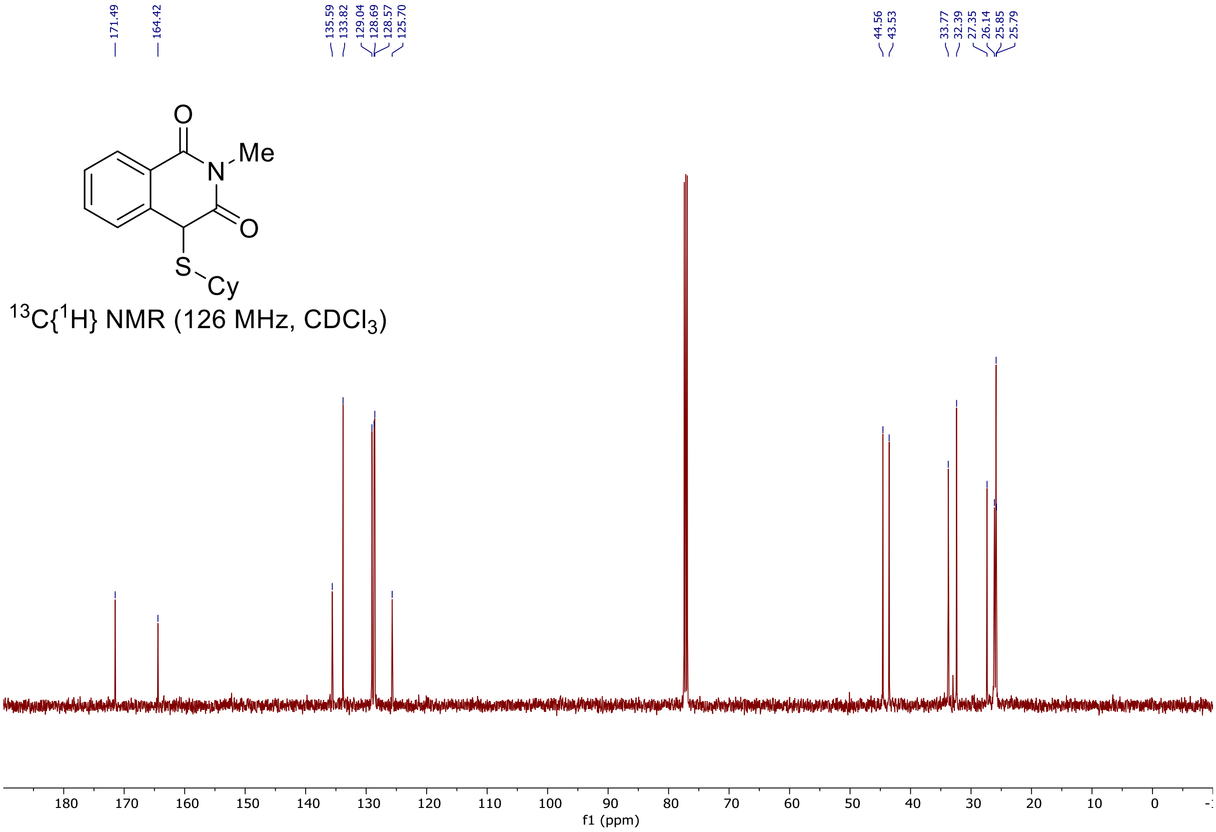

4-(benzylthio)-2-methylisoquinoline-1,3(2*H*,4*H*)-dione (**24**)

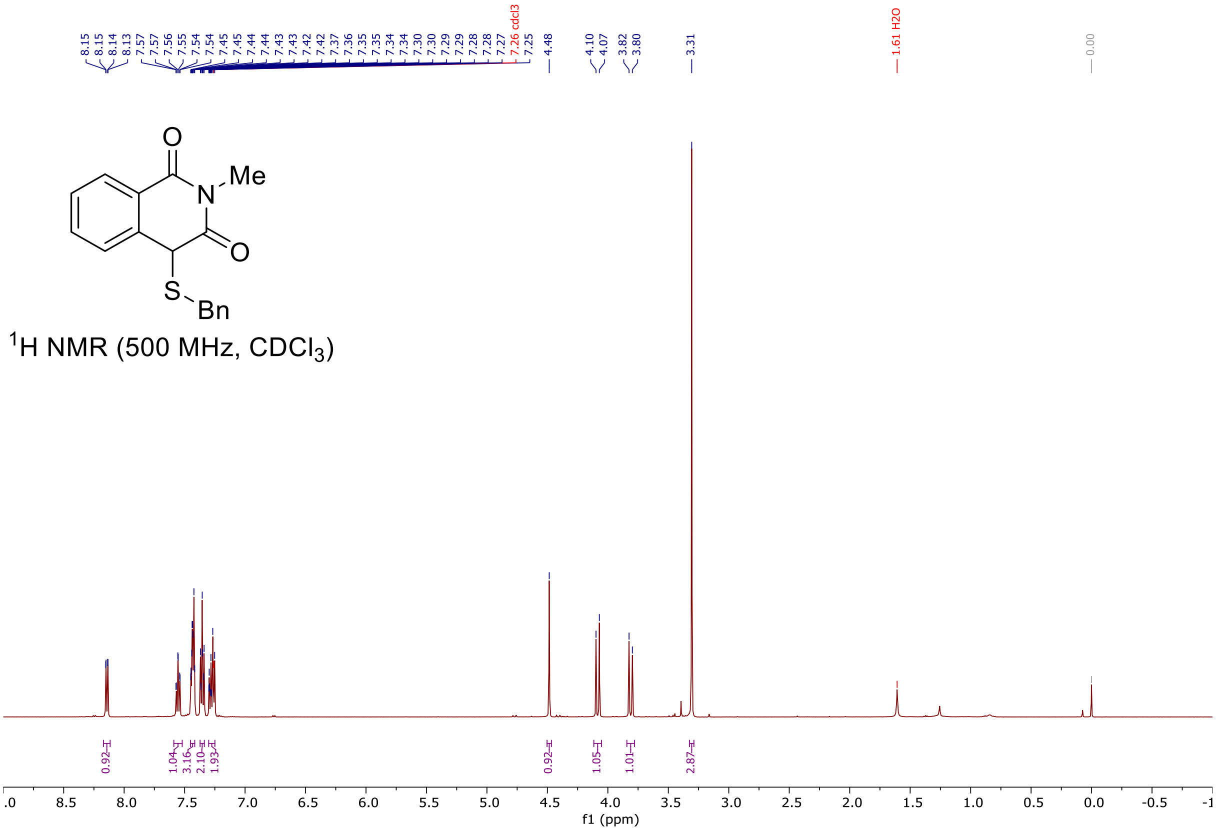

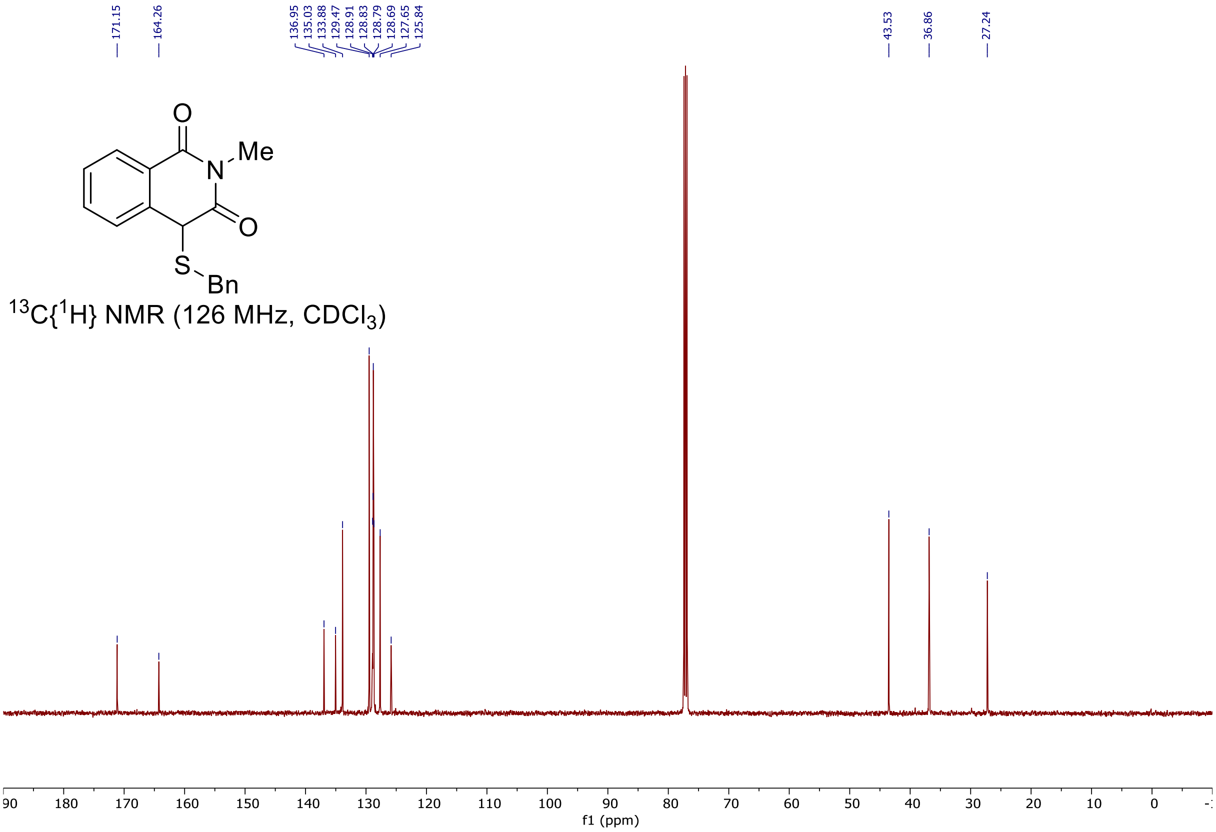

4-(dodecylthio)-2-methylisoquinoline-1,3(2*H*,4*H*)-dione (**25**)

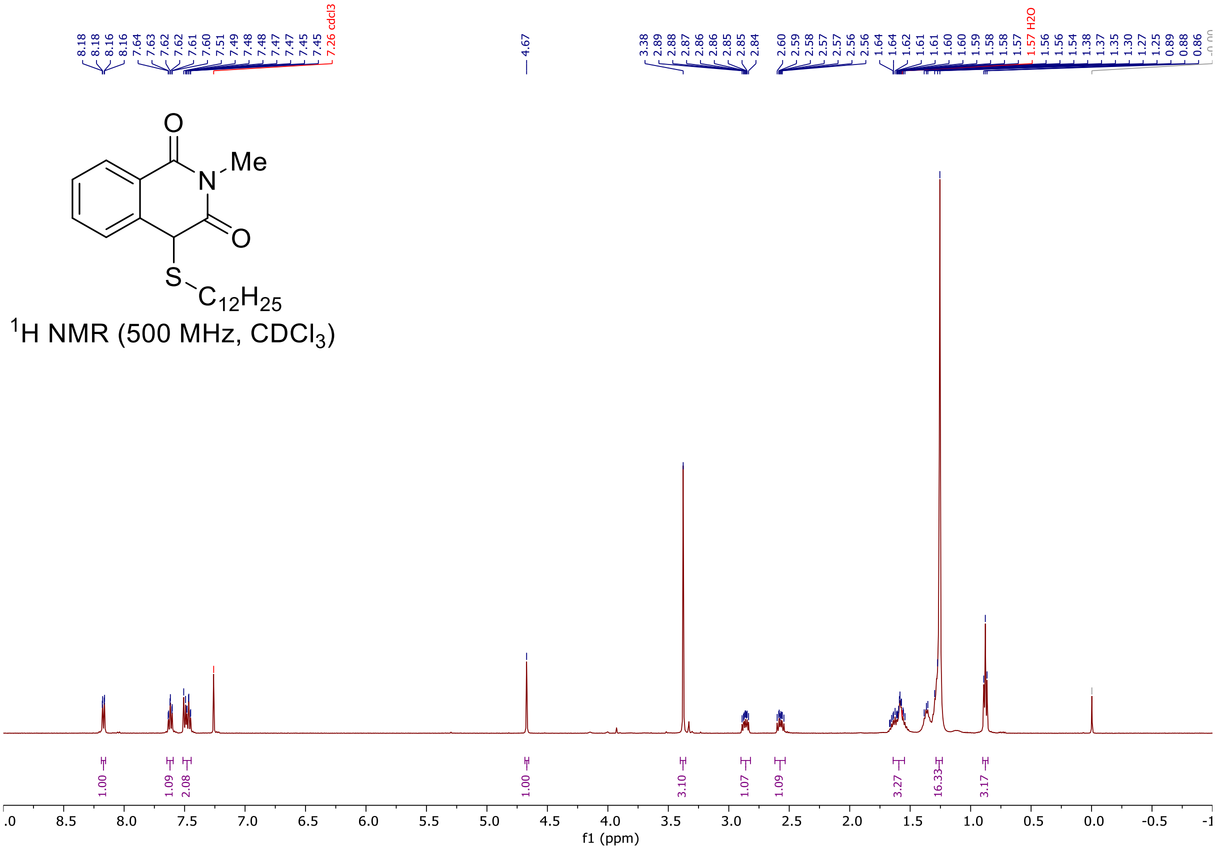

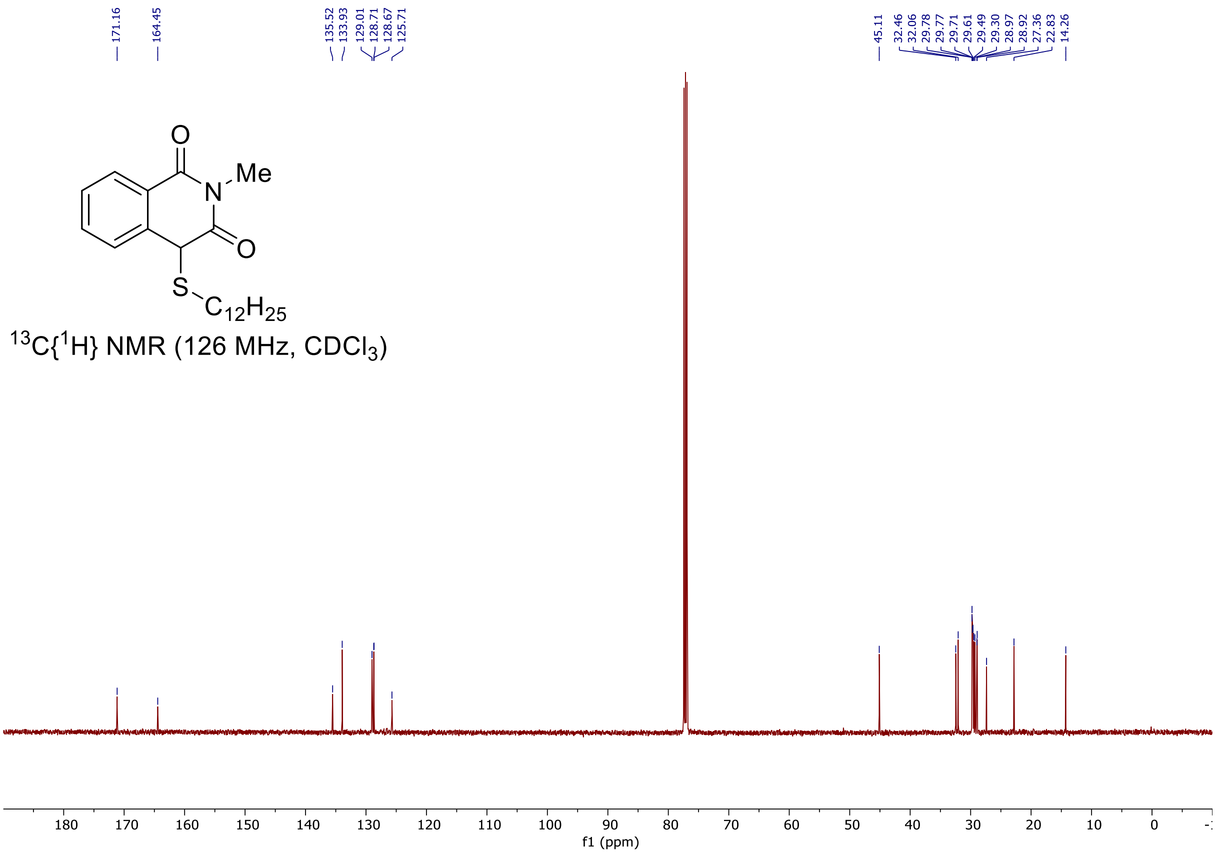

4-((2-hydroxyethyl)thio)-2-methylisoquinoline-1,3(2*H*,4*H*)-dione (**26**)

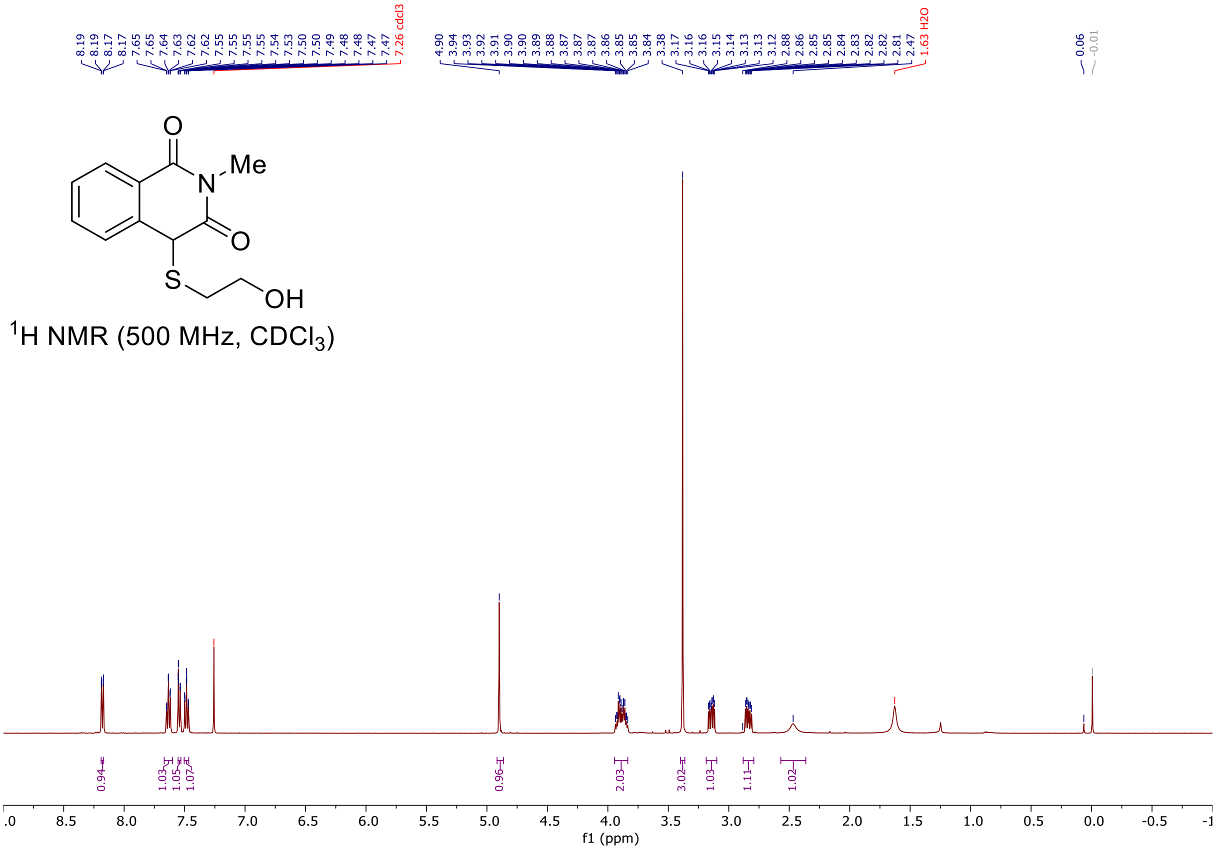

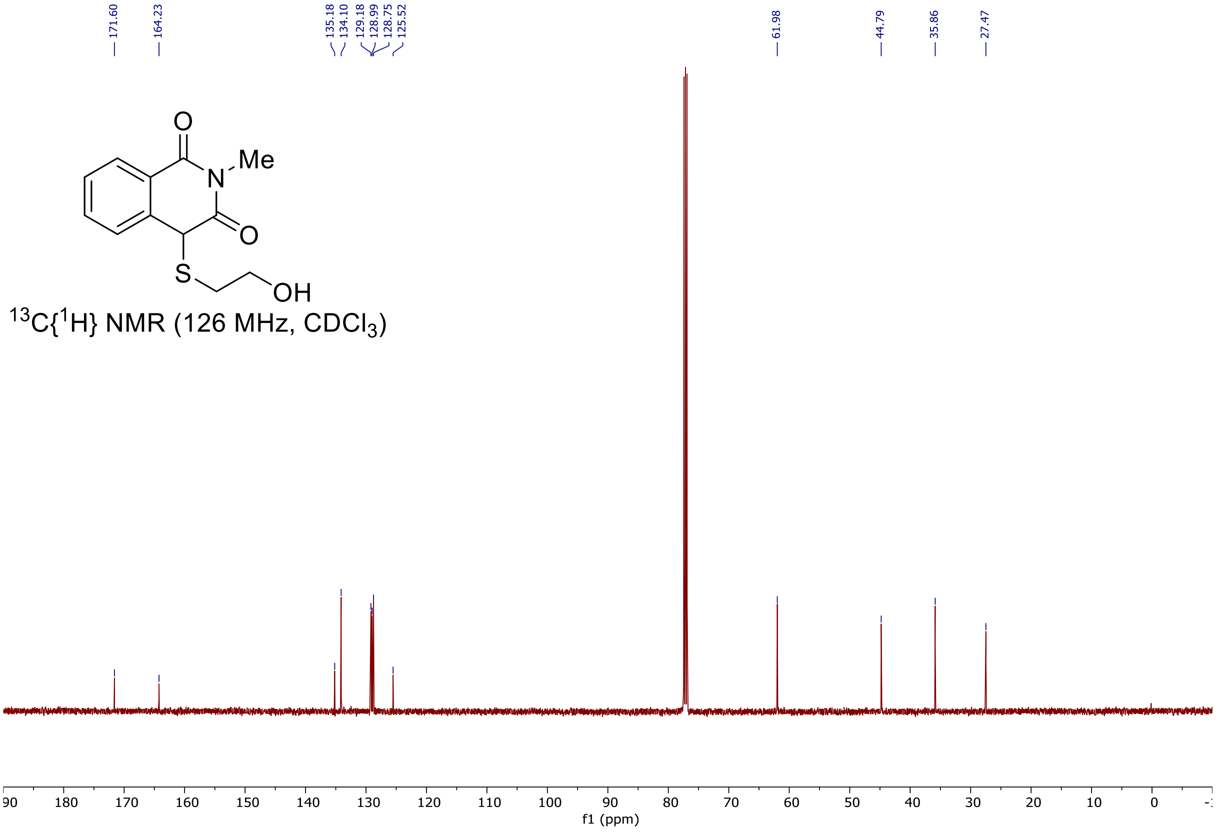

2-methyl-4-phenylisoquinoline-1,3(2*H*,4*H*)-dione (**28**)

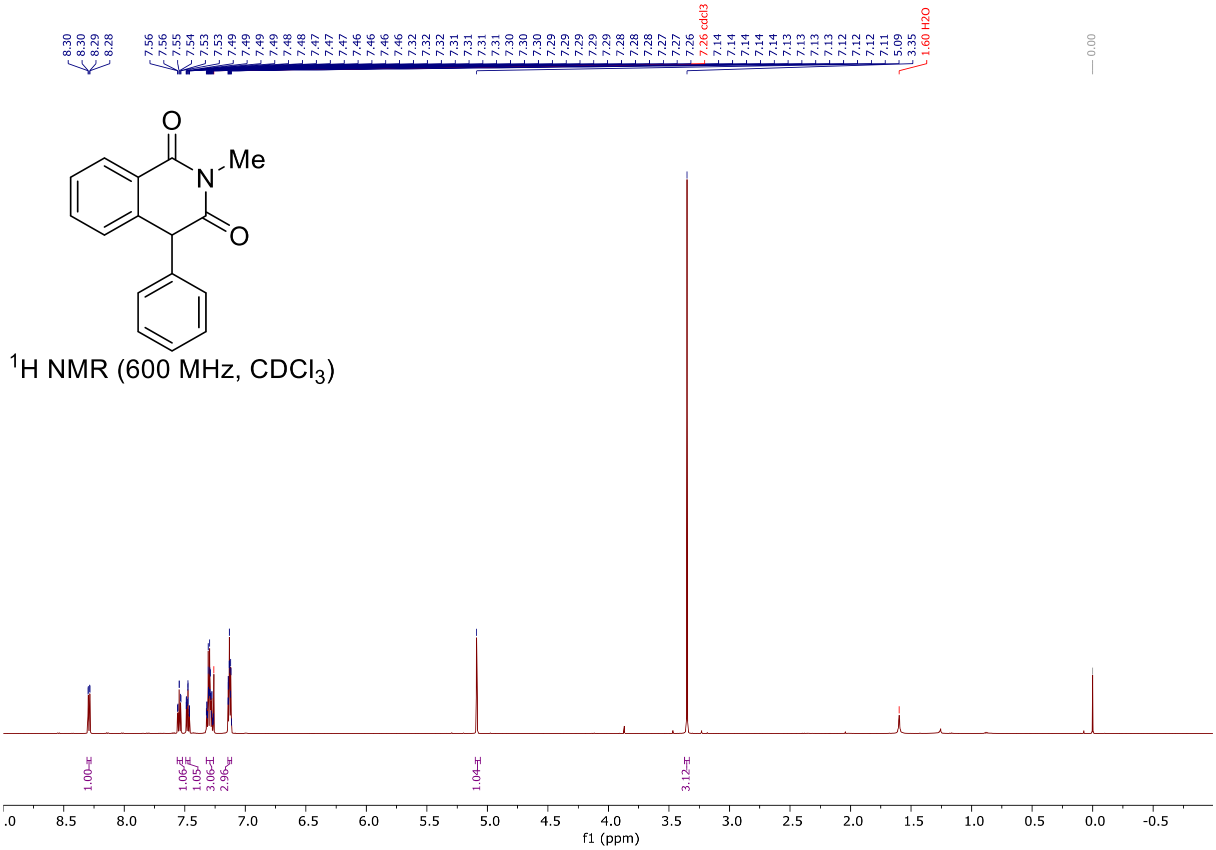

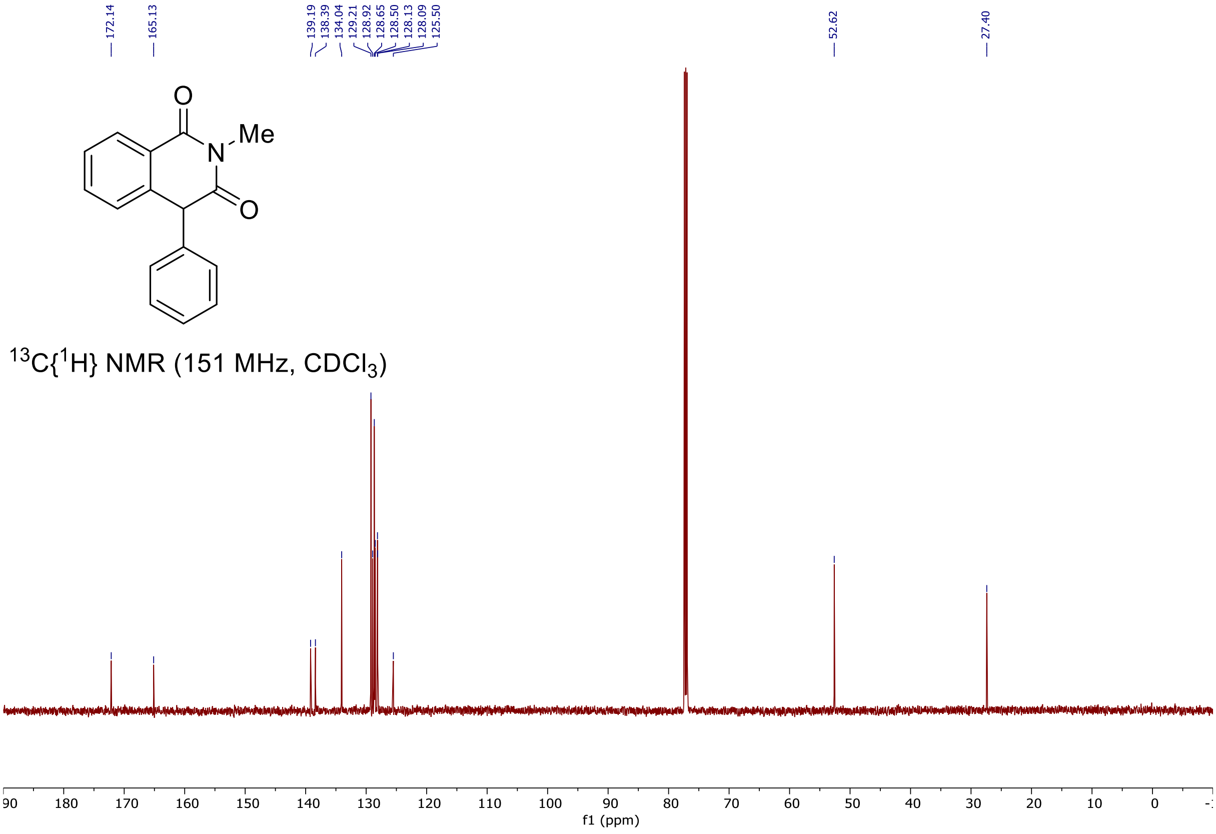

4-(2,5-dimethylphenyl)-2-methylisoquinoline-1,3(2*H*,4*H*)-dione (**29**)

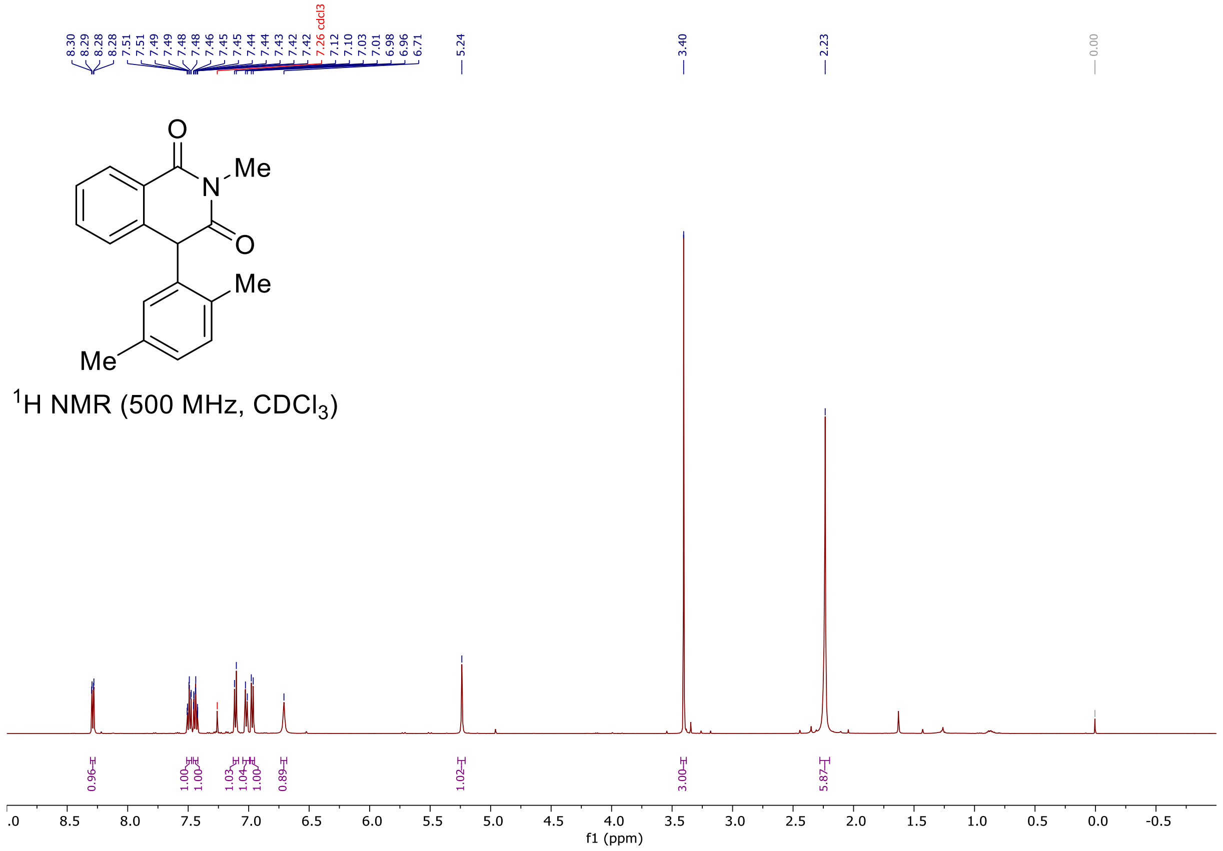

^{1}H NMR (500 MHz, $CDCl_3$)

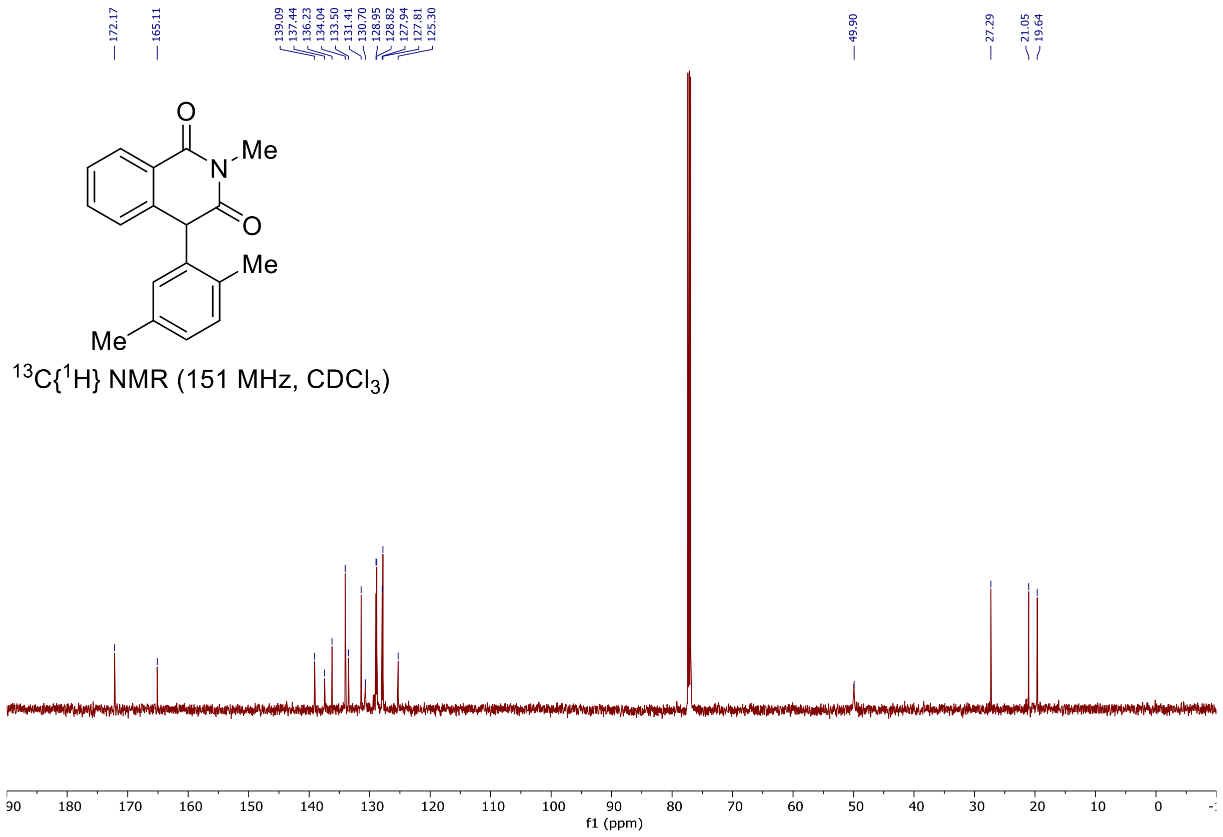

^{13}C{^{1}H} NMR (151 MHz, $CDCl_3$)

4-mesityl-2-methylisoquinoline-1,3(*2H,4H*)-dione (**30**)

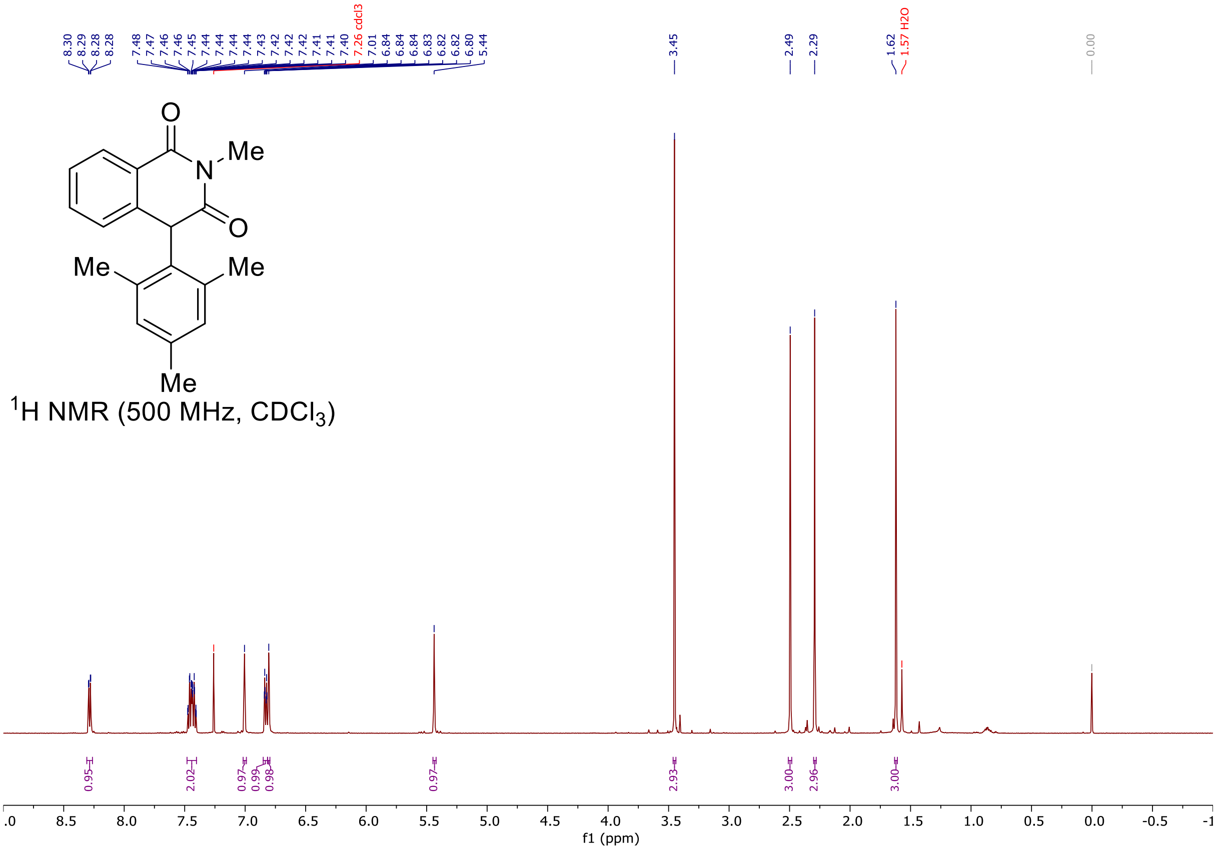

^{1}H NMR (500 MHz, $CDCl_3$)

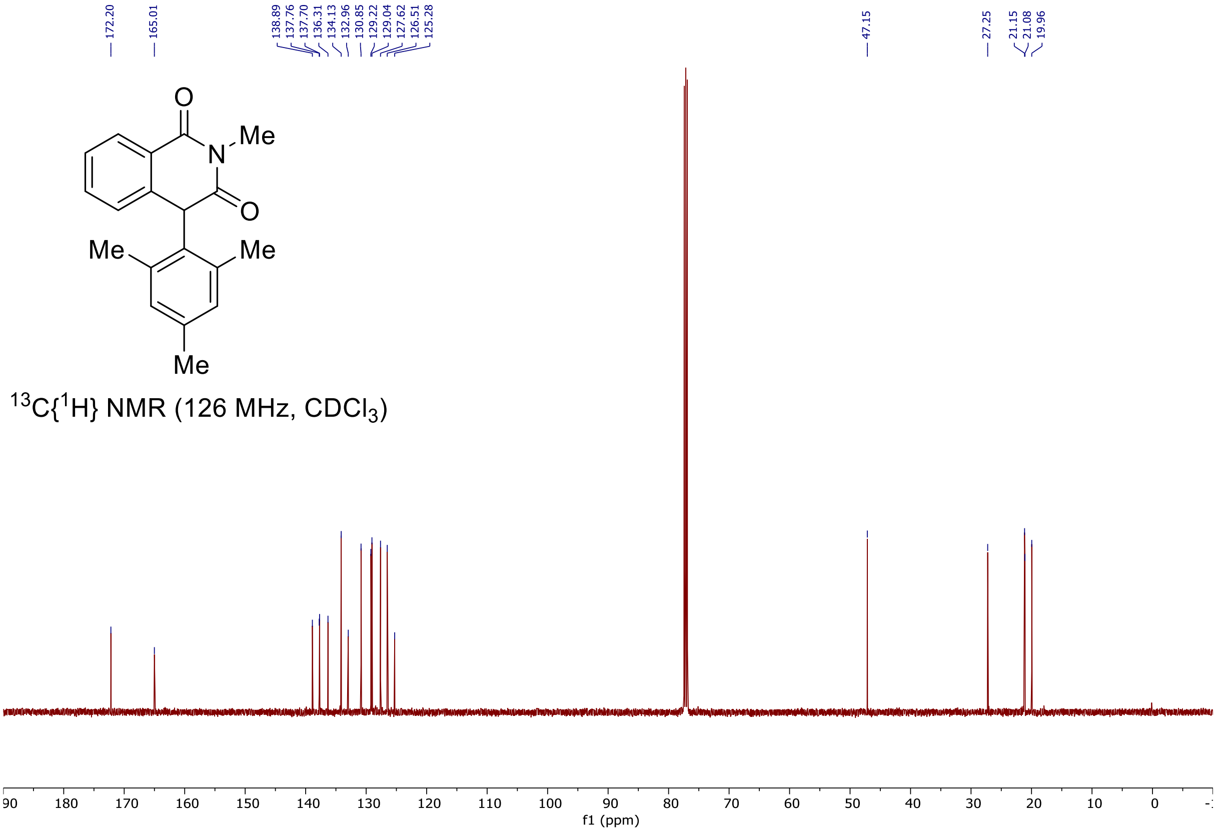

^{13}C{^{1}H} NMR (126 MHz, $CDCl_3$)

4-(5-bromo-2-methoxyphenyl)-2-methylisoquinoline-1,3(2*H*,4*H*)-dione (**31**)

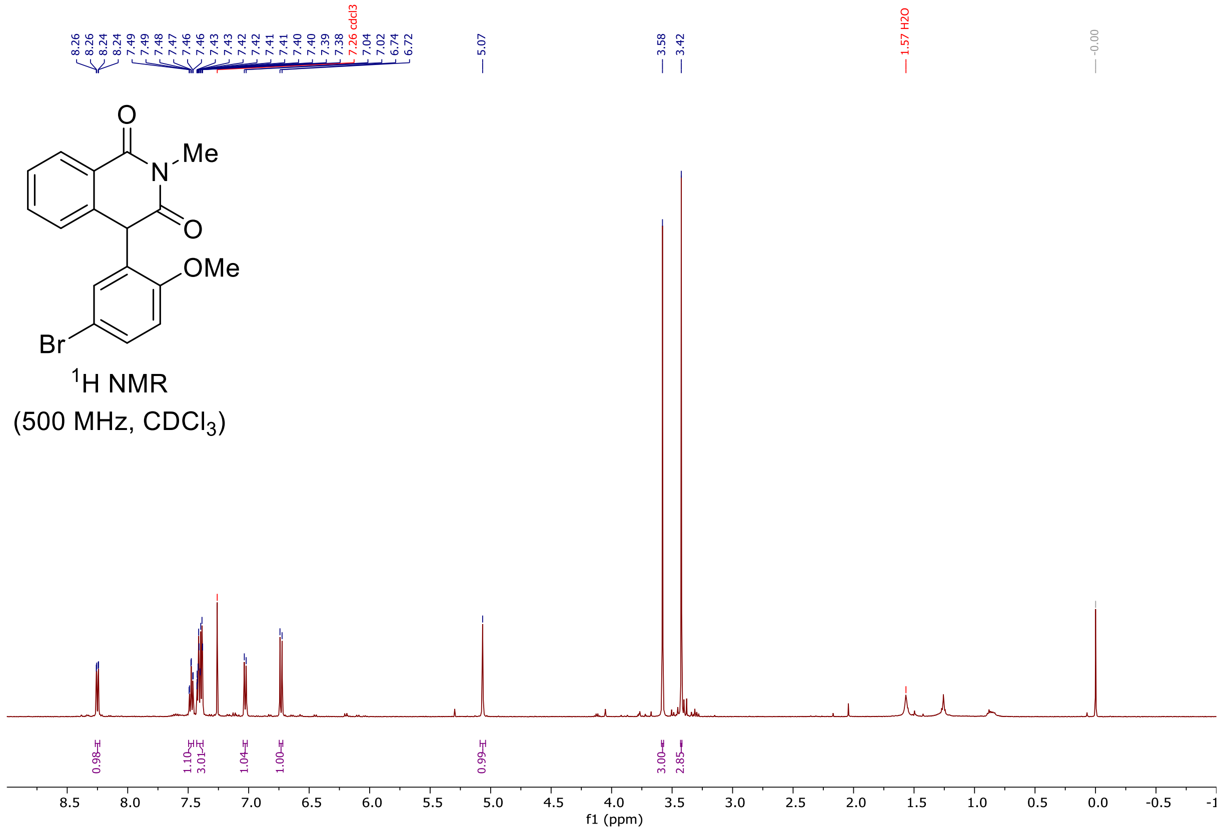

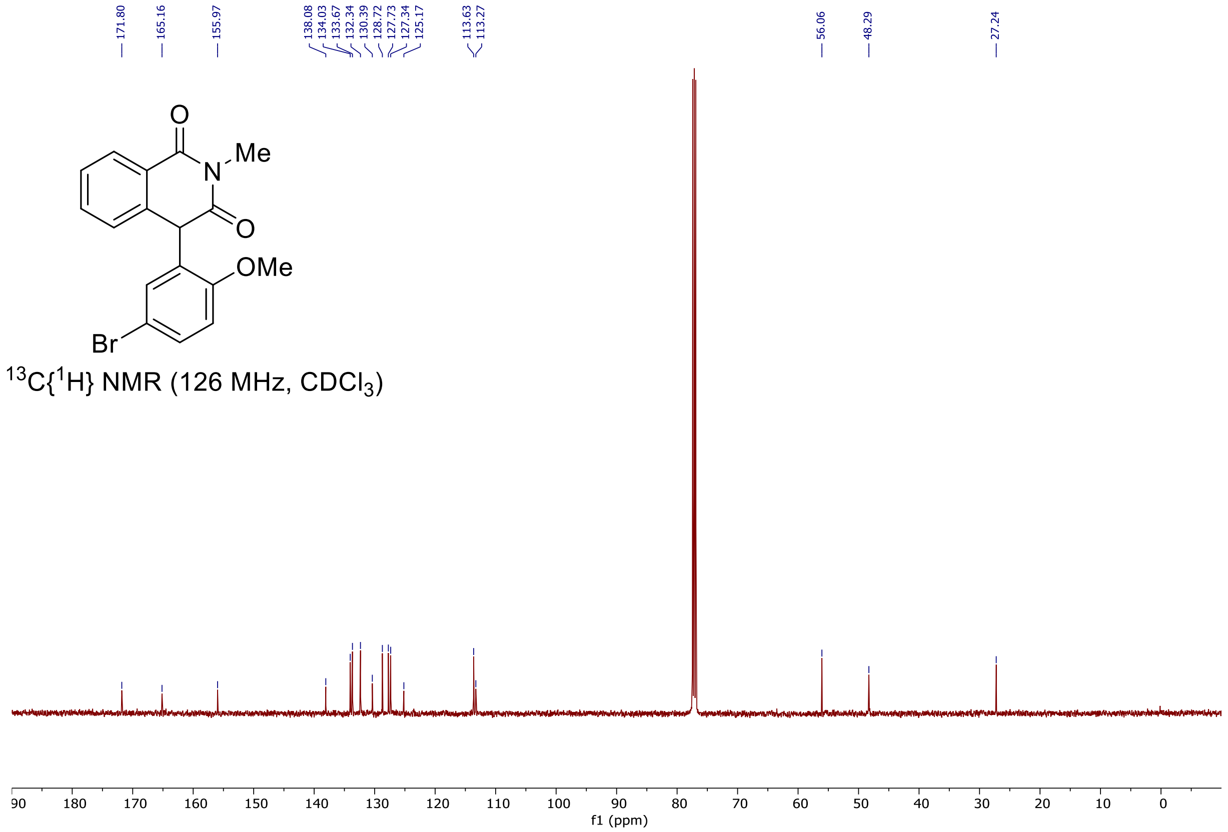

4-(2,4-dimethoxyphenyl)-2-methylisoquinoline-1,3(2*H*,4*H*)-dione (**32**) and 4-(2,6-dimethoxyphenyl)-2-methylisoquinoline-1,3(2*H*,4*H*)-dione (**33**) (2:1 rr)

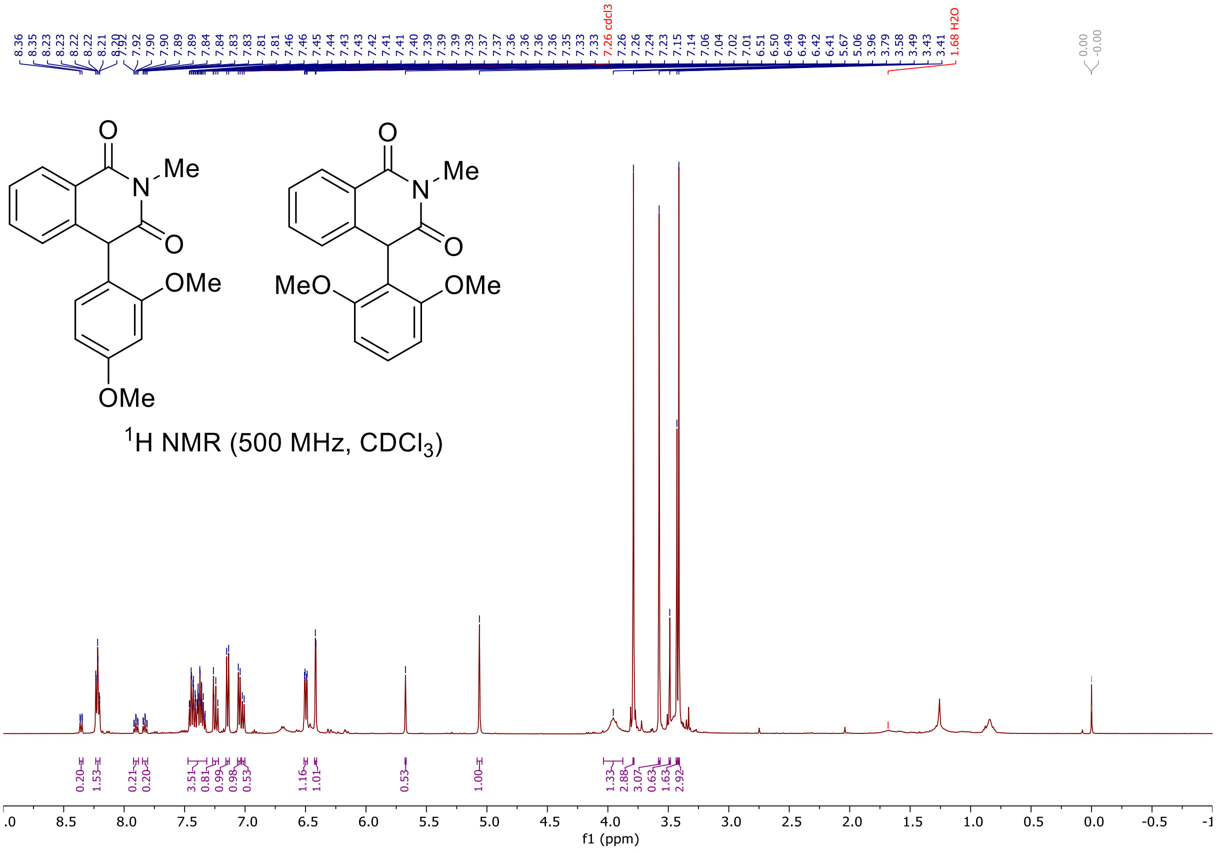

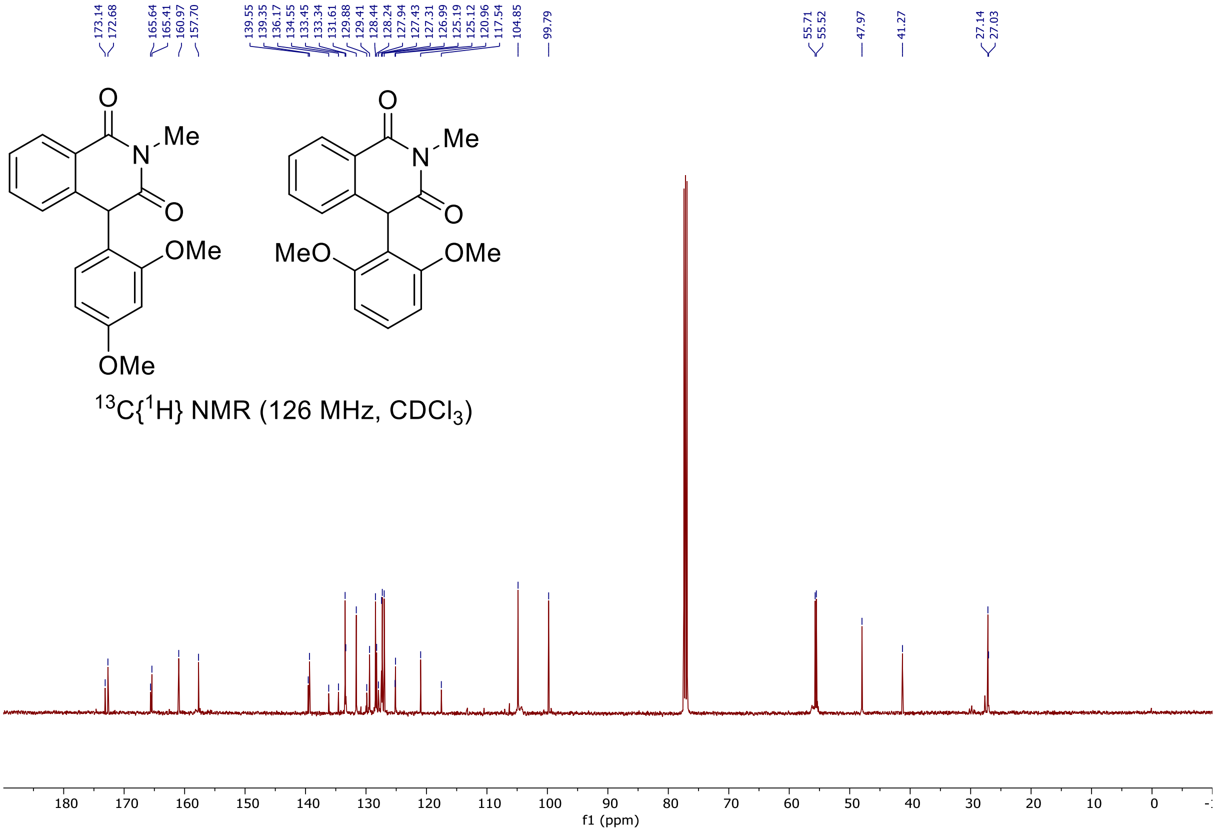

4-((1,1,1,3,3,3-hexafluoropropan-2-yl)oxy)-2-methyl-2H-benzo[*e*][1,2]thiazin-3(4*H*)-one 1,1-dioxide (**35**)

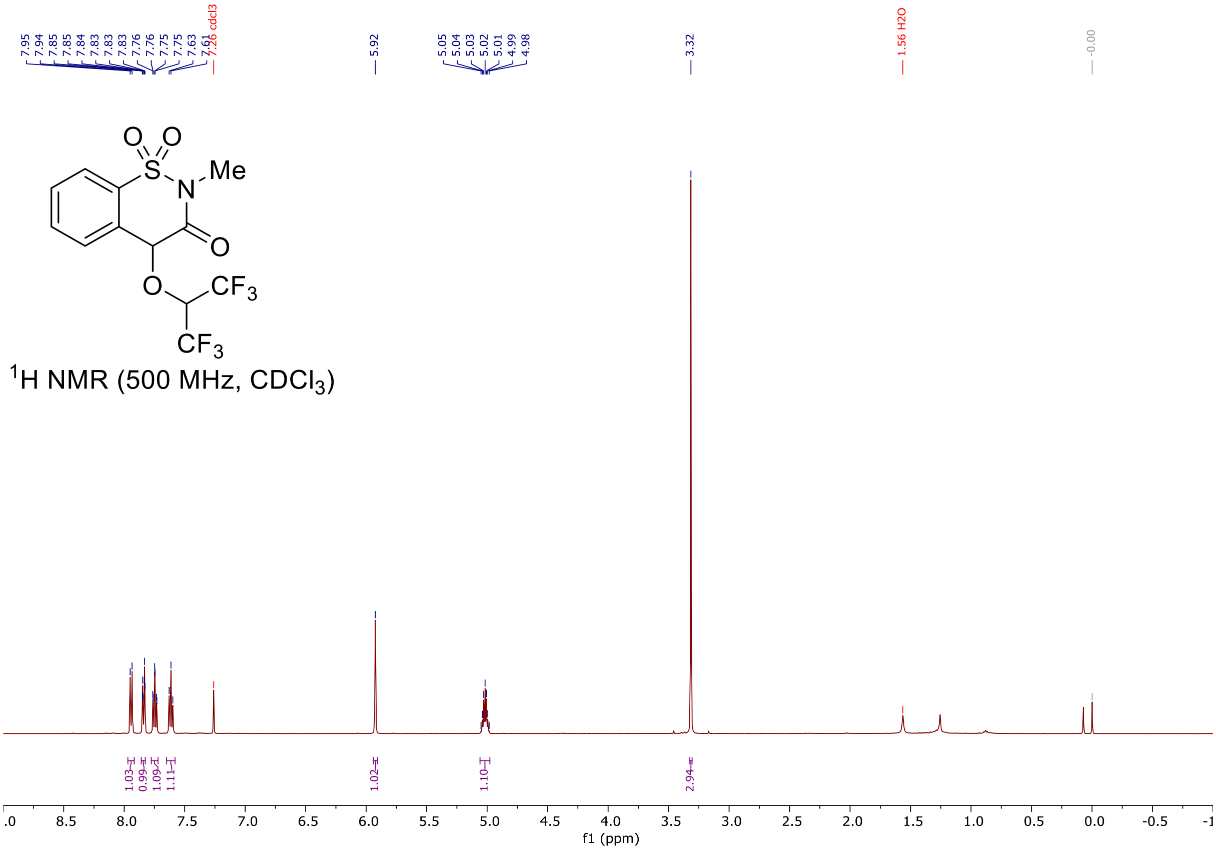

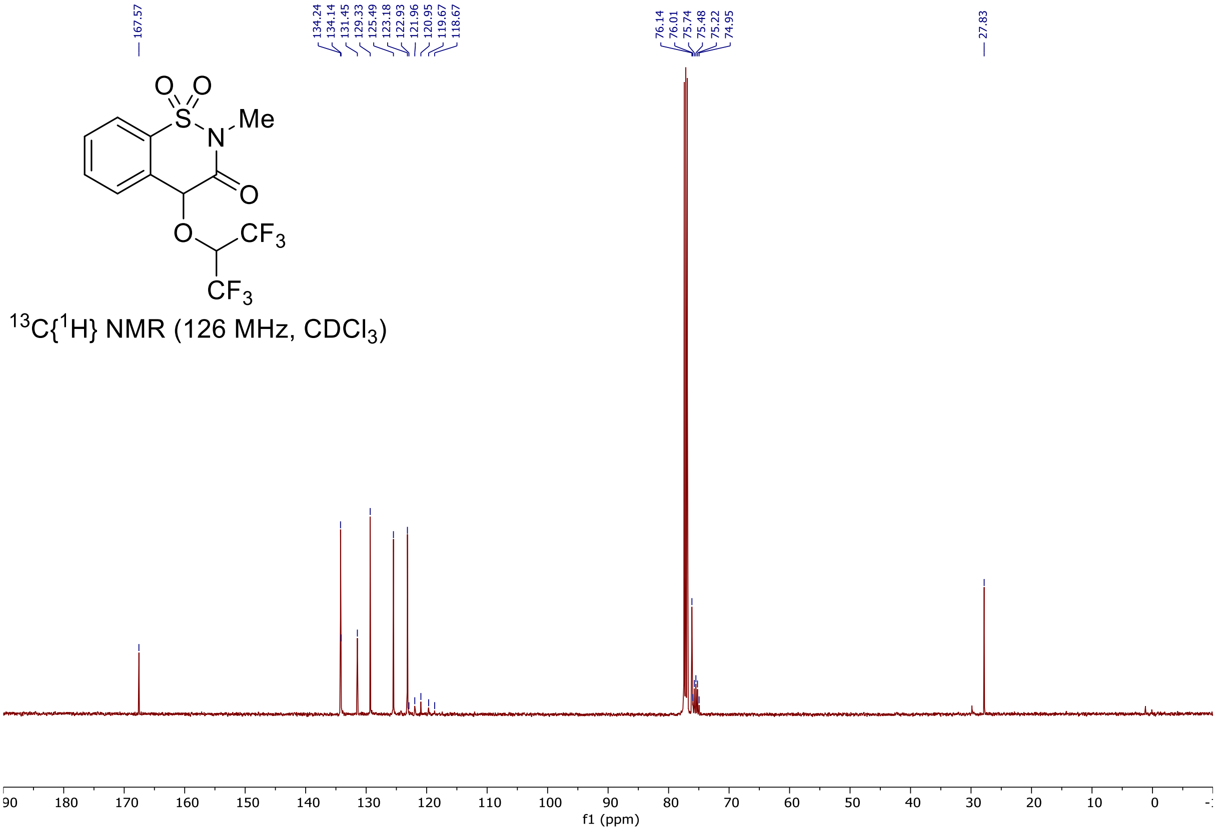

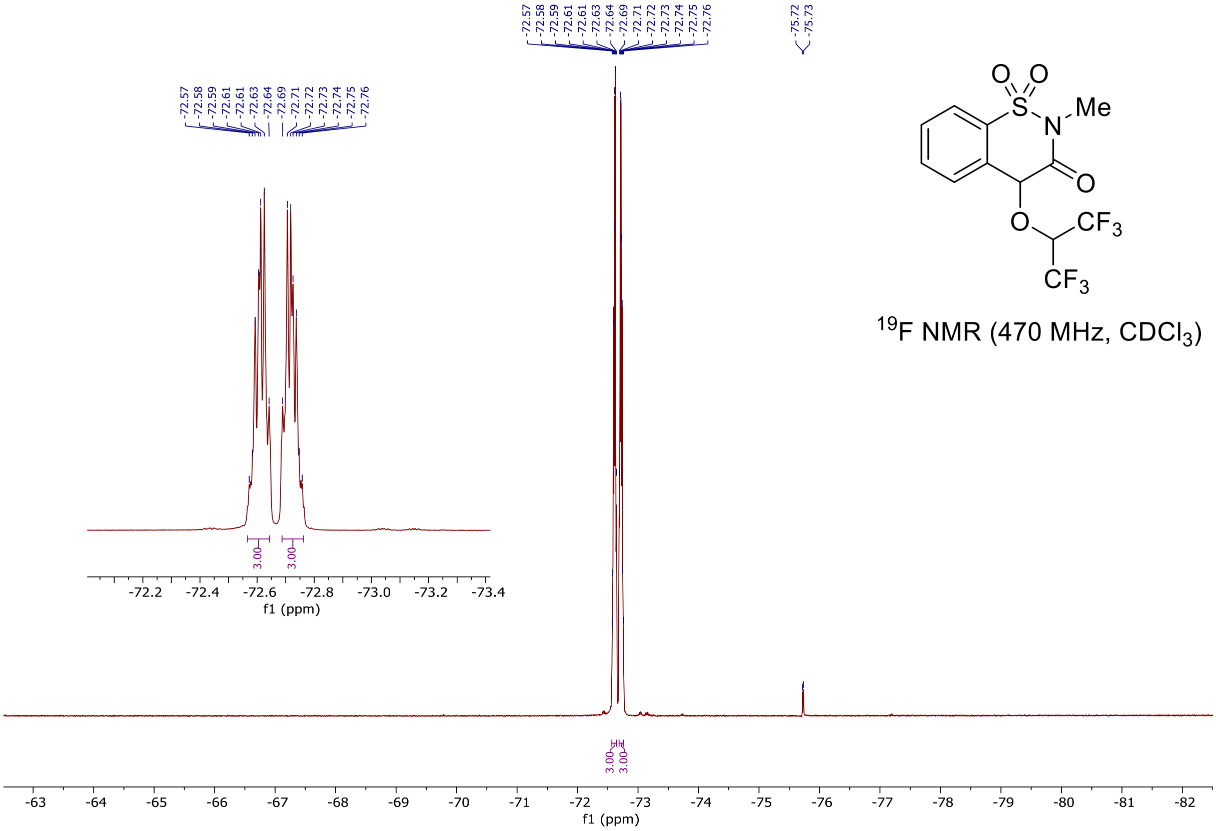

2-benzyl-4-((1,1,1,3,3,3-hexafluoropropan-2-yl)oxy)-2H-benzo[e][1,2]thiazin-3(4H)-one 1,1-dioxide (**36**)

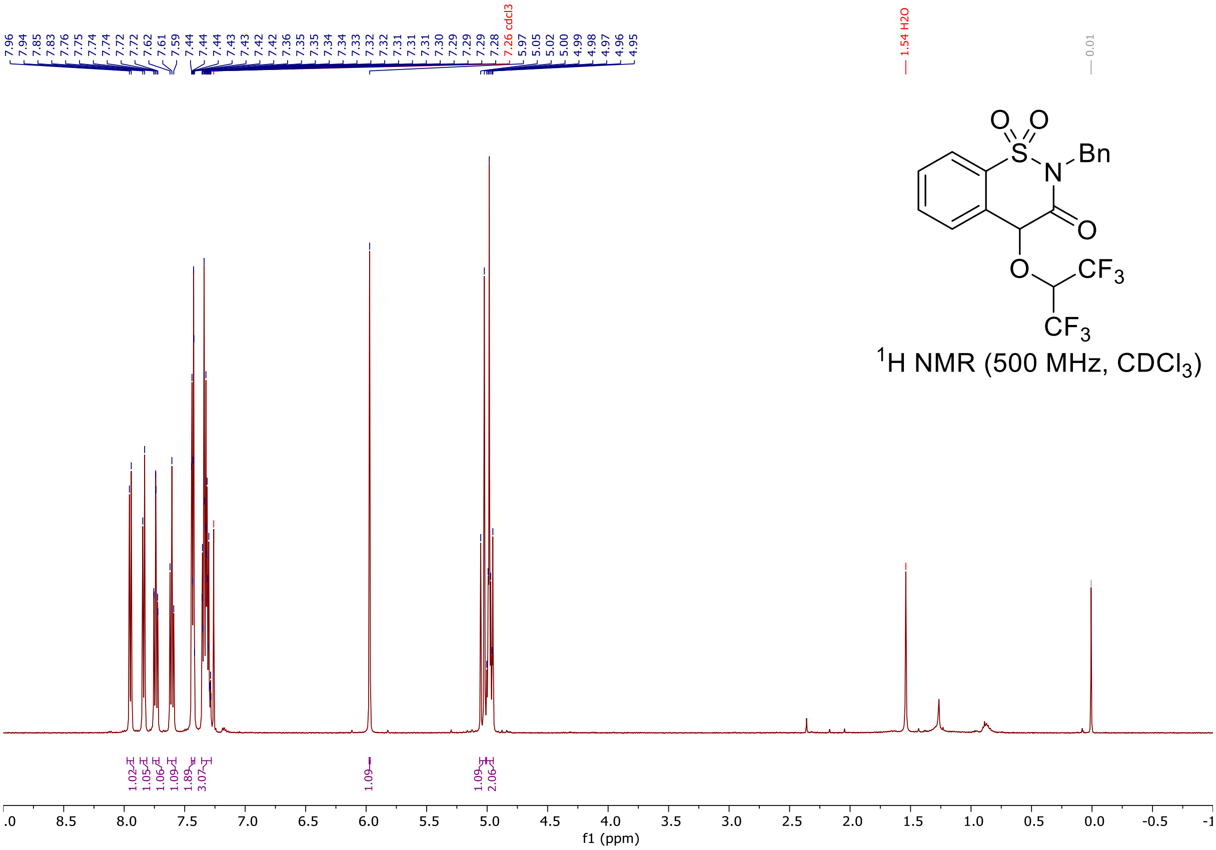

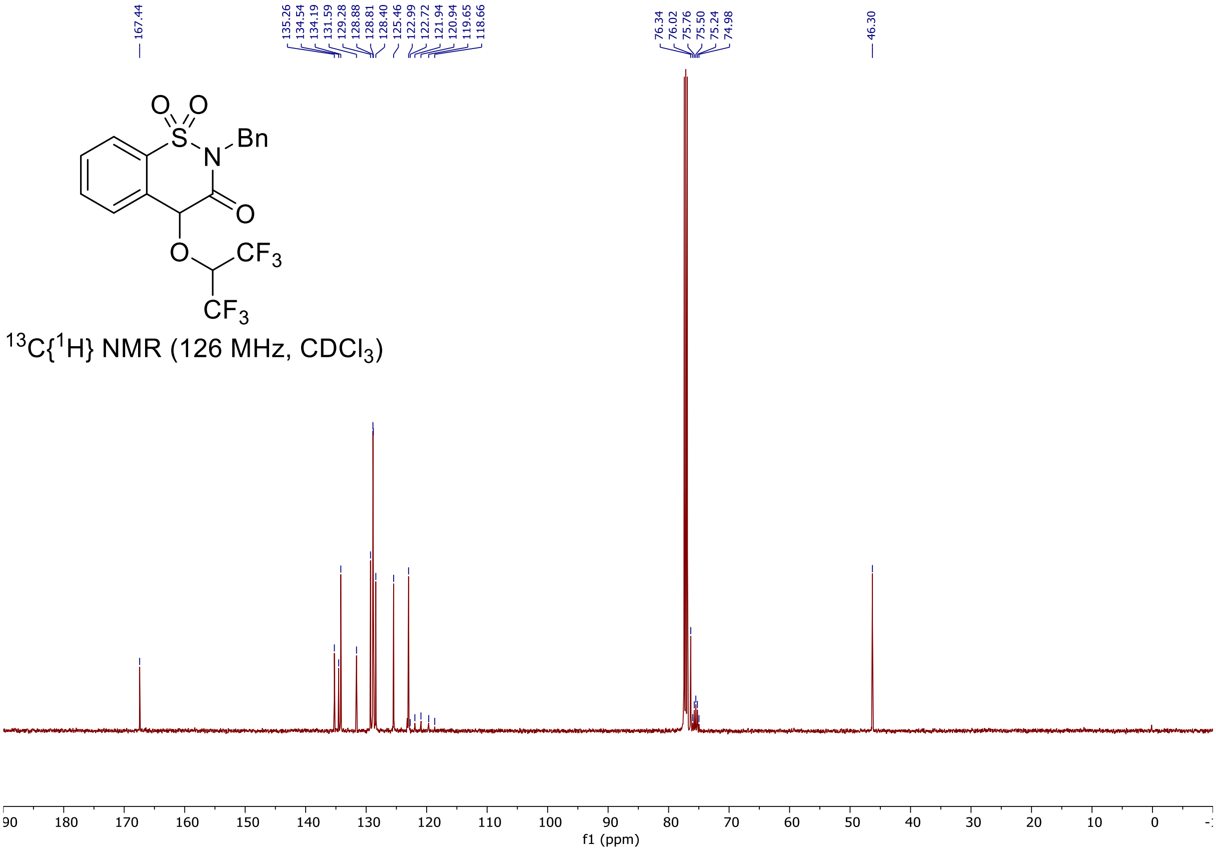
167.44
135.26
134.54
134.19
131.59
129.28
128.88
128.81
128.40
125.46
122.99
122.72
121.94
120.94
119.65
118.66
76.34
76.02
75.76
75.50
75.24
74.98
46.30
O
O
S
N
Bn
O
O
CF3
CF3
13C{1H} NMR (126 MHz, CDCl3)
f1 (ppm)

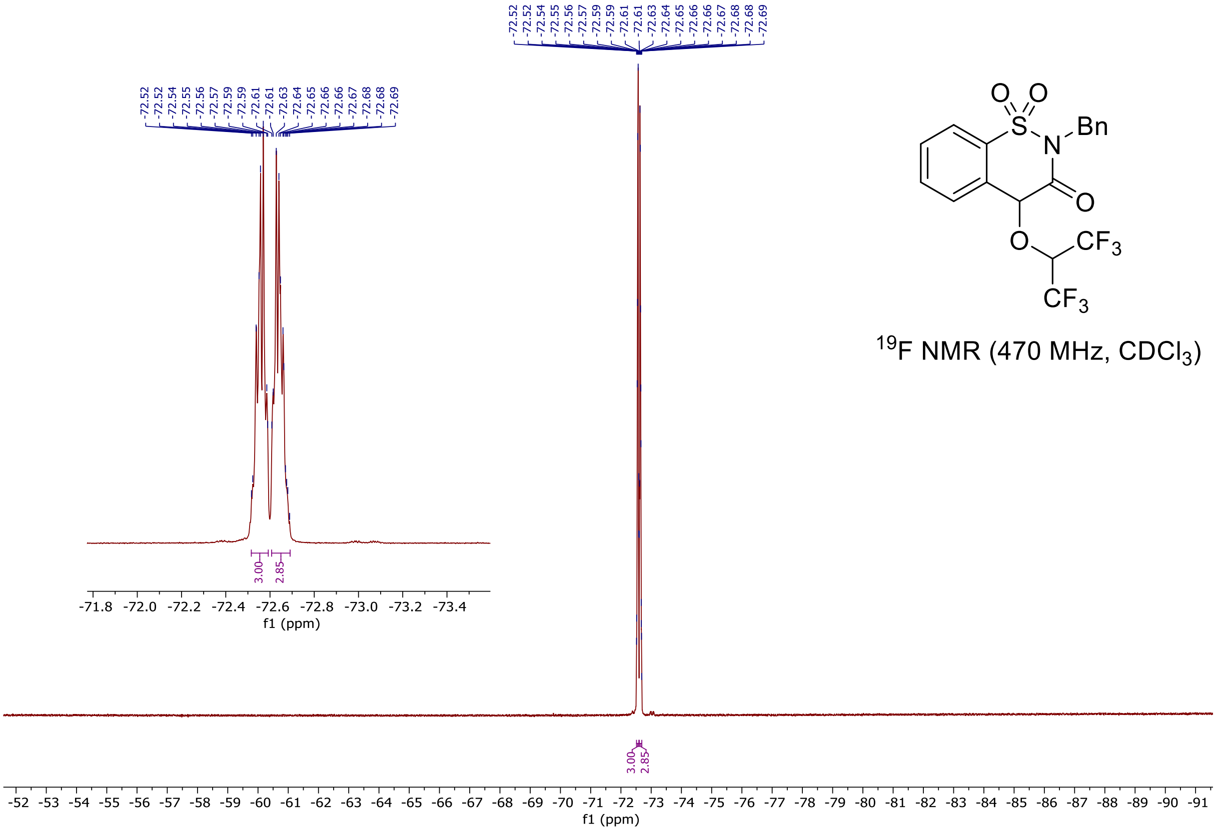
-72.52
-72.52
-72.54
-72.55
-72.56
-72.57
-72.59
-72.59
-72.61
-72.61
-72.63
-72.64
-72.65
-72.66
-72.66
-72.67
-72.68
-72.68
-72.69
3.00
2.85
f1 (ppm)
O
O
S
N
Bn
O
O
CF3
CF3
19F NMR (470 MHz, CDCl3)

2-allyl-4-((1,1,1,3,3,3-hexafluoropropan-2-yl)oxy)-2*H*-benzo[e][1,2]thiazin-3(4*H*)-one 1,1-dioxide (**37**)

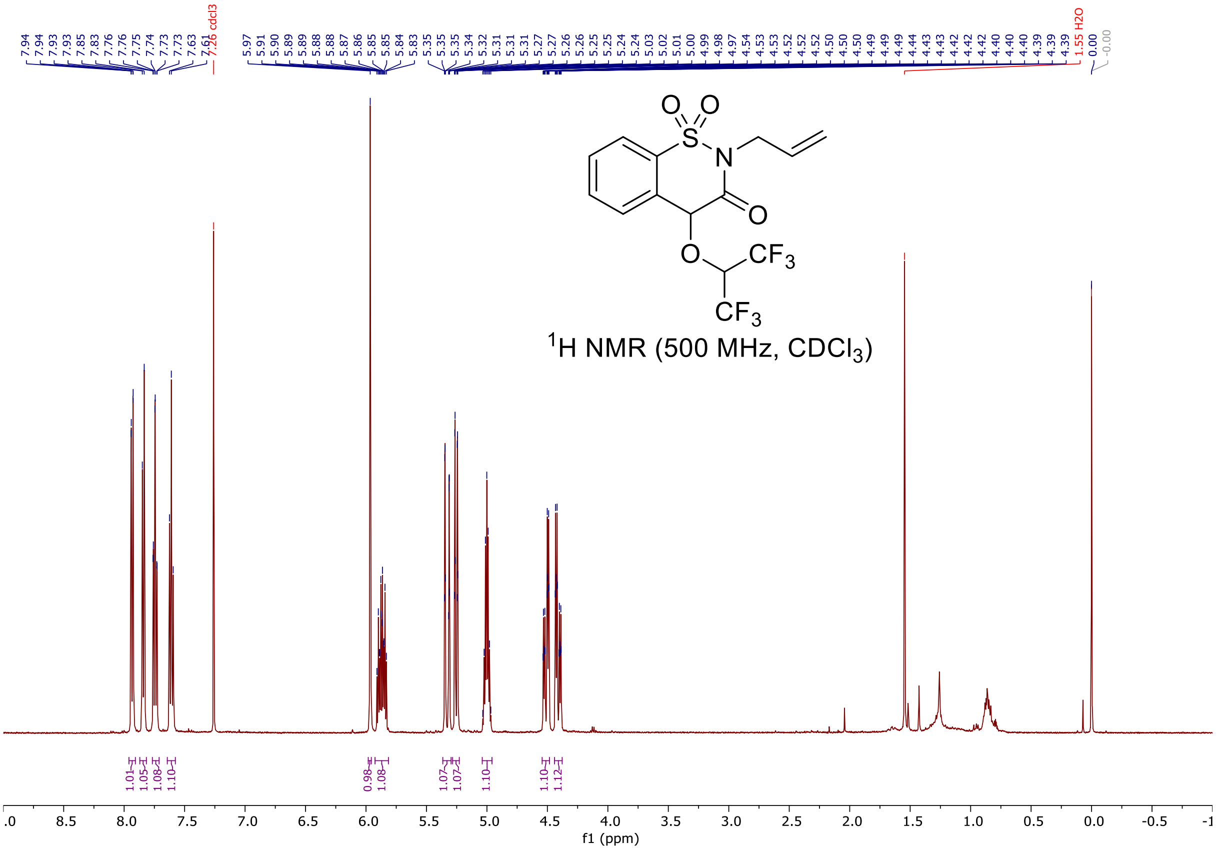

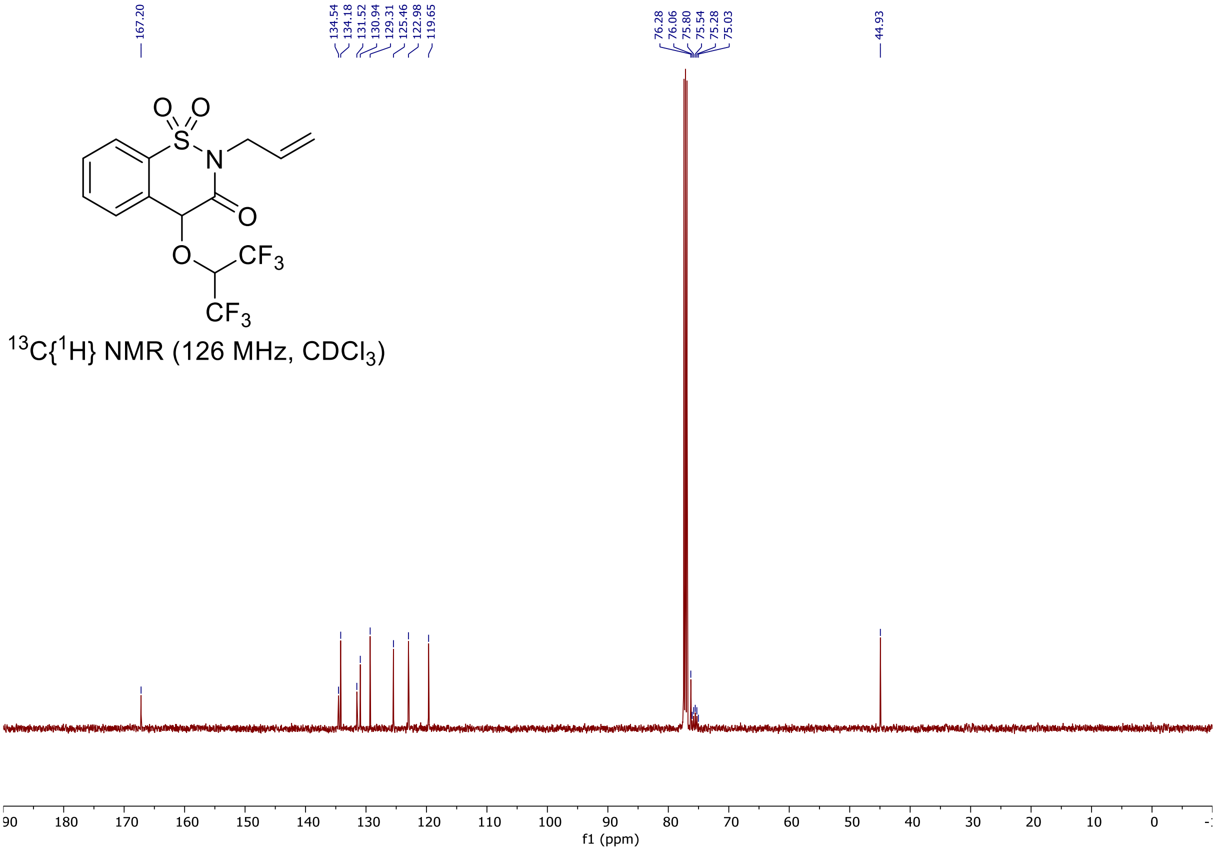

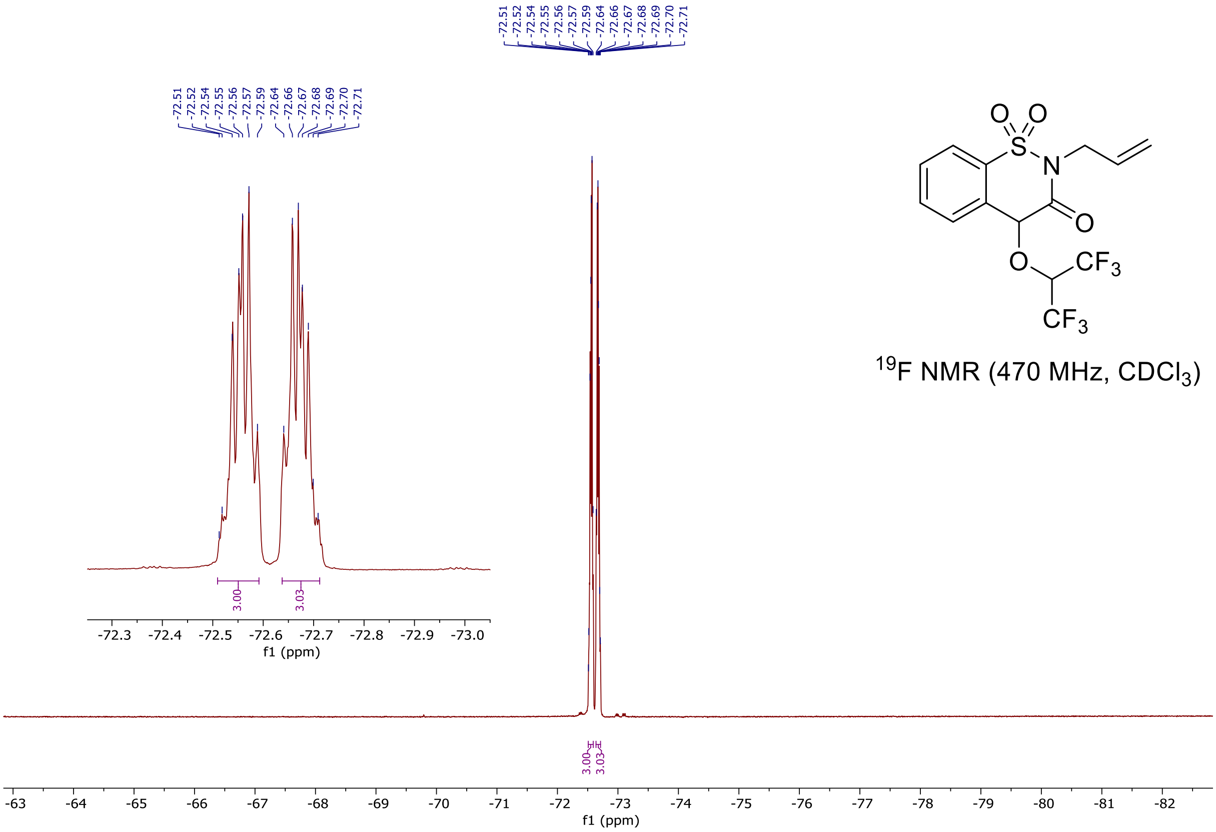

2-methyl-4-(2,2,3,3-tetrafluoropropoxy)-2H-benzo[*e*][1,2]thiazin-3(4*H*)-one 1,1-dioxide (**38**)

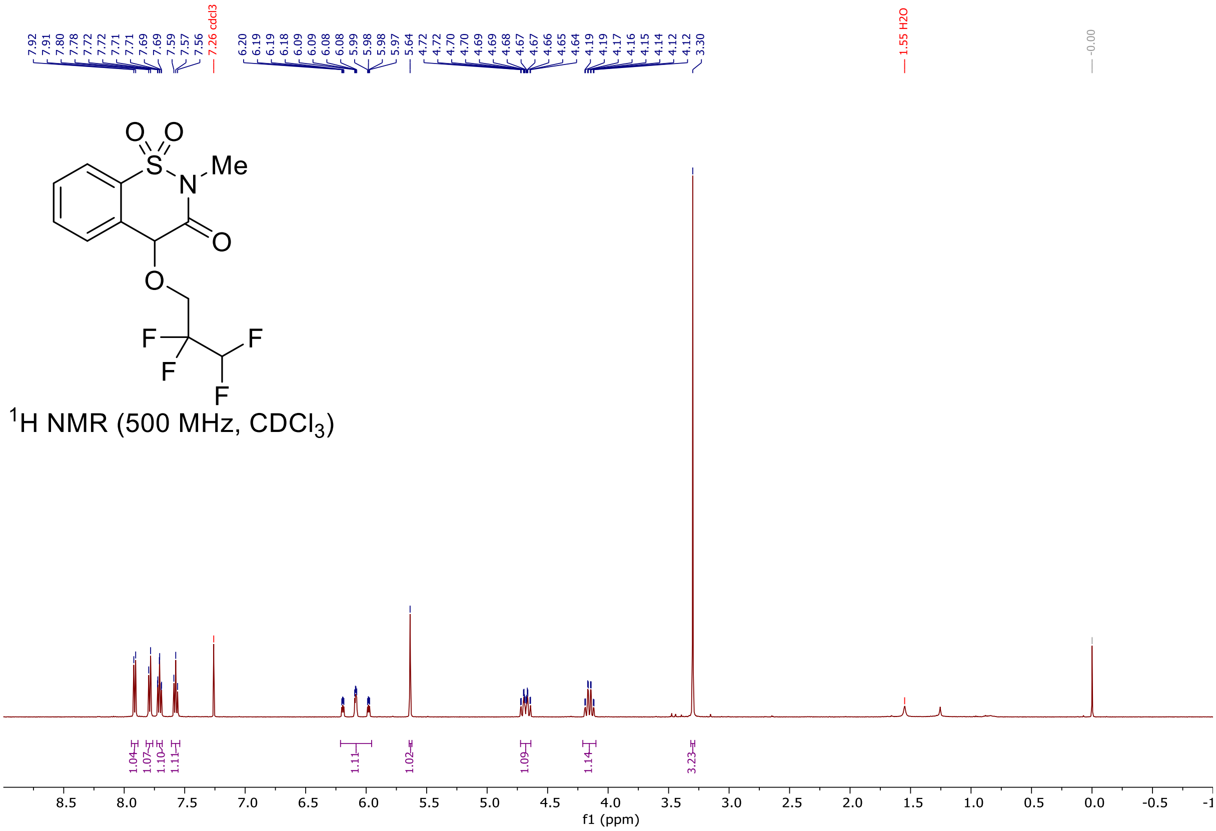

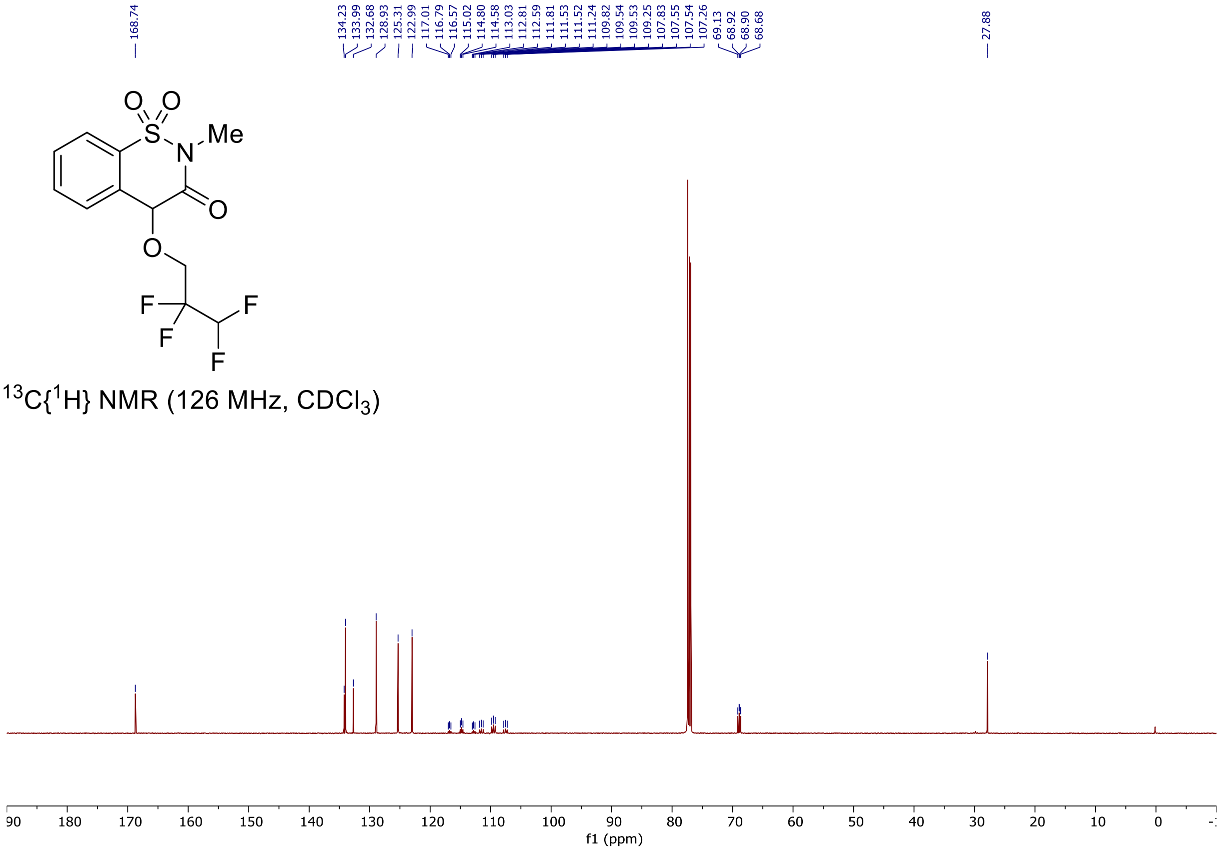
13C{1H} NMR (126 MHz, CDCl3)
f1 (ppm)

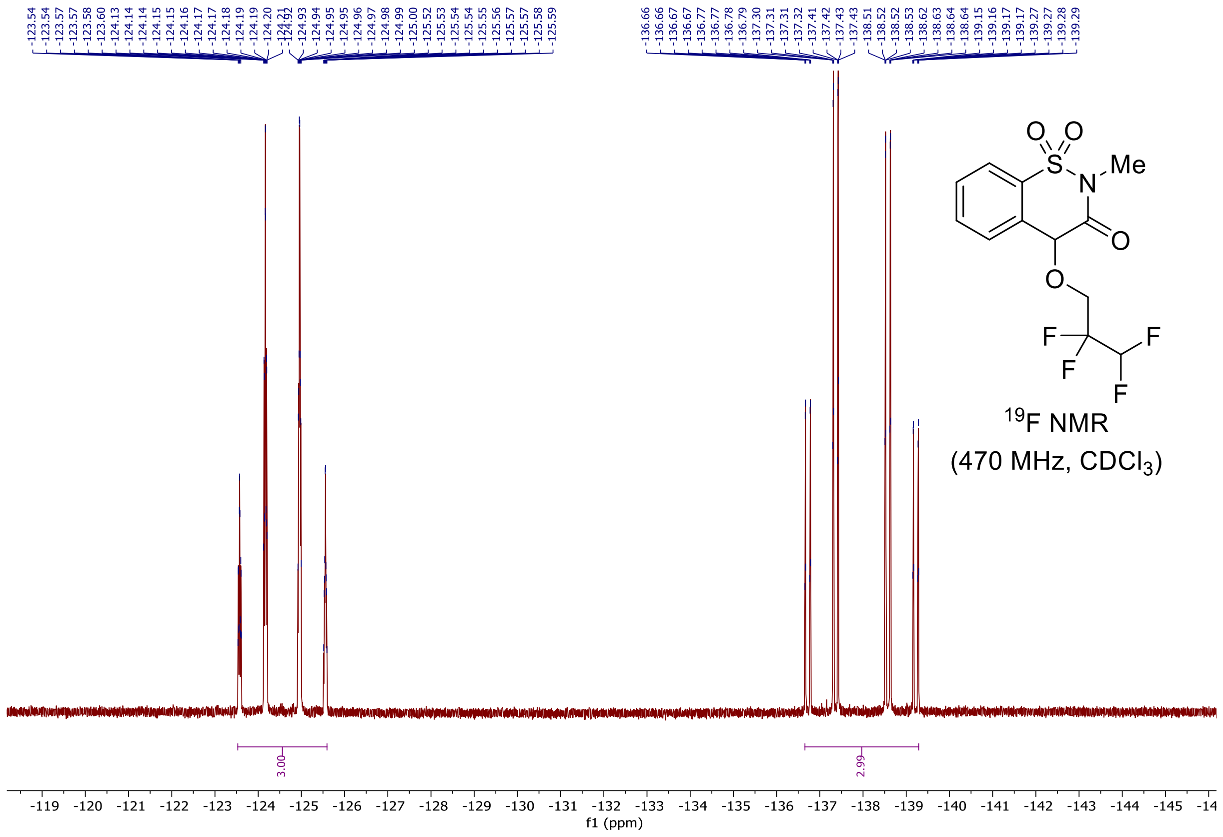
19F NMR
(470 MHz, CDCl3)
f1 (ppm)

4-(cyclohexylthio)-2-methyl-2H-benzo[*e*][1,2]thiazin-3(4*H*)-one 1,1-dioxide (**39**)

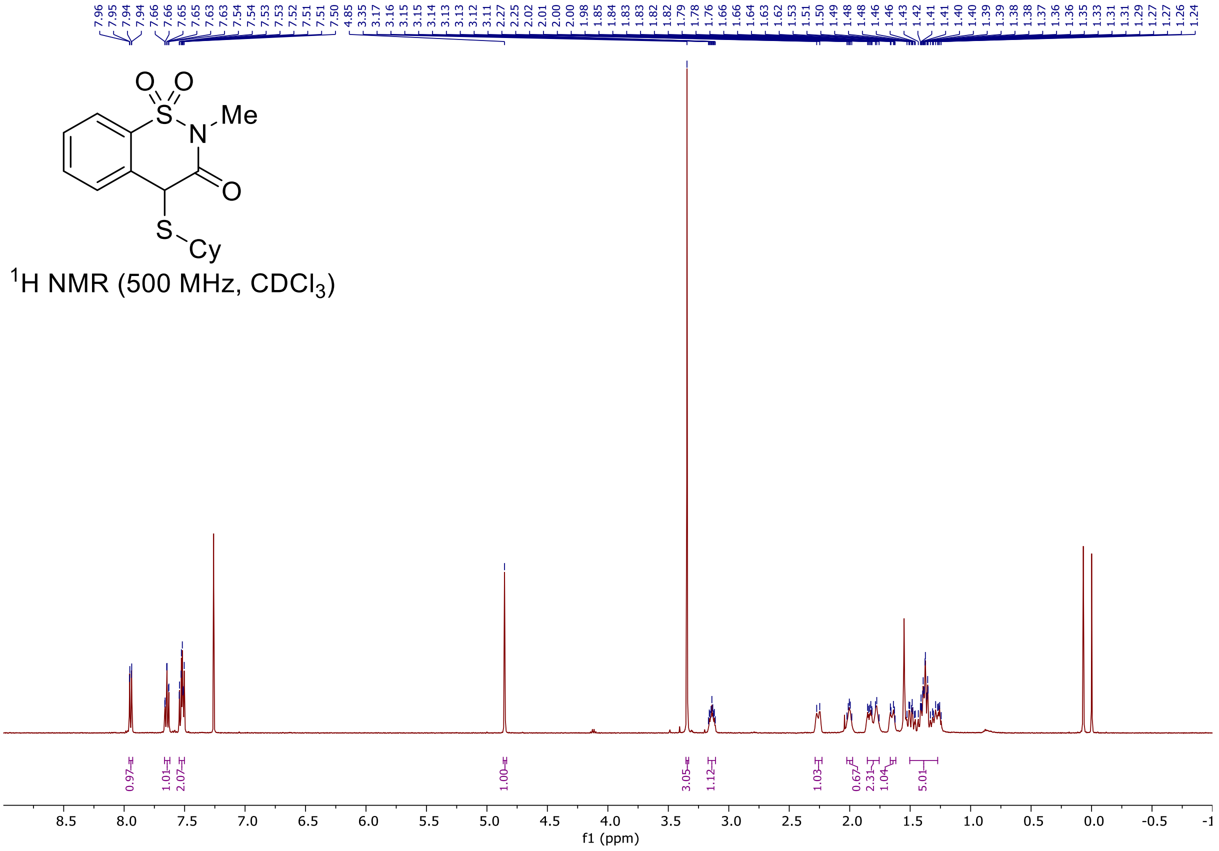

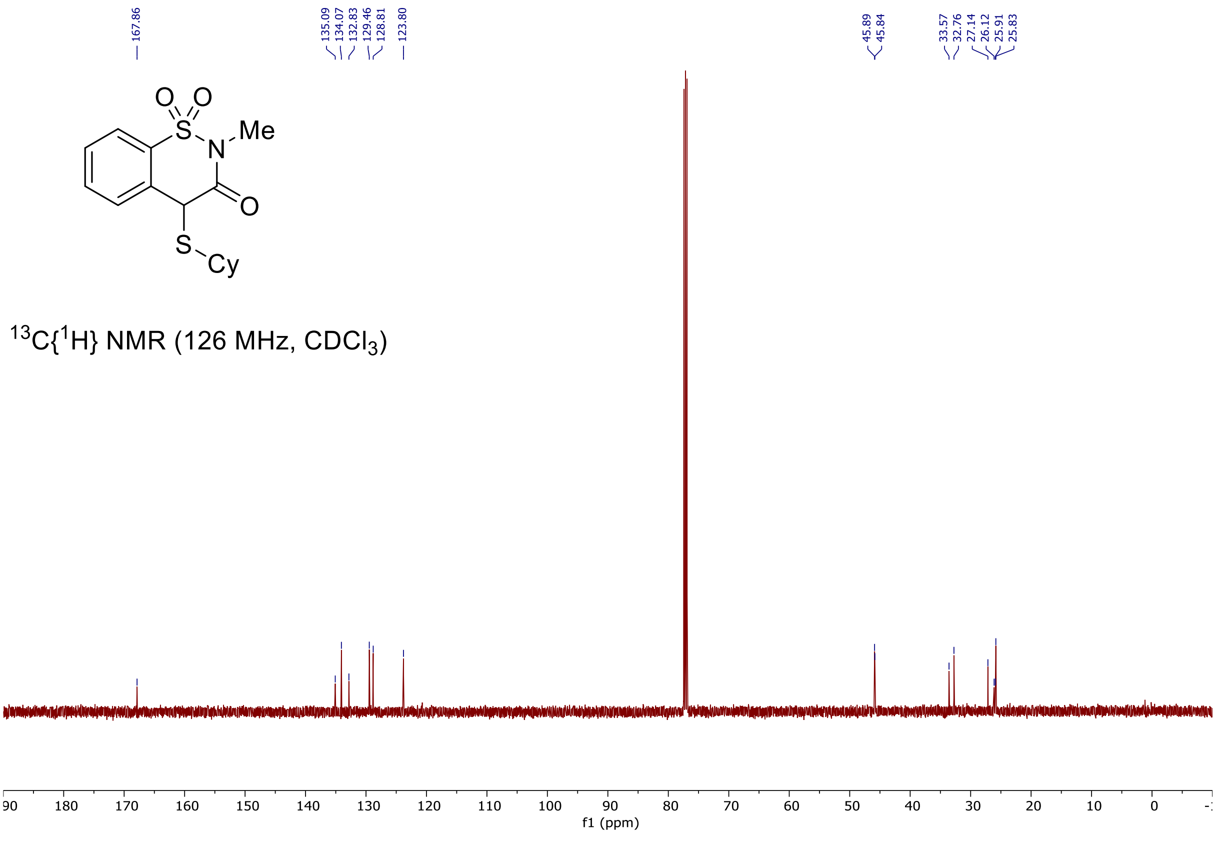

Supplement: Supplementary file 1 [file gg5c00017_si_001.pdf]
